# Mild N‑Arylation of Sulfoximines With (Hetero)aryl Chlorides Enabled by α‐Methylnaphthyl‐*t*BuBrettPhos Palladium Triflate

**DOI:** 10.1002/chem.70908

**Published:** 2026-04-08

**Authors:** Sourav Manna, Mahima Pilania, Kumarjit Sen, Nikolaos V. Tzouras, Angelino Doppiu, Lukas J. Gooßen

**Affiliations:** ^1^ Faculty of Chemistry and Biochemistry Ruhr‐Universität Bochum Bochum Germany; ^2^ Precious Metals Chemistry Umicore AG & Co. KG Hanau Germany

**Keywords:** arylation, catalysis, cationic complexes, palladium, sulfoximines

## Abstract

N‐Arylated sulfoximines are privileged motifs in pharmaceuticals, natural products, and chiral ligands, yet their synthesis remains challenging. We report a readily accessible, air‐ and moisture‐stable precatalyst, [Pd(1‐MeNAP)(*t*BuBrettPhos)]OTf, that promotes the N‐arylation of NH‐sulfoximines with exceptional efficiency. The mild conditions (Cs_2_CO_3_, 25°C) enable a broad substrate scope, tolerating e.g. aryl chlorides with free NH, OH, and COOH groups and sensitive, drug‐like heterocycles, thereby allowing late‐stage functionalization beyond current systems. Mechanistic studies reveal that the methylnaphthyl substituent and non‐coordinating triflate counterion cooperatively accelerate precatalyst activation via transmetalation/reductive elimination, generating the active monoligated Pd(0) species even in the presence of weakly nucleophilic sulfoximines.

## Graphical abstract

A readily accessible, air‐ and moisture‐stable palladium precatalyst, [Pd(1‐MeNAP)(*t*BuBrettPhos)]OTf, enables the direct N‐arylation of NH‐sulfoximines with (hetero)aryl chlorides under exceptionally mild conditions ideal for late‐stage diversification of drug‐like scaffolds. The methylnaphthyl substituent and non‐coordinating triflate counterion cooperatively promote rapid, spontaneous precatalyst activation already at room temperature.

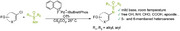

## 1 Introduction

**SCHEME 1:**
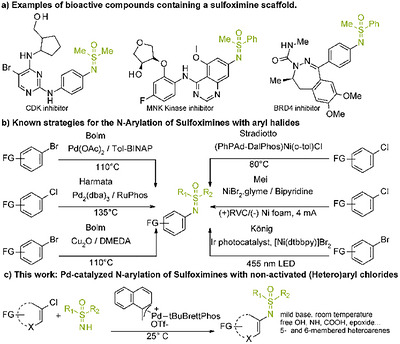
Strategies for N‐arylation of sulfoximines.

The sulfoximine functionality has emerged as a versatile motif in contemporary chemical and medicinal research, owing to its dual capacity to act as both a hydrogen‐bond donor and acceptor. This property renders sulfoximines particularly attractive in the design of biologically active molecules [1]. Indeed, N‐aryl sulfoximines constitute key structural elements in commercial drugs, chiral auxiliaries, asymmetric ligands, and pseudopeptide frameworks (Scheme 1a) [2, 3, 4, 5]. Consequently, efficient and broadly applicable synthetic entries to this compound class remain in high demand.

In the pursuit of late‐stage diversification of complex, drug‐like molecules, the catalytic N‐arylation of NH‐sulfoximines with readily available (hetero)aryl electrophiles represents a particularly appealing approach (Scheme 1b). It was pioneered by Bolm and coworkers [6], who demonstrated a Pd‐catalyzed C–N cross‐coupling between aryl bromides and NH‐sulfoximines using Pd(OAc)_2_/(tol)BINAP at 110°C. Subsequent developments employing Pd [7, 8, 9, 10, 11, 12, 13, 14, 15, 16, 17, 18], Cu [19, 20, 21, 22, 23, 24, 25, 26, 27, 28, 29, 30], Ni [31], and Fe [32] catalysts have broadened the scope of this transformation; however, these protocols typically require high catalyst loadings and elevated temperatures. More recently, photochemical [33, 34, 35] and electrochemical [36, 37] variants have emerged, yet these methods rely on specialized equipment and often remain substrate‐specific. While the Chan–Evans–Lam coupling proceeds under favorable, mild conditions, its practical utility is hindered by reliance on expensive and limited boronic acid reagents [38, 39].

From both synthetic and sustainability perspectives, the direct use of abundant, inexpensive, yet low‐reactivity (hetero)aryl chlorides as aryl sources is particularly desirable. Despite this clear incentive, only two catalyst systems have been reported to date that enable such couplings. Stradiotto and coworkers [31] achieved N‐arylations of NH‐sulfoximines with a limited range of (hetero)aryl chlorides using (PhPAd‐DalPhos)Ni(o‐tol)Cl at 80°C, while Harmata [13] and Chen [16] reported related Pd‐catalyzed variants with RuPhos and BrettPhos ligands, at temperatures well above 100°C. The development of a general, mild, and operationally simple catalytic system for N‐arylation of NH‐sulfoximines with (hetero)aryl chlorides therefore remains an unmet challenge.

We herein describe the rational development of an α‐methylnaphthyl (MeNAP) substituted palladium precatalyst that enables N‐arylation of NH‐sulfoximines with diversely functionalized aryl and heteroaryl chlorides under exceptionally mild conditions (rt, Cs_2_CO_3_) as depicted in Scheme 1c.

## 2 Results and Discussion

**SCHEME 2:**
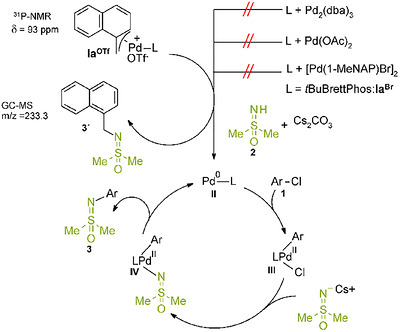
Catalyst activation and catalytic cycle of sulfoximination.

Our design rationale is summarized in Scheme 2. The C–N cross‐coupling cycle likely involves oxidative addition (**II→III**), salt metathesis (**III→IV**), and reductive elimination (**IV→II**) steps. Because monoligated Pd(0) complexes with Buchwald‐type ligands—such as the RuPhos system used by Harmata—readily activate aryl chlorides at room temperature, oxidative addition (**II→III**) is unlikely to limit turnover. Likewise, reductive elimination (**IV→II**) should be facile for weakly nucleophilic sulfoximines [40]. The salt metathesis step (**III→IV**) appeared to be the only potentially slow transformation within the catalytic cycle.

However, we reasoned that the true limitation of known systems might precede the catalytic cycle altogether—namely, the inefficient formation of the active L–Pd(0) species **II** under sulfoximination conditions. In the absence of strongly nucleophilic or reducing nucleophiles, Pd(II) precursors, such as Pd(OAc)_2_, [Pd(allyl)Cl]_2_, or [Pd(cinnamyl)Cl]_2_ convert only sluggishly into monoligated Pd(II) intermediates and reduce poorly to Pd(0) [41, 42, 43]. The Pd_2_(dba)_3_ precursor introduces competition from the stabilizing dba ligand, impeding coordination of both the phosphine ligand and the sulfoximine [44, 45]. We therefore hypothesized that previously reported sulfoximinations require forcing conditions not because of a slow step within the catalytic cycle, but because the catalytically active Pd(0) species does not form efficiently.

Indeed, ^31^P NMR monitoring of reactions of Pd_2_(dba)_3_ or Pd(OAc)_2_ with *t*BuBrettPhos, sulfoximine **2a,** and Cs_2_CO_3_ at room temperature revealed mostly the signal for non‐coordinated ligand (33.4 ppm), confirming that monoligated complexes do not readily form under these conditions. MeNAP–Pd precursors have been shown to form monoligated Pd(II) complexes much more readily, even with bulky ligands [46]. Reaction of [Pd(1‐MeNAP)Br]_2_ with *t*BuBrettPhos under these conditions indeed afforded [Pd(1‐MeNAP)(*t*BuBrettPhos)Br] (**Ia^Br^**, 83** **ppm in ^31^P‐NMR), albeit slowly (16 h). However, **Ia^Br^** turned out to be unreactive toward **2a**/Cs_2_CO_3_, with no formation of **II** being detected (Scheme S1).

To facilitate salt metathesis, we replaced the bromide in **Ia^Br^** with a non‐coordinating triflate by treating it with silver triflate, yielding [Pd(1‐MeNAP)(*t*BuBrettPhos)]OTf (**Ia^OTf^**). When stirred with **2a**/Cs_2_CO_3_, its ^31^P NMR signal at 93 ppm decreased while new resonances at 71 and 60 ppm appeared, consistent with formation of Pd(0) species [46], which slowly decomposed with formation of free ligand and Pd black. No Pd–sulfoximine intermediate was detected, suggesting rapid reductive elimination following salt exchange. After 2 h, GC–MS analysis revealed the reductive‐elimination product **3′** in a 10:1 ratio relative to methylnaphthalene (the hydrolysis product of **Ia^OTf^**), suggesting that **Ia^OTf^** converts into the catalytically active Pd(0) species **II** already at room temperature. Under identical conditions, the analogous cinnamyl precursor [Pd(cinnamyl)(*t*BuBrettPhos)]OTf afforded the two products only a 1:1 product ratio (Scheme S2). The superior reactivity of the MeNAP complex likely arises from aromatization to naphthalene upon η^3^→η^1^ transition, providing a thermodynamic driving force for catalyst activation [47, 48, 49, 50].

Having established that MeNAP–Pd triflate complexes convert into active Pd(0) species under non‐reducing sulfoximination conditions, we next examined whether this ease of activation translates into superior catalytic performance. This was probed using the model coupling of 4‐chloroanisole (**1a**) with dimethylsulfoximine (**2a**), systematically evaluating different catalysts, bases, and solvents (Table 1). We began with the RuPhos ligand, as this had been employed by Harmata in the state‐of‐the‐art sulfoximination protocol. Under the desired mild conditions (room temperature, Cs_2_CO_3_ as base), no conversion was observed within 16 h when using Pd(OAc)_2_, Pd_2_(dba)_3_, or [Pd(1‐MeNAP)Br]_2_ as precursors. To our delight, the preformed [Pd(1‐MeNAP)(RuPhos)]OTf complex afforded the desired product **3aa**, albeit in low yield. A systematic study of preformed complexes with various other ligands revealed [Pd(1‐MeNAP)(*t*BuBrettPhos)]OTf to be most effective, providing **3aa** in near‐quantitative yield (entry 6).

**TABLE 1:** Effects of Pd‐precursors, ligands, and bases^a^.

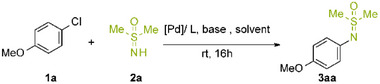					
#	Catalyst	Base	Solvent	Additive	Yield of 3aa (%)
1	Pd(OAc)_2_ + Tol‐Binap	Cs_2_CO_3_	Toluene	—	0
2	Pd_2_(dba)_3_ + RuPhos	„	„	—	0
3	[Pd(1‐MeNAP)Br]_2_ + RuPhos	„	„	—	0
4	[Pd(1‐MeNAP)(RuPhos)]OTf	„	„	—	6
5	[Pd(1‐MeNAP)(BrettPhos)]OTf	„	„	—	18
6	[Pd(1‐MeNAP)(tBuBrettPhos)]OTf	„	„	—	> 99
7	Pd_2_(dba)_3_ + *t*BuBrettPhos	„	„	—	0
8	Pd(OAc)_2 + _ *t*BuBrettPhos	„	„	—	0
9	[Pd(allyl)(*t*BuBrettPhos)]OTf	„	„	—	27
10	[Pd(cinnamyl)(*t*BuBrettPhos)]OTf	„	„	—	67
11	*t*BuBrettphosPd‐G3	„	„	—	69
12	[Pd(1‐MeNAP)(*t*BuBrettphos)]Br	„	„	—	0
13	[Pd(1‐MeNAP)(*t*BuBrettPhos)]OTf	NaOH	„	—	46
14	„	K_2_CO_3_	„	—	84
15	„	K_3_PO_4_	„	—	95
16	„	Cs_2_CO_3_	MeCN	—	55
17	„	„	*i*‐PrOH	—	99
18	„	„	THF	—	99
19	„	„	*t*‐BuOH	—	99
20	„	„	Toluene	Air atm.	0
21	„	„	„	10 µL H_2_O	99
22	„	„	„	dba	0
23				β‐Methyl styrene	86
24	None	„	„	—	0

Abbreviation: dba, dibenzylideneacetone.

^a^ Conditions: 0.25 mmol **1a**, 1.20 equiv **2a**, 2.0 mol% [Pd], 2.0 mol % ligand, 1.40 equiv Cs_2_CO_3_, 2.0 mL toluene, 25°C, 16 h, yields determined by GC analysis using *n*‐hexadecane as internal standard.

Comparative experiments revealed that the *t*BuBrettPhos ligand is ineffective in combination with Pd(OAc)_2_ and Pd_2_(dba)_3_, and even preformed allyl and cinnamyl complexes gave only moderate yields (entries 7–10). Notably, a G3‐type Pd–*t*BuBrettPhos complex also showed inferior reactivity, although it is activated via intramolecular extrusion of carbazole‐a mechanism not dependent on strong nucleophiles. This observation aligns with reports noting a poisoning effect of carbazole [51, 52]. Moreover, the addition of dba or β‐methylstyrene significantly reduced the catalytic activity of complex **Ia^OTf^** (entries 22 and 23), demonstrating that such alkenes—unavoidably present when using conventional precatalysts—adversely affect C–N couplings with weak nucleophiles. Further studies revealed that K_3_PO_4_ and Cs_2_CO_3_ are the most effective bases. An extensive solvent screening revealed that the reaction shows remarkably little dependence on the solvent environment. It can be performed in “green” solvents such as isopropanol and *t*BuOH. The reaction is sensitive to oxygen but less affected by water (entries 20 and 21). In the absence of Pd, no reaction occurs (entry 24).

The comparative data helps to understand why RuPhos had previously been identified as the “optimal” ligand in conventional screenings: at elevated temperatures, RuPhos at least partially forms active monoligated species from Pd(OAc)_2_, whereas bulkier ligands such as *t*BuBrettPhos do not, preventing their catalytic potential from being recognized. The present findings therefore highlight a general principle: in many other cross‐coupling reactions, ligand selection may have been similarly biased by catalyst activation efficiency rather than true catalytic competence [53, 54, 55]. By decoupling activation from catalysis using readily activated monoligated MeNAP–Pd triflate precatalysts, it should now be possible to identify ligands that are genuinely optimal for turnover, not merely those that happen to form active species under forcing conditions.

The one‐component pre‐catalyst [Pd(1‐MeNAP)(*t*BuBrettPhos)]OTf (**Ia^OTf^**) is easily accessible by stirring a mixture of [Pd(1‐MeNAP)Br]_2_ with AgOTf for 30 min at room temperature, followed by the addition of *t*BuBrettPhos [41]. This way, **Ia^OTf^** was isolated in 94% yield as an air‐ and moisture‐stable yellow solid. Single‐crystal x‐ray diffraction analysis revealed a η^3^‐bound MeNAP fragment, with long Pd─C bonds (2.109(3)–2.441(3) Å) and an outer sphere triflate counterion. In contrast to the corresponding bromide complex, no insertion of the ligand into the Pd‐MeNAP bond is observed.

With this thoroughly optimized catalyst system in hand, we next explored the synthetic scope of the transformation. Using Cs_2_CO_3_ as a base and toluene as a solvent, reactions were performed for 16 h at room temperature with **Ia^OTf^** as a catalyst.

The scope with respect to aryl halides was investigated using dimethylsulfoximine (**2a**) as a coupling partner. As shown in Table 2, the reaction proceeds with consistently high yields across electron‐rich and electron‐deficient aryl chlorides and tolerates substituents in ortho, meta, and para positions (**3aa**–**3xa**). Even the highly electron‐rich *o,p*‐dimethoxychlorobenzene reacts smoothly at room temperature (**3ta**), and only substrates bearing two ortho substituents require gentle heating for full conversion (**3sa**). The scalability of the reaction was confirmed by a gram‐scale synthesis of **3aa** in 94% yield without chromatographic purification. The reaction is not limited to aryl chlorides: expectedly, aryl bromides and aryl triflates react even faster, affording products in near‐quantitative yield. The catalysts display remarkable chemoselectivity: when both OTf and Cl groups are present, the triflate reacts selectively, leaving a handle for diversification by follow‐up cross‐couplings (**3oa**).

**TABLE 2:** Scope of the reaction^a^.

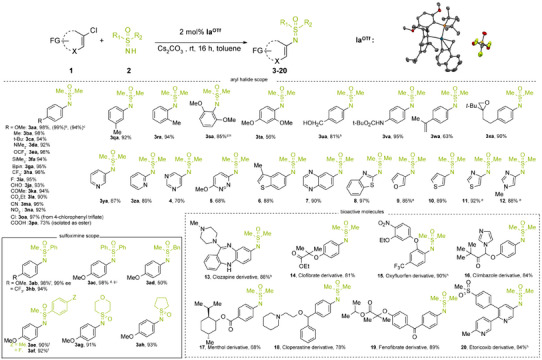

^a^ Conditions: 0.50 mmol **1**, 1.20 equiv **2**, 2.0 mol % [Pd(1‐MeNAP)(*t*BuBrettPhos)]OTf, 1.40 equiv Cs_2_CO_3_, 3.0 mL toluene, 25 °C, 16 h. Isolated yields. The X‐ray molecular structure of complex IaOTf is presented, showing thermal displacement ellipsoids at the 50% probability level, and hydrogen atoms are omitted for clarity (CCDC: 2501824) [56].

^b^ With ArBr, ArOTf.

^c^ Gram scale reaction.

^d^ 100 °C.

^e^ Heteroaryl bromide was used.

^f^ 20 h.

^g^ [Pd(1‐MeNAP)(BrettPhos)]OTf was used.

^h^ *t*BuOH was used as a solvent.

^i^ K_3_PO_4_ as base and dioxane as solvent.

The exceptionally mild conditions translate into a remarkable functional‐group tolerance, accommodating aldehyde, ester, ketone, nitro, epoxide, and even unprotected OH, NH, COOH, and acidic C–H functionalities (**3ja–3xa**). The compatibility of SiMe_3_ and BPin substituents makes the transformation orthogonal to Suzuki–Miyaura and Hiyama couplings, thereby expanding its synthetic utility (**3fa–3ga**).

A key strength of this protocol lies in its broad applicability to heteroaryl substrates. Five‐membered heterocycles such as thiazoles, imidazoles, furans, and thiophenes (**9**–**12**) as well as six‐membered systems including pyridines, pyrimidines, benzothiophenes, quinoxalines, pyridazines, and benzothiazoles (**3ya**–**3za**, **4**–**8**) all undergo efficient coupling. To the best of our knowledge, no previously reported method offers comparable breadth, particularly for drug‐like heterocycles, underscoring the suitability of this approach for late‐stage functionalization. This potential is exemplified by successful couplings of complex bioactive molecules containing aryl chloride motifs, including clozapine, oxyfluorfen, climbazole, cloperastine, and etoricoxib (**13–20**).

The scope of sulfoximine substrates was then examined using 4‐chloroanisole (**1a**) as the aryl partner and was found to extend to various diaryl, alkyl‐aryl, and dialkyl NH–sulfoximines (**3ab–3ah**). Notably, the coupling of enantiopure NH‐methylphenylsulfoximine with 4‐chloroanisole proceeded without loss of optical purity, underscoring the exceptional mildness of the new catalytic system (*S*)**‐3ab**.

**SCHEME 3:**
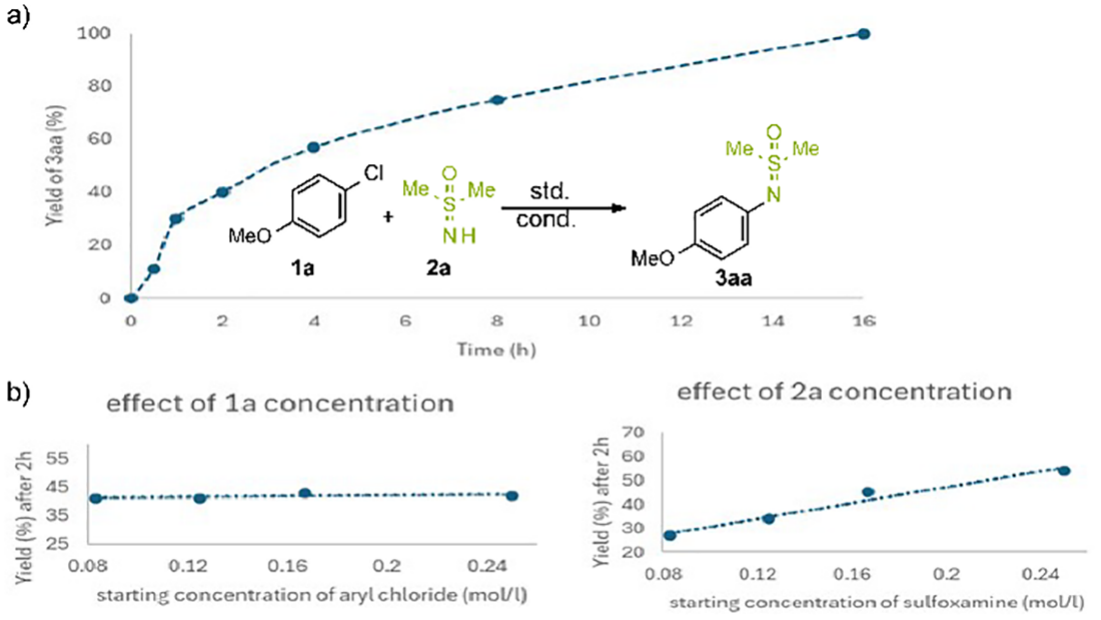
(a) Kinetic plot (b) Effect of sulfoximine and aryl chloride concentrations on the reaction rate.

Several experiments were conducted to elucidate the reaction mechanism. The kinetic profile of the reaction exhibits a nearly exponential progression with only negligible sigmoidal induction period, indicating that catalyst activation is no longer rate‐limiting (Scheme 3a). Variation of the aryl chloride **1a** concentration had no significant effect on the reaction rate, ruling out oxidative addition as the turnover‐limiting step (Scheme 3b). In contrast, increasing the concentration of sulfoximine **2a** accelerated the reaction, consistent with a rate‐determining transmetallation step. A one‐pot competitive coupling of **2a** with an electron‐deficient aryl chloride (**1** **h**) and an electron‐rich analogue (**1a**) revealed preferential conversion of **1** **h**, while in separate parallel reactions, both substrates reacted at comparable rates. This behavior indicates that oxidative addition, facilitated by electron‐withdrawing substituents, is irreversible under the reaction conditions. The competition experiments give further evidence that neither the oxidative addition nor the reductive elimination is rate‐determining overall (Scheme S3). To probe the influence of the counterion on the ligand insertion–deinsertion equilibrium of the menap complex, we interconverted the bromide and triflate complexes **Ia^Br^** and **Ia^OTf^**. In the triflate complex [Pd(1‐MeNAP)(*t*BuBrettPhos)]OTf, the MeNAP fragment adopts the expected η^3^‐coordination while the arene ring of the ligand remains fully aromatic. Upon addition of NaBr, the bromide analogue **Ia^Br^** forms, in which the arene ring of the ligand inserts into the Pd–C bond of the MeNAP fragment, resulting in dearomatization of the phosphine arene and formation of a *σ*‐bond. Subsequent addition of AgOTf cleanly regenerated **Ia^OTf^**, with its η^3^‐coordinated MeNAP substituent, as verified by ^3^ ^1^P NMR spectroscopy. Subsequent addition of NaBr to the same solution reversed the transformation, yielding **Ia^Br^** with its dearomized *t*BuBrettPhos ligand.

**SCHEME 4:**
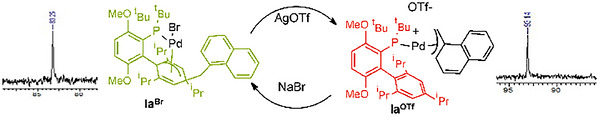
Counterion governs the structural preference.

These results demonstrate that the arene insertion is fully reversible and dictated by the coordinating strength of the counterion: coordinating halides favor the dearomatized form, while weakly coordinating triflate stabilizes the catalytically competent η^3^‐complex (Scheme 4). Since the de‐aromatization of the ligand resembles an off‐cycle equilibrium, it provides an additional explanation for the higher catalytic activity of the triflate‐substituted complex **Ia^OTf^** in comparison to **Ia^Br^**.

## 3 Conclusion

In conclusion, [Pd(1‐MeNAP)(*t*BuBrettPhos)]OTf enables the N‐arylation of NH‐sulfoximines with an exceptionally broad range of (hetero)aryl chlorides under mild, operationally simple conditions, making it ideally suited for late‐stage functionalization. The swift formation of catalytically active monoligated Pd(0) species from MeNAP complexes allows ligand optimization based on true catalytic competence rather than activation efficiency, a concept that promises to unlock new opportunities in many related cross‐coupling reactions.

## Conflicts of Interest

Some of the authors (S.M., N.V.T., A.D and L.J.G.) have filed a patent related to the Pd‐methylnaphthyl complexes described in this article.

## Supporting information




**Supporting File**: chem70908‐sup‐0001‐SuppMat.pdf.

## Data Availability

The data that support the findings of this study are openly available in the sciflection repository (https://identifiers.org/sciflection:8edaaf47‐8a83‐479d‐a004‐9c533d6dc460).
