# Supplementary material for: Mild N‑Arylation of Sulfoximines With (Hetero)aryl Chlorides Enabled by α‐Methylnaphthyl‐tBuBrettPhos Palladium Triflate
Source: Chemistry. 2026 Apr 8;32(23):e70908. doi: 10.1002/chem.70908 (PMC13282904; doi:10.1002/chem.70908)
Supplement: Supplementary file 1 — Supporting File: chem70908‐sup‐0001‐SuppMat.pdf. [file CHEM-32-e70908-s001.pdf]

Supporting Information

Mild N-Arylation of Sulfoximines with (Hetero)aryl Chlorides Enabled by α-Methylnaphthyl-tBuBrettPhos Palladium Triflate

Sourav Manna,[a] Mahima Pilania,[a] Kumarjit Sen,[a] Nikolaos V. Tzouras,[a] Angelino Doppiu,[b] and Lukas J. Gooßen*[a]

[a]Faculty for Chemistry and Biochemistry, Ruhr Universität Bochum, Universitätsstr. 150, 44801 Bochum, Germany

[b]Precious Metals Chemistry, Umicore AG & Co. KG, Rodenbacher Chaussee 4, 63457 Hanau-Wolfgang, Germany.

*E-mail: lukas.goossen@rub.de

Abstract: N-Arylated sulfoximines are privileged motifs in pharmaceuticals, natural products, and chiral ligands, yet their synthesis remains challenging. We report a readily accessible, air- and moisture-stable precatalyst, [Pd(1-MeNAP)(*t*BuBrettPhos)]OTf, that promotes the N-arylation of NH-sulfoximines with exceptional efficiency. The mild conditions (Cs_2CO_3, 25 °C) enable a broad substrate scope, tolerating e.g. aryl chlorides with free NH, OH and COOH groups and sensitive, drug-like heterocycles, thereby allowing late-stage functionalization beyond current systems. Mechanistic studies reveal that the methylnaphthyl substituent and non-coordinating triflate counterion cooperatively accelerate precatalyst activation via reductive elimination, generating the active monoligated Pd(0) species even in the presence of weakly nucleophilic sulfoximines.

Table of Contents

1. General information 2
2. General procedures 3
3. Screening tables of the reaction conditions and additional experiments 4
4. Mechanistic study 8
5. Synthesis and characterization of preformed [Pd(1-MeNAP)L]OTf complexes 10
6. Synthesis and Characterization of Products 18
7. NMR-Spectra of product 36
8. References 90
9. Author Contributions 90

1. General information

All reactions were performed in oven-dried glassware containing a Teflon-coated stirring bar and dry septum under argon atmosphere. Optimization reactions were monitored by GC analysis using n-hexadecane as internal standard. ^{1}H and ^{13}C{1H} NMR spectra were recorded on an Avance-III-300 or Avance-Neo-400 spectrometer at 25 °C if not stated otherwise. ^{19}F NMR and ^{31}P-NMR spectra were recorded on Spinsolve Benchtop NMR (MAGRITEK) spectrometers at 25 °C. All values of the chemical shift are in ppm regarding the δ-scale. To display multiplicities and signal forms correctly the following abbreviations were used: s = singlet, d = doublet, t=triplet, q = quartet, quin = quintet, m = multiplet, dd = doublet of doublet, br = broad signal. GC analyses were carried out using an HP-5 capillary column (Phenyl methyl siloxane, 30 m × 320 × 0.25, 100/2.3-30-300/3) using the following conditions: Carrier gas: N_2; Initial Temperature: 60 °C; Final Temperature: 300 °C; Rate: 30 °C·min−1 ; Hold Time: 8 min; Column Flow: 1.0 mL min−1 ; Detector: FID, Temperature FID: 330 °C. Column chromatography was performed on a CombiFlash Companion (Isco) and a Pure C-815 Flash (Büchi) using Reveleris packed columns (12 g or 40 g). Mass spectrometric data were acquired on a GC-MS Agilent 5977B MSD. HRMS analyses were acquired using a GC-MS system consisting of an Agilent 7250 GC/Q-TOF, in which ionization was achieved by EI, or an HPLC-MS system consisting of an UltiMate 3000 HPLC (ThermoFisher) with an EC 4/2 Universal RP pre-column (Macherey-Nagel), eluting at 40 °C with acetonitrile/water/formic acid (3:1:0.01) and a flow rate of 0.3 mL/min, coupled with a maXisCompact high-resolution mass spectrometer (Bruker Daltonik GmbH). The MS ionization was achieved by ESI. Chiral HPLC was performed on a Shimadzu LC-20AD using a chiral column (Chiralpak® IA, DaicelTM, 25 cm x 4.6 mm, 5µm, n-hexane: isopropanol 95:5 or 97.5:2.5, 0.5 mL/min). Infrared spectra were recorded on Bruker Vertex 70 Spectrometer with Universal ATR Sampling Accessory. Melting points were measured on a Mettler Toledo MP70. Commercial substrates were used as received unless otherwise stated. Solvents were dried over molecular sieves or obtained from the solvent-drying system (Braun SPS System) and stored over 3 or 4 Å molecular sieves. Molecular sieves were activated in the microwave prior to use. All solvents and liquid reagents were degassed by bubbling nitrogen through the solvent. Prior to use, Cs_2CO_3 is ground to a fine powder with a mortar and pestle, which has a notable impact on the reproducibility of the reaction. For the reaction using t-BuOH, the hot plate was maintained at 27–29 °C to keep the t-BuOH from freezing.Commercial substrates (aryl halides and sulfoximines) were used as received unless otherwise stated. Unless otherwise mentioned, phosphine ligands, imidazolium salts, Pd sources, and well-defined Pd pre-catalysts were purchased from commercial sources or were donated by UMICORE.

2. General procedures

General procedure of the C–N cross-coupling of Sulfoximines (GP-A)

A crimp cap vial equipped with a Teflon coated magnetic stirring bar was charged with [Pd(1-MeNAP)(*t*BuBrettPhos)]OTf] (2.0 mol%), aryl chloride (0.50 mmol, 1 equiv. if solid), sulfoximines (1.20 equiv. if solid). The vial was entered inside a glovebox, where Cs_2CO_3 (1.40 equiv.) [Prior to use, Cs_2CO_3 was ground to a fine powder with a mortar and pestle, which has a notable impact on the reproducibility of the reaction] was added. The vial was sealed, transferred out of the glovebox and aryl chloride (if liquid, 0.50 mmol, 1.0 equiv.) and sulfoximines (1.20 equiv. if liquid) was added *via* syringe under nitrogen unless mentioned otherwise. Then anhydrous toluene (or t-BuOH) (3 mL) was added sequentially via syringe. The reaction mixture was stirred for 16 h at room temperature. Afterwards, the reaction was quenched with few drops of MeOH, diluted with EtOAc and extracted with $NaHCO_3$, water and brine. The combined organic phase was dried over $MgSO_4$ and purified by automated flash column chromatography to yield the product.

General procedure of the C–N cross-coupling of Sulfoximines (GP-B)

A crimp cap vial equipped with a Teflon coated magnetic stirring bar was charged with [Pd(1-MeNAP)(*t*BuBrettPhos)]OTf] (2.0 mol%), aryl chloride (0.50 mmol, 1 equiv. if solid), sulfoximines (1.20 equiv. if solid). The vial was entered inside a glovebox, where Cs_2CO_3 (1.40 equiv.) [Prior to use, Cs_2CO_3 was ground to a fine powder with a mortar and pestle, which has a notable impact on the reproducibility of the reaction was added]. The vial was sealed, transferred out of the glovebox and aryl chloride (if liquid, 0.50 mmol, 1.0 equiv.) and sulfoximines (1.20 equiv. if liquid) was added *via* syringe under nitrogen unless mentioned otherwise. Then anhydrous toluene (or t-BuOH) (3 mL) was added sequentially via syringe. The reaction mixture was stirred for 16 h at room temperature. Afterwards, the reaction was quenched with few drops of MeOH, diluted with EtOAc/MeOH. After that reaction mixture was filtered through celite and combined with silica and solvents were removed using rotary evaporation. The resulting drypack was used in automated flash chromatography to yield the product.

3. Screening tables of the reaction conditions and additional experiments

Table S1: Screening of Pd source and ligand[a]

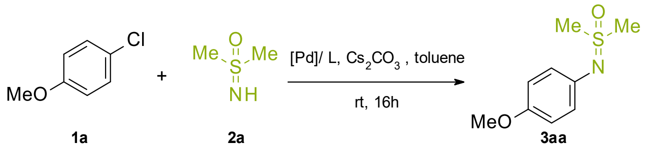

Entry	Pd source	ligand	Yield 3aa [%]
**1**	[Pd(1-menap) Br]$_2$	BrettPhos	0
**2**	„	RuPhos	0
**3**	„	^{t}BuBrettPhos	0
**4**	„	R-BINAP	0
**5**	„	P^{t}Bu$_3$.HBF$_4$	0
**6**	„	XPhos	0
**7**	„	KeYPhos	0
**8**	„	PinkYPhos	0
**9**	„	XanthPhos	0
**10**	„	N-XanthPhos	0
**11**	„	SPhos	0
**12**	„	CataCXium A	0
**13**	[Pd(cinnamyl)(BrettPhos)]OTf	-	2

[a]Reaction conditions: **1a** (0.250 mmol), **2a** (1.20 equiv.), [Pd] (2.0 mol%), ligand (2 mol%), Cs_2CO_3 (1.40 equiv.), toluene (2 mL). Yields were determined by GC using *n*-hexadecane as internal standard.

Table S2: Screening of base and solvent[a]

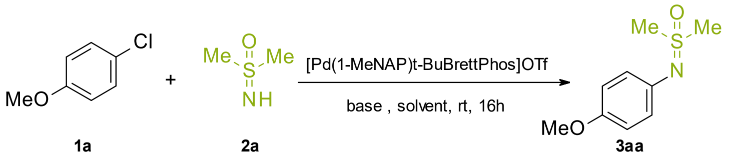

Entry	Base	solvent	Yield 3aa [%]
1	Cs_2CO_3	toluene	>99
2	NaO^{t}Bu	“	0
3	Cs_2CO_3	DMSO	0
4	„	Propylene carbonate	47
5	„	Ethylene glycol	0
6	„	1-Butanol	25
7	„	DCM	0
8	„	NMP	97

[a]Reaction conditions: **1a** (0.250 mmol), **2a** (1.20 equiv.), [Pd] (2.0 mol%), ligand (2 mol%), base (1.40 equiv.), solvent (2 mL). Yields were determined by GC using *n*-hexadecane as internal standard.

Gram scale reaction:

A crimp cap vial equipped with a Teflon coated magnetic stirring bar was charged with [Pd(1-MeNAP)(*t*BuBrettPhos)]OTf] (2.0 mol%), aryl chloride **1a** (6.0 mmol, 1 equiv), sulfoximines **2a** (1.20 equiv.). The vial was entered inside a glovebox, where Cs_2CO_3 (1.40 equiv.) was added. Then anhydrous toluene (30 mL) was added sequentially via syringe. The reaction mixture was stirred for 16 h at room temperature. Afterwards, the reaction was quenched with few drops of MeOH, diluted with EtOAc and extracted with 2 N NaOH, water and brine. The combined organic phase was dried over $MgSO_4$ and dried in high vacuum to yield (94%) the product **3aa**.

Crude GC of the reaction:

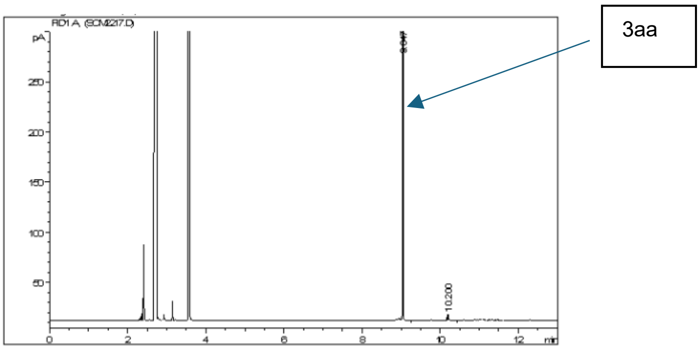

Area Percent Report

Sorted By : Signal
Multiplier : 1.0000
Dilution : 1.0000
Use Multiplier & Dilution Factor with ISTDs

Signal 1: FID1 A,

Peak #	RetTime [min]	Type	Width [min]	Area [pA*s]	Height [pA]	Area %
1	9.047	VV	0.0169	385.38129	347.23648	97.67911
2	10.200	VV	0.0248	9.15678	5.13263	2.32089

Totals : 394.53806 352.36911

^{1}H-NMR after NaOH wash:

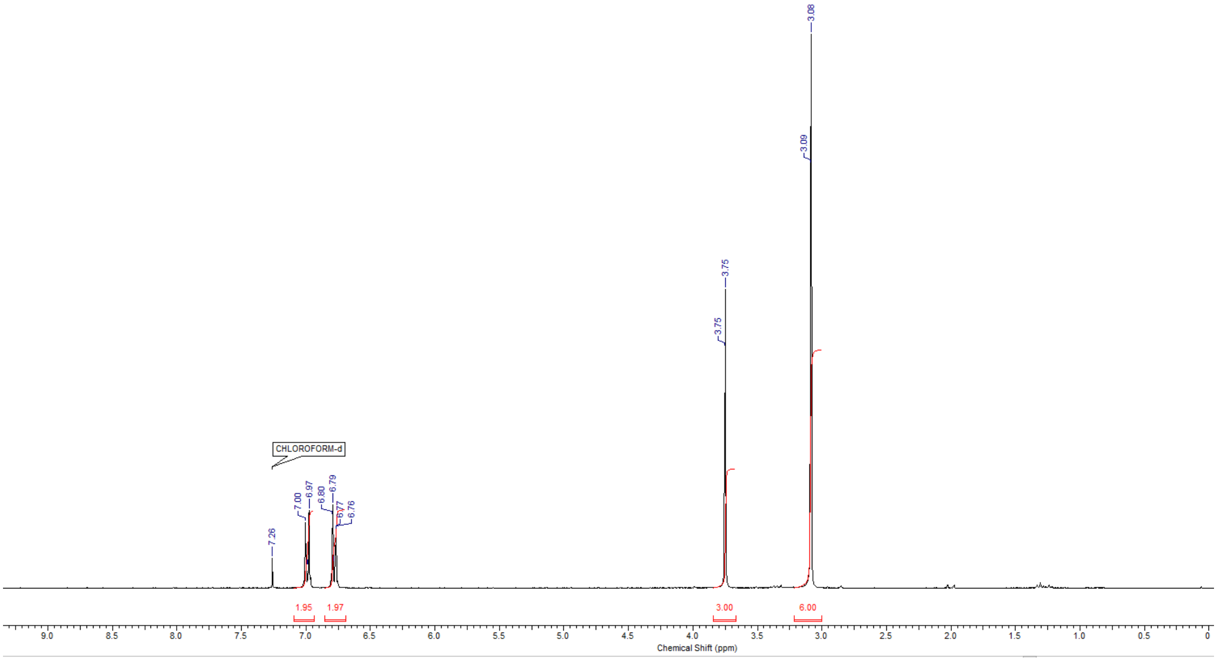

Reaction with chiral sulfoximine:

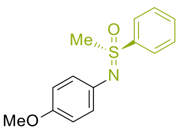

(S)-3ab

(S)-(4-methoxyphenyl)imino-methyl-oxo-phenyl-λ⁶-sulfane

A crimp cap vial equipped with a Teflon coated magnetic stirring bar was charged with [Pd(1-MeNAP)(*t*BuBrettPhos)]OTf] (2.0 mol%), (S)-imino-methyl-oxo-phenyl-λ⁶-sulfane **S-2b** (1.20 equiv.).[(S)-imino-methyl-oxo-phenyl-λ⁶-sulfane is prepared following the method report by Gais and coworkers[7]] .The vial was entered inside a glovebox, where Cs_2CO_3 (1.40 equiv.) was added. The vial was sealed, transferred out of the glovebox and aryl chloride (0.50 mmol, 1.0 equiv.) was added via syringe under nitrogen unless mentioned otherwise. Then anhydrous toluene (3 mL) was added sequentially via syringe. The reaction mixture was stirred for 18 h at room temperature. Afterwards, the reaction was quenched with few drops of MeOH, diluted with EtOAc and extracted with $NaHCO_3$, water and brine. The combined organic phase was dried over $MgSO_4$ and purified by automated flash column chromatography to yield the product (S)-**3ab**.

Chiral HPLC was performed on a Shimadzu LC-20AD using a chiral column (Chiralpak® IA, DaicelTM, 25 cm x 4.6 mm, 5µm, n-hexane: isopropanol 90:10, 1.5 mL/min).

For racemic **3ab**:

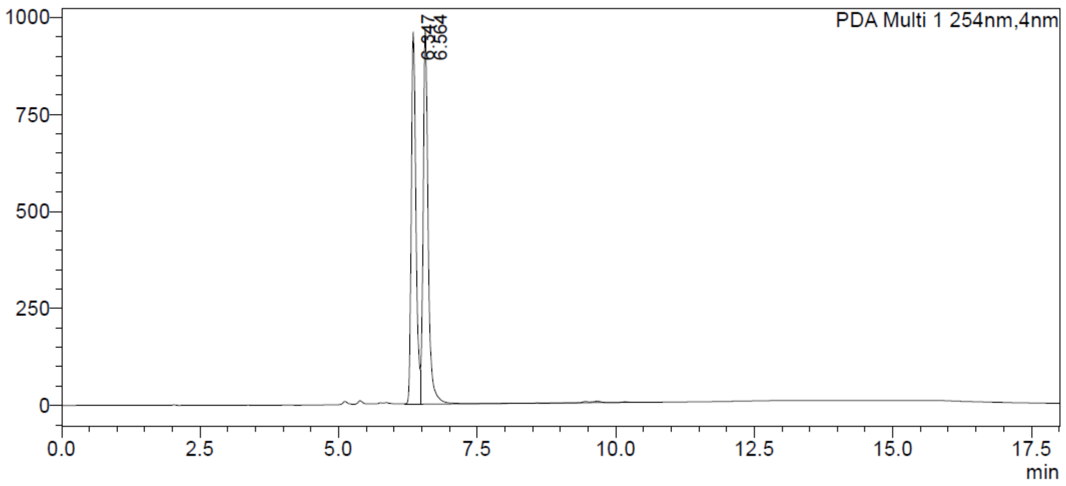

<Peak Table>

PDA Ch1 254nm

Peak#	Ret. Time	Area	Height	Area%
1	6.347	5601238	960134	48.085
2	6.564	6047445	965375	51.915
Total		11648683	1925508	100.000

For (S)-**3ab**:

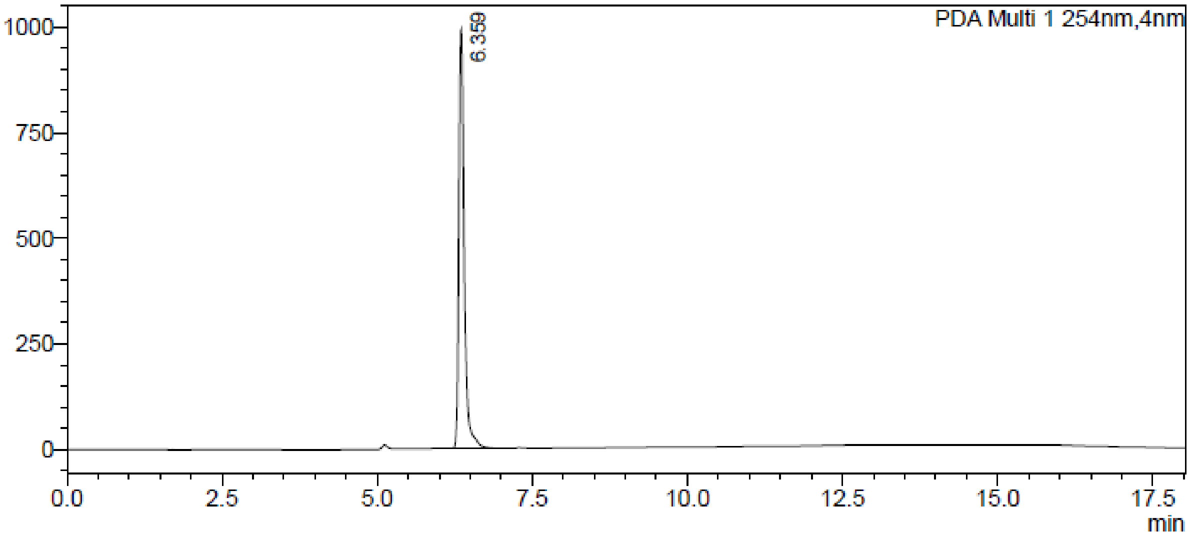

<Peak Table>

PDA Ch1 254nm

Peak#	Ret. Time	Area	Height	Area%
1	6.359	6088210	774025	100.000

Peak#	Ret. Time	Area	Height	Area%
Total		6088210	774025	100.000

4. Mechanistic study

^{31}P-NMR Study:

Pd precatalyst (1 equiv. 0.04 mmol) and ligand (0.04 mmol) or preformed monoligated complex (0.04 mmol) and sulfoximine (5.0 equiv.) were weighed into an oven-dry vial. The vial was entered inside a glovebox, where Cs_2CO_3 (6.0 equiv.) was added. The vial was sealed, transferred out of the glovebox, then anhydrous toluene (1.5 mL) was added sequentially via syringe. The reaction mixture was stirred for 2 h at room temperature and analysed via ^{31}P-NMR. (NMR samples are prepared in inert atmosphere.)

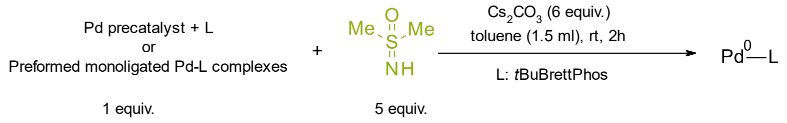

Pd(OAc)$_2$: Only the free ligand is observed; even the monoligated Pd(II) complex is not formed.

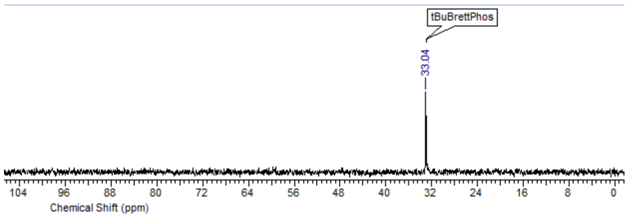

Pd$_2$(dba)$_3$: Mostly free ligand was observed; only a small amount of Pd(0)–L (it is coordinated with dba) complexes is visible.

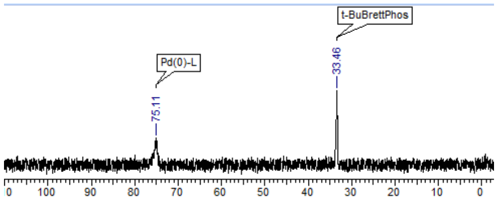

[Pd(1-MeNAP)(*t*BuBrettPhos)Br] : ^{31}P-NMR peak of [Pd(1-MeNAP)(*t*BuBrettPhos)Br] only present, no formation of Pd(0) complex or free ligand was observed.

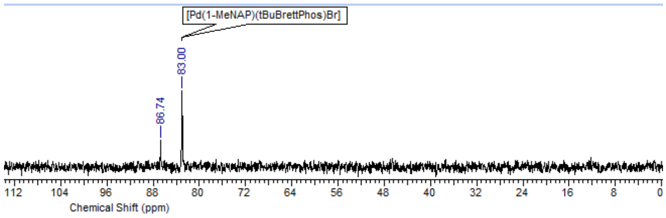

[Pd(1-MeNAP)(*t*BuBrettPhos)OTf] :

Complete disappearance of the ^{31}P-NMR resonance at 93 ppm, corresponding to [Pd(1-MeNAP)(*t*BuBrettPhos)OTf], was observed. The resulting spectrum exhibited a new peak at 60 ppm, which is characteristic of a Pd(0) species, owing to the rapid formation of Pd black, a signal corresponding to the free ligand appeared at 33 ppm.

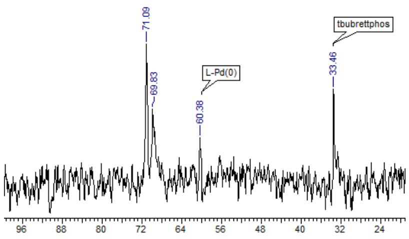

Scheme S1: ^{31}P-NMR Study

GC/GC-MS Study:

Pd precatalyst (1 equiv. 0.04 mmol) and ligand (0.04 mmol) or preformed monoligated complex (0.04 mmol) and sulfoximine (5.0 equiv.) were weighed into an oven-dry vial. The vial was entered inside a glovebox, where Cs_2CO_3 (6.0 equiv.) was added. The vial was sealed, transferred out of the glovebox, then anhydrous toluene (1.5 mL) and n-hexadecane (10µL) were added sequentially via syringe. The reaction mixture was stirred for 2 h at room temperature. Reaction was quenched by NH_4Cl then analysed by GC/GC-MS.

[Pd(1-MeNAP)(*t*BuBrettPhos)OTf]:

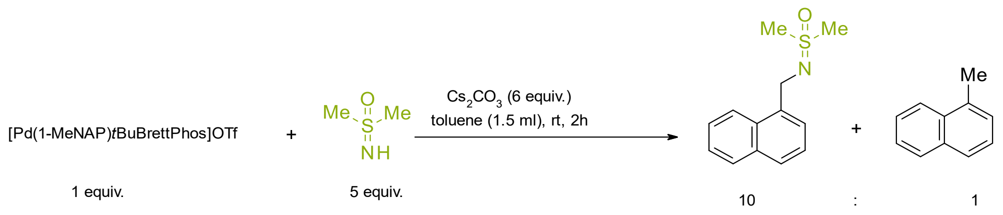

[Pd(cinnamyl)(*t*BuBrettPhos)OTf]:

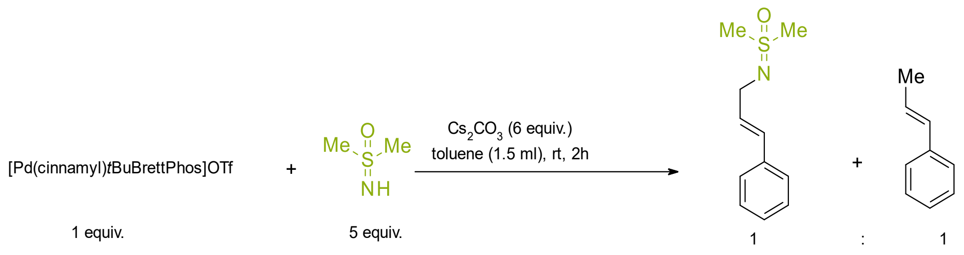

Scheme S2: GC/ GC-MS study

One pot and parallel reaction:

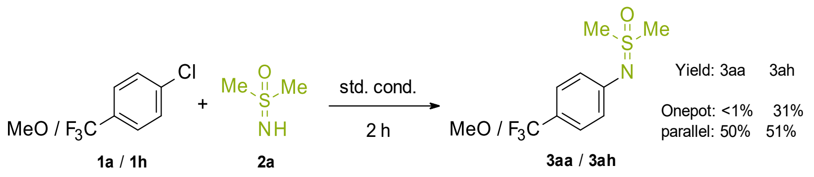

Scheme S3: competition study

In a one-pot competitive coupling of **2a** with an electron-deficient aryl chloride (**1h**) and an electron-rich counterpart (**1a**), preferential coupling of the electron-deficient aryl chloride **1h** was observed. However, in parallel reactions, 1h and 1a were consumed at comparable rates, suggesting that while oxidative addition is promoted by electron-withdrawing substituents, it is not the rate-determining step overall. Furthermore, since the rate of conversion of 1a was not higher in the parallel experiment, reductive elimination can also be excluded as the rate-limiting step.

5. Synthesis and characterization of preformed [Pd(1-MeNAP)L]OTf complexes.

A dry Schlenk flask equipped with a Teflon-coated magnetic stir bar was charged with $[Pd(1\text{-}MeNAP)Br]_2$ (0.50 mmol, 0.50 equiv) followed by AgOTf (1.00 mmol, 1.00 equiv). The flask was fitted with a rubber septum, evacuated, and backfilled with nitrogen. This evacuation/nitrogen backfill cycle was repeated two additional times. Solvent (10 mL of THF) was added, and the reaction mixture was stirred at rt for 30 min while protected from light. A second dry Schlenk flask was equipped with a magnetic stir bar, fitted with a Schlenk frit, and charged with the appropriate ligand (1.00 mmol, 1.00 equiv). The flask was fitted with a rubber septum, and it was evacuated and backfilled with nitrogen. This evacuation/nitrogen backfill cycle was repeated two additional times. The solution from the first Schlenk flask was transferred via cannula through the Schlenk frit (to remove AgCl) and into the second Schlenk flask containing the ligand, rinsing with 5 mL of additional solvent (THF). This mixture was stirred at rt for 2 h. 30 mL of hexanes was then added to fully precipitate the product. The solid materials were then collected by suction filtration, washed with additional pentane (or hexanes), and dried in vacuo.

[Pd(1-MeNAP)(*t*BuBrettPhos)OTf] (**Ia**$^{\mathbf{OTf}}$):

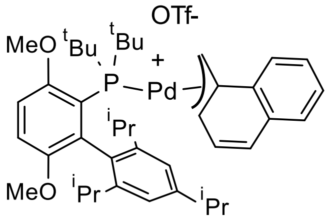

Following general procedure and using *t*BuBrettPhos as ligand, the title compound was obtained as a yellow solid. (840 mg, 0.953 mmol, 95%).

Crystals suitable for x-ray analysis were grown from a saturated THF solution layered with pentane at -20 °C.

^{1}**H NMR** (400 MHz, $CDCl_3$): δ = 8.14 (dd, *J* = 4.3, 8.4 Hz, 1 H), 7.94 - 7.88 (m, 1 H), 7.81 (d, *J* = 7.6 Hz, 1 H), 7.71 - 7.60 (m, 2 H), 7.20 - 7.13 (m, 1 H), 7.05 (dd, *J* = 3.0, 8.9 Hz, 1 H), 6.91 (d, *J* = 8.9 Hz, 1 H), 6.63 - 6.52 (m, 1 H), 6.26 (br. s., 1 H), 5.67 (t, *J* = 6.4 Hz, 1 H), 4.90 (br. s., 1 H), 3.88 - 3.84 (m, 3 H), 3.50 - 3.42 (m, 1 H), 3.42 - 3.37 (m, 1 H), 3.25 (s, 3 H), 2.85 (br. s., 1 H), 2.63 (br. s., 1 H), 1.67 - 1.53 (m, 10 H), 1.51 - 1.32 (m, 17 H), 0.86 (d, *J* = 4.0 Hz, 3 H), 0.38 (br. s.,3 H), -0.14 (d, *J* = 3.6 Hz, 3 H) ppm.

^{13}**C NMR** (101 MHz, $CDCl_3$): δ = 154.7, 154.4(d, *J* = 2.2 Hz), 151.6, 151.4, 136.8, 136.6, 135.3 (d, *J* = 6.5 Hz), 134.6 (d, *J* = 3.6 Hz), 129.7, 129.4, 129.3, 129.1, 127.4 (d, *J* = 6.5 Hz), 125.7, 125.5, 123.7, 122.7, 115.3, 114.9, 112.6 (d, *J* = 5.1 Hz), 112.1(d, *J* = 5.0 Hz), 111.2, 111.1, 70.7, 54.7 (d, *J* = 5.0 Hz), 42.6 (d, *J* = 5.1 Hz), 33.6, 32.3, 30.83, 26.6, 26.0, 24.9, 24.2(d, *J* = 18.9 Hz), 23.3 (d, *J* = 18.9 Hz) ppm [observed complexity due to C–P and C–F coupling].

IR (ATR): $\tilde{\nu}$ = 2979, 2951, 2840, 1580, 1463, 1426, 1267, 1139, 1088, 1051 cm^{-1}

^{19}**F NMR** (41 MHz, CDCl3, $C_6H_4F_2$): δ= −77.71 (s) ppm.

^{31}**P{^{1}H} NMR** (162 MHz, CDCl3): δ= 94.04 ppm.

EA Anal. Calcd for $C_{43}H_{58}F_3O_5PPdS$: C, 58.60; H, 6.63; N, 0.00; S, 3.64 Found: C, 58.73; H, 6.21; N,0.24; S, 3.31

^{1}H NMR (400 MHz, $CDCl_3$)

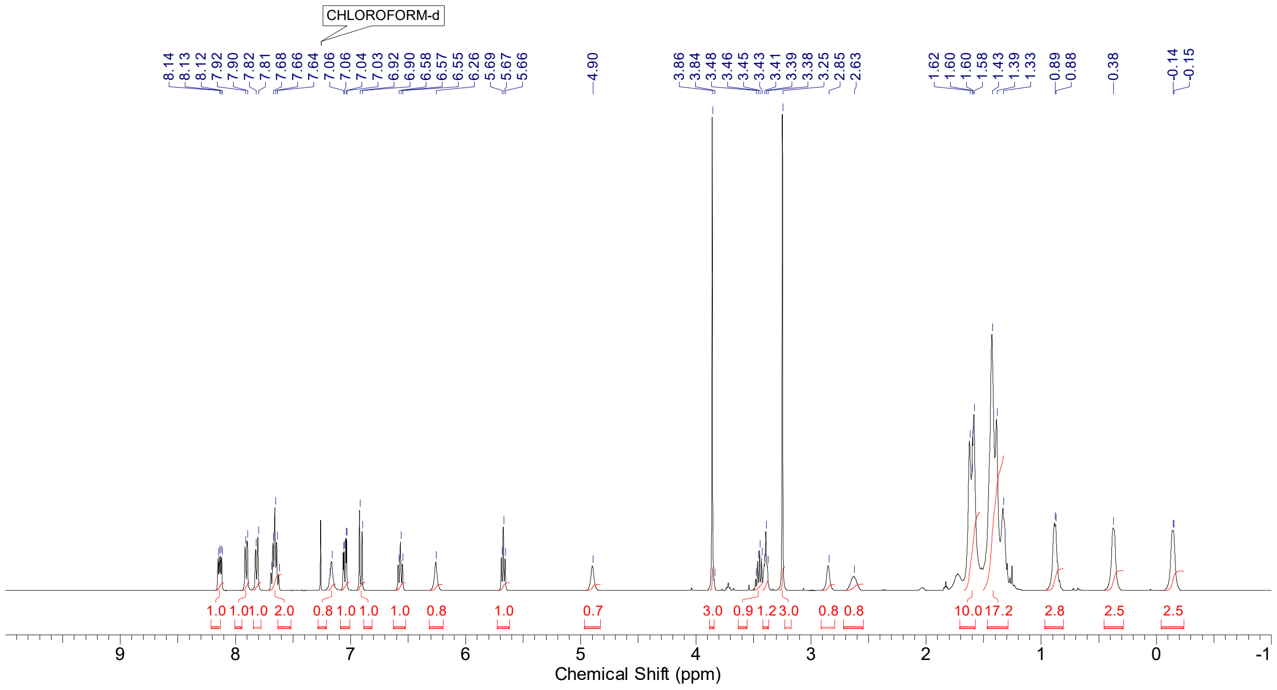

^{13}C NMR (101 MHz, $CDCl_3$)

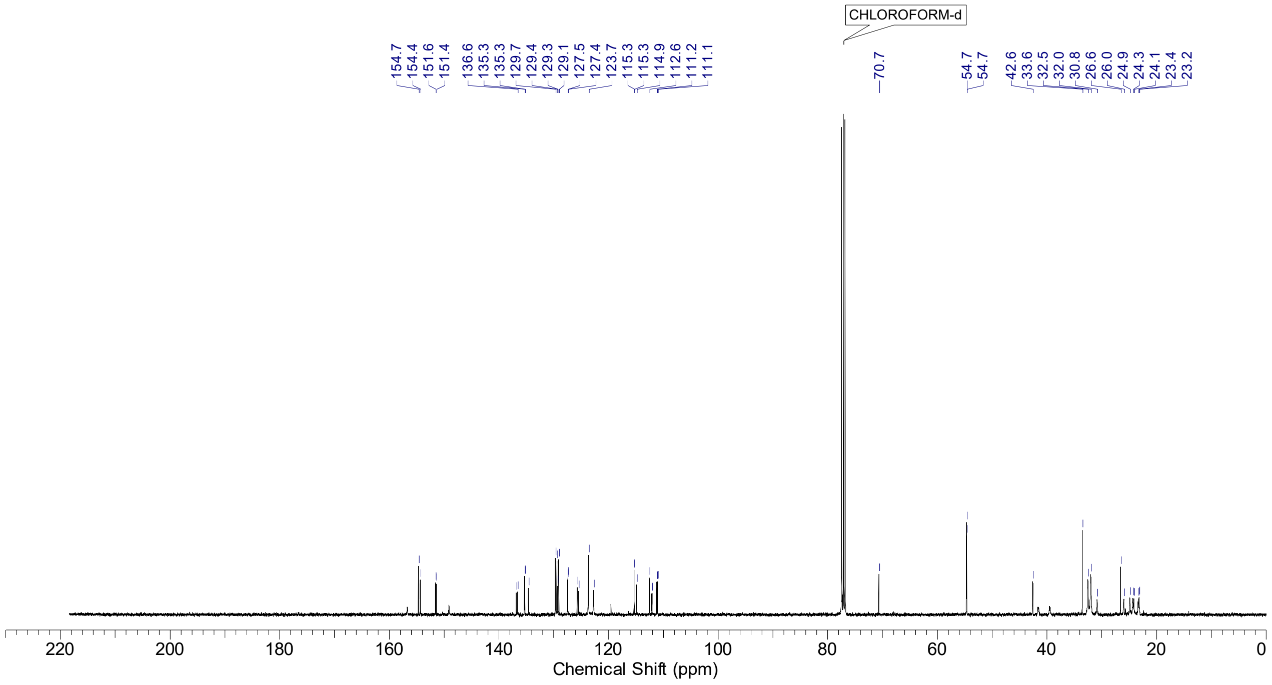

^{19}F NMR (41 MHz, CDCl3, $C_6H_4F_2$)

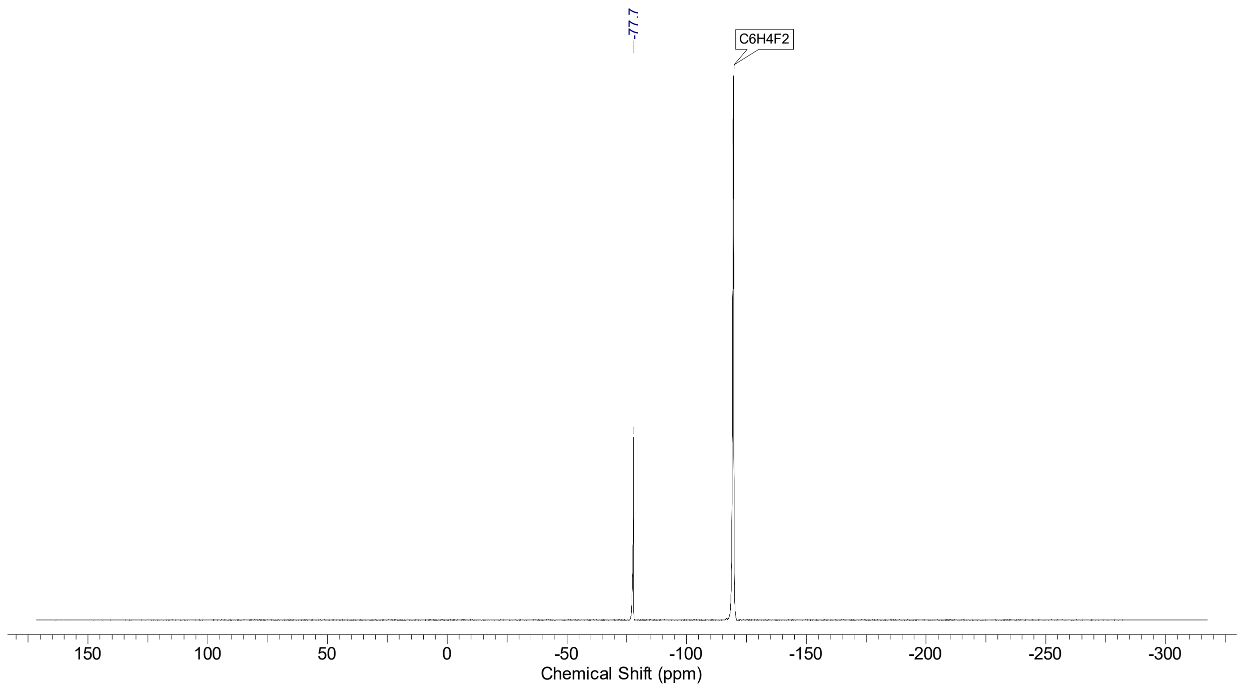

$^{31}P\{^1H\}$ NMR (162 MHz, CDCl3)

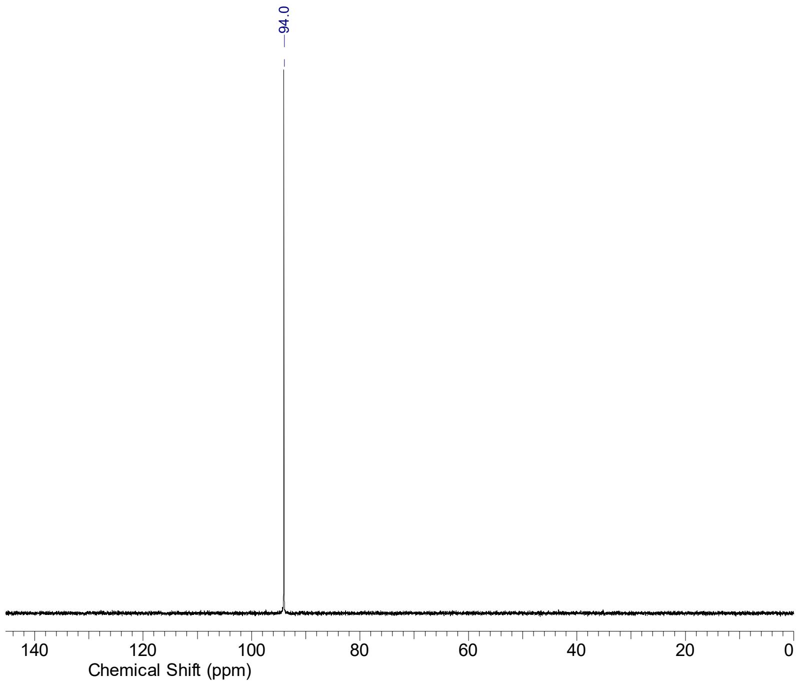

[Pd(1-MeNAP)(RuPhos)]OTf:

^{1}H NMR (400 MHz, $CDCl_3$): δ = 8.51 (dd, J = 4.0, 8.0 Hz, 1 H), 8.00 - 7.90 (m, 1 H), 7.80 - 7.69 (m, 2 H), 7.65 - 7.55 (m, 3 H), 7.49 - 7.36 (m, 2 H), 7.02 - 6.91 (m, 1 H), 6.75 - 6.53 (m, 2 H), 5.93 (t, J = 6.1 Hz, 1 H), 5.55 (br. s., 1 H), 4.66 (br. s., 1 H), 4.33 (br. s., 1 H), 3.66 (br. s., 1 H), 2.94 (br. s., 1 H), 2.61 - 2.06 (m, 3 H), 1.97 (br. s., 1 H), 1.91 - 1.71 (m, 11 H), 1.55 - 1.27 (m, 10 H), 1.21 - 1.13 (m, 1 H), 1.08 - 0.94 (m, 3 H), 0.88 (t, J = 7.1 Hz, 1 H), 0.65 (br. s., 2 H), -0.03 (br. s., 2 H) ppm.

^{13}C NMR (101 MHz, $CDCl_3$): δ = 145.5, 145.3, 135.1, 134.7, 132.9(d, J = 6.0 Hz), 132.0(d, J = 3.1 Hz), 131.6, 131.3, 131.2, 130.0, 129.6, 129.1, 128.2, 127.7(d, J = 6.1 Hz), 127.5(d, J = 7.0 Hz), 122.8, 122.4, 119.2, 116.0, 114.0, 114.0, 111.9, 111.7, 102.1, 102.1, 37.5(d, J = 4.0 Hz), 26.8, 26.6, 26.0, 21.6 ppm [observed complexity due to C–P and C–F coupling].

^{19}F NMR (376.51 MHz, $CDCl_3$, $C_6H_4F_2$): δ= −77.36 (s) ppm.

^{31}P{^{1}H} NMR (162 MHz, $CDCl_3$): δ= 60.48 ppm.

^{1}H NMR (400 MHz, $CDCl_3$)

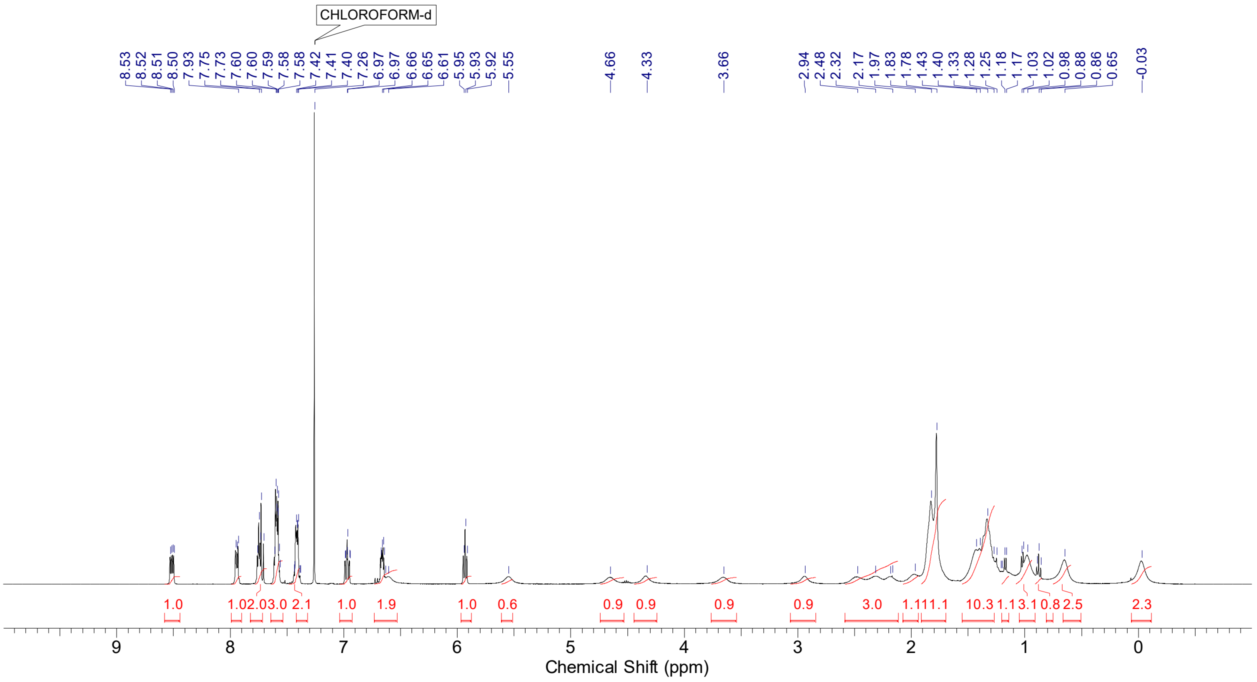

^{13}C NMR (101 MHz, $CDCl_3$)

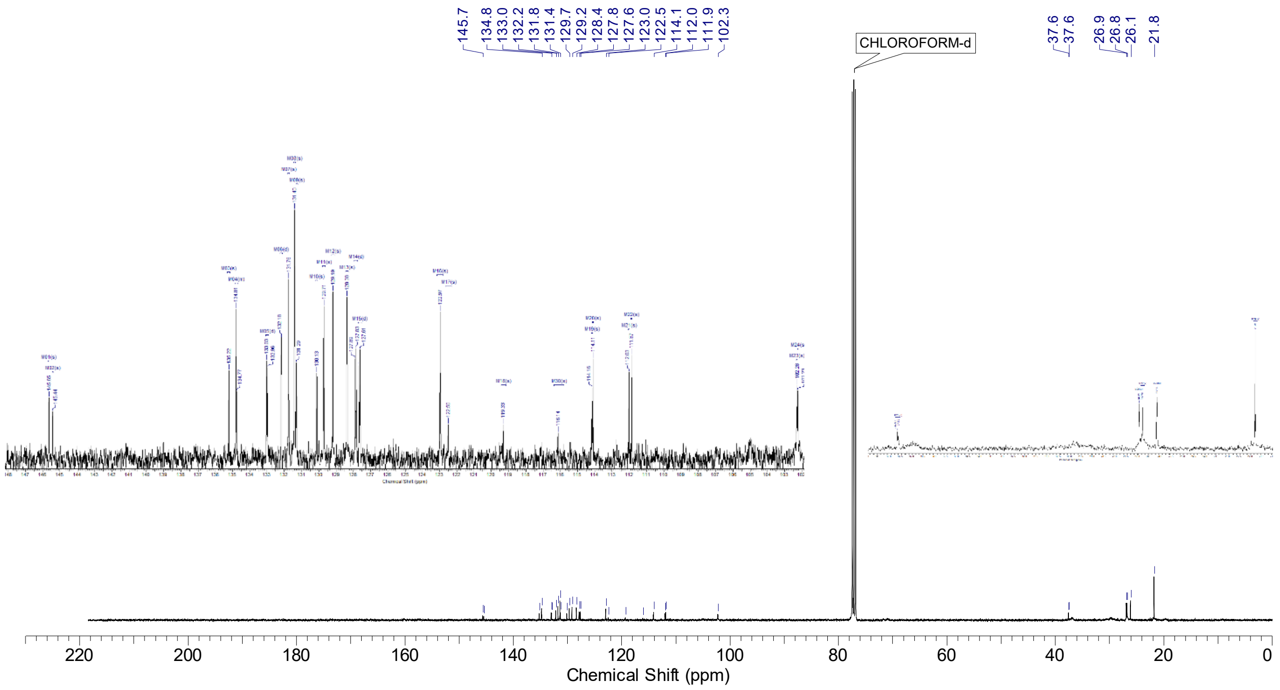

^{19}F NMR (376.51 MHz, $CDCl_3$, $C_6H_4F_2$)

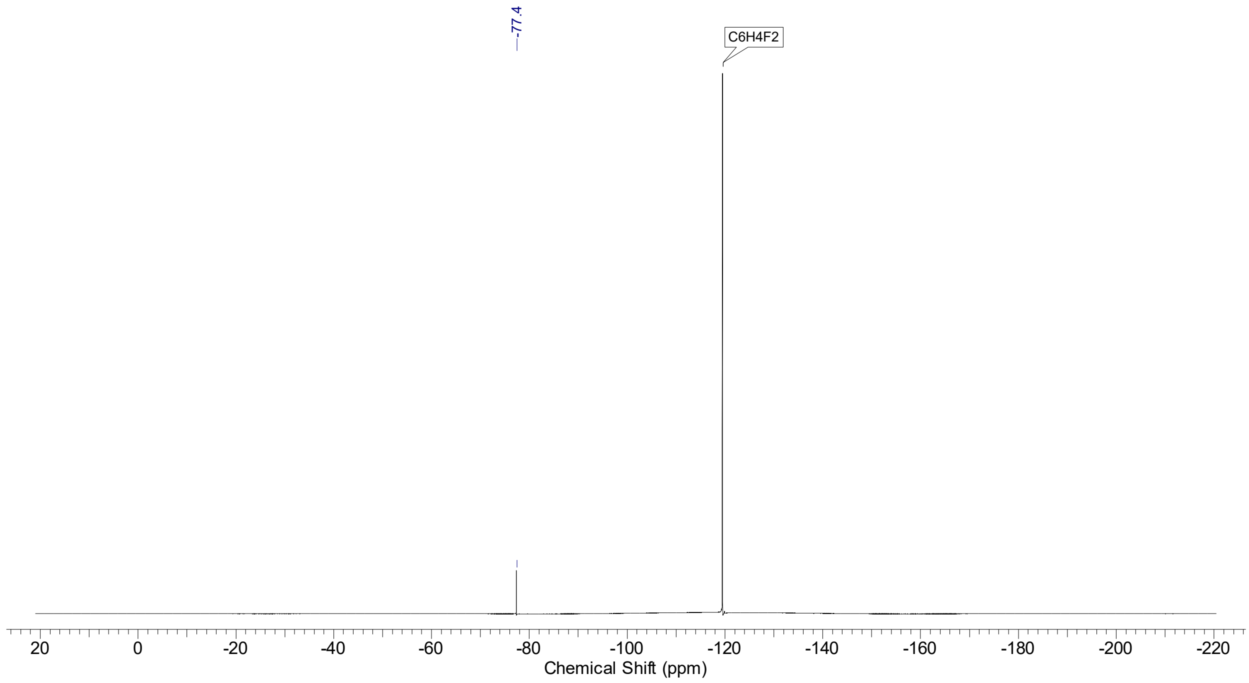

$^{31}P\{^1H\}$ NMR (162 MHz, $CDCl_3$)

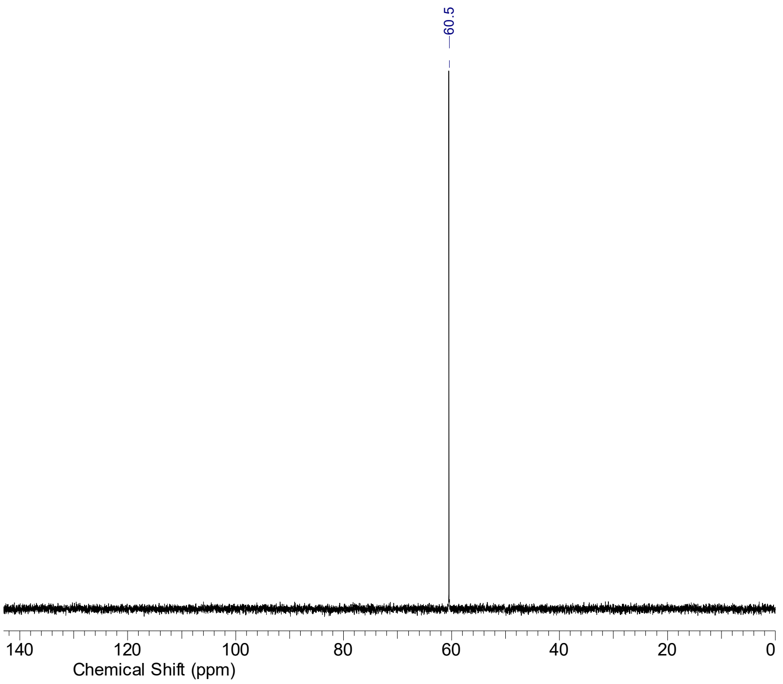

[Pd(1-MeNAP)(BrettPhos)]OTf:

^{1}H NMR (400 MHz, $CDCl_3$): δ = 8.04 (dd, J = 4.3, 8.5 Hz, 1 H), 7.90 - 7.82 (m, 2 H), 7.67 - 7.62 (m, 2 H), 7.05 - 6.99 (m, 1 H), 6.91 (d, J = 8.9 Hz, 1 H), 6.67 - 6.60 (m, 1 H), 6.43 (br. s., 1 H), 5.68 (t, J = 6.4 Hz, 1 H), 4.66 (br. s., 1 H), 3.91 (s, 3 H), 3.74 - 3.70 (m, 2 H), 3.44 - 3.35 (m, 3 H), 3.29 (s, 3 H), 3.00 (d, J = 8.0 Hz, 1 H), 2.88 (br. s., 1 H), 2.79 (br. s., 1 H), 2.66 (q, J = 11.0 Hz, 1 H), 2.48 (br. s., 1 H), 2.21 - 2.07 (m, 2 H), 1.94 (br. s., 2 H), 1.85 - 1.81 (m, 3 H), 1.66 (br. s., 3 H), 1.61 - 1.56 (m, 4 H), 1.47 (d, J = 7.0 Hz, 2 H), 1.40 (d, J = 6.3 Hz, 3 H), 1.35 (d, J = 5.9 Hz, 4 H), 1.28 (d, J = 6.8 Hz, 2 H), 0.88 - 0.83 (m, 4 H), 0.46 (d, J = 5.4 Hz, 3 H), -0.26 (d, J = 5.5 Hz, 3 H) ppm.

^{13}C NMR (101 MHz, $CDCl_3$): δ = 154.6(d, J = 2.9 Hz), 153.9, 151.7(d, J = 16.0 Hz), 149.3, 135.8, 135.6, 134.7(d, J = 2.9 Hz), 133.9(d, J = 6.5 Hz), 129.7, 129.4, 129.0, 128.6, 127.6(d, J = 6.5 Hz), 124.0, 123.7, 123.6, 123.5, 122.5, 122.2, 119.4, 115.7(d, J = 5.1 Hz), 115.3, 114.1(d, J = 5.1 Hz), 112.7(d, J = 4.4 Hz), 110.4(d, J = 15.3 Hz), 70.7, 68.1, 56.1, 54.8, 39.0, 37.9(d, J = 4.4 Hz), 34.2, 33.5, 32.3, 31.9, 30.4, 27.3, 26.6, 26.0, 25.7, 24.3, 23.9, 23.3(d, J = 16.0 Hz), 22.4, 14.1 ppm [observed complexity due to C–P and C–F coupling].

^{19}F NMR (376.51 MHz, $CDCl_3$, $C_6H_4F_2$): δ= −77.68 (s) ppm.

$^{31}P\{^1H\}$ NMR (162 MHz, $CDCl_3$): δ= 59.58 ppm.

^{1}H NMR (400 MHz, $CDCl_3$)

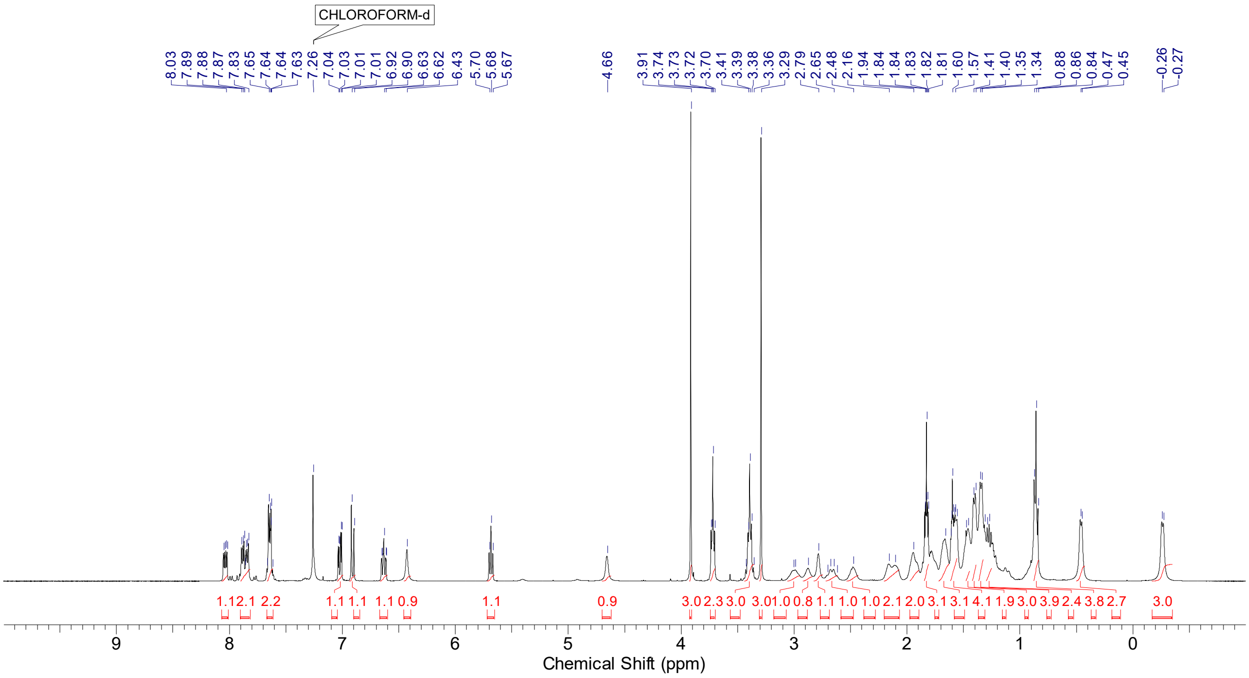

^{13}C NMR (101 MHz, $CDCl_3$)

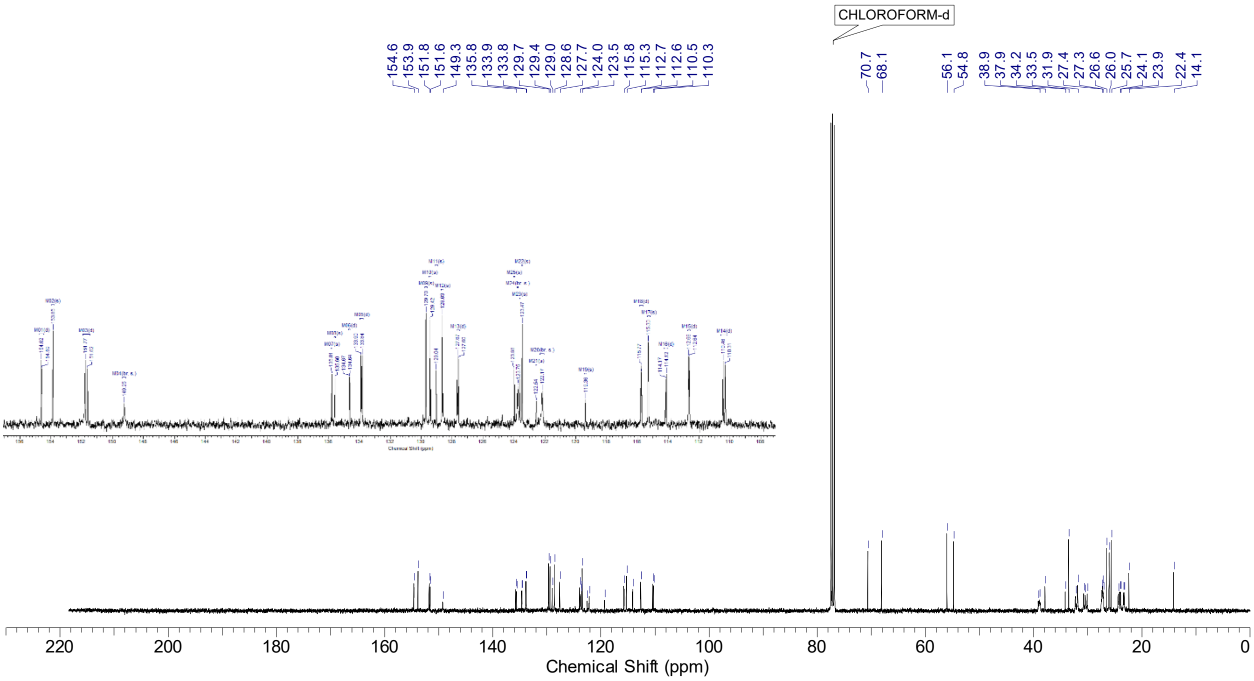

^{19}F NMR (376.51 MHz, $CDCl_3$, $C_6H_4F_2$)

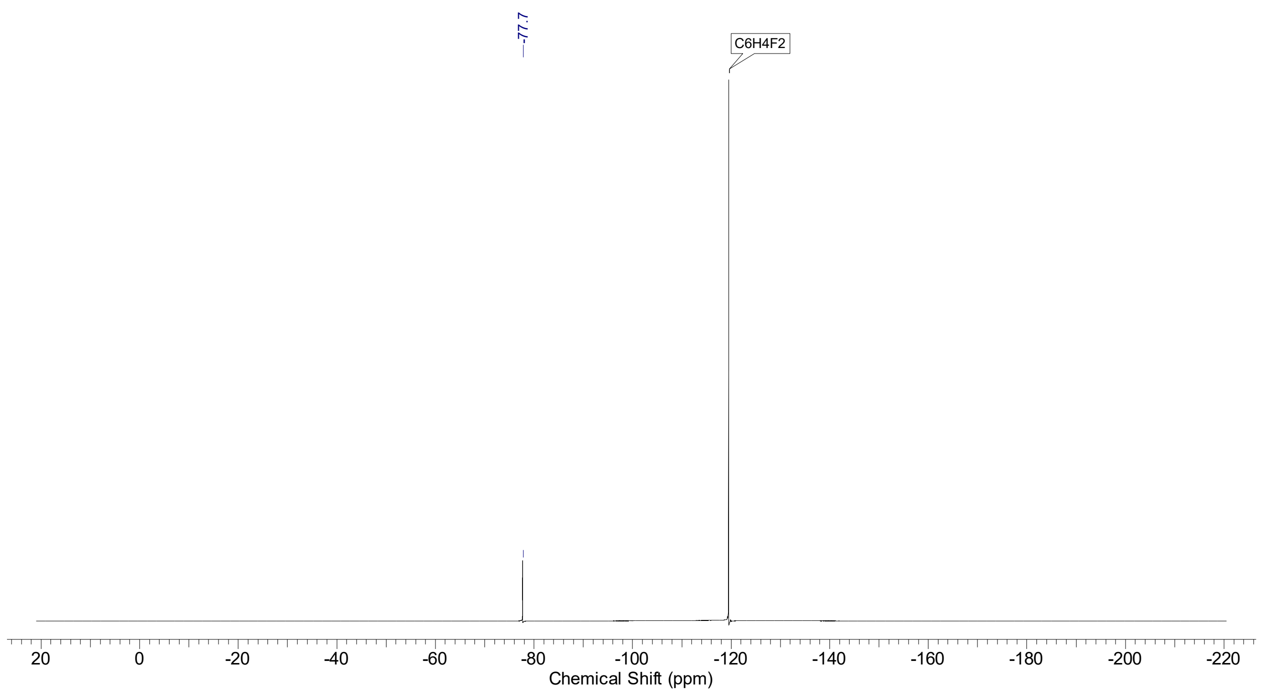

$^{31}P\{^1H\}$ NMR (162 MHz, $CDCl_3$)

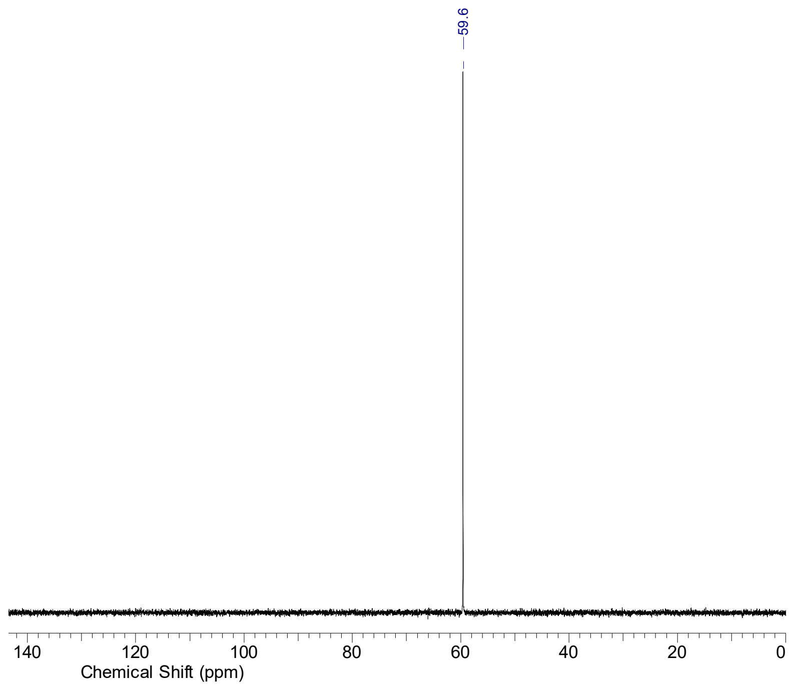

6. Synthesis and Characterization of Products

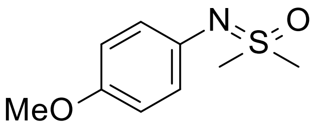

(4-methoxyphenyl)imino-dimethyl-oxo-λ⁶-sulfane [CAS: 58873-28-2] **(3aa)**

Following general procedure, **A** and starting from 1-chloro-4-methoxy-benzene (72.7 mg, 0.50 mmol), the title compound was obtained as a brown liquid (97.6 mg, 0.490 mmol, 98%). Purification by Column chromatography (silica gel, (0–100% ethyl acetate in cyclohexane).

m.p.: 124-125 °C.

^{1}H NMR (400 MHz, $CDCl_3$): δ = 7.05 - 6.94 (m, 2 H), 6.85 - 6.72 (m, 2 H), 3.77 (s, 3 H), 3.11 (s, 6 H) ppm.

^{13}C NMR (101 MHz, $CDCl_3$): δ = 155.5, 137.9, 124.9, 114.7, 55.6, 41.9 ppm.

IR (ATR): $\tilde{\nu}$ = 2995, 2955, 2917, 1501, 1295, 1172, 1054, 828, 674, 457 cm^{-1}.

HRMS (EI-TOF) $[M]^+$ calcd. for $C_9H_{13}NO_2S$: 199.0667; found: 199.0663.

The NMR data are similar to those reported in literature.[1]

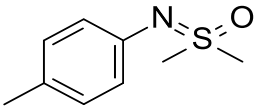

dimethyl-oxo-(p-tolylimino)-λ⁶-sulfane [CAS: 56157-99-4] **(3ba)**

Following general procedure **A** and starting from 1-Chloro-4-methylbenzene (63.3 mg, 0.50 mmol), the title compound was obtained as a white solid (90 mg, 0.491 mmol, 98%). Purification by Column chromatography (silica gel, (0–100% ethyl acetate in cyclohexane).

m.p.: 76-77 °C.

^{1}H NMR (400 MHz, $CDCl_3$): δ = 7.07 - 7.01 (m, 2 H), 7.00 - 6.94 (m, 2 H), 3.11 (s, 6 H), 2.28 (s, 3 H) ppm.

^{13}C NMR (101 MHz, $CDCl_3$): δ = 142.3, 131.8, 129.9, 123.5, 42.0, 20.8 ppm.

IR (ATR): $\tilde{\nu}$ = 3001, 2922, 2860, 1504, 1280, 1260, 1182, 1054, 818, 470 cm^{-1}.

HRMS (EI-TOF) $[M]^+$ calcd. for $C_9H_{13}NOS$: 183.0717; found: 183.0716.

The NMR data are similar to those reported in literature.[1]

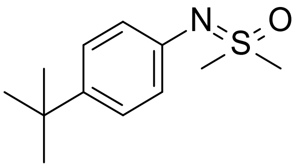

(4-tert-butylphenyl)imino-dimethyl-oxo-λ⁶-sulfane [CAS: 1374007-21-2] **(3ca)**

Following general procedure **A** and starting from 1-tert-Butyl-4-chlorobenzene (84.3 mg, 0.50 mmol), the title compound was obtained as a yellow liquid (106 mg, 0.470 mmol, 94%). Purification by Column chromatography (silica gel, (0–100% ethyl acetate in cyclohexane).

^{1}H NMR (400 MHz, $CDCl_3$): δ = 7.29 - 7.19 (m, 2 H), 6.99 (d, J=8.5 Hz, 2 H), 3.13 (s, 6 H), 1.29 (s, 9 H) ppm.

^{13}C NMR (101 MHz, $CDCl_3$): δ = 145.1, 142.3, 126.2, 123.0, 42.1, 34.2, 31.6 ppm.

IR (ATR): $\tilde{\nu}$ = 2934, 1503, 1444, 1460, 1281, 1233, 1172, 1037, 825, 733 cm^{-1}.

HRMS (EI-TOF) $[M]^+$ calcd. for $C_{12}H_{19}NOS$: 225.1187; found: 225.1185.

The NMR data are similar to those reported in literature.[4]

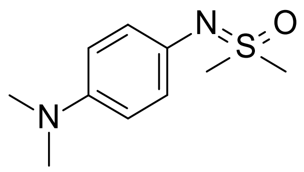

4-[[dimethyl(oxo)-λ[6]-sulfanylidene]amino]-N,N-dimethyl-aniline **(3da)**

Following general procedure **A** and starting from 4-chloro-N,N-dimethyl-aniline (80.2 mg, 0.50 mmol), the title compound was obtained as a black liquid (98 mg, 0.462 mmol, 92%). Purification by Column chromatography (silica gel, (0–100% ethyl acetate in cyclohexane).

^{1}H NMR (400 MHz, $CDCl_3$): *δ* = 6.98 (d, *J* = 8.9 Hz, 2 H), 6.68 (d, *J* = 8.9 Hz, 2 H), 3.10 (s, 6 H), 2.89 (s, 6 H) ppm.

^{13}C NMR (101 MHz, $CDCl_3$): *δ* = 147.1, 134.9, 124.8, 114.3, 41.8, 41.4 ppm.

IR (ATR): $\tilde{\nu}$ = 3010, 2911, 2806, 1679, 1509, 1256, 1172, 1059, 937, 812 cm^{-1}.

HRMS (EI-TOF) $[M]^+$ calcd. for $C_{10}H_{16}N_2OS$: 212.0983; found: 212.0982.

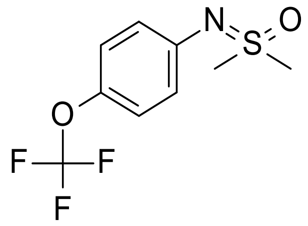

dimethyl-oxo-[4-(trifluoromethoxy)phenyl]imino-λ[6]-sulfane **(3ea)**

Following general procedure **A** and starting from 1-chloro-4-(trifluoromethoxy) benzene (98.3 mg, 0.50 mmol), the title compound was obtained as a Brown sticky liquid (124 mg, 0.490 mmol, 98%). Purification by Column chromatography (silica gel, (0–10% methanol in ethyl acetate).

^{1}H NMR (300 MHz, $CDCl_3$): *δ* = 7.11 - 7.01 (m, 4 H), 3.13 (s, 6 H) ppm.

^{13}C NMR (75 MHz, $CDCl_3$): *δ* = 144.3 (q, *J* = 1.5 Hz), 144.1, 124.2, 122.1, 120.6 (q, *J* = 245.25 Hz), 42.2 ppm.

^{19}F NMR (76 MHz, CDCl3, $C_6H_4F_2$): *δ*= −57.97 ppm.

IR (ATR): $\tilde{\nu}$ = 3017, 2931, 1603, 1499, 1245, 1189, 1150, 1047, 933, 846 cm^{-1}.

HRMS (EI-TOF) $[M]^+$ calcd. for $C_9H_{10}F_3NO_2S$: 253.0384; found: 253.0379.

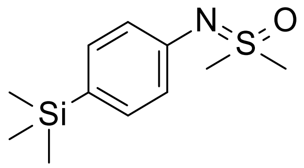

[4-[dimethyl(oxo)-λ[6]-sulfanylidene]amino]phenyl]-trimethyl-silane] **(3fa)**

Following general procedure **A** and starting from (4-chlorophenyl)-trimethyl-silane (95.2 mg, 0.50 mmol), the title compound was obtained as a yellow solid (114 mg, 0.472 mmol, 94%). Purification by Column chromatography (silica gel, (0–100% ethyl acetate in cyclohexane).

m.p.: 62-64 °C.

^{1}H NMR (400 MHz, $CDCl_3$): *δ* = 7.39 (d, *J* = 8.3 Hz, 2 H), 7.06 (d, *J* = 8.3 Hz, 2 H), 3.16 (s, 6 H), 0.24 (s, 9 H) ppm.

^{13}C NMR (101 MHz, $CDCl_3$): *δ* = 145.9, 134.6, 133.1, 122.8, 42.3, -0.8 ppm.

IR (ATR): $\tilde{\nu}$ = 3005, 2952, 2927, 1587, 1496, 1282, 1180, 1048, 832, 753 cm^{-1}.

HRMS (EI-TOF) $[M]^+$ calcd. for $C_{11}H_{19}NOSSi$: 241.0956; found: 241.0955.

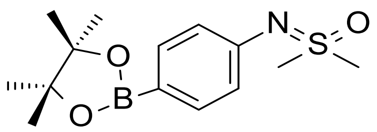

Dimethyl-oxo-[4-(4,4,5,5-tetramethyl-1,3,2-dioxaborolan-2-yl)phenyl]imino-λ⁶-sulfane[CAS: 1644499-93-3] **(3ga)**

Following general procedure **A** and starting from 2-(4-chlorophenyl)-4,4,5,5-tetramethyl-1,3,2-dioxaborolane (122 mg, 0.50 mmol), the title compound was obtained as a yellow solid (140 mg, 0.474 mmol, 95%). Purification by Column chromatography (silica gel, (0–100% ethyl acetate in cyclohexane).

m.p.: 119.5-121.7 °C.

^{1}H NMR (300 MHz, $CDCl_3$): δ = 7.73 - 7.65 (m, 2 H), 7.11 - 7.01 (m, 2 H), 3.16 (s, 6 H), 1.32 (s, 12 H) ppm.

^{13}C NMR (75 MHz, $CDCl_3$): δ = 148.5, 136.2, 122.5, 83.7, 42.3, 25.0 ppm. (B-C resonance not observed due to quadrupolar B).

IR (ATR): $\tilde{\nu}$ = 2977, 2930, 1738, 1600, 1355, 1278, 1139, 1041, 857, 654 cm^{-1}.

HRMS (EI-TOF) $[M]^+$ calcd. for $C_{14}H_{22}BNO_3S$: 295.1449; found: 295.1449.

The NMR data are similar to those reported in literature.[1]

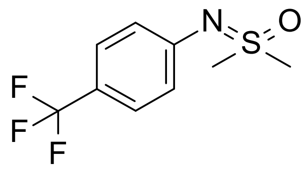

dimethyl-oxo-[4-(trifluoromethyl)phenyl]imino-λ⁶-sulfane [CAS: 2060038-95-9] **(3ha)**

Following general procedure **A** and starting from 1-chloro-4-(trifluoromethyl)benzene (90.3 mg, 0.50 mmol), the title compound was obtained as a white solid (96.1 mg, 0.481 mmol, 96%). Purification by Column chromatography (silica gel, (0–100% ethyl acetate in cyclohexane).

m.p.: 82-84 °C.

^{1}H NMR (400 MHz, $CDCl_3$): δ = 7.47 (d, J = 8.3 Hz, 2 H), 7.14 (d, J = 8.3 Hz, 2 H), 3.18 (s, 6 H) ppm.

^{13}C NMR (101 MHz, $CDCl_3$): δ = 148.9 (q, J = 2.02 Hz), 126.5 (q, J = 3.03 Hz), 124.7 (q, J = 272.7 Hz), 123.9 (q, J = 32.3 Hz), 122.9, 42.4 ppm.

^{19}F NMR (76 MHz, CDCl3, $C_6H_4F_2$): δ= −61.53 ppm.

IR (ATR): $\tilde{\nu}$ = 3015, 2971, 2902, 1610, 1513, 1156, 1107, 1037, 843, 499 cm^{-1}.

HRMS (EI-TOF) $[M]^+$ calcd. for $C_9H_{10}F_3NOS$: 237.0435; found: 237.0431.

The NMR data are similar to those reported in literature.[2]

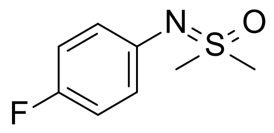

(4-fluorophenyl)imino-dimethyl-oxo-λ⁶-sulfane [CAS: 58873-25-9] **(3ia)**

Following general procedure **A** and starting from 1-Chloro-4-fluorobenzene (65.3 mg, 0.50 mmol), the title compound was obtained as a white solid (89 mg, 0.475 mmol, 95%). Purification by Column chromatography (silica gel, (0–10% methanol in ethyl acetate).

m.p.: 93.0-94.5 °C.

^{1}H NMR (300 MHz, $CDCl_3$): δ = 7.06 - 6.98 (m, 2 H), 6.97 - 6.88 (m, 2 H), 3.12 (s, 6 H) ppm.

^{13}C NMR (75 MHz, $CDCl_3$): δ = 158.9 (d, J = 239.3 Hz), 140.9 (d, J = 2.8 Hz), 124.8 (d, J = 7.7 Hz), 115.8 (d, J = 21.8 Hz), 42.1 ppm.

^{19}F NMR (76 MHz, CDCl3, $C_6H_4F_2$): δ= −121.53 (m) ppm.

IR (ATR): $\tilde{\nu}$ = 3005, 2924, 2215, 1590, 1329, 1207, 1055, 938, 833, 763 cm^{-1}.

HRMS (EI-TOF) $[M]^+$ calcd. for $C_8H_{10}FNOS$: 187.0467; found: 187.0462.

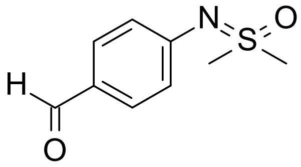

4-[[dimethyl(oxo)-λ^6-sulfanylidene]amino]benzaldehyde **(3ja)**

Following general procedure **A** and starting from 4-chlorobenzaldehyde (72.5 mg, 0.50 mmol), the title compound was obtained as a yellow solid (92 mg, 0.466 mmol, 93%). Purification by Column chromatography (silica gel, (0–100% ethyl acetate in cyclohexane).

m.p.: 68-70°C.

^{1}H NMR (400 MHz, $CDCl_3$): δ = 9.86 (s, 1 H), 7.75 (d, J = 8.5 Hz, 2 H), 7.16 (d, J = 8.5 Hz, 2 H), 3.22 (s, 6 H) ppm.

^{13}C NMR (101 MHz, $CDCl_3$): δ = 191.1, 152.4, 131.6, 130.5, 122.7, 42.7 ppm.

IR (ATR): $\tilde{\nu}$ = 3009, 2922, 1671, 1591, 1501, 1266, 1192, 1161, 1040, 838 cm^{-1}.

HRMS (EI-TOF) $[M]^+$ calcd. for $C_9H_{11}NO_2S$: 197.0510; found: 197.0509.

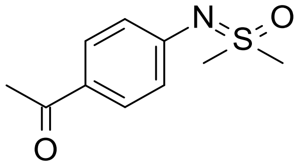

1-[4-[[dimethyl(oxo)-λ^6-sulfanylidene]amino]phenyl]ethenone **(3ka)**

Following general procedure **A** and starting from 1-(4-chlorophenyl)ethenone (78.9 mg, 0.50 mmol), the title compound was obtained as a white solid (93.6 mg, 0.468 mmol, 94%). Purification by Column chromatography (silica gel, (0–10% methanol in ethyl acetate).

m.p.: 120-123°C.

^{1}H NMR (300 MHz, $CDCl_3$): δ = 7.90 - 7.79 (m, 2 H), 7.15 - 7.04 (m, 2 H), 3.20 (s, 6 H), 2.54 (s, 3 H) ppm.

^{13}C NMR (75 MHz, $CDCl_3$): δ = 197.2, 150.9, 131.1, 130.2, 122.4, 42.6, 26.5 ppm.

IR (ATR): $\tilde{\nu}$ = 3013, 2990, 2927, 2215, 1657, 1588, 1293, 1185, 1045, 830 cm^{-1}.

HRMS (EI-TOF) $[M]^+$ calcd. for $C_{10}H_{13}NO_2S$: 211.0666; found: 211.0662.

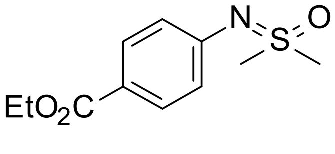

Ethyl 4-[[dimethyl(oxo)-λ^6-sulfanylidene]amino]benzoate [CAS: 2943218-46-8] **(3la)**

Following general procedure **A** and starting from ethyl 4-chlorobenzoate (94.2mg, 0.50 mmol), the title compound was obtained as a brown solid (109 mg, 0.452 mmol, 90%). Purification by Column chromatography (silica gel, (0–100% ethyl acetate in cyclohexane).

m.p.: 70-72 °C.

^{1}**H NMR** (400 MHz, $CDCl_3$): δ = 7.88 (d, J = 8.6 Hz, 2 H), 7.05 (d, J = 8.6 Hz, 2 H), 4.30 (q, J = 7.1 Hz, 2 H), 3.15 (s, 6 H), 1.33 (t, J = 7.1 Hz, 3 H) ppm.

^{13}**C NMR** (101 MHz, $CDCl_3$): δ = 166.6, 150.4, 131.0, 123.7, 122.3, 60.6, 42.3, 14.4 ppm.

IR (ATR): $\tilde{\nu}$ = 3011, 2919, 1689, 1595, 1504, 1260, 1211, 1142, 855, 770, 498 cm^{-1}.

HRMS (EI-TOF) $[M]^+$ calcd. for $C_{11}H_{15}NO_3S$: 241.0772; found: 241.0772.

The NMR data are similar to those reported in literature.[3]

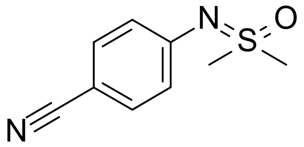

4-[[dimethyl(oxo)-λ^6-sulfanylidene]amino]benzonitrile [CAS: 56158-12-4] **(3ma)**

Following general procedure **A** and starting from 4-chlorobenzonitrile (69.5 mg, 0.50 mmol), the title compound was obtained as a yellow solid (93 mg, 0.479 mmol, 96%). Purification by Column chromatography (silica gel, (0–100% ethyl acetate in cyclohexane).

m.p.: 108-109 °C.

^{1}**H NMR** (400 MHz, $CDCl_3$): δ = 7.54 - 7.46 (m, 2 H), 7.15 - 7.05 (m, 2 H), 3.20 (s, 6 H) ppm.

^{13}**C NMR** (101 MHz, $CDCl_3$): δ = 150.5, 133.6, 123.1, 119.7, 104.6, 42.7 ppm.

IR (ATR): $\tilde{\nu}$ = 3003, 2922, 2216, 1599, 1495, 1325, 1266, 1039, 830, 553 cm^{-1}.

HRMS (EI-TOF) $[M]^+$ calcd. for $C_9H_{10}N_2OS$: 194.0513; found: 194.0512.

The NMR data are similar to those reported in literature.[3]

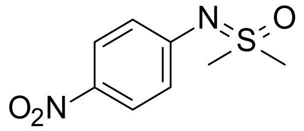

Dimethyl-(4-nitrophenyl)imino-oxo-λ^6-sulfane [CAS: 56158-00-0] **(3na)**

Following general procedure **A** and starting from 1-chloronaphtalene (79.6 mg, 0.50 mmol), the title compound was obtained as a yellow solid (98.2 mg, 0.458 mmol, 92%). Purification by Column chromatography (silica gel, (0–100% ethyl acetate in cyclohexane).

m.p.: 158-160°C.

^{1}**H NMR** (400 MHz, $CDCl_3$): δ = 8.10 (d, J = 9.1 Hz, 2 H), 7.10 (d, J = 9.1 Hz, 2 H), 3.23 (s, 6 H) ppm.

^{13}**C NMR** (101 MHz, $CDCl_3$): δ = 153.0, 142.0, 125.5, 122.2, 42.8 ppm.

IR (ATR): $\tilde{\nu}$ = 3012, 2925, 1674, 1585, 1485, 1270, 1193, 1038, 931, 847 cm^{-1}.

HRMS (EI-TOF) $[M]^+$ calcd. for $C_8H_{10}N_2O_3S$: 214.0412; found: 214.0410.

The NMR data are similar to those reported in literature.[2]

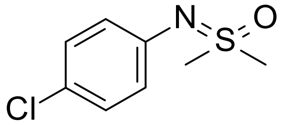

(4-chlorophenyl)imino-dimethyl-oxo-λ^6-sulfane **(3oa)**

Following general procedure **B** and starting from (4-chlorophenyl) trifluoromethanesulfonate (137 mg, 0.50 mmol), the title compound was obtained as a yellow liquid (99.1mg, 0.487 mmol, 97%). Purification by Column chromatography (silica gel, (0–100% ethyl acetate in cyclohexane).

^{1}H NMR (400 MHz, $CDCl_3$): δ = 7.19 - 7.15 (m, 2 H), 7.01 - 6.96 (m, 2 H), 3.12 (s, 6 H) ppm.

^{13}C NMR (101 MHz, $CDCl_3$): δ = 143.9, 129.3, 127.5, 124.7, 42.1 ppm.

IR (ATR): $\tilde{\nu}$ = 3018, 2993, 1586, 1484, 1329, 1182, 1049, 941, 829, 517 cm^{-1}.

HRMS (EI-TOF) $[M]^+$ calcd. for $C_8H_{10}ClNOS$: 203.0171; found: 203.0172.

The NMR data are similar to those reported in literature.[2]

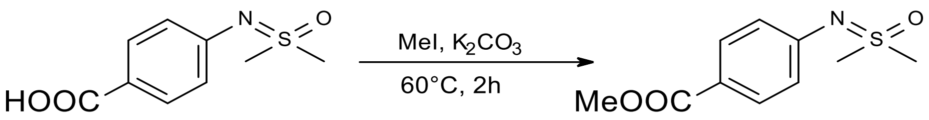

4-[[dimethyl(oxo)-λ^6-sulfanylidene]amino]benzoic acid **(3pa)**

Following general procedure, **A** and starting from 4-chlorobenzoic acid (79.1 mg, 0.50 mmol), the esterified compound was obtained as a yellow liquid after esterification (after reaction with Methyl iodide and K_2CO_3) (78 mg, 0.366 mmol, 73%). Purification by Column chromatography (silica gel+ 1% HCOOH, (0–100% ethyl acetate in 1% HCOOH + cyclohexane).

^{1}H NMR (300 MHz, $CDCl_3$): δ = 7.96 - 7.86 (m, 2 H), 7.13 - 7.04 (m, 2 H), 3.87 (s, 3 H), 3.20 (s, 6 H) ppm.

^{13}C NMR (75 MHz, $CDCl_3$): δ = 167.2, 150.5, 131.2, 123.5, 122.4, 52.0, 42.5 ppm.

IR (ATR): $\tilde{\nu}$ = 3014, 2925, 1698, 1596, 1502, 1437, 1207, 1170, 1043, 854 cm^{-1}.

HRMS (EI-TOF) $[M]^+$ calcd. for $C_{10}H_{13}NO_3S$: 227.0616; found: 227.0614.

The NMR data are similar to those reported in literature.[1]

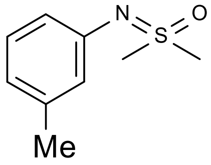

dimethyl-(m-tolylimino)-oxo-λ^6-sulfane **(3qa)**

Following general procedure **A** and starting from 3-Chlorotoluene (64.6 mg, 0.50 mmol), the title compound was obtained as a yellow liquid (84 mg, 0.458 mmol, 92%). Purification by Column chromatography (silica gel, (0–100% ethyl acetate in cyclohexane).

^{1}H NMR (400 MHz, $CDCl_3$): δ = 7.15 - 7.06 (m, 1 H), 6.92 - 6.83 (m, 2 H), 6.80 (d, J = 7.5 Hz, 1 H), 3.12 (s, 6 H), 2.29 (s, 3 H) ppm.

^{13}C NMR (101 MHz, $CDCl_3$): δ = 145.0, 139.1, 129.0, 124.2, 123.1, 120.4, 42.0, 21.5 ppm.

IR (ATR): $\tilde{\nu}$ = 3012, 2933, 1602, 1481, 1309, 1263, 1191, 1061, 890, 779 cm^{-1}.

HRMS (EI-TOF) $[M]^+$ calcd. for $C_9H_{13}NOS$: 183.0717; found: 183.0719.

The NMR data are similar to those reported in literature.[1]

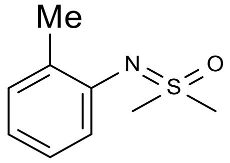

Dimethyl-(o-tolylimino)-oxo-λ⁶-sulfane [CAS: 2060024-93-1] **(3ra)**

Following general procedure, **A** and starting from 2-Chlorotoluene (64.6 mg, 0.50 mmol), the title compound was obtained as a brown liquid (86 mg, 0.469 mmol, 94%). Purification by Column chromatography (silica gel, (0–100% ethyl acetate in cyclohexane).

^{1}**H NMR** (400 MHz, $CDCl_3$): δ = 7.15 (d, J = 7.8 Hz, 2 H), 7.07 (t, J = 7.5 Hz, 1 H), 6.95 - 6.87 (m, 1 H), 3.13 (s, 6 H), 2.24 (s, 3 H) ppm.

^{13}**C NMR** (101 MHz, $CDCl_3$): δ = 143.4, 132.8, 130.6, 126.5, 122.8, 122.5, 42.4, 18.5 ppm.

IR (ATR): $\tilde{\nu}$ = 3013, 2927, 1594, 1485, 1282, 1202, 1116, 1054, 933, 756 cm^{-1}.

HRMS (EI-TOF) $[M]^+$ calcd. for $C_9H_{13}NOS$: 183.0717; found: 183.0717.

The NMR data are similar to those reported in literature.[1]

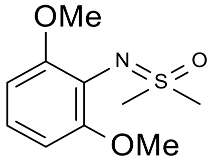

(2,6-dimethoxyphenyl)imino-dimethyl-oxo-λ⁶-sulfane **(3sa)**

Following general procedure **B** (with reaction temperature 100 °C and solvent t-BuOH) and starting from 1-chloro-2,6-dimethoxybenzene (86.3 mg, 0.50 mmol), the title compound was obtained as a brown liquid (98 mg, 0.427 mmol, 85%). Purification by Column chromatography (silica gel, (0–10% methanol in ethyl acetate).

^{1}**H NMR** (400 MHz, $CDCl_3$): δ = 6.95 (t, J = 8.3 Hz, 1 H), 6.57 (d, J = 8.4 Hz, 2 H), 3.83 (s, 6 H), 3.15 (s, 6 H) ppm.

^{*13}**C NMR** (101 MHz, $CDCl_3$): δ = 155.1, 123.3, 121.6, 105.0, 56.2, 43.7 ppm.

IR (ATR): $\tilde{\nu}$ = 3026, 2932, 2831, 1581, 1471, 1435, 1290, 1178, 1099, 758 cm^{-1}.

HRMS (EI-TOF) $[M]^+$ calcd. for $C_{10}H_{15}NO_3S$: 229.0772; found: 229.0774.

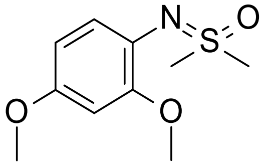

(2,4-dimethoxyphenyl)imino-dimethyl-oxo-λ⁶-sulfane **(3ta)**

Following general procedure **B** and starting from 1-chloro-2,4-dimethoxybenzene (88.1mg, 0.50 mmol), the title compound was obtained as a brown liquid (64.2 mg, 0.280 mmol, 56%). Purification by Column chromatography (silica gel, (0–10% methanol in ethyl acetate).

^{1}**H NMR** (400 MHz, $CDCl_3$): δ = 7.05 (d, J = 8.5 Hz, 1 H), 6.46 (d, J = 4.0 Hz, 1 H), 6.37 (dd, J = 2.7, 8.6 Hz, 1 H), 3.79 (s, 3 H), 3.75 (s, 3 H), 3.10 (s, 6 H) ppm.

^{13}**C NMR** (101 MHz, $CDCl_3$): δ = 156.5, 154.4, 126.1, 125.9, 104.1, 99.8, 55.7, 55.6, 42.2 ppm.

IR (ATR): $\tilde{\nu}$ = 3012, 2959, 1608, 1578, 1501, 1467, 1240, 1176, 1058, 755 cm^{-1}.

HRMS (EI-TOF) $[M]^+$ calcd. for $C_{10}H_{15}NO_3S$: 229.0772; found: 229.0775.

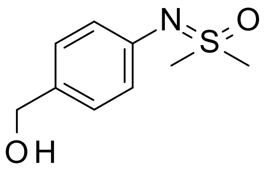

[4-[[dimethyl(oxo)-λ6-sulfanylidene]amino]phenyl] methanol **(3ua)**

Following general procedure **B** and starting from (4-chlorophenyl)methanol (72 mg, 0.50 mmol), and solvent t-BuOH, the title compound was obtained as a yellow liquid (80.8 mg, 0.405 mmol, 81%). Purification by Column chromatography (silica gel, (0–100% ethyl acetate in cyclohexane).

^{1}H NMR (400 MHz, $CDCl_3$): δ = 7.28 - 7.19 (m, 2 H), 7.06 (d, J = 8.3 Hz, 2 H), 4.62 (s., 2 H), 3.15 (s, 6 H), 1.66 (br. s., 1 H) ppm.

^{13}C NMR (101 MHz, $CDCl_3$): δ = 144.8, 134.8, 128.5, 123.6, 65.3, 42.1 ppm.

IR (ATR): $\tilde{\nu}$ = 3628, 3520, 3004, 2293, 2253, 1506, 1420, 1040, 918, 749 cm^{-1}.

HRMS (EI-TOF) [M]$^+$ calcd. for $C_9H_{13}NO_2S$: 199.0666; found: 199.0671.

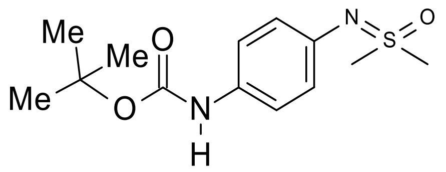

tert-butyl N-[4-[[dimethyl(oxo)-λ6-sulfanylidene]amino]phenyl]carbamate **(3va)**

Following general procedure **B** and starting from tert-butyl N-(4-chlorophenyl)carbamate (114 mg, 0.50 mmol), the title compound was obtained as a brown liquid (135 mg, 0.475 mmol, 95%). Purification by Column chromatography (silica gel, (0–100% ethyl acetate in cyclohexane).

^{1}H NMR (300 MHz, $CDCl_3$): δ = 7.18 (d, J = 8.6 Hz, 2 H), 7.01 - 6.90 (m, 2 H), 6.71 (br. s., 1 H), 3.05 (s, 6 H), 1.46 (s, 9 H) ppm.

^{13}C NMR (75 MHz, $CDCl_3$): δ = 153.2, 140.2, 133.2, 124.0, 120.1, 80.2, 41.7, 28.4 ppm.

IR (ATR): $\tilde{\nu}$ = 3361, 2983, 2962, 1731, 1508, 1228, 1188, 1046, 828, 640 cm^{-1}.

HRMS (EI-TOF) [M]$^+$ calcd. for $C_{13}H_{20}N_2O_3S$: 284.1194; found: 284.1203.

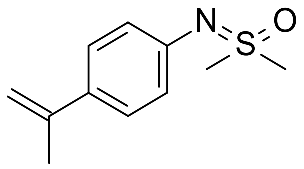

(4-isopropenylphenyl)imino-dimethyl-oxo-λ6-sulfane **(3wa)**

Following general procedure **A** and starting from 1-chloro-4-(prop-1-en-2-yl) benzene (77.4 mg, 0.50 mmol), the title compound was obtained as a yellow liquid (66 mg, 0.315 mmol, 63%). Purification by Column chromatography (silica gel, (0–100% ethyl acetate in cyclohexane).

^{1}H NMR (300 MHz, $CDCl_3$): δ = 7.41 - 7.32 (m, 2 H), 7.08 - 6.98 (m, 2 H), 5.31 (s, 1 H), 5.02 - 4.96 (m, 1 H), 3.15 (s, 6 H), 2.12 (s, 3 H) ppm.

^{13}C NMR (75 MHz, $CDCl_3$): δ = 144.7, 142.8, 135.2, 126.5, 123.1, 110.9, 42.2, 21.9 ppm.

IR (ATR): $\tilde{\nu}$ = 3005, 2914, 1622, 1595, 1503, 1258, 1182, 1048, 936, 836 cm^{-1}.

HRMS (EI-TOF) [M]$^+$ calcd. for $C_{11}H_{15}NOS$: 209.0874; found: 209.0869.

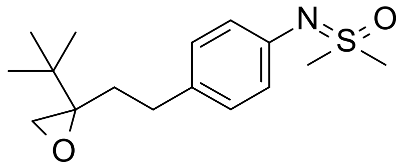

[4-[2-(2-tert-butyloxiran-2-yl)ethyl]phenyl]imino-dimethyl-oxo-λ6-sulfane **(3xa)**

Following general procedure **B** and starting from 2-tert-Butyl-2-[2-(4-chlorophenyl)ethyl]oxirane (124 mg, 0.50 mmol), the title compound was obtained as a yellow solid (133.2 mg, 0.450 mmol, 90%). Purification by Column chromatography (silica gel, (0–100% ethyl acetate in cyclohexane).

m.p.: 114-116 °C.

^{1}H NMR (300 MHz, $CDCl_3$): δ = 7.06 - 7.00 (m, 2 H), 7.00 - 6.93 (m, 2 H), 3.11 (s, 6 H), 2.74 (d, J = 4.2 Hz, 1 H), 2.64 (d, J = 4.2 Hz, 1 H), 2.57 - 2.28 (m, 2 H), 2.19 - 1.85 (m, 2 H), 0.95 (s, 9 H) ppm.

^{13}C NMR (75 MHz, $CDCl_3$): δ = 142.8, 136.0, 129.1, 123.5, 63.5, 48.0, 42.0, 34.0, 31.6, 30.0, 26.1 ppm.

IR (ATR): $\tilde{\nu}$ = 2966, 2925, 1607, 1460, 1433, 1278, 1265, 1187,1056, 833 cm^{-1}.

HRMS (EI-TOF) $[M]^+$ calcd. for $C_{16}H_{25}NO_2S$: 295.1606; found: 295.1617.

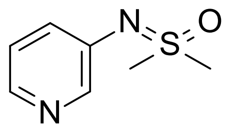

dimethyl-oxo-(3-pyridylimino)-λ⁶-sulfane [CAS: 1621962-58-0] **(3ya)**

Following general procedure **B** and starting from 3-chloropyridine (56.8 mg, 0.50 mmol), the title compound was obtained as orange liquid (74.1 mg, 0.435 mmol, 87%). Purification by Column chromatography (silica gel +1% Et_3N, (0–10% methanol +1% Et_3N in ethyl acetate).

^{1}H NMR (300 MHz, $CDCl_3$): δ = 8.35 (d, J = 2.6 Hz, 1 H), 8.22 (dd, J = 1.4, 4.7 Hz, 1 H), 7.43 - 7.36 (m, 1 H), 7.18 - 7.09 (m, 1 H), 3.16 (s, 6 H) ppm.

^{13}C NMR (75 MHz, $CDCl_3$): δ = 145.4, 143.4, 141.9, 129.9, 123.8, 42.4 ppm.

IR (ATR): $\tilde{\nu}$ = 2992, 2914, 1716, 1577, 1324, 1197, 1054, 1031, 938, 804 cm^{-1}.

HRMS (EI-TOF) $[M]^+$ calcd. for $C_7H_{10}N_2OS$: 170.0513; found: 170.0511.

The NMR data are similar to those reported in literature.[1]

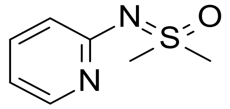

dimethyl-oxo-(2-pyridylimino)-λ⁶-sulfane [CAS: 2059938-84-8] **(3za)**

Following general procedure **B** and starting from 2-chloropyridine (57.3 mg, 0.50 mmol), the title compound was obtained as a yellow solid (75.52 mg, 0.443 mmol, 89%). Purification by Column chromatography (silica gel, 0–10% methanol in ethyl acetate).

m.p.: 114-116 °C.

^{1}H NMR (300 MHz, $CDCl_3$): δ = 8.20 - 8.14 (m, 1 H), 7.53 - 7.44 (m, 1 H), 6.80 - 6.71 (m, 2 H), 3.35 (s, 6 H) ppm.

^{13}C NMR (75 MHz, $CDCl_3$): δ = 159.5, 147.7, 138.0, 116.7, 116.0, 42.8 ppm.

IR (ATR): $\tilde{\nu}$ = 3010, 2923, 1588, 1503, 1463, 1432, 1325, 1181, 1055, 1031 cm^{-1}.

HRMS (EI-TOF) $[M]^+$ calcd. for $C_7H_{10}N_2OS$: 170.0513; found: 170.0508.

The NMR data are similar to those reported in literature.[2]

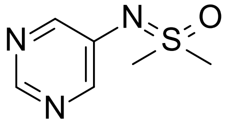

dimethyl-oxo-pyrimidin-5-ylimino-λ⁶-sulfane [CAS: 2377300-50-8] **(4)**

Following general procedure **B** and starting from 5-chloropyrimidine (58.4 mg, 0.50 mmol), the title compound was obtained as a yellow sticky liquid (59.91 mg, 0.349 mmol, 70%). Purification by Column chromatography (silica gel, 0–10% methanol in ethyl acetate).

^{1}H NMR (300 MHz, $CDCl_3$): δ = 8.82 (s, 1 H), 8.49 (s, 2 H), 3.21 (s, 6 H) ppm.

^{13}C NMR (75 MHz, $CDCl_3$): δ = 152.2, 150.6, 140.8, 42.7 ppm.

IR (ATR): $\tilde{\nu}$ = 3371, 3029, 2986, 2912, 1566, 1417, 1294, 1175, 1048, 719 cm^{-1}.

HRMS (EI-TOF) $[M]^+$ calcd. for $C_6H_9N_3OS$: 171.0466; found: 171.0462.

The NMR data are similar to those reported in literature.[3]

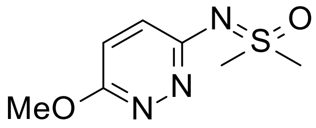

(6-methoxypyridazin-3-yl)imino-dimethyl-oxo-λ^6-sulfane (**5)**

Following general procedure **B** and starting from 3-chloro-6-methoxypyridazine (72.6 mg, 0.50 mmol), the title compound was obtained as a yellow sticky liquid (67.93 mg, 0.337 mmol, 68%). Purification by Column chromatography (silica gel, 0–10% methanol in ethyl acetate).

^{1}H NMR (300 MHz, $CDCl_3$): δ = 6.98 - 6.82 (m, 2 H), 4.02 (s, 3 H), 3.39 (s, 6 H) ppm.

^{13}C NMR (75 MHz, $CDCl_3$): δ = 161.0, 158.0, 126.8, 120.5, 54.5, 42.5 ppm.

IR (ATR): $\tilde{\nu}$ = 3013, 2927, 1463, 1414, 1433, 1351, 1274, 1188, 1045, 847 cm^{-1}.

HRMS (EI-TOF) $[M]^+$ calcd. for $C_7H_{11}N_3O_2S$: 201.0571; found: 201.0567.

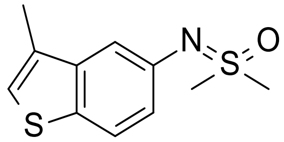

dimethyl-(3-methylbenzothiophen-5-yl)imino-oxo-λ^6-sulfane **(6)**

Following general procedure **B** and starting from 1-chloronaphtalene (94.2 mg, 0.50 mmol), the title compound was obtained as a brown sticky liquid (105 mg, 0.439 mmol, 88%). Purification by Column chromatography (silica gel, (0–100% ethyl acetate in cyclohexane).

^{1}H NMR (400 MHz, $CDCl_3$): δ = 7.69 (d, J = 8.5 Hz, 1 H), 7.41 (d, J = 3.0 Hz, 1 H), 7.13 (dd, J = 1.8, 8.0 Hz, 1 H), 7.04 (s, 1 H), 3.16 (s, 6 H), 2.39 (d, J = 1.0 Hz, 3 H) ppm.

^{13}C NMR (101 MHz, $CDCl_3$): δ = 141.7, 141.0, 134.6, 132.0, 123.3, 122.3, 121.7, 115.9, 42.0, 14.1 ppm.

IR (ATR): $\tilde{\nu}$ = 3005, 2993, 2916, 1590, 1438, 1293, 1267, 1053, 936, 835 cm^{-1}.

HRMS (EI-TOF) $[M]^+$ calcd. for $C_{11}H_{13}NOS_2$: 239.0438; found: 239.0438.

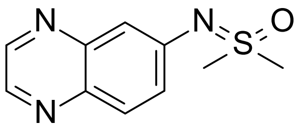

dimethyl-oxo-quinoxalin-6-ylimino-λ^6-sulfane [CAS: 3067858-92-5] **(7)**

Following general procedure **B** and starting from 6-chloroquinoxaline (84.8 mg, 0.50 mmol), the title compound was obtained as a white solid (99.8 mg, 0.451 mmol, 90%). Purification by Column chromatography (silica gel, (0–100% ethyl acetate in cyclohexane).

m.p.: 138-139 °C.

^{1}H NMR (400 MHz, $CDCl_3$): δ = 8.74 (s, 1 H), 8.68 (s, 1 H), 7.97 (d, J = 8.5 Hz, 1 H), 7.73 (d, J = 2.1 Hz, 1 H), 7.52 (dd, J = 2.3, 8.5 Hz, 1 H), 3.27 (s, 6 H) ppm.

^{13}C NMR (101 MHz, $CDCl_3$): δ = 148.0, 144.7, 144.2, 142.5, 139.8, 130.2, 129.5, 117.8, 42.5 ppm.

IR (ATR): $\tilde{\nu}$ = 3685, 3670, 2988, 2970, 2911, 2366, 1966, 1489, 1233, 1076 cm^{-1}.

HRMS (EI-TOF) $[M]^+$ calcd. for $C_{10}H_{11}N_3OS$: 221.0622; found: 221.0632.

The NMR data are similar to those reported in literature.[3]

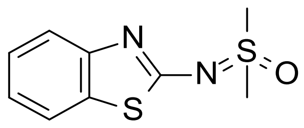

1,3-benzothiazol-2-ylimino-dimethyl-oxo-λ⁶-sulfane [CAS: 3067858-93-6] **(8)**

Following general procedure **B** and starting from 2-chloro-1,3-benzothiazole (84.8 mg, 0.50 mmol), the title compound was obtained as a yellow solid (110 mg, 0.486 mmol, 97%). Purification by Column chromatography (silica gel, (0–100% ethyl acetate in cyclohexane).

m.p.: 107-108 °C.

^{1}H NMR (400 MHz, $CDCl_3$): δ = 7.67 (d, J = 7.8 Hz, 1 H), 7.62 (dd, J = 0.6, 7.8 Hz, 1 H), 7.34 - 7.27 (m, 1 H), 7.19 - 7.12 (m, 1 H), 3.41 (s, 6 H) ppm.

^{13}C NMR (101 MHz, $CDCl_3$): δ = 167.8, 151.9, 133.1, 125.6, 122.8, 120.9, 120.4, 42.0 ppm.

IR (ATR): $\tilde{\nu}$ = 3023, 2924, 2215, 1592, 1477, 1286, 1204, 1068, 989, 754 cm^{-1}.

HRMS (EI-TOF) $[M]^+$ calcd. for $C_9H_{10}N_2OS_2$: 226.0234; found: 226.0233.

The NMR data are similar to those reported in literature.[3]

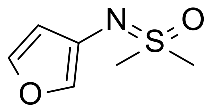

3-furylimino-dimethyl-oxo-λ⁶-sulfane **(9)**

Following general procedure **B** and starting from 3-bromofuran (74.2 mg, 0.50 mmol), the title compound was obtained as a brown liquid (68 mg, 0.427 mmol, 85%). Purification by Column chromatography (silica gel, (0–100% ethyl acetate in cyclohexane).

^{1}H NMR (300 MHz, $CDCl_3$): δ = d = 7.16 - 7.04 (m, 2 H), 6.17 - 6.08 (m, 1 H), 2.96 (s, 6 H) ppm.

^{13}C NMR (75 MHz, $CDCl_3$): δ = 142.4, 132.3, 130.1, 109.5, 41.4 ppm.

IR (ATR): $\tilde{\nu}$ = 3141, 3011, 2934, 1753, 1568, 1504, 1276, 1192, 1081, 766 cm^{-1}.

HRMS (EI-TOF) $[M]^+$ calcd. for $C_6H_9NO_2S$: 159.0353; found: 159.0350.

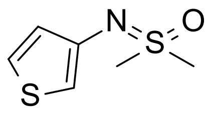

dimethyl-oxo-(3-thienylimino)-λ⁶-sulfane [CAS: 2377300-49-5] **(10)**

Following general procedure **B** and starting from 3-chlorothiophene (60.5 mg, 0.50 mmol), the title compound was obtained as a yellow liquid (78 mg, 0.445 mmol, 89%). Purification by Column chromatography (silica gel, (0–10% methanol in ethyl acetate).

^{1}H NMR (300 MHz, $CDCl_3$): δ = 7.17 (dd, J = 3.1, 5.0 Hz, 1 H), 6.82 (dd, J = 1.4, 5.0 Hz, 1 H), 6.64 (dd, J = 1.4, 3.1 Hz, 1 H), 3.11 (s, 6 H) ppm.

^{13}C NMR (75 MHz, $CDCl_3$): δ = 142.9, 125.5, 124.6, 109.0, 41.5 ppm.

IR (ATR): $\tilde{\nu}$ = 3099, 3013, 2920, 1513, 1407, 1367, 1241, 1193, 1043, 824 cm^{-1}.

HRMS (EI-TOF) $[M]^+$ calcd. for $C_6H_9NOS_2$: 175.0125; found: 175.0131.

The NMR data are similar to those reported in literature.[2]

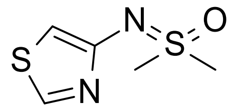

dimethyl-oxo-thiazol-4-ylimino-λ⁶-sulfane **(11)**

Following general procedure **B** and starting from 4-bromo-1,3-thiazole (82 mg, 0.50 mmol), the title compound was obtained as a brown solid (81 mg, 0.460 mmol, 92%). Purification by Column chromatography (silica gel, (0–10% methanol in ethyl acetate).

m.p.: 85-87 °C.

^{1}H NMR (300 MHz, $CDCl_3$): δ = 8.60 (d, *J* = 2.3 Hz, 1 H), 6.43 (d, *J* = 2.2 Hz, 1 H), 3.30 (s, 6 H) ppm.

^{13}C NMR (75 MHz, $CDCl_3$): δ = 155.5, 150.7, 99.5, 42.9 ppm.

IR (ATR): $\tilde{\nu}$ = 3114, 3008, 1624, 1498, 1323, 1251, 1184, 1048, 940, 873 cm^{-1}.

HRMS (EI-TOF) $[M]^+$ calcd. for $C_5H_8N_2OS_2$: 176.0078; found: 176.0074.

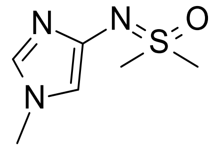

dimethyl-(1-methylimidazol-4-yl)imino-oxo-λ⁶-sulfane **(12)**

Following general procedure **B** and starting from 4-bromo-1-methyl-1H-imidazole (80.5 mg, 0.50 mmol), the title compound was obtained as a yellow liquid (75.89 mg, 0.438 mmol, 88%). Purification by Column chromatography (silica gel, (0–20% methanol in ethyl acetate).

^{1}H NMR (300 MHz, $CDCl_3$): δ = 7.09 (s, 1 H), 6.34 - 6.32 (m, 1 H), 3.51 - 3.50 (m, 3 H), 3.16 - 3.14 (m, 6 H)

^{13}C NMR (75 MHz, $CDCl_3$): δ = 143.3, 134.0, 107.7, 42.2, 33.6 ppm.

IR (ATR): $\tilde{\nu}$ = 3132, 3011, 2924, 1713, 1585, 1545, 1350, 1208, 1065, 936 cm^{-1}.

HRMS (EI-TOF) $[M]^+$ calcd. for $C_6H_{11}N_3OS$: 173.0622; found: 173.0620.

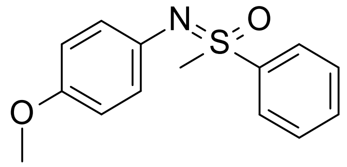

(4-methoxyphenyl)imino-methyl-oxo-phenyl-λ⁶-sulfane [CAS: 83706-37-0] **(3ab)**

Following general procedure, **A** (reaction duration 18h) and starting from 1-chloro-4-methoxybenzene (71.4 mg, 0.50 mmol) and imino-methyl-oxo-phenyl-λ⁶-sulfane (1.20 equiv), the title compound was obtained as a brown solid (128.19 mg, 0.490 mmol, 98%). Purification by Column chromatography (silica gel, (0–100% ethyl acetate in cyclohexane).

m.p.: 64-66 °C.

^{1}H NMR (400 MHz, $CDCl_3$): δ = 7.97 (d, *J*=8.0 Hz, 2 H), 7.62 - 7.48 (m, 3 H), 6.96 (d, *J*=8.8 Hz, 2 H), 6.68 (d, *J*=8.8 Hz, 2 H), 3.70 (s, 3 H), 3.22 (s, 3 H) ppm.

^{13}C NMR (101 MHz, $CDCl_3$): δ = 155.0, 139.6, 137.9, 133.3, 129.6, 128.9, 124.5, 114.5, 55.5, 45.8 ppm.

IR (ATR): $\tilde{\nu}$ = 2966, 2922, 1717, 1501, 1408, 1261, 1191, 1090, 1043, 827 cm^{-1}.

HRMS (EI-TOF) $[M]^+$ calcd. for $C_{14}H_{15}NO_2S$: 261.0823; found: 261.0818.

The NMR data are similar to those reported in literature.[4]

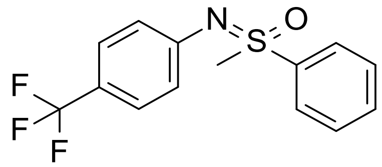

methyl-oxo-phenyl-[4-(trifluoromethyl)phenyl]imino-λ⁶-sulfane [CAS: 257955-73-0] **(3hb)**

Following general procedure, **A** and starting from 1-chloro-4-(trifluoromethyl) benzene (90.5 mg, 0.50 mmol) and imino-methyl-oxo-phenyl-λ⁶-sulfane (1.20 equiv), the title compound was obtained as a yellow solid (140.12 mg, 0.468 mmol, 94%). Purification by Column chromatography (silica gel, (0–100% ethyl acetate in cyclohexane).

m.p.: 112-113 °C.

^{1}H NMR (400 MHz, $CDCl_3$): δ = 7.96 (d, J = 8.0 Hz, 2 H), 7.66 - 7.59 (m, 1 H), 7.59 - 7.51 (m, 2 H), 7.35 (d, J = 8.0 Hz, 2 H), 7.05 (d, J = 8.0 Hz, 2 H), 3.27 (s, 3 H) ppm.

^{13}C NMR (101 MHz, $CDCl_3$): δ = 148.8, 138.9, 133.7, 129.8, 128.6, 126.3 (q, J = 3.1 Hz), 124.9 (q, J = 272.3 Hz), 123.4 (q, J = 32.3 Hz), 122.9, 46.4 ppm.

^{19}F NMR (76 MHz, CDCl3, $C_6H_4F_2$): δ= −61.53 ppm.

IR (ATR): $\tilde{\nu}$ = 3675, 2988, 2971, 2901, 2356, 1968, 1406, 1255,1103, 882 cm^{-1}.

HRMS (EI-TOF) $[M]^+$ calcd. for $C_{14}H_{12}F_3NOS$: 299.0591; found: 299.0597.

The NMR data are similar to those reported in literature.[6]

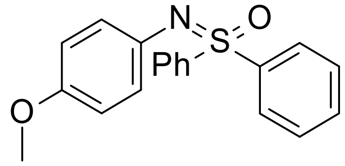

(4-methoxyphenyl)imino-oxo-diphenyl-λ⁶-sulfane [CAS: 1374007-24-5] **(3ac)**

Following general procedure, **A** ([Pd(1-MeNAP)(BrettPhos)]OTf as a catalyst and 100 °C as reaction temperature) and starting from 1-chloro-4-methoxybenzene (71.4 mg, 0.50 mmol), and imino-oxo-diphenyl-λ⁶-sulfane (1.20 equiv), base as K_3PO_4 and t-BuOH as solvent, the title compound was obtained as a white solid (158.8 mg, 0.491 mmol, 98%). Purification by Column chromatography (silica gel, (0–100% ethyl acetate in cyclohexane).

m.p.: 143-145 °C.

^{1}H NMR (300 MHz, $CDCl_3$): δ = 8.08 - 8.01 (m, 4 H), 7.52 - 7.42 (m, 6 H), 7.11 - 7.04 (m, 2 H), 6.74 - 6.66 (m, 2 H), 3.70 (s, 3 H) ppm.

^{13}C NMR (75 MHz, $CDCl_3$): δ = 154.9, 141.1, 137.8, 132.7, 129.4, 128.8, 124.8, 114.4, 55.5 ppm.

IR (ATR): $\tilde{\nu}$ = 2989, 2926, 1499, 1445, 1275, 1224, 1083, 1024, 837, 732 cm^{-1}.

HRMS (EI-TOF) $[M]^+$ calcd. for $C_{19}H_{17}NO_2S$: 323.0979; found: 323.0993.

The NMR data are similar to those reported in literature.[4]

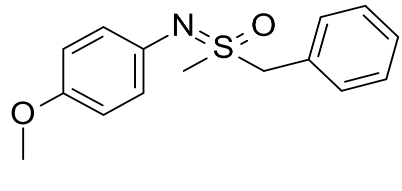

benzyl-(4-methoxyphenyl)imino-methyl-oxo-λ⁶-sulfane **(3ad)**

Following general procedure **A** and starting from 1-chloro-4-methoxybenzene (72.7 mg, 0.50 mmol), and benzyl-imino-oxo-phenyl-λ⁶-sulfane (1.20 equiv), the title compound was obtained as a white solid (69 mg, 0.251 mmol, 50%). Purification by Column chromatography (silica gel, (0–100% ethyl acetate in cyclohexane).

m.p.: 123-125 °C.

^{1}H NMR (300 MHz, $CDCl_3$): δ = 7.43 - 7.31 (m, 5 H), 7.11 - 6.99 (m, 2 H), 6.85 - 6.75 (m, 2 H), 4.53 - 4.33 (m, 2 H), 3.77 (s, 3 H), 2.81 (s, 3 H) ppm.

^{13}C NMR (75 MHz, $CDCl_3$): δ = 155.3, 138.2, 130.9, 129.6, 129.1, 129.0, 124.7, 114.7, 59.8, 55.6, 38.6 ppm.

IR (ATR): $\tilde{\nu}$ = 3684, 2970, 2901, 2365, 1500, 1306, 1259, 1187, 1074, 833 cm^{-1}.

HRMS (EI-TOF) $[M]^+$ calcd. for $C_{15}H_{17}NO_2S$: 275.0979; found: 275.0991.

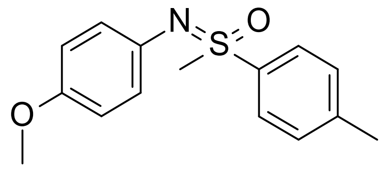

(4-methoxyphenyl)imino-methyl-oxo-(p-tolyl)-λ⁶-sulfane [CAS: 2811710-96-8] **(3ae)**

Following general procedure **A** and starting from 1-chloro-4-methoxybenzene (72.7 mg, 0.50 mmol), and imino-methyl-oxo-(p-tolyl)-λ⁶-sulfane (1.20 equiv), the title compound was obtained as a white solid (124 mg, 0.450 mmol, 90%). Purification by Column chromatography (silica gel, (0–100% ethyl acetate in cyclohexane).

m.p.: 82-85 °C.

^{1}H NMR (300 MHz, $CDCl_3$): δ = 7.82 (d, J = 8.3 Hz, 2 H), 7.29 (d, J = 8.8 Hz, 2 H), 6.95 - 6.91 (m, 2 H), 6.68 - 6.65 (m, 2 H), 3.67 (s, 3 H), 3.18 (s, 3 H), 2.38 (s, 3 H) ppm.

^{13}C NMR (75 MHz, $CDCl_3$): δ = 154.7, 144.1, 138.2, 136.4, 130.2, 128.8, 124.4, 114.3, 55.4, 45.9, 21.6 ppm.

IR (ATR): $\tilde{\nu}$ = 3014, 2957, 2911, 1595, 1503, 1269, 1238, 1190, 1104, 813 cm^{-1}.

HRMS (EI-TOF) $[M]^+$ calcd. for $C_{15}H_{17}NO_2S$: 275.0979; found: 275.0974.

The NMR data are similar to those reported in literature.[5]

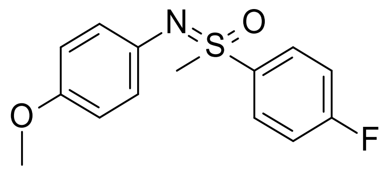

(4-fluorophenyl)-(4-methoxyphenyl)imino-methyl-oxo-λ⁶-sulfane **(3af)**

Following general procedure **A** and starting from 1-chloro-4-methoxybenzene (72.7 mg, 0.50 mmol), and (4-fluorophenyl)-imino-methyl-oxo-λ⁶-sulfane (1.20 equiv), he title compound was obtained as a white sticky liquid (127.82 mg, 0.457 mmol, 92%). Purification by Column chromatography (silica gel, (0–100% ethyl acetate in cyclohexane).

^{1}H NMR (300 MHz, $CDCl_3$): δ = 7.99 - 7.92 (m, 2 H), 7.21 - 7.13 (m, 2 H), 6.96 - 6.91 (m, 2 H), 6.70 - 6.65 (m, 2 H), 3.69 (s, 3 H), 3.20 (s, 3 H) ppm.

^{13}C NMR (75 MHz, $CDCl_3$): δ = 165.6(d, J = 254 Hz,), 154.9, 137.7, 135.3(d, J = 3 Hz), 131.6(d, J = 9.8 Hz), 124.4, 116.8(d, J = 22.5 Hz), 114.4, 55.4, 45.9 ppm.

^{19}F NMR (76 MHz, CDCl3, $C_6H_4F_2$): δ= −104.68 (m) ppm.

IR (ATR): $\tilde{\nu}$ = 3024, 1588, 1493, 1275, 1226, 1195, 1106, 1037, 937, 830 cm^{-1}.

HRMS (EI-TOF) $[M]^+$ calcd. for $C_{14}H_{14}FNO_2S$: 279.0729; found: 279.0725.

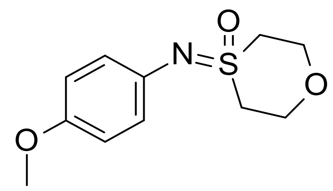

4-(4-methoxyphenyl)imino-1,4-oxathiane 4-oxide **(3ag)**

Following general procedure **A** and starting from 1-chloro-4-methoxybenzene (72.7 mg, 0.50 mmol), and 4-imino-1,4-oxathiane 4-oxide(1.20 equiv), the title compound was obtained as a white sticky liquid (109 mg, 0.452 mmol, 91%). Purification by Column chromatography (silica gel, (0–100% ethyl acetate in cyclohexane).

^{1}H NMR (300 MHz, $CDCl_3$): *δ* = 7.07 - 6.97 (m, 2 H), 6.82 - 6.72 (m, 2 H), 4.21 - 3.94 (m, 4 H), 3.75 (s, 3 H), 3.40 - 3.10 (m, 4 H) ppm.

^{13}C NMR (75 MHz, $CDCl_3$): *δ* = 155.4, 137.1, 124.7, 114.7, 66.3, 55.6, 51.4 ppm.

IR (ATR): $\tilde{\nu}$ = 2933, 2840, 1504, 1454, 1378, 1264, 1173, 1022, 978, 825 cm^{-1}.

HRMS (EI-TOF) $[M]^+$ calcd. for $C_{11}H_{15}NO_3S$: 241.0772; found: 241.0767.

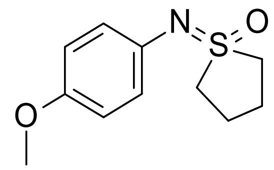

1-(4-methoxyphenyl)iminothiolane 1-oxide **(3ah)**

Following general procedure **A** and starting from 1-chloro-4-methoxybenzene (72.7 mg, 0.50 mmol), and 1-iminothiolane 1-oxide (1.20 equiv), the title compound was obtained as a white sticky liquid (105.02 mg, 0.466 mmol, 93%). Purification by Column chromatography (silica gel, (0–100% ethyl acetate in cyclohexane).

^{1}H NMR (300 MHz, $CDCl_3$): *δ* = 7.03 - 6.93 (m, 2 H), 6.81 - 6.72 (m, 2 H), 3.74 (s, 3 H), 3.41 - 3.26 (m, 2 H), 3.15 - 3.01 (m, 2 H), 2.36 - 2.11 (m, 4 H) ppm.

^{13}C NMR (75 MHz, $CDCl_3$): *δ* = 155.0, 139.0, 123.9, 114.6, 55.5, 52.1, 23.8 ppm.

IR (ATR): $\tilde{\nu}$ = 3035, 2979, 2933, 1570, 1461, 1281, 1181, 1134, 1076, 825 cm^{-1}.

HRMS (EI-TOF) $[M]^+$ calcd. for $C_{11}H_{15}NO_2S$: 225.0823; found: 225.0820.

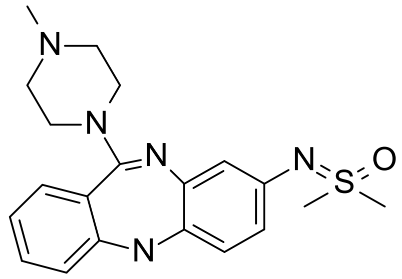

dimethyl-[[6-(4-methylpiperazin-1-yl)-11H-benzo[b][1,4]benzodiazepin-3-yl]imino]-oxo-λ^6-sulfane **(13)**

Following general procedure **B** **(**t-BuOH as solvent) and starting from 3-chloro-6-(4-methylpiperazin-1-yl)-11H-benzo[b][1,4]benzodiazepine (163mg, 0.50 mmol), the title compound was obtained as a yellow solid (170mg, 0.443mmol, 88%). Purification by Column chromatography (silica gel, (0–20% methanol in ethyl acetate).

m.p.: 134-136 °C.

^{1}H NMR (400 MHz, $CDCl_3$): *δ* = 7.34 - 7.26 (m, 2 H), 7.06 - 6.95 (m, 1 H), 6.89 - 6.77 (m, 2 H), 6.65 - 6.51 (m, 2 H), 4.88 (br. s., 1 H), 3.46 (br. s., 4 H), 3.09 (s, 6 H), 2.52 (br. s., 4 H), 2.36 (s, 3 H) ppm.

^{13}C NMR (101 MHz, $CDCl_3$): δ = 162.5, 153.7, 141.4, 141.2, 137.1, 131.7, 130.4, 123.6, 122.7, 122.0, 119.9, 119.8, 119.3, 55.2, 47.3, 46.2, 42.0 ppm.

IR (ATR): $\tilde{\nu}$ = 3669, 2983, 2901, 2366, 2201, 1597, 1456, 1378, 1241, 1051 cm^{-1}.

HRMS (EI-TOF) $[M]^+$ calcd. for $C_{20}H_{25}N_5OS$: 383.1779; found: 383.1793.

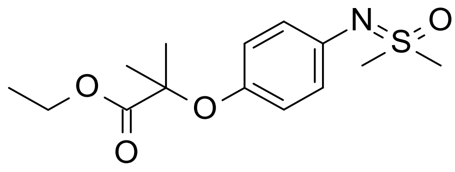

ethyl 2-[4-[[dimethyl(oxo)-λ^6-sulfanylidene]amino]phenoxy]-2-methyl-propanoate **(14)**

Following general procedure **B** and starting from ethyl 2-(4-chlorophenoxy)-2-methylpropanoate (124 mg, 0.50 mmol), the title compound was obtained as a white solid (121 mg, 0.404 mmol, 81%). Purification by Column chromatography (silica gel, (0–100% ethyl acetate in cyclohexane).

m.p.: 113-114 °C.

^{1}H NMR (300 MHz, $CDCl_3$): δ = 6.97 - 6.86 (m, 2 H), 6.80 - 6.69 (m, 2 H), 4.22 (dq, J = 0.9, 7.1 Hz, 2 H), 3.11 (d, J = 1.1 Hz, 6 H), 1.54 (d, J = 0.9 Hz, 6 H), 1.26 (dt, J = 0.9, 7.1 Hz, 3 H) ppm.

^{13}C NMR (75 MHz, $CDCl_3$): δ = 174.5, 150.8, 139.7, 124.2, 121.0, 79.5, 61.4, 42.0, 25.5, 14.2 ppm.

IR (ATR): $\tilde{\nu}$ = 3670, 2968, 2925, 1732, 1607, 1411, 1278, 1186, 1056, 826 cm^{-1}.

HRMS (EI-TOF) $[M]^+$ calcd. for $C_{14}H_{21}NO_4S$: 299.1191; found: 299.1204.

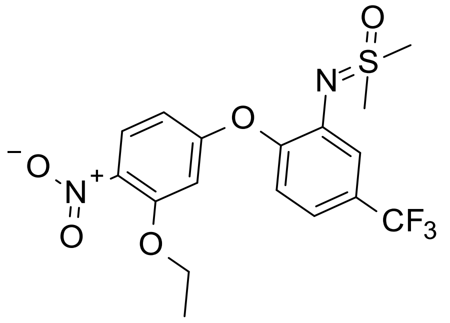

[2-(3-ethoxy-4-nitro-phenoxy)-5-(trifluoromethyl)phenyl]imino-dimethyl-oxo-λ^6-sulfane **(15)**

Following general procedure **B** (t-BuOH as solvent) and starting from 2-chloro-1-(3-ethoxy-4-nitro-phenoxy)-4-(trifluoromethyl)benzene (181 mg, 0.50 mmol), the title compound was obtained as a brown solid (187.78 mg, 0.448 mmol, 90%). Purification by Column chromatography (silica gel, (0–100% ethyl acetate in cyclohexane+ 1% Et_3N).

m.p.: 156-158 °C.

^{1}H NMR (400 MHz, $CDCl_3$): δ = 7.89 - 7.82 (m, 1 H), 7.53 (s, 1 H), 7.24 (s, 1 H), 7.12 (d, J = 8.4 Hz, 1 H), 6.58 (d, J = 2.4 Hz, 1 H), 6.41 - 6.32 (m, 1 H), 4.07 (q, J = 7.0 Hz, 2 H), 3.03 (s, 6 H), 1.48 - 1.39 (m, 3 H)

^{13}C NMR (101 MHz, $CDCl_3$): δ = 162.9, 155.0, 150.1, 138.2, 134.4, 128.7 (q, J = 33.33 Hz), 127.9, 122.6, 121.6 (d, J =4 Hz), 120.1 (d, J =3 Hz), 123.1 (q, J = 273.71 Hz), 107.5, 102.8, 65.7, 43.0, 14.6 ppm.

^{19}F NMR (76 MHz, CDCl3, $C_6H_4F_2$): δ= −61.92 ppm.

IR (ATR): $\tilde{\nu}$ = 3086, 3000, 2984, 1618, 1578, 1420, 1217, 1119, 1048, 847 cm^{-1}.

HRMS (EI-TOF) $[M]^+$ calcd. for $C_{17}H_{17}F_3N_2O_5S$: 418.0810; found: 418.0809.

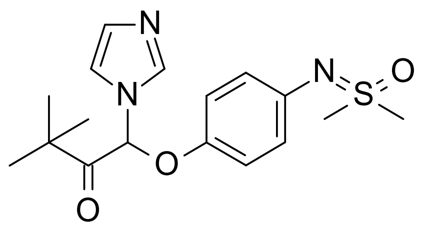

1-[4-[[dimethyl(oxo)-λ⁶-sulfanylidene]amino]phenoxy]-1-imidazol-1-yl-3,3-dimethyl-butan-2-one **(16)**

Following general procedure **B** and starting from 1-(4-chlorophenoxy)-1-(1H-imidazol-1-yl)-3,3-dimethylbutan-2-one (149 mg, 0.50 mmol), the title compound was obtained as a yellow solid (147 mg, 0.421 mmol, 84%). Purification by Column chromatography (silica gel, (0–100% ethyl acetate in cyclohexane).

m.p.: 96-98 °C.

^{1}H NMR (300 MHz, $CDCl_3$): δ = 7.61 (s, 1 H), 7.13 (s, 1 H), 7.07 (s, 1 H), 6.99 - 6.87 (m, 2 H), 6.76 - 6.64 (m, 2 H), 6.38 (s, 1 H), 3.08 (s, 6 H), 1.26 (s, 9 H) ppm.

^{13}C NMR (75 MHz, $CDCl_3$): δ = 204.1, 150.9, 141.7, 136.8, 130.1, 124.7, 118.8, 117.9, 82.8, 44.0, 42.1, 26.3 ppm.

IR (ATR): $\tilde{\nu}$ = 3103, 2977, 1716, 1497, 1472, 1285, 1190, 1068, 988, 838, 743 cm^{-1}.

HRMS (EI-TOF) $[M]^+$ calcd. for $C_{17}H_{23}N_3O_3S$: 349.1460; found: 349.1474.

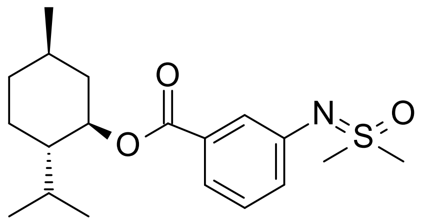

[(1R,2S,5R)-2-isopropyl-5-methyl-cyclohexyl]3-[[dimethyl(oxo)-λ⁶sulfanylidene]amino] benzoate **(17)**

Following general procedure **B** and starting from [(1R,2S,5R)-2-isopropyl-5-methyl-cyclohexyl] 3-chlorobenzoate (155 mg, 0.50 mmol), the title compound was obtained as a white liquid (119 mg, 0.339 mmol, 68%). Purification by Column chromatography (silica gel, (0–100% ethyl acetate in cyclohexane).

^{1}H NMR (300 MHz, $CDCl_3$): δ = 7.96 - 7.80 (m, 2 H), 7.14 - 6.98 (m, 2 H), 4.86 (dt, J = 4.4, 10.8 Hz, 1 H), 3.15 (s, 6 H), 2.15 - 2.01 (m, 1 H), 2.00 - 1.86 (m, 1 H), 1.77 - 1.63 (m, 2 H), 1.62 - 1.41 (m, 2 H), 1.22 - 0.97 (m, 2 H), 0.95 - 0.83 (m, 7 H), 0.76 (d, J = 6.9 Hz, 3 H) ppm.

^{13}C NMR (75 MHz, $CDCl_3$): δ = 166.1, 150.3, 131.0, 124.1, 122.3, 74.4, 47.3, 42.3, 41.1, 34.4, 31.5, 26.5, 23.7, 22.1, 20.8, 16.6 ppm.

IR (ATR): $\tilde{\nu}$ = 2953, 2927, 1734, 1599, 1456, 1309, 1200, 1167, 1040, 771 cm^{-1}.

HRMS (EI-TOF) $[M]^+$ calcd. for $C_{19}H_{29}NO_3S$: 351.1868; found: 351.1876.

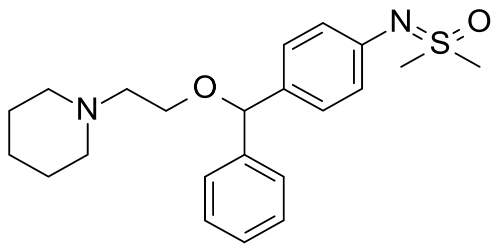

dimethyl-oxo-[4-[phenyl-[2-(1-piperidyl)ethoxy]methyl]phenyl]imino-λ⁶-sulfane **(18)**

Following general procedure **B** and starting from racemic cloperastine (170 mg, 0.50 mmol), the title compound was obtained as a yellow liquid (149 mg, 0.385 mmol, 78%). Purification by Column chromatography (silica gel, (0–100% ethyl acetate in cyclohexane).

^{1}H NMR (300 MHz, $CDCl_3$): δ = 7.42 - 7.26 (m, 5 H), 7.25 - 7.19 (m, 2 H), 7.09 - 6.99 (m, 2 H), 5.36 (s, 1 H), 3.62 (t, J = 6.4 Hz, 2 H), 3.16 (s, 6 H), 2.68 (t, J = 6.4 Hz, 2 H), 2.56 - 2.39 (m, 4 H), 1.60 (quin, J = 5.5 Hz, 4 H), 1.52 - 1.37 (m, 2 H) ppm.

^{13}C NMR (75 MHz, $CDCl_3$): δ = 144.4, 142.6, 136.2, 128.4, 128.2, 127.4, 127.1, 123.3, 83.8, 67.0, 58.7, 55.1, 42.2, 26.1, 24.4 ppm.

IR (ATR): $\tilde{\nu}$ = 2930, 2853, 1734, 1604, 1503, 1326, 1196, 1095, 932, 727 cm^{-1}.

HRMS (EI-TOF) $[M]^+$ calcd. for [$C_{22}H_{30}N_2O_2S$ - $C_7H_{15}N$]: 273.0823; found: 273.0834.

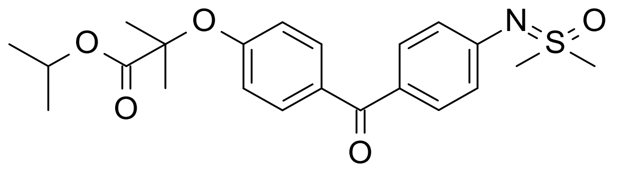

Isopropyl2-[4-[4-[[dimethyl(oxo)-λ^6-sulfanylidene]amino]benzoyl]phenoxy]-2-methyl-propanoate **(19)**

Following general procedure **B** and starting from propan-2-yl 2-[4-(4-chlorobenzoyl)phenoxy]-2-methylpropanoate (184mg, 0.50 mmol), the title compound was obtained as a yellow solid (185 mg, 0.443 mmol, 89%). Purification by Column chromatography (silica gel, (0–100% ethyl acetate in cyclohexane).

m.p.: 135-137 °C.

^{1}H NMR (300 MHz, $CDCl_3$): δ = 7.76 - 7.60 (m, 4 H), 7.15 - 7.04 (m, 2 H), 6.89 - 6.77 (m, 2 H), 5.06 (spt, J = 6.0 Hz, 1 H), 3.19 (s, 6 H), 1.63 (s, 6 H), 1.18 (d, J = 6.3 Hz, 6 H) ppm. (ethyl acetate impurity)

^{13}C NMR (75 MHz, $CDCl_3$): δ = 194.7, 173.3, 159.1, 150.1, 131.8, 131.8, 131.4, 131.4, 122.2, 117.2, 79.4, 69.3, 42.5, 25.4, 21.6 ppm.

IR (ATR): $\tilde{\nu}$ = 2990, 2930, 1712, 1638, 1590, 1417, 1284, 1182, 1045, 768 cm^{-1}.

HRMS (EI-TOF) $[M]^+$ calcd. for $C_{22}H_{27}NO_5S$: 417.1609; found: 417.1631.

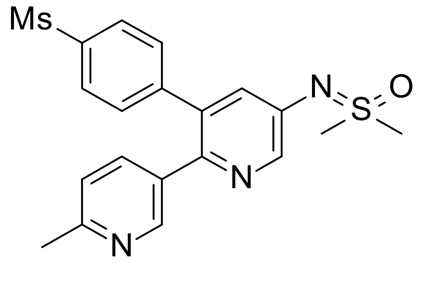

dimethyl-[[6-(6-methyl-3-pyridyl)-5-(4-methylsulfonylphenyl)-3-pyridyl]imino]-oxo-λ^6-sulfane **(20)**

Following general procedure **B** (t-BuOH as solvent) and starting from 5-chloro-2-(6-methyl-3-pyridyl)-3-(4-methylsulfonylphenyl) pyridine (179 mg, 0.50 mmol), the title compound was obtained as a white solid (175 mg, 0.421 mmol, 84%). Purification by Column chromatography (silica gel, (0–10% methanol in ethyl acetate).

m.p.: 217-218 °C.

^{1}H NMR (300 MHz, $CDCl_3$): δ = 8.50 (d, J = 2.5 Hz, 1 H), 8.37 (d, J = 2.2 Hz, 1 H), 7.91 - 7.82 (m, 2 H), 7.55 (dd, J = 2.3, 8.0 Hz, 1 H), 7.44 (d, J = 2.5 Hz, 1 H), 7.42 - 7.36 (m, 2 H), 7.06 (d, J = 8.0 Hz, 1 H), 3.25 (s, 6 H), 3.08 (s, 3 H), 2.52 (s, 3 H) ppm.

^{13}C NMR (75 MHz, $CDCl_3$): δ = 157.5, 149.9, 147.2, 145.4, 144.8, 141.5, 139.5, 137.3, 134.6, 132.3, 131.5, 130.6, 127.7, 122.7, 44.6, 42.7, 24.2 ppm.

IR (ATR): $\tilde{\nu}$ = 3670, 3648, 2988, 2901, 2356, 1405, 1250, 1075, 1066, 895 cm^{-1}.

HRMS (EI-TOF) $[M]^+$ calcd. for $C_{20}H_{21}N_3O_3S_2$: 415.1024; found: 415.1002.

7. NMR-Spectra of product

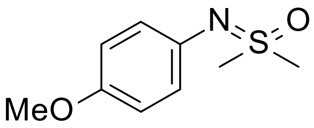

(4-methoxyphenyl)imino-dimethyl-oxo-λ⁶-sulfane [CAS: 58873-28-2] **(3aa)**

^{1}H NMR (400 MHz, $CDCl_3$)

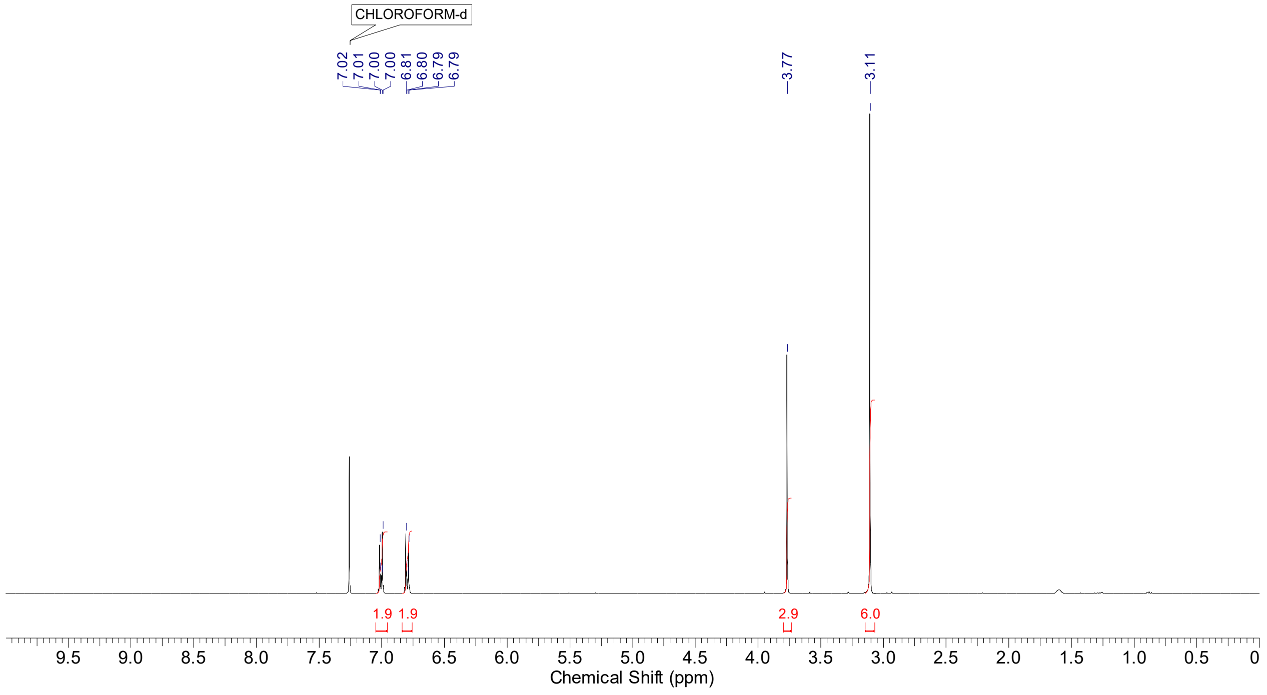

^{13}C NMR (101 MHz, $CDCl_3$)

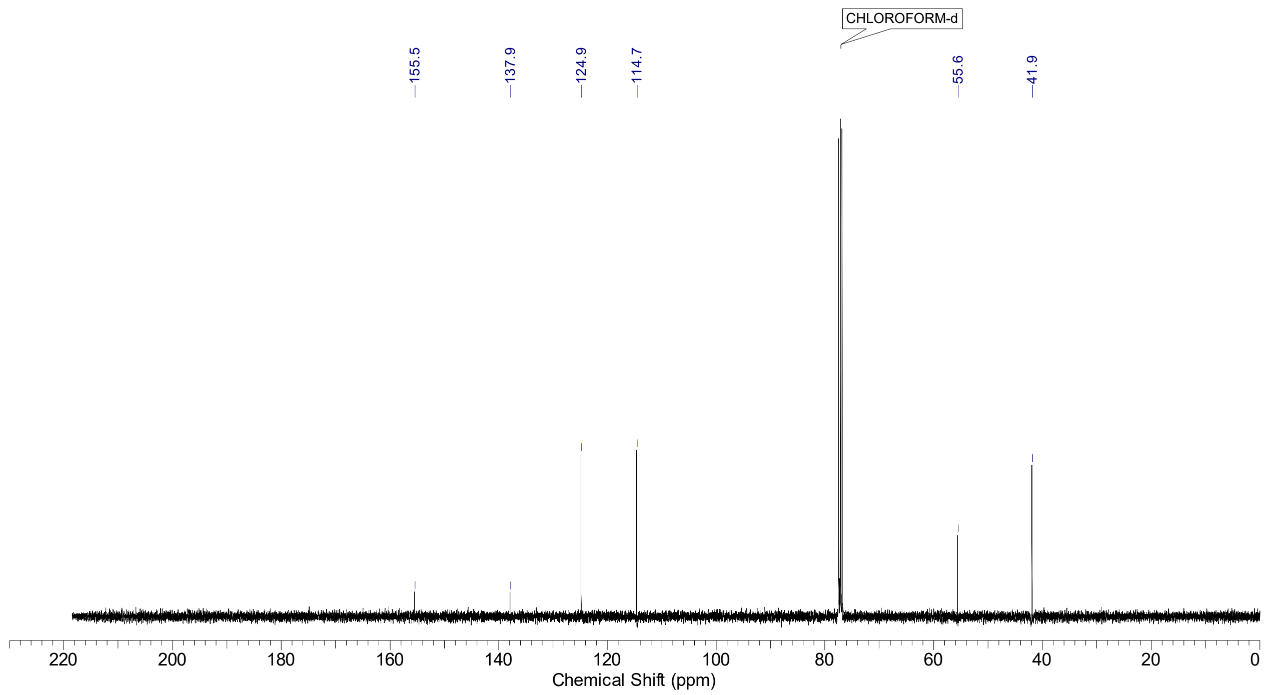

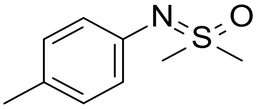

dimethyl-oxo-(p-tolylimino)-λ⁶-sulfane [CAS: 56157-99-4] **(3ba)**

^{1}H NMR (400 MHz, $CDCl_3$)

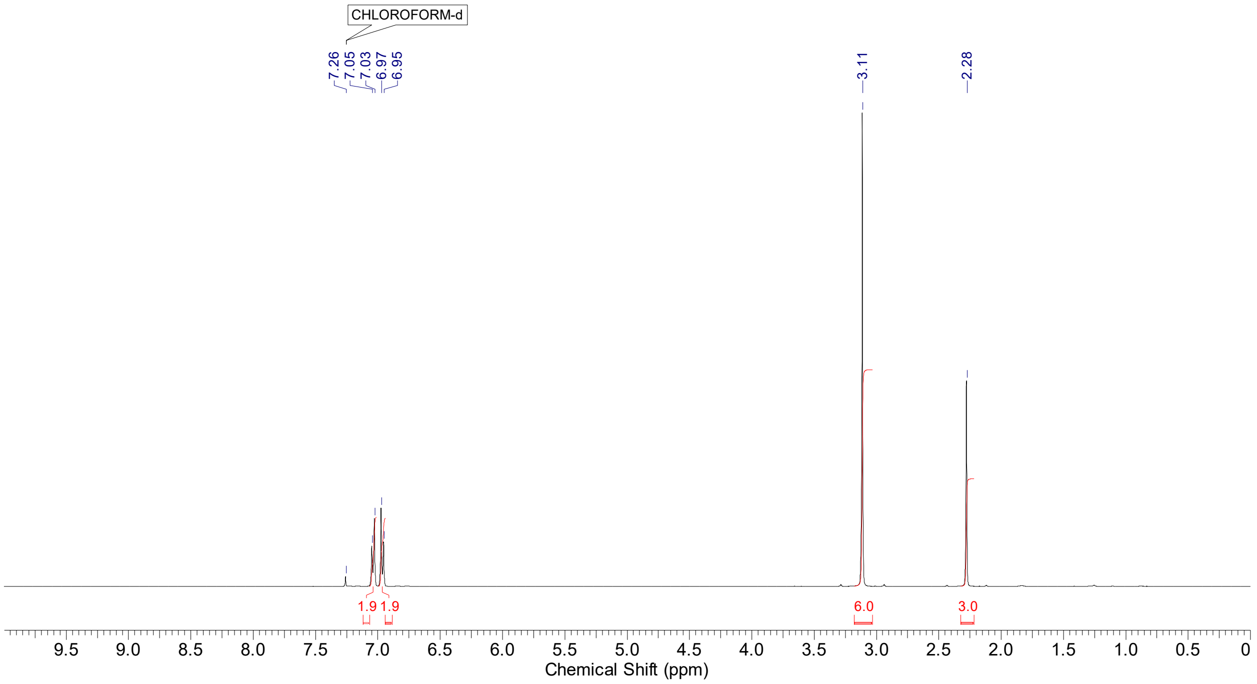

^{13}C NMR (101 MHz, $CDCl_3$)

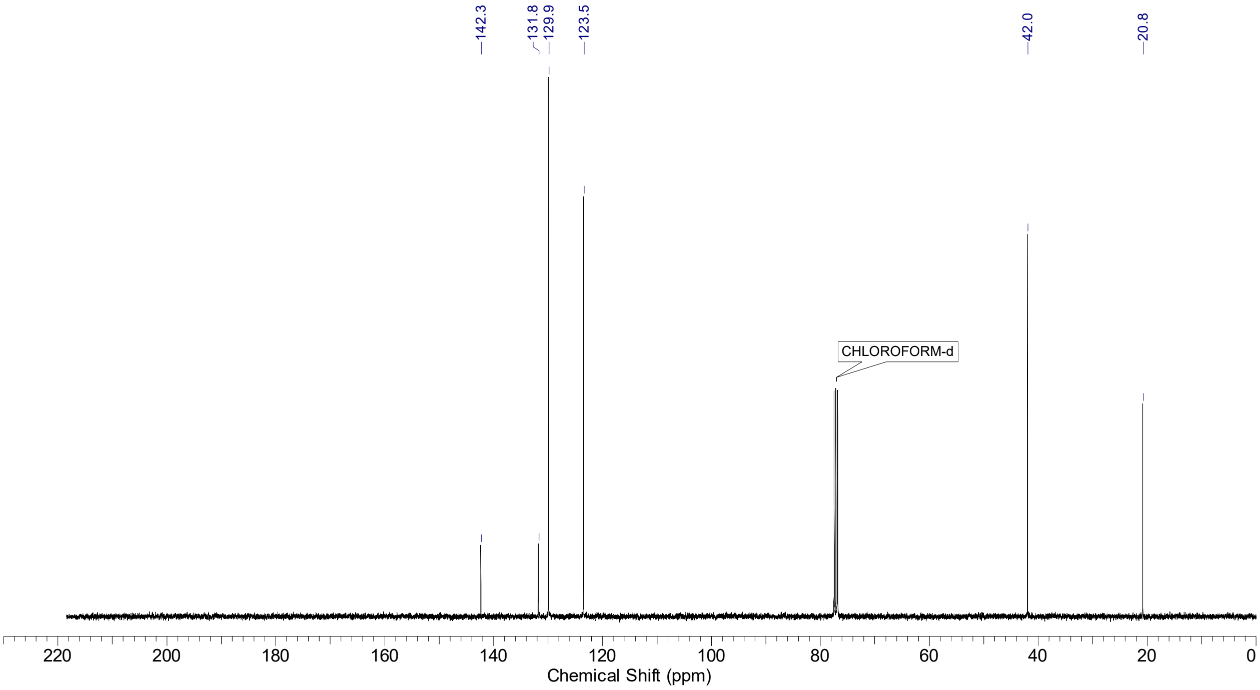

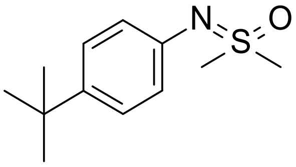

(4-tert-butylphenyl)imino-dimethyl-oxo-λ⁶-sulfane [CAS: 1374007-21-2] **(3ca)**

^{1}H NMR (400 MHz, $CDCl_3$)

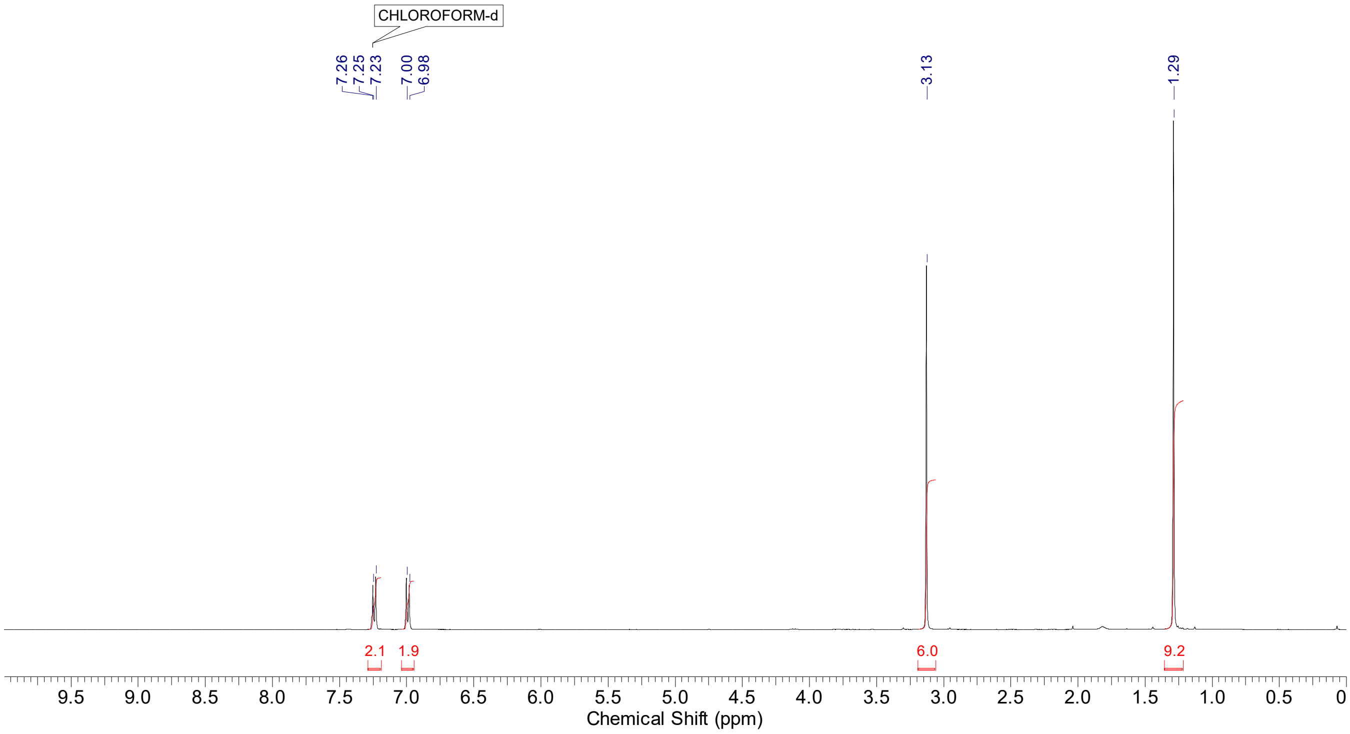

^{13}C NMR (101 MHz, $CDCl_3$)

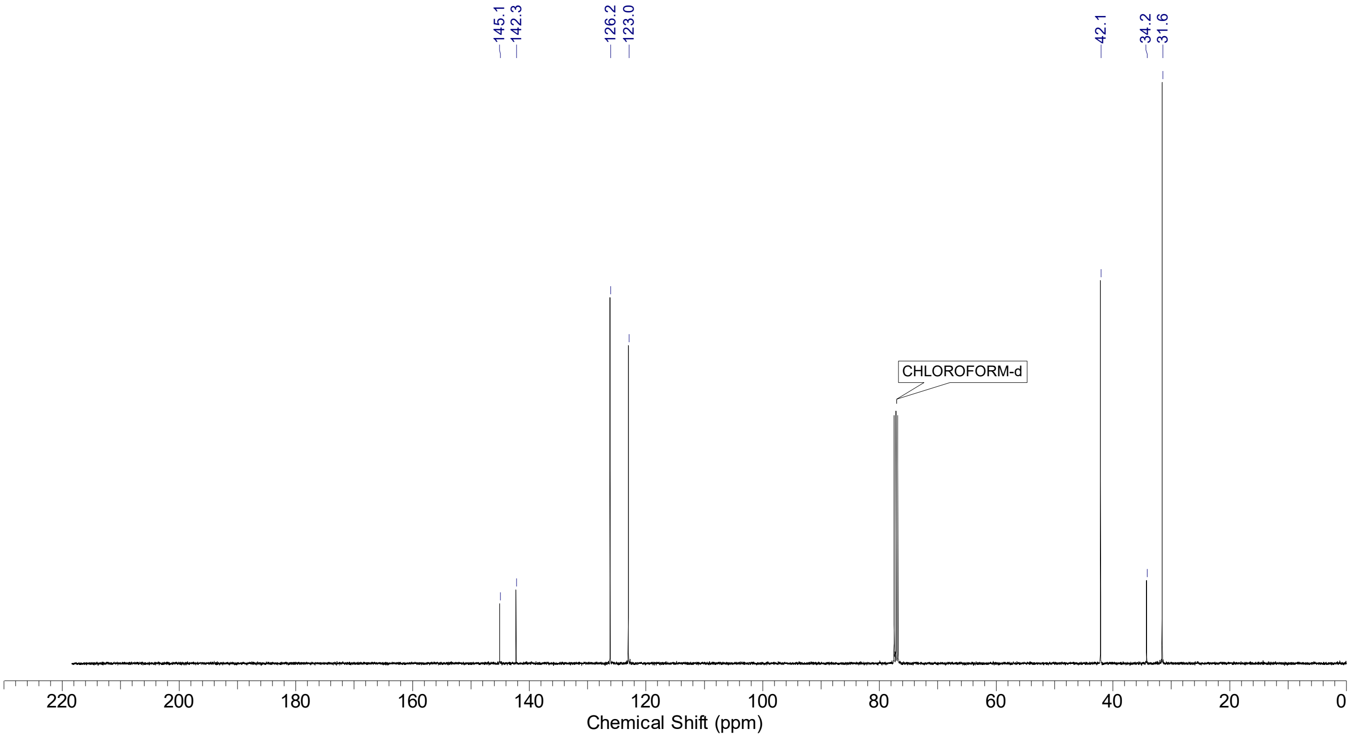

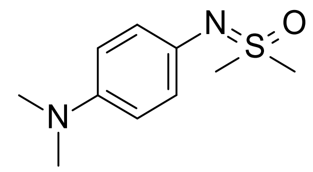

4-[[dimethyl(oxo)-λ⁶-sulfanylidene]amino]-N,N-dimethyl-aniline **(3da)**

^{1}H NMR (400 MHz, $CDCl_3$)

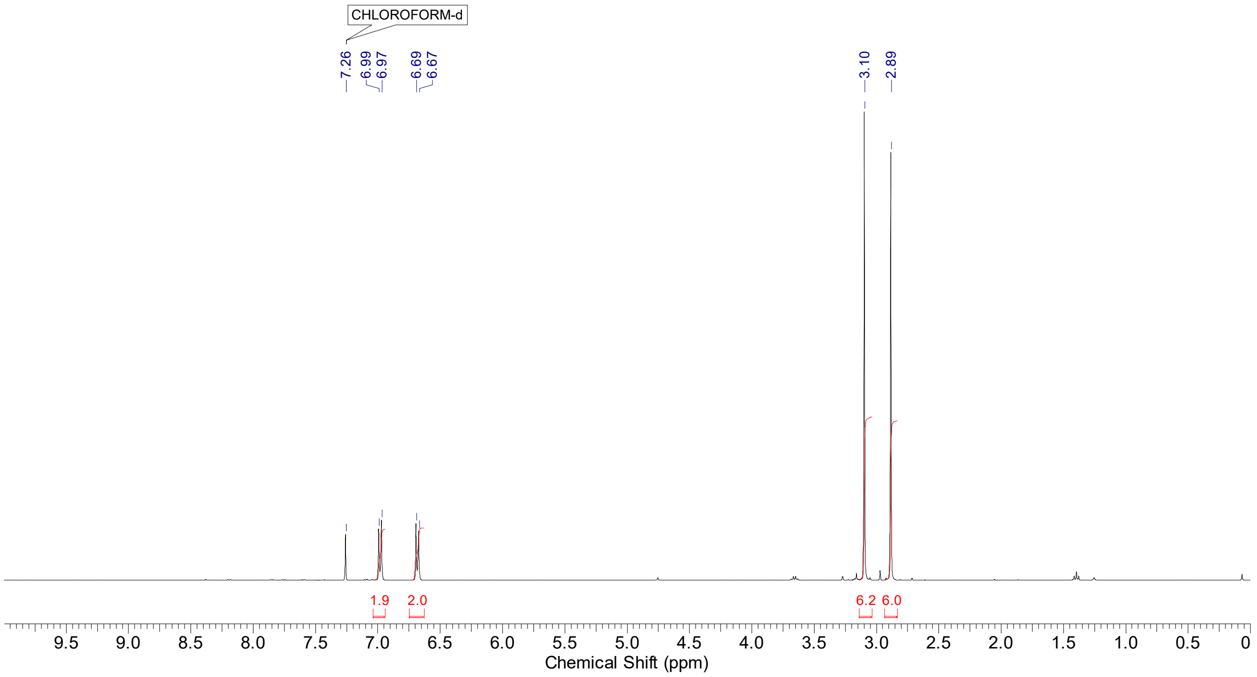

^{13}C NMR (101 MHz, $CDCl_3$)

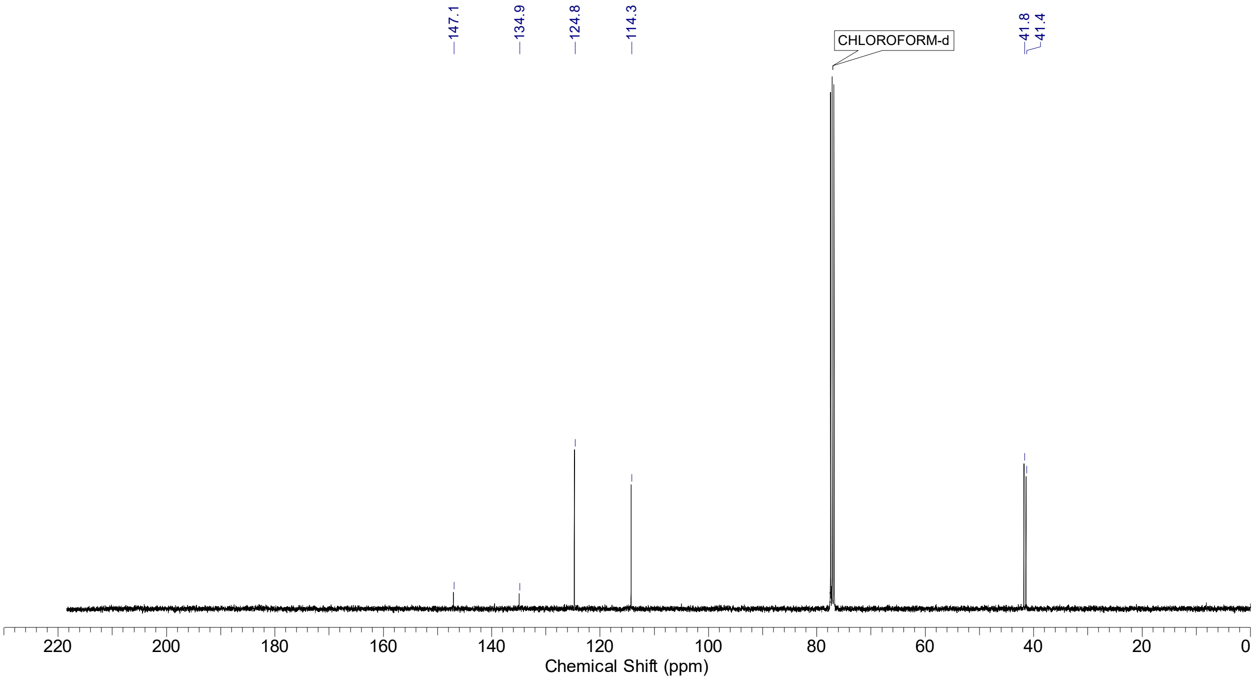

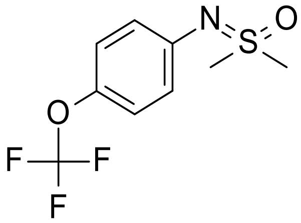

dimethyl-oxo-[4-(trifluoromethoxy)phenyl]imino-λ⁶-sulfane **(3ea)**

^{1}H NMR (300 MHz, $CDCl_3$)

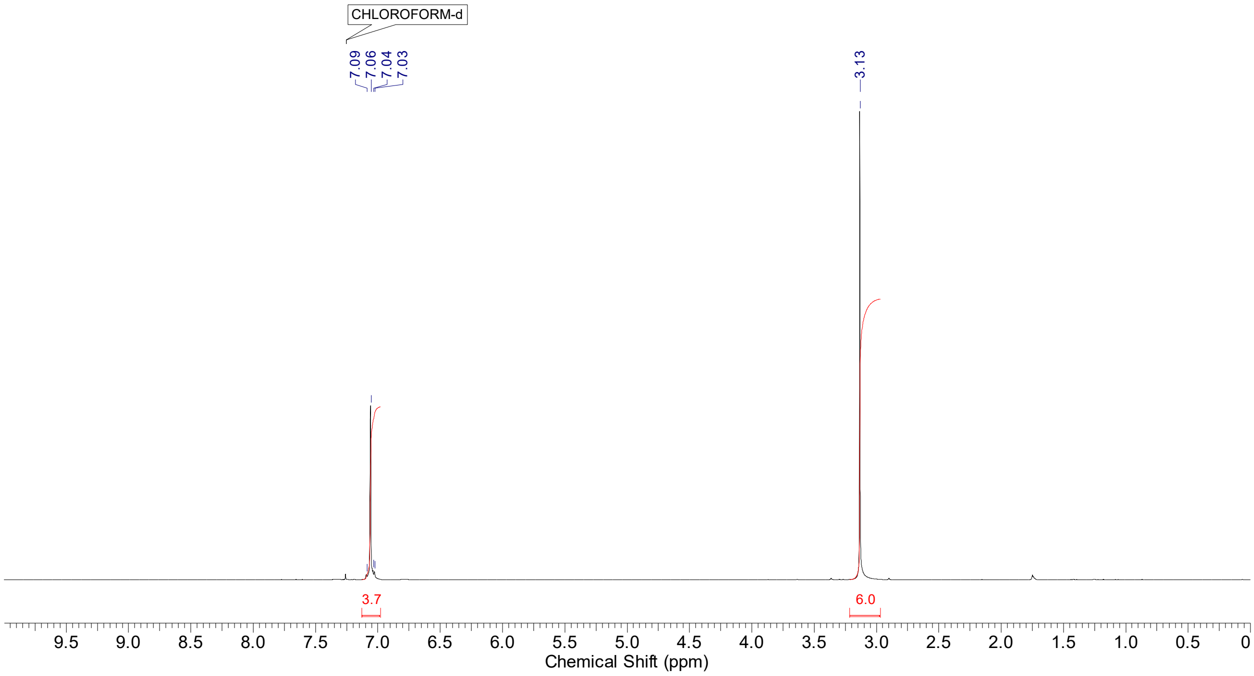

^{13}C NMR (75 MHz, $CDCl_3$)

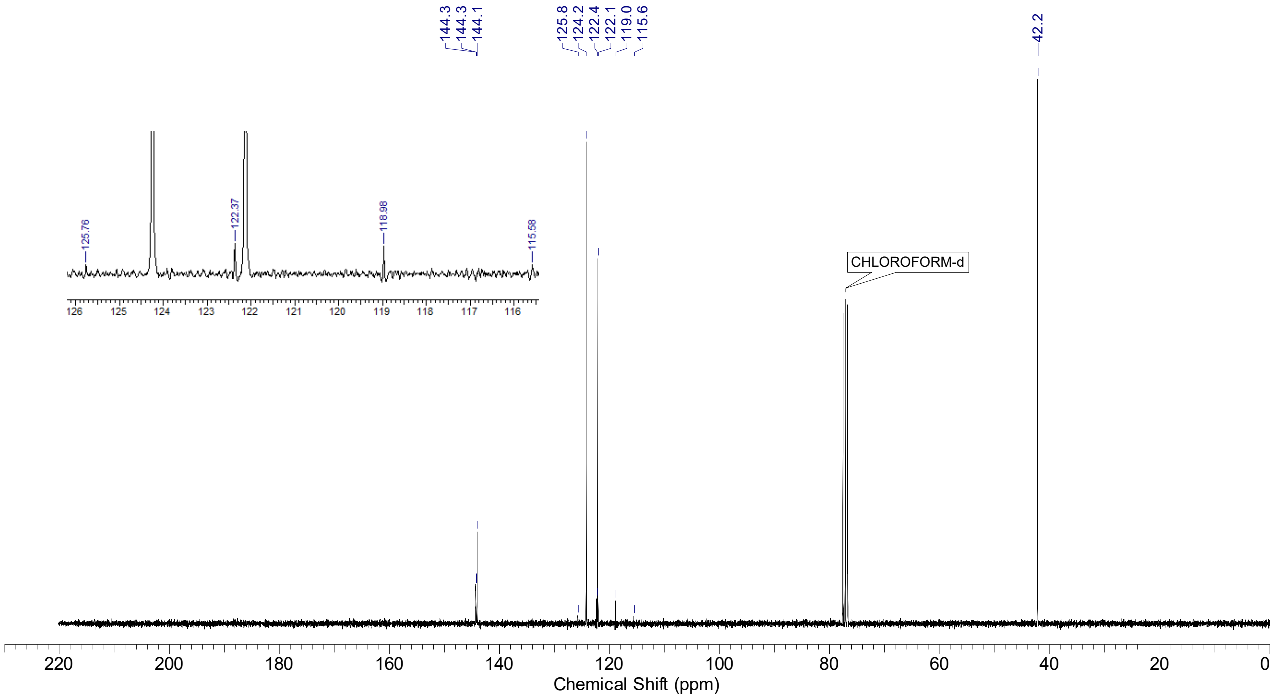

^{19}F NMR (76 MHz, CDCl3, $C_6H_4F_2$)

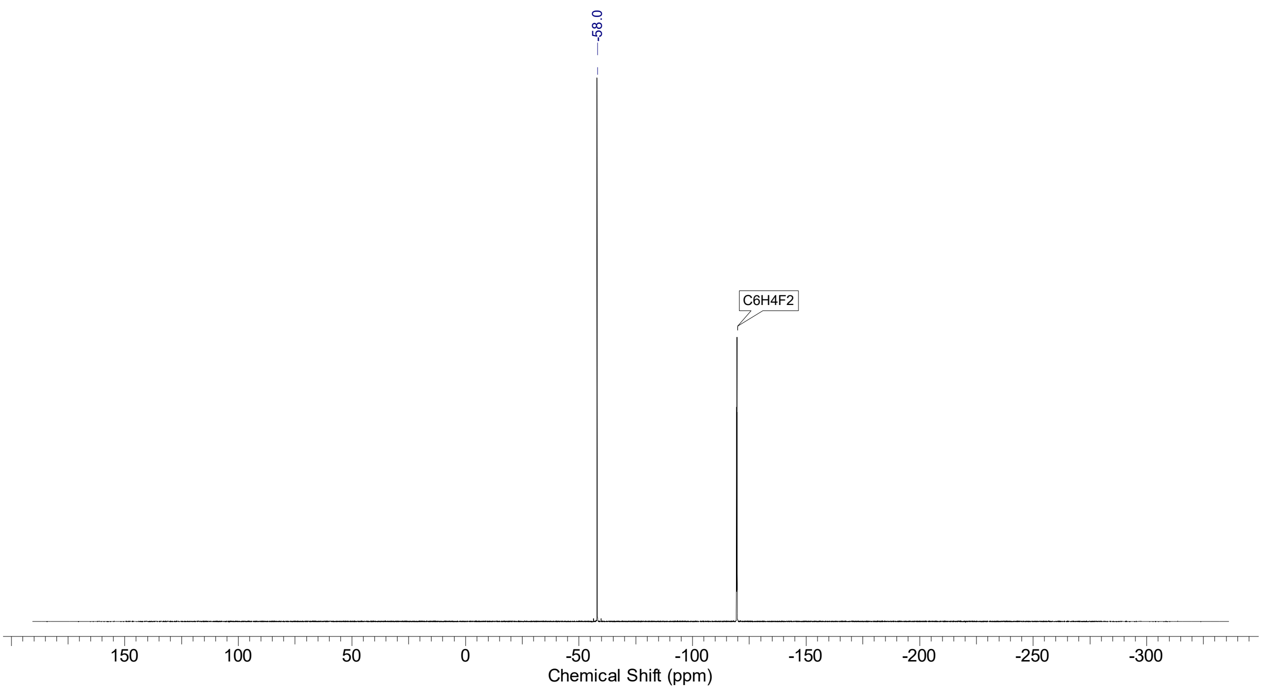

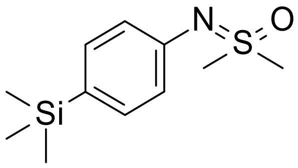

[4-[dimethyl(oxo)-λ^6-sulfanylidene]amino]phenyl]-trimethyl-silane] **(3fa)**

^{1}H NMR (400 MHz, $CDCl_3$)

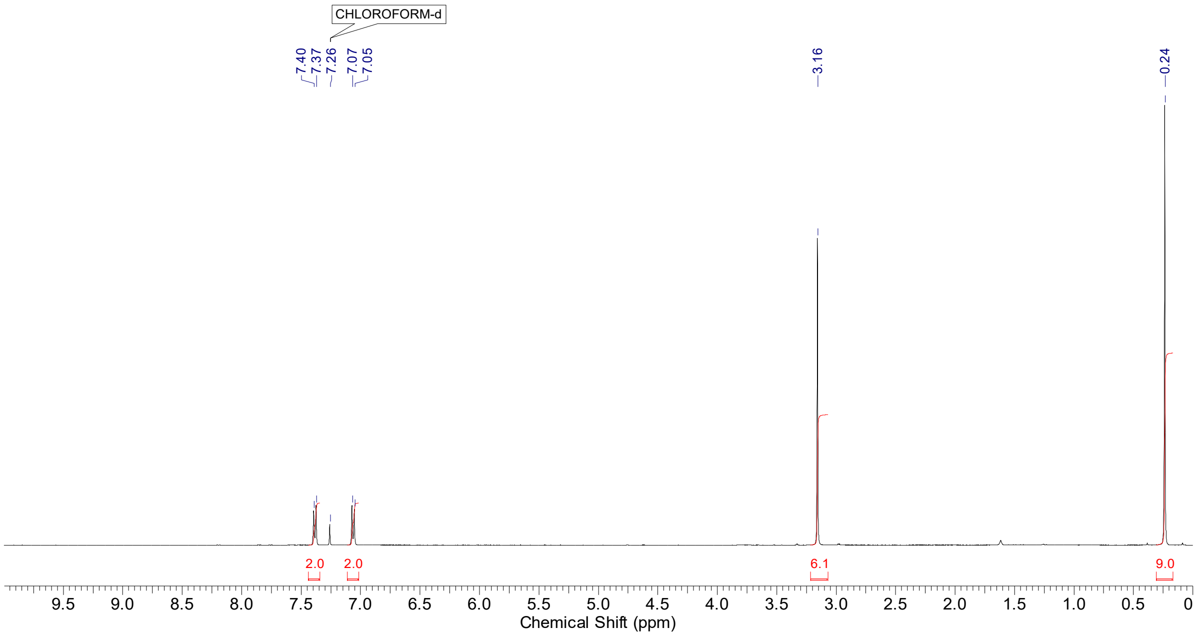

^{13}C NMR (101 MHz, $CDCl_3$)

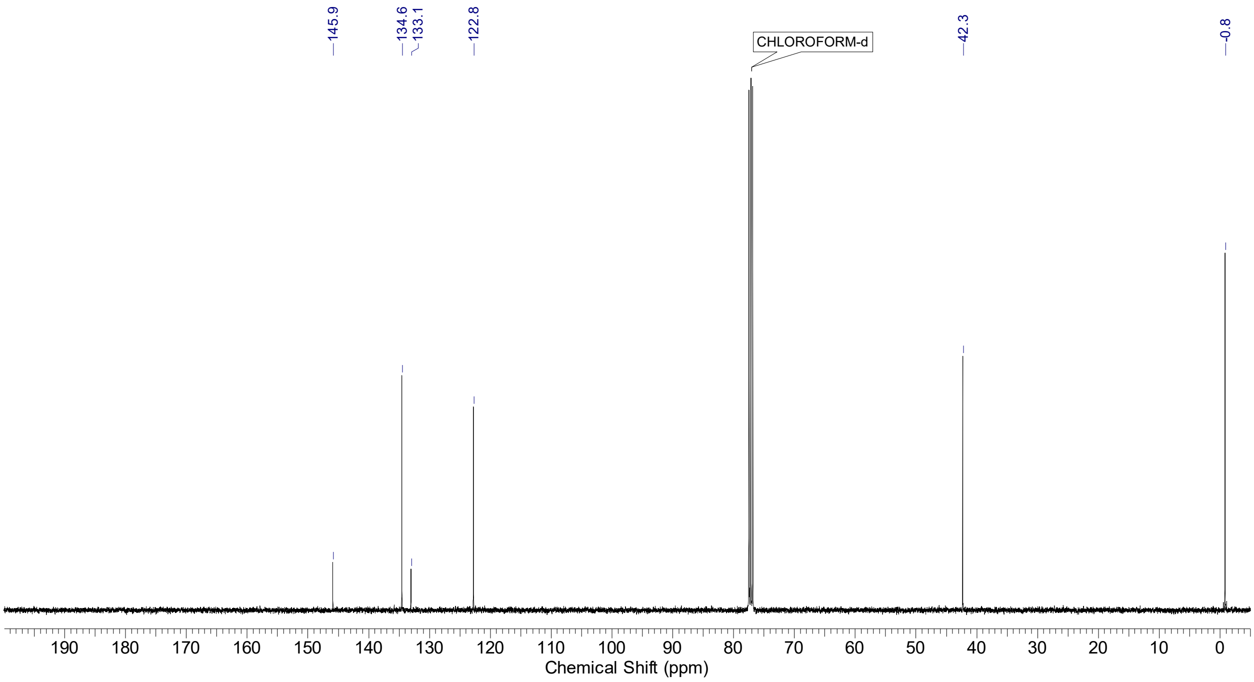

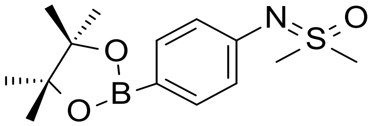

Dimethyl-oxo-[4-(4,4,5,5-tetramethyl-1,3,2-dioxaborolan-2-yl)phenyl]imino-λ⁶-sulfane[CAS: 1644499-93-3] **(3ga)**

^{1}H NMR (300 MHz, $CDCl_3$)

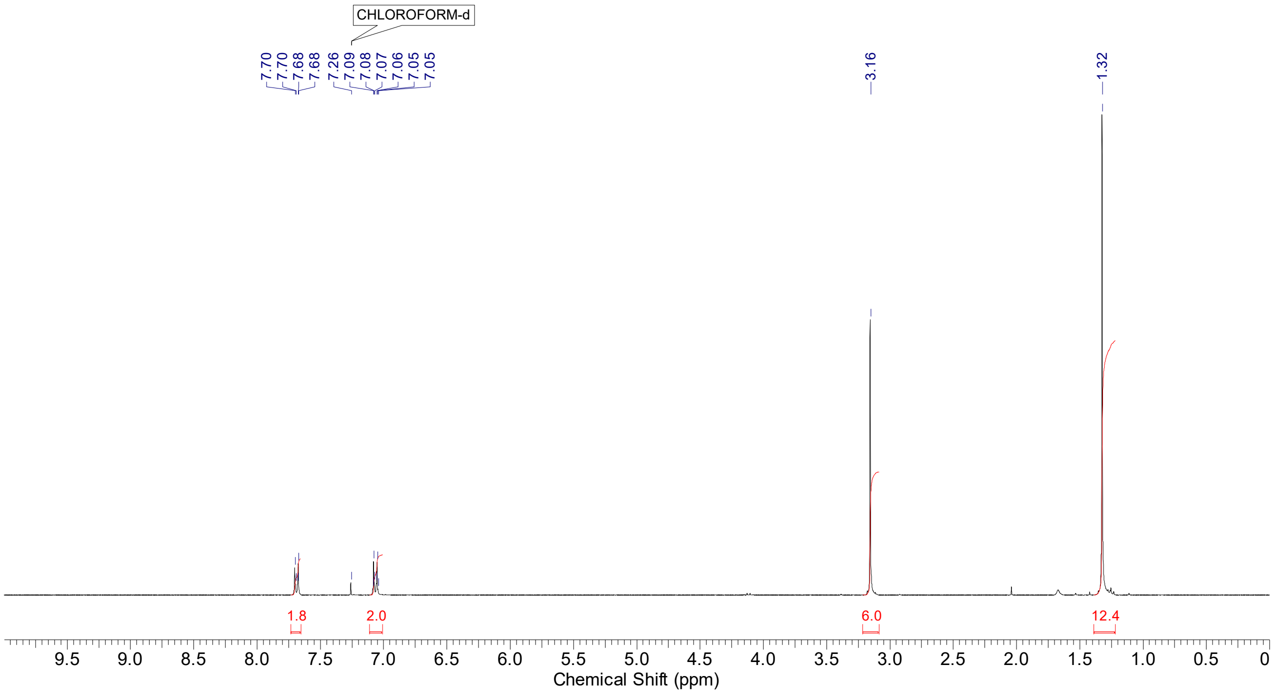

^{13}C NMR (75 MHz, $CDCl_3$)

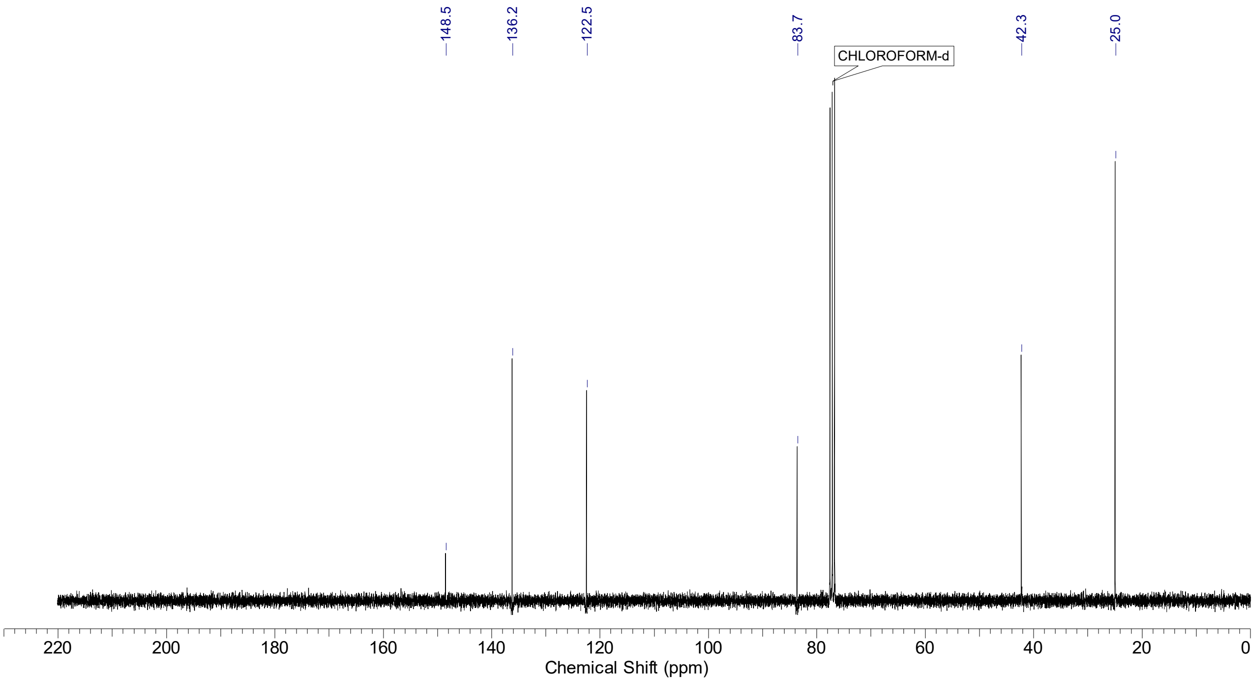

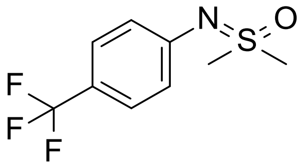

dimethyl-oxo-[4-(trifluoromethyl)phenyl]imino-λ⁶-sulfane [CAS: 2060038-95-9] **(3ha)**

^{1}H NMR (400 MHz, $CDCl_3$)

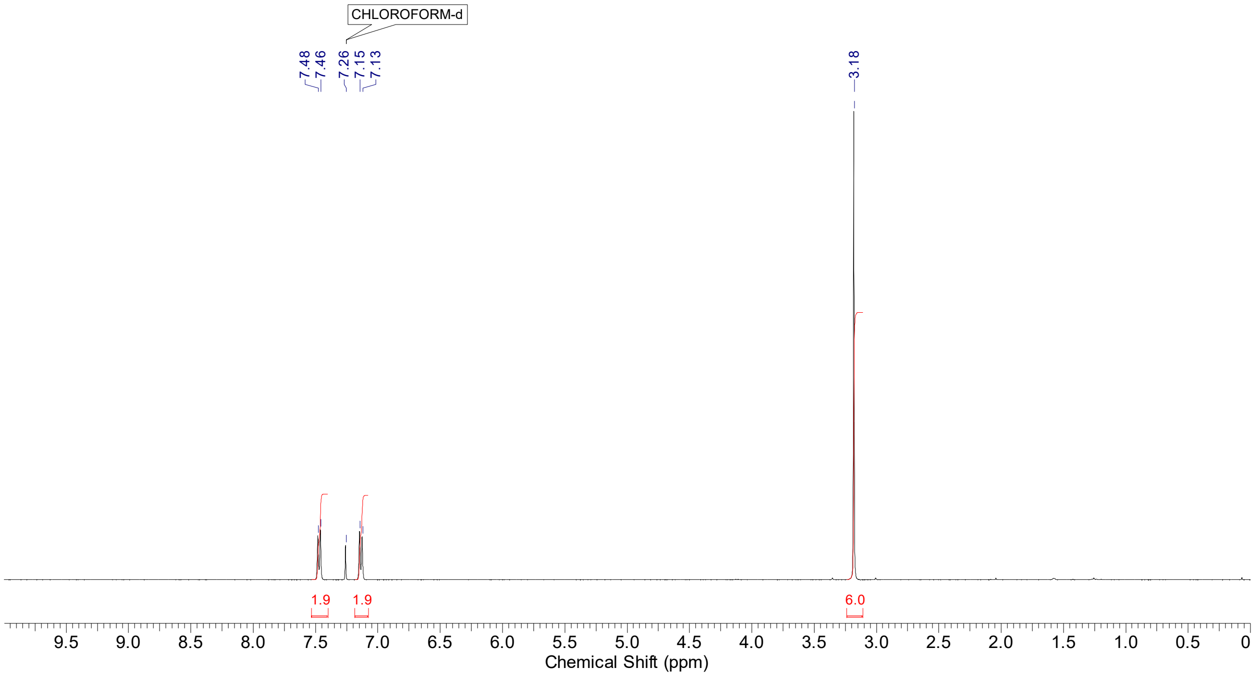

^{13}C NMR (101 MHz, $CDCl_3$)

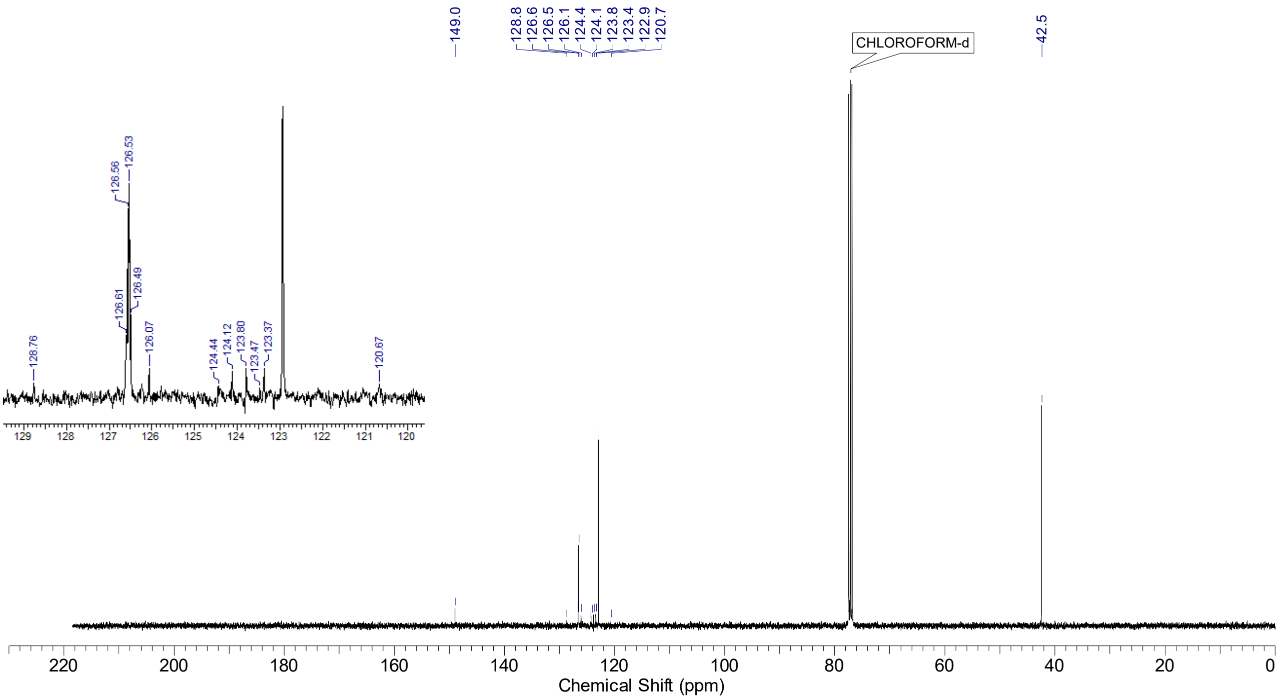

^{19}F NMR (76 MHz, CDCl3, $C_6H_4F_2$)

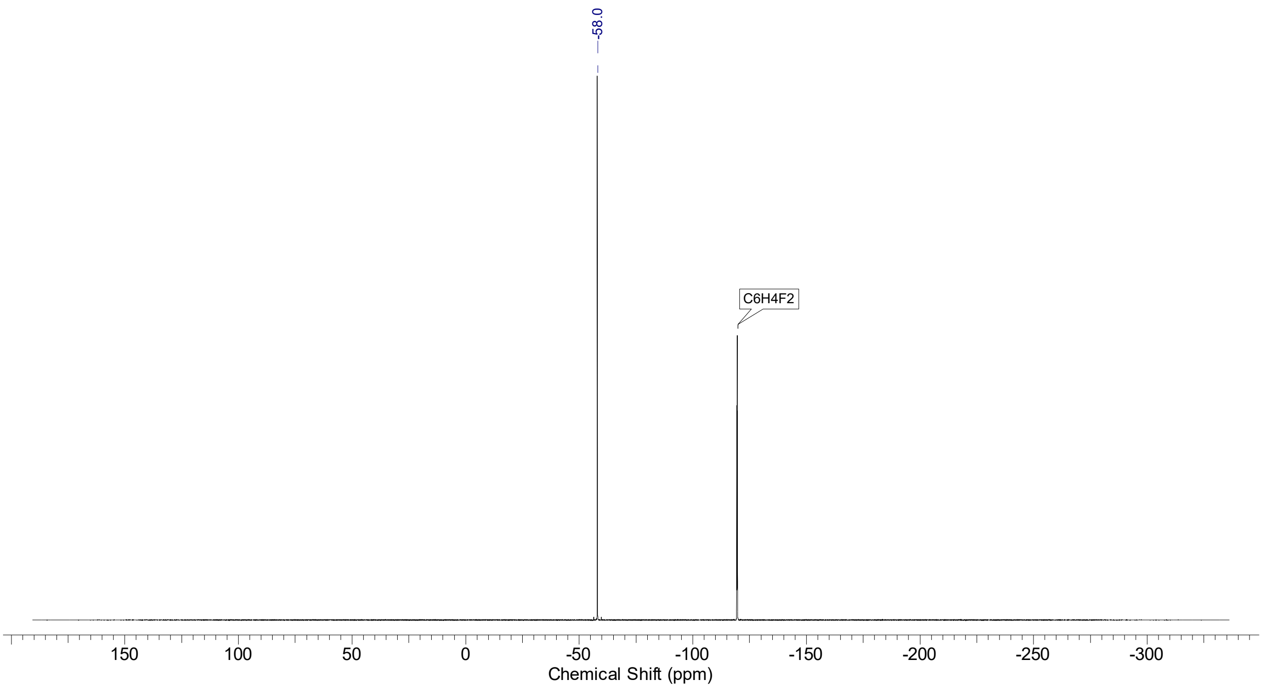

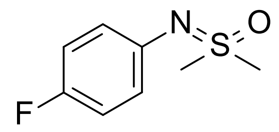

(4-fluorophenyl)imino-dimethyl-oxo-λ⁶-sulfane [CAS: 58873-25-9] **(3ia)**

^{1}H NMR (300 MHz, $CDCl_3$)

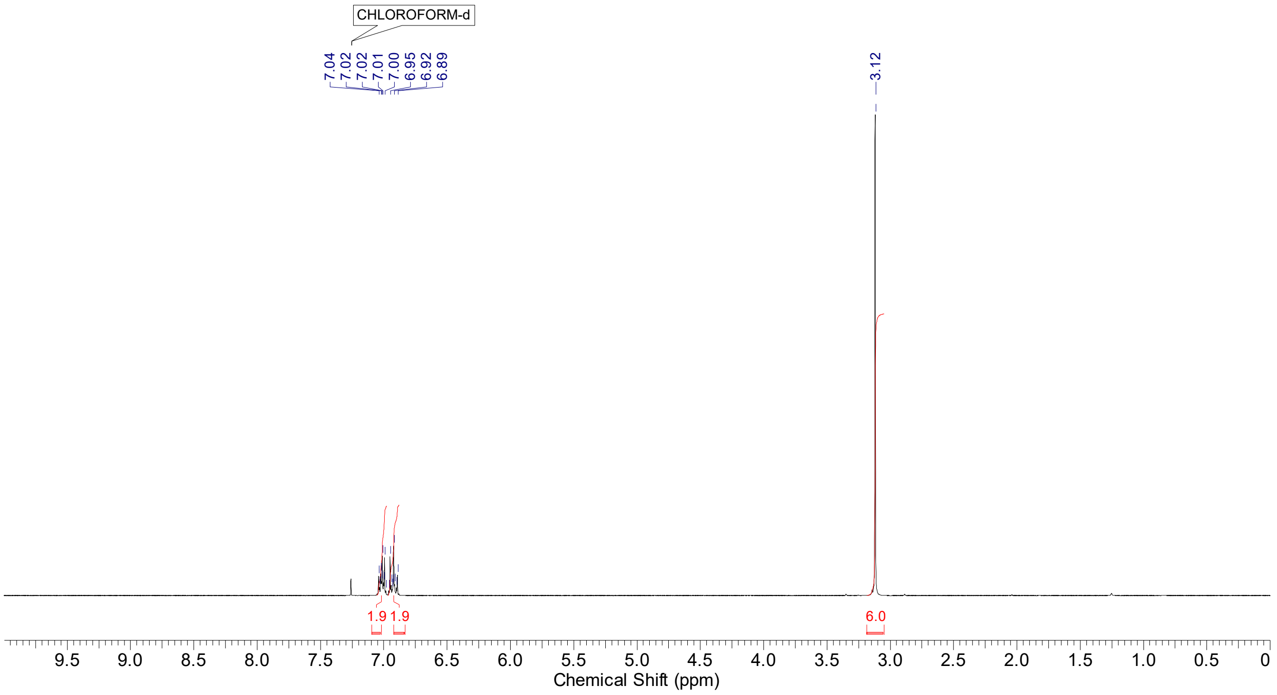

^{13}C NMR (75 MHz, $CDCl_3$)

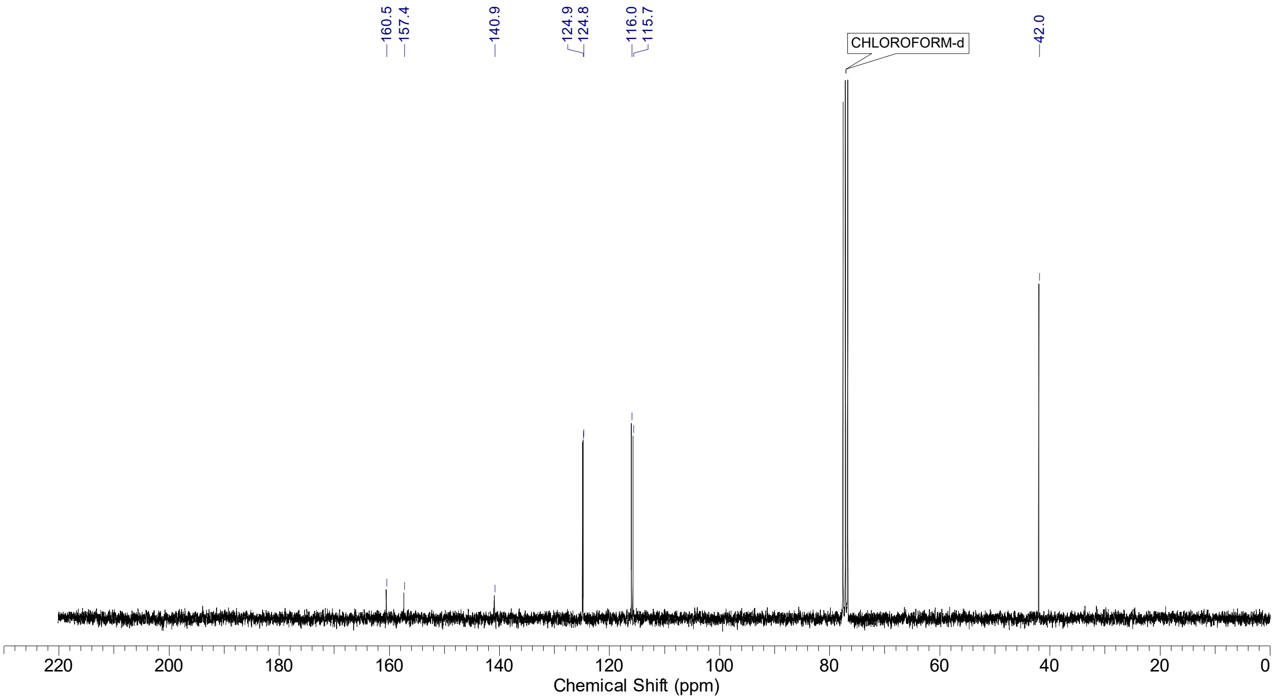

^{19}F NMR (76 MHz, CDCl3, $C_6H_4F_2$)

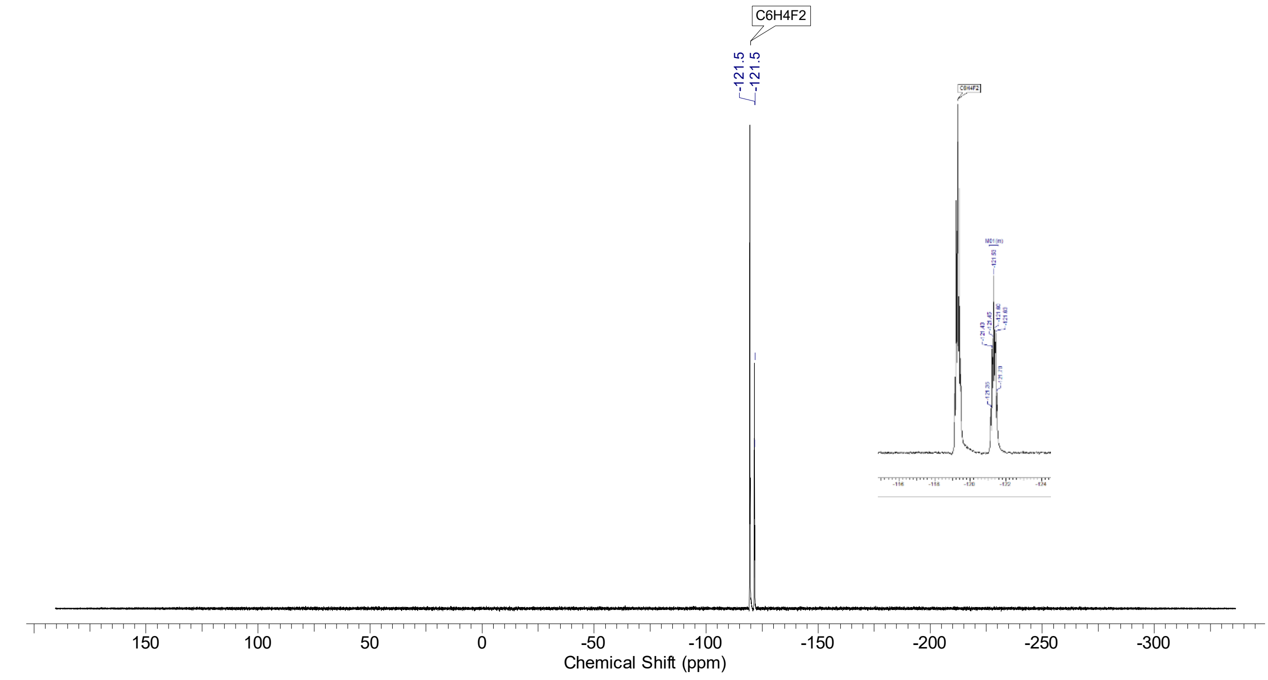

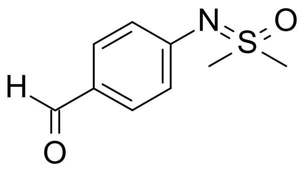

4-[[dimethyl(oxo)-λ⁶-sulfanylidene]amino]benzaldehyde **(3ja)**

^{1}H NMR (400 MHz, $CDCl_3$)

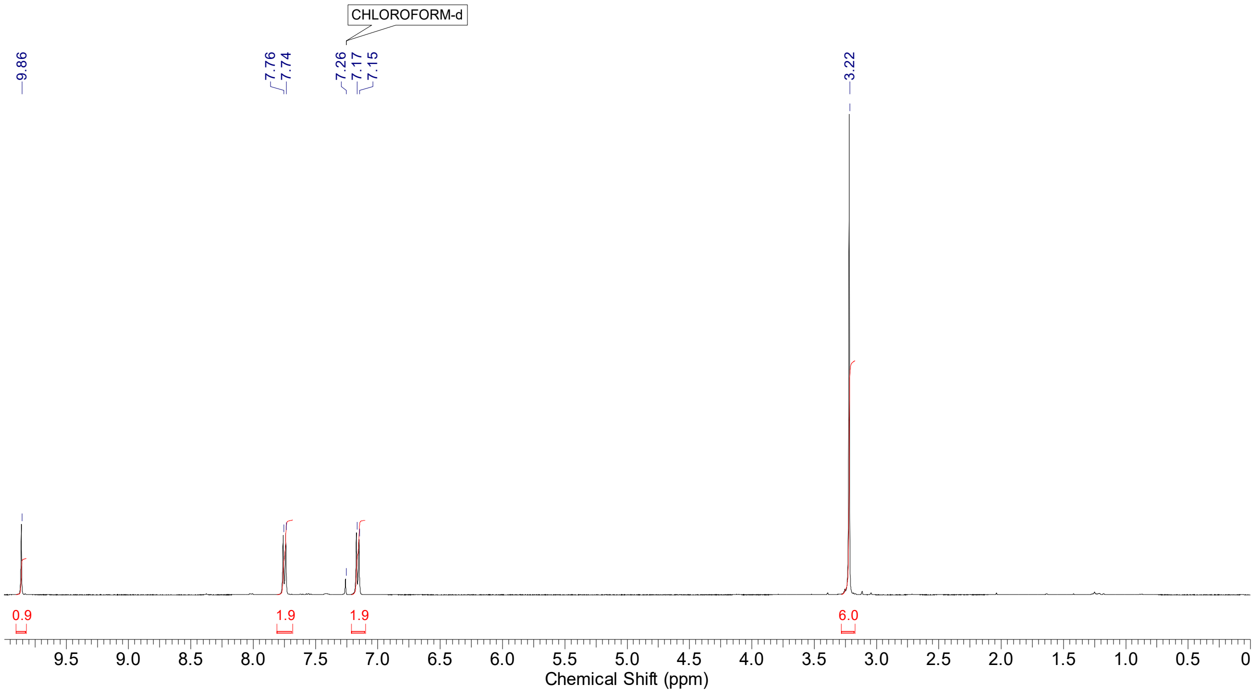

^{13}C NMR (101 MHz, $CDCl_3$)

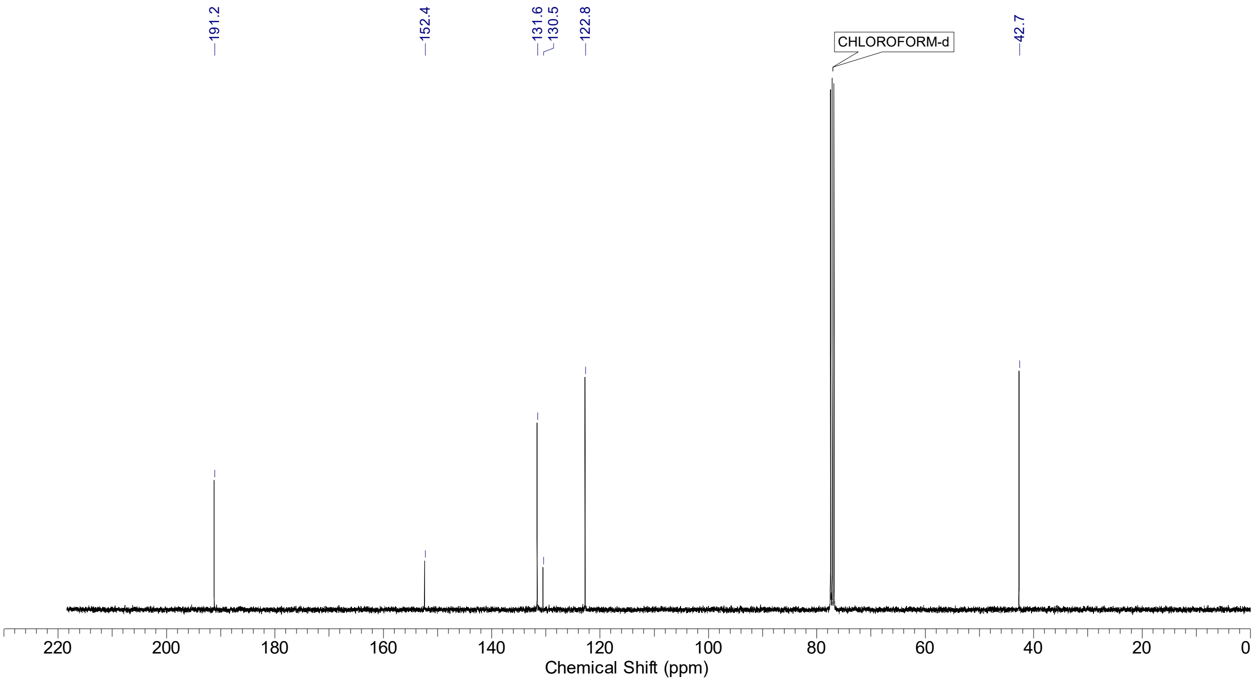

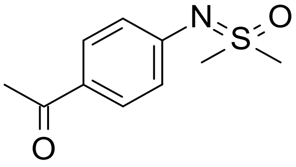

1-[4-[[dimethyl(oxo)-λ⁶-sulfanylidene]amino]phenyl]ethenone **(3ka)**

^{1}H NMR (300 MHz, $CDCl_3$)

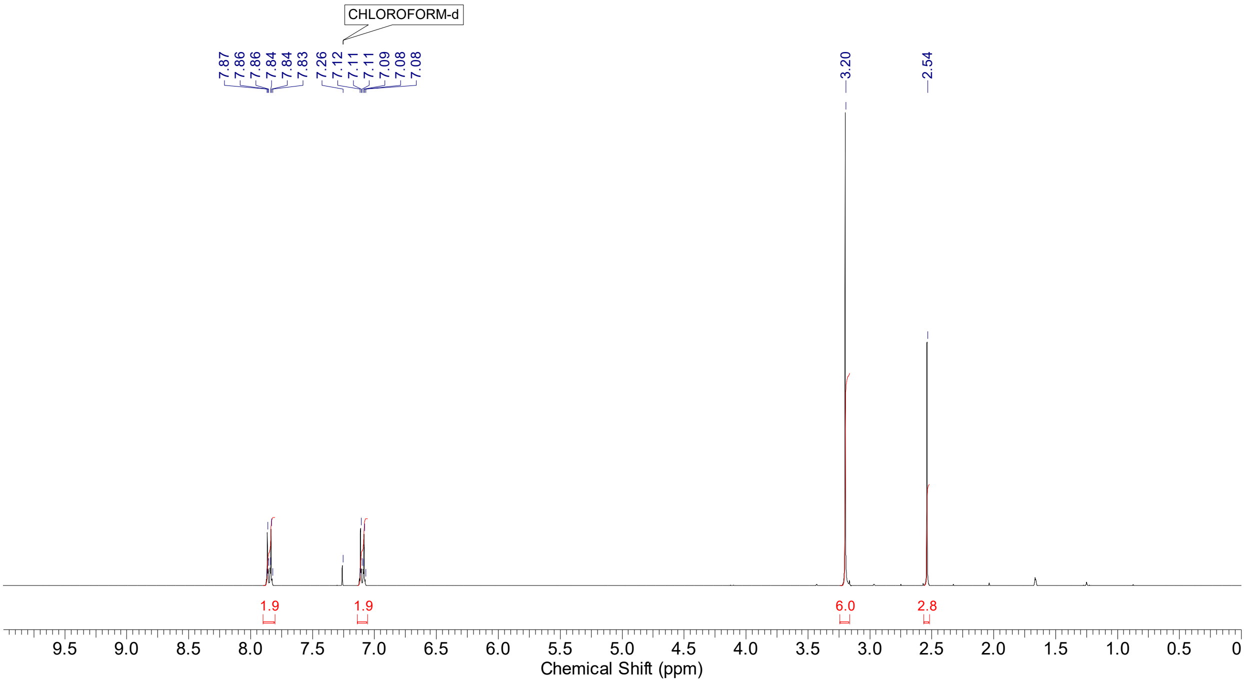

^{13}C NMR (75 MHz, $CDCl_3$)

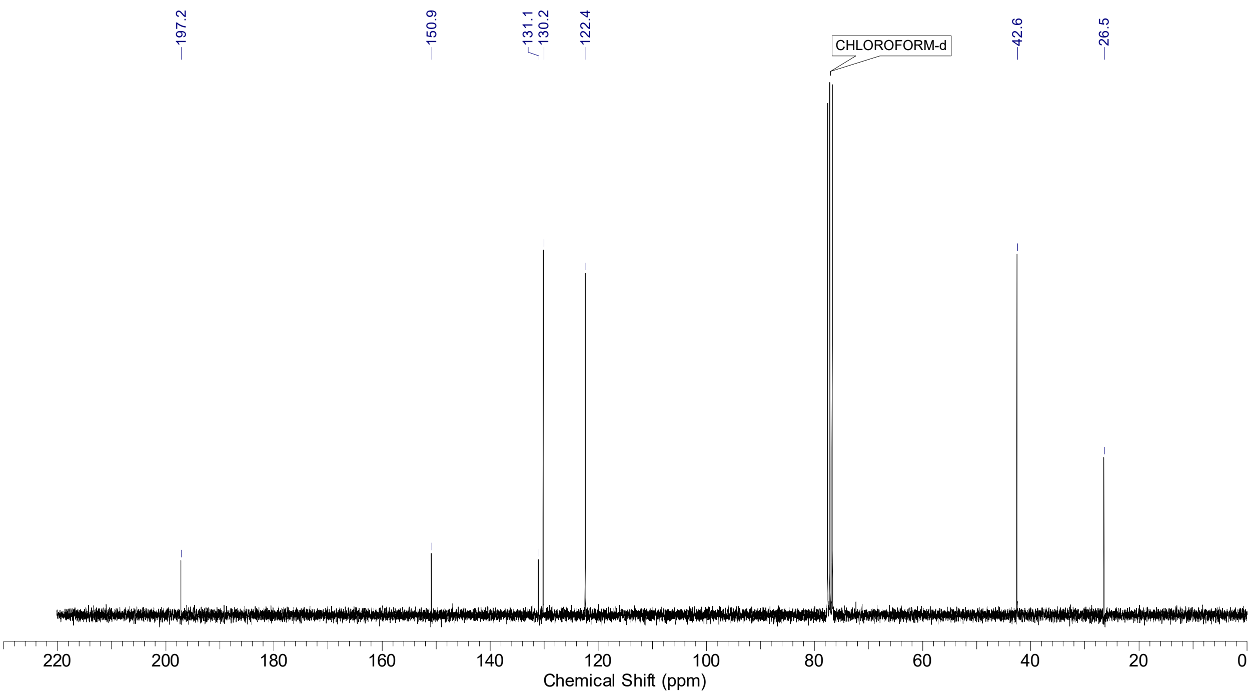

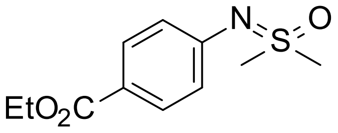

Ethyl 4-[[dimethyl(oxo)-λ⁶-sulfanylidene]amino]benzoate [CAS: 2943218-46-8] **(3la)**

^{1}H NMR (400 MHz, $CDCl_3$)

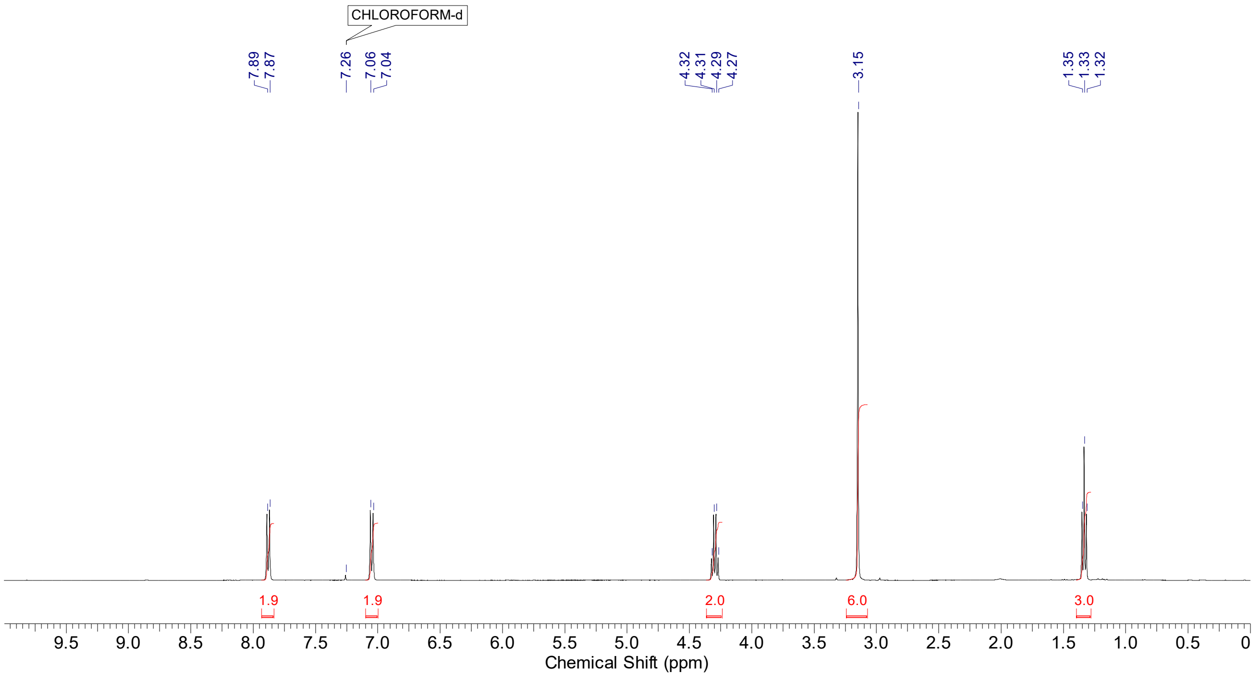

^{13}C NMR (101 MHz, $CDCl_3$)

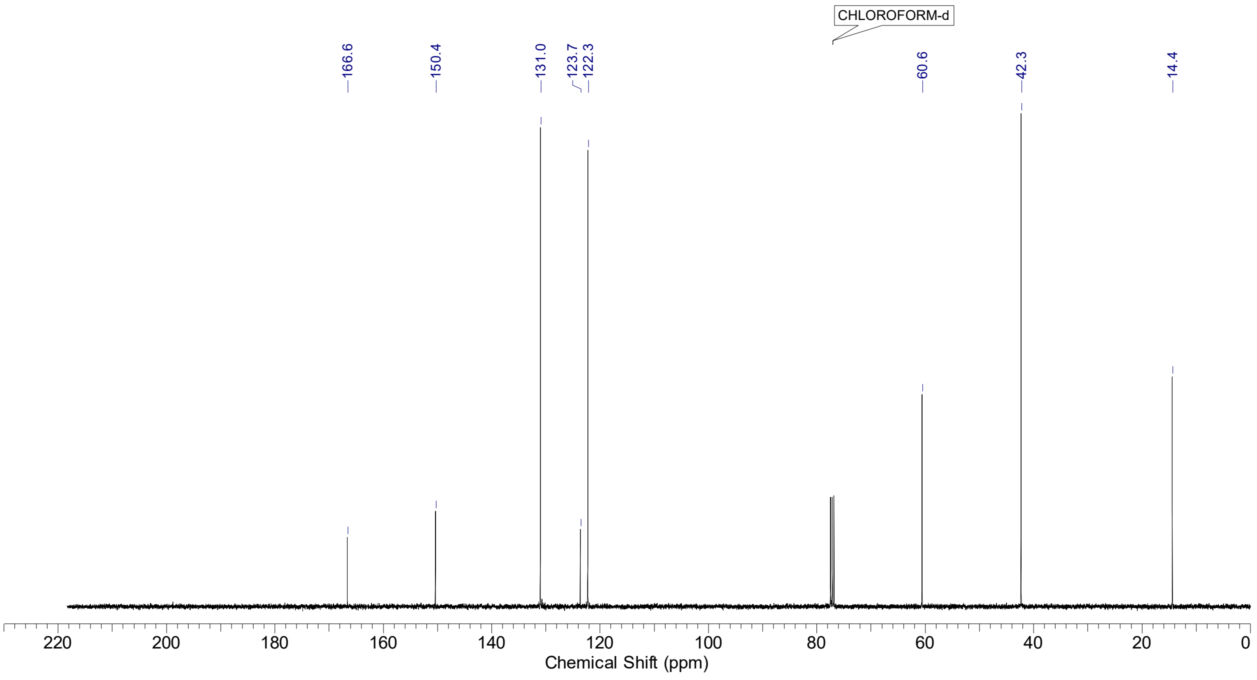

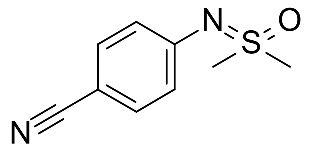

4-[[dimethyl(oxo)-λ^6-sulfanylidene]amino]benzonitrile [CAS: 56158-12-4] **(3ma)**

^{1}H NMR (400 MHz, $CDCl_3$)

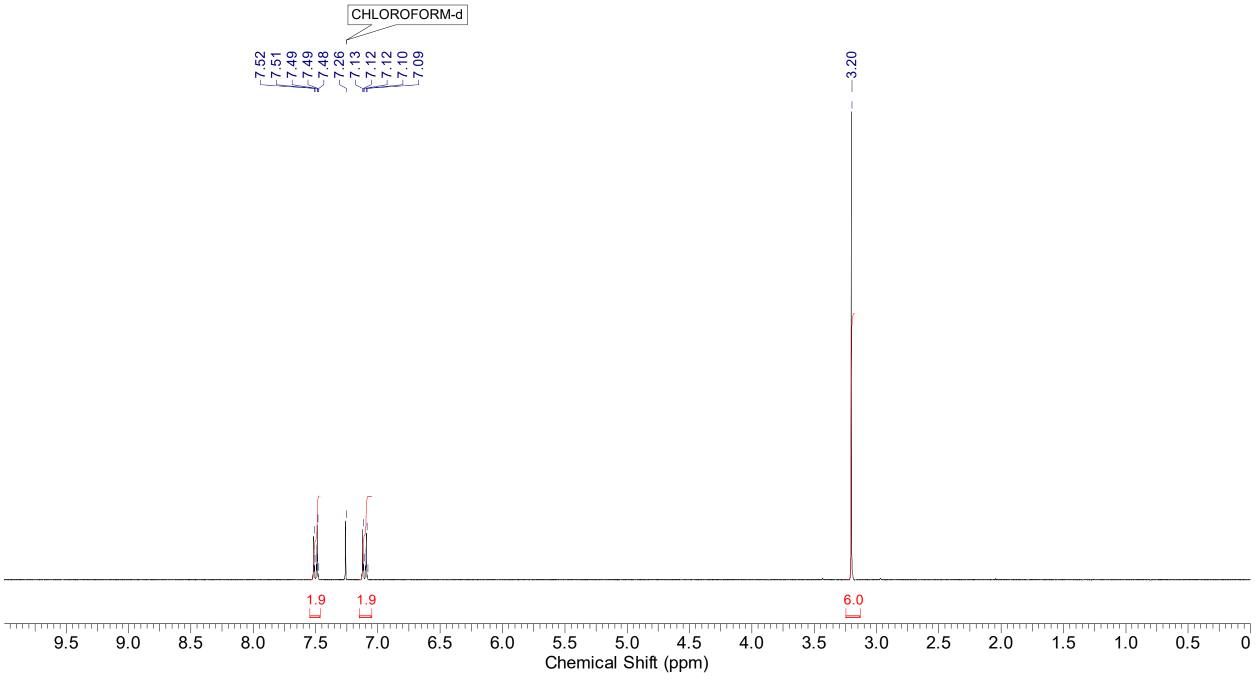

^{13}C NMR (101 MHz, $CDCl_3$)

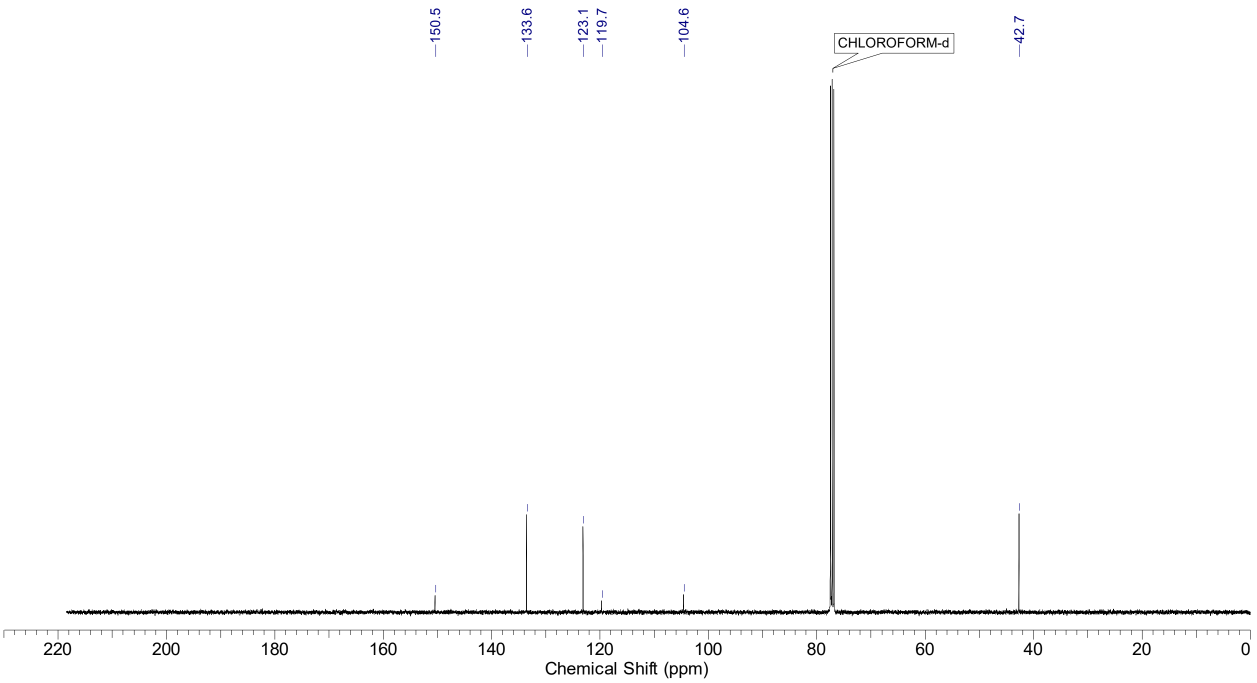

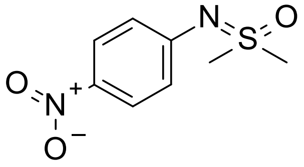

Dimethyl-(4-nitrophenyl)imino-oxo-λ⁶-sulfane [CAS: 56158-00-0] **(3na)**

^{1}H NMR (400 MHz, $CDCl_3$)

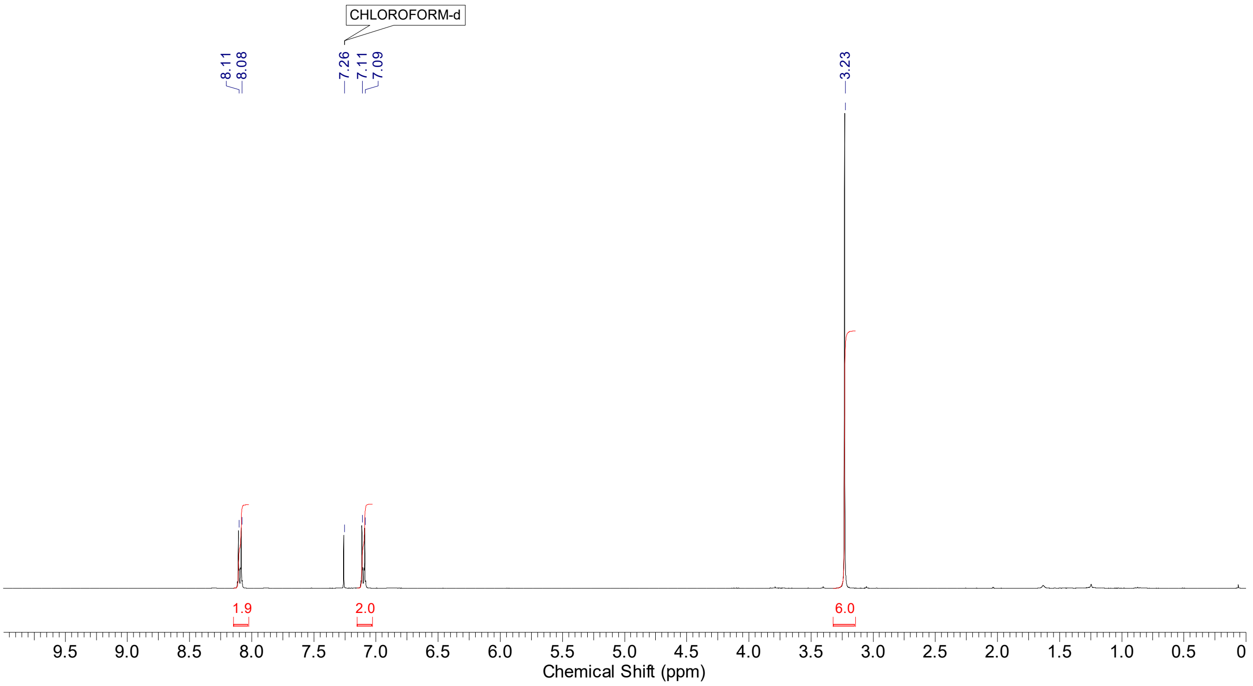

^{13}C NMR (101 MHz, $CDCl_3$)

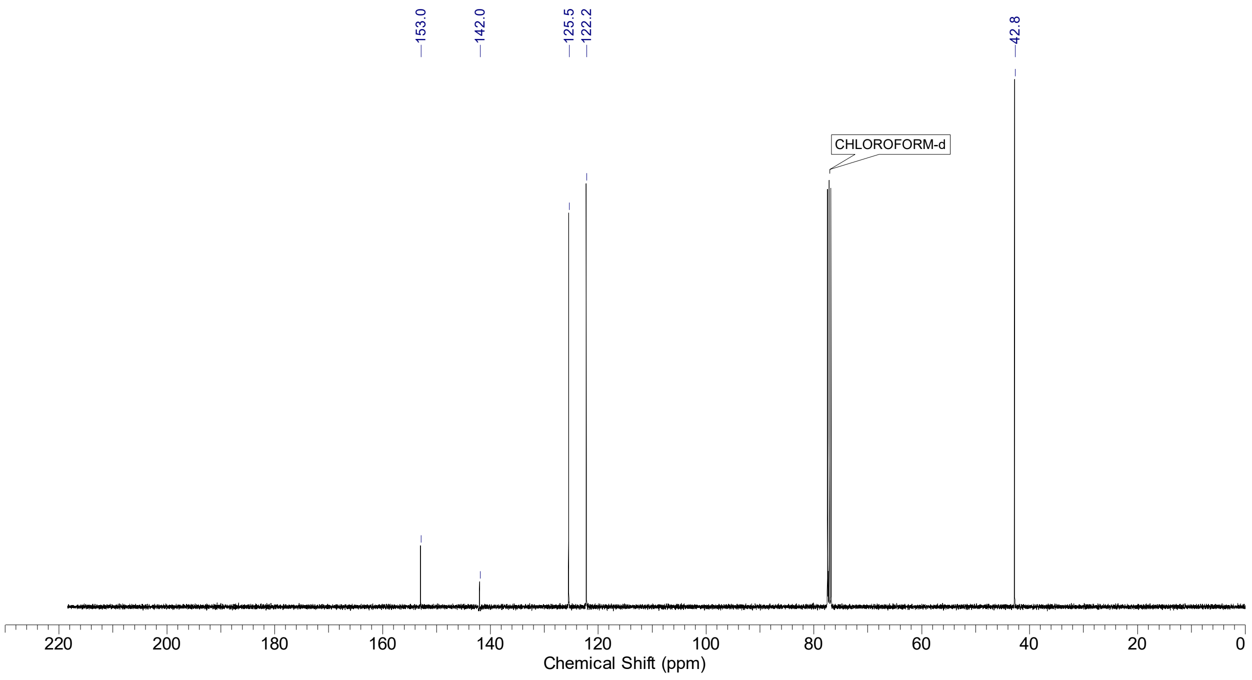

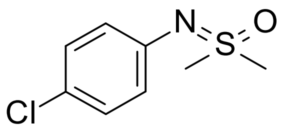

(4-chlorophenyl)imino-dimethyl-oxo-λ⁶-sulfane **(3oa)**

^{1}H NMR (400 MHz, $CDCl_3$)

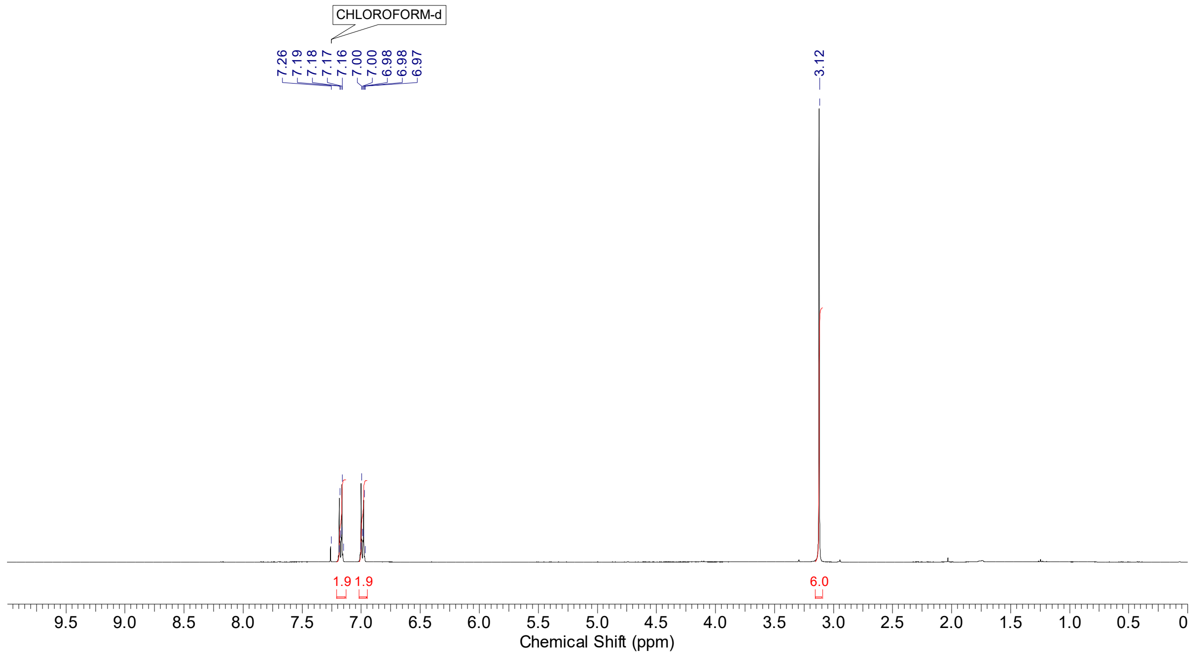

^{13}C NMR (101 MHz, $CDCl_3$)

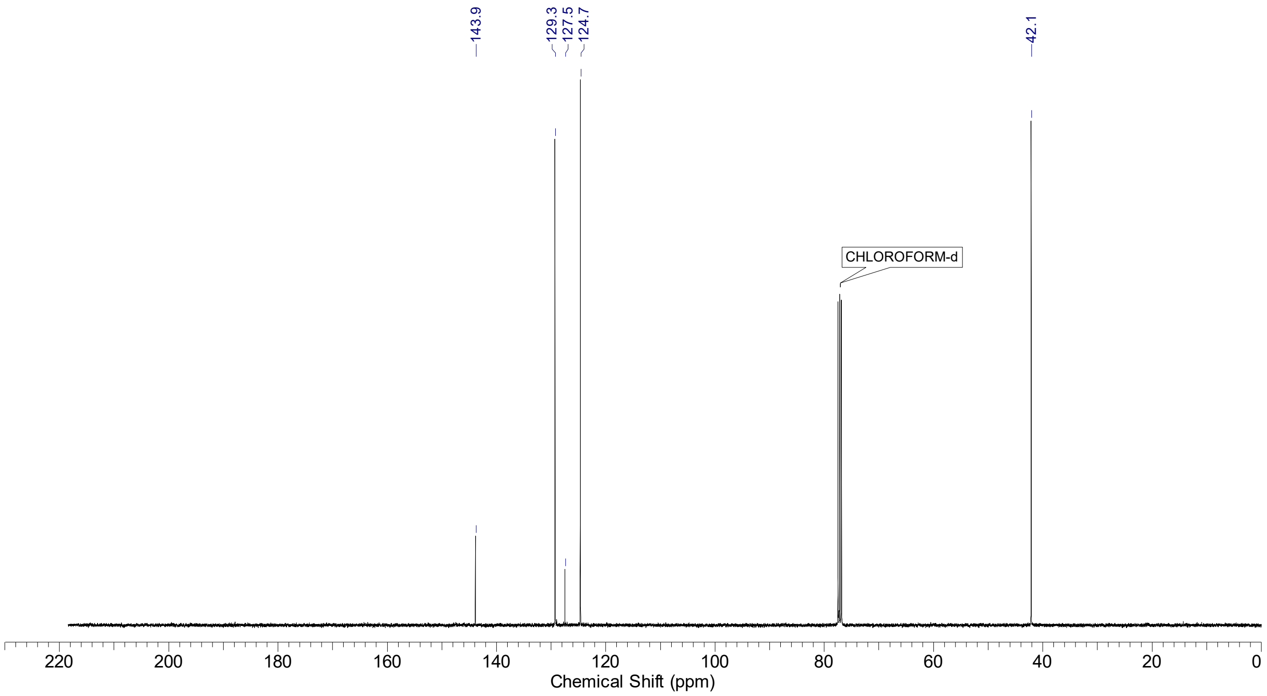

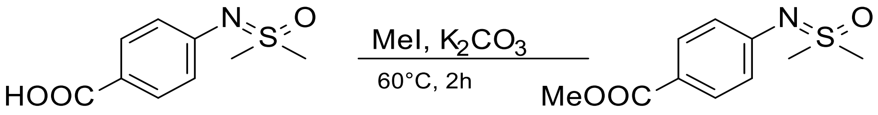

4-[[dimethyl(oxo)-λ⁶-sulfanylidene]amino]benzoic acid **(3pa)**

^{1}H NMR (300 MHz, $CDCl_3$)

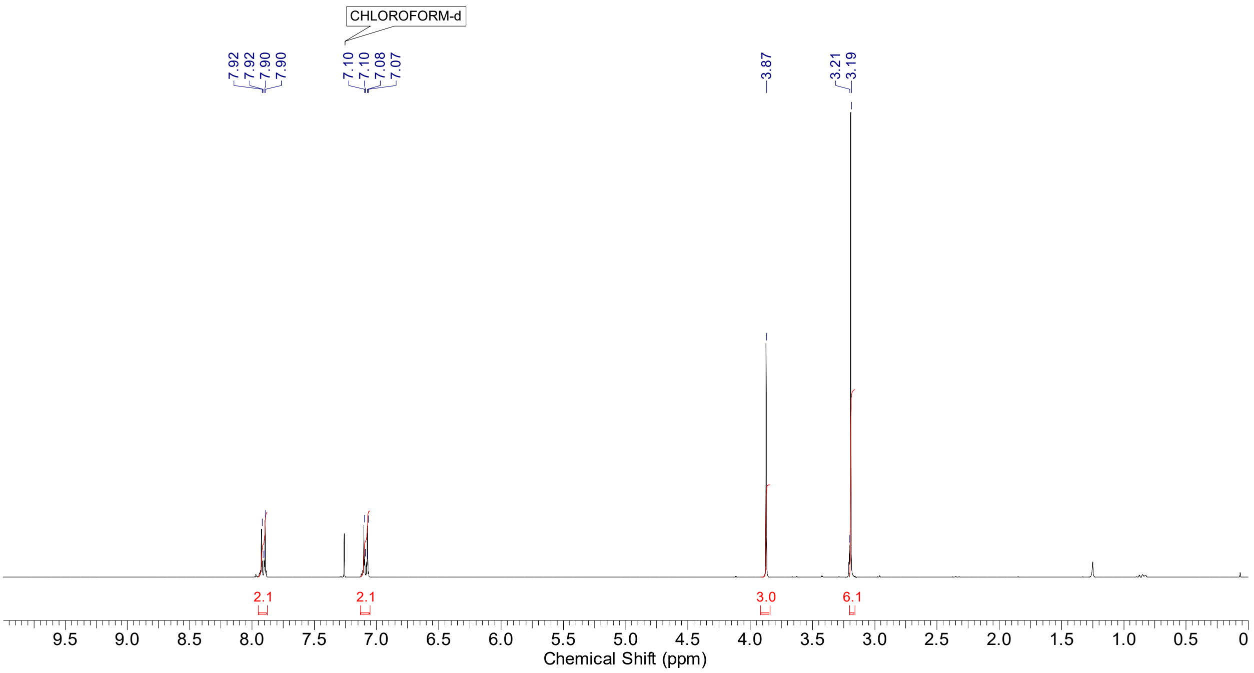

^{13}C NMR (75 MHz, $CDCl_3$)

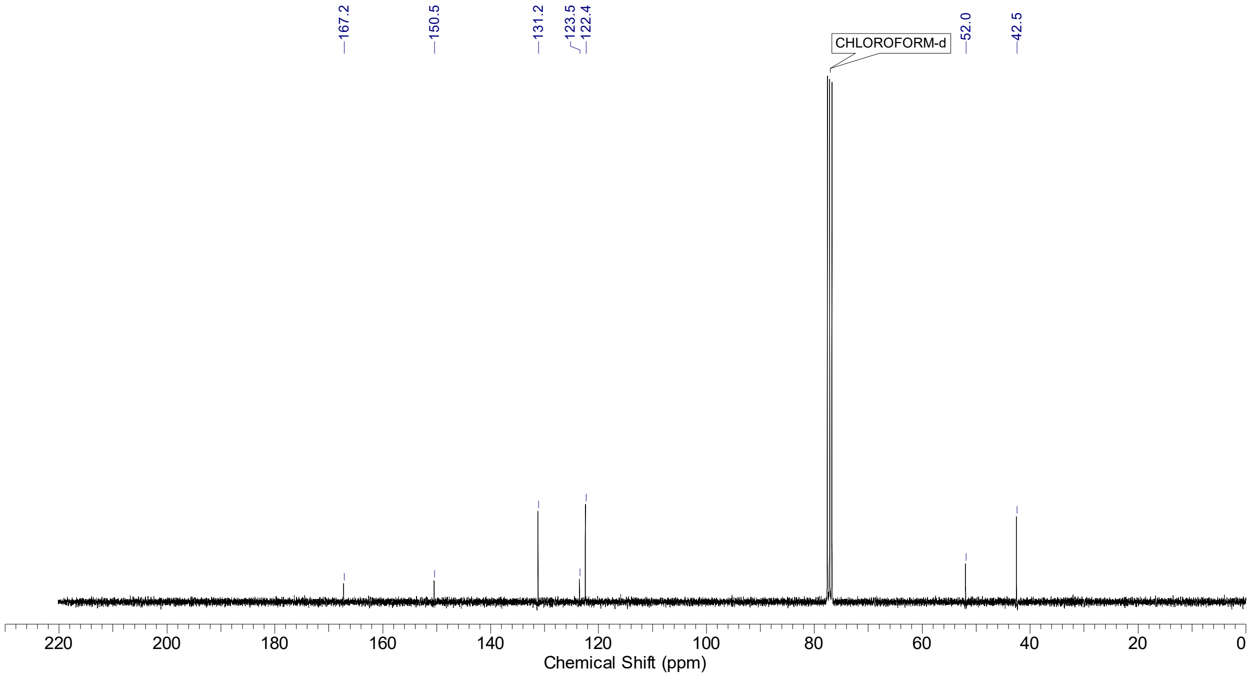

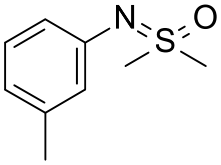

dimethyl-(m-tolylimino)-oxo-λ⁶-sulfane **(3qa)**

^{1}H NMR (400 MHz, $CDCl_3$)

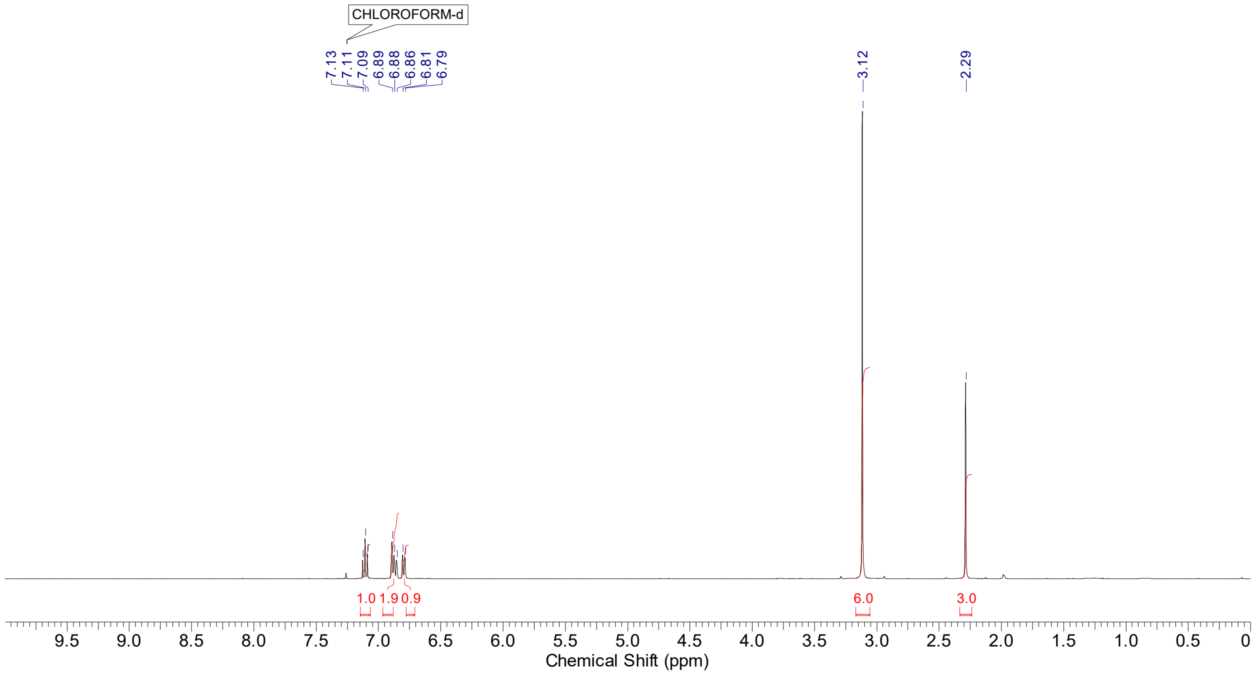

^{13}C NMR (101 MHz, $CDCl_3$)

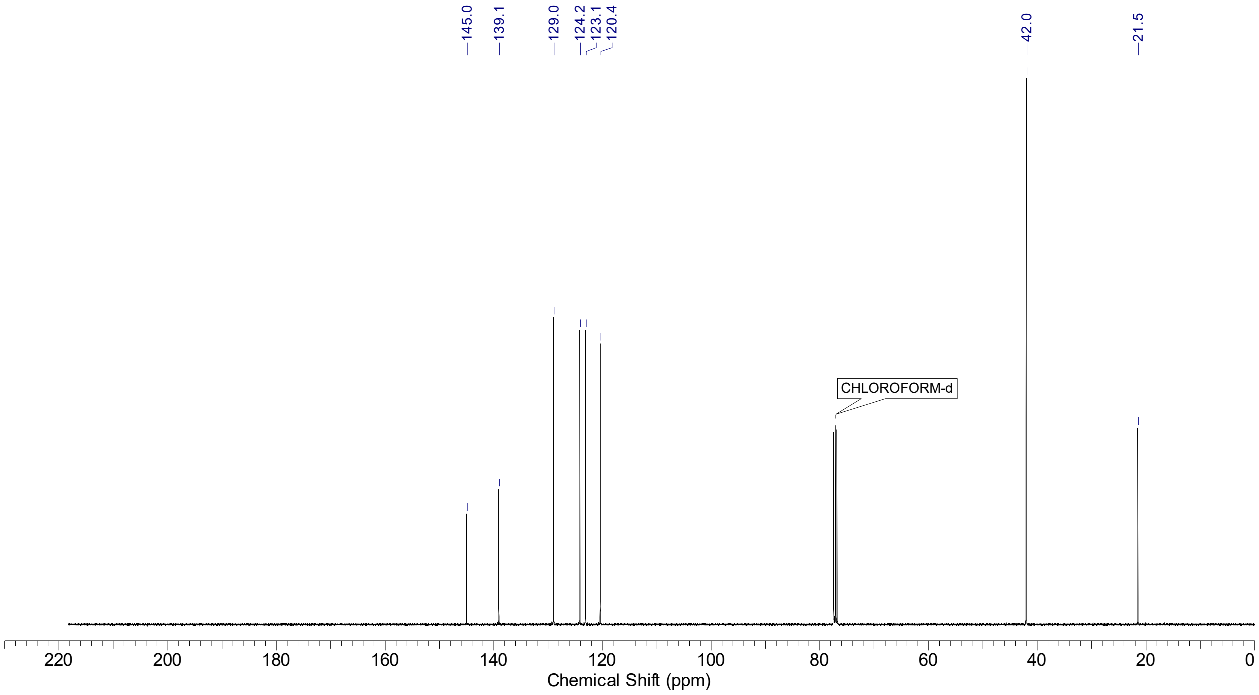

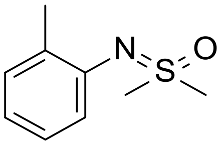

Dimethyl-(o-tolylimino)-oxo-λ^6-sulfane [CAS: 2060024-93-1] **(3ra)**

^1H NMR (400 MHz, $CDCl_3$)

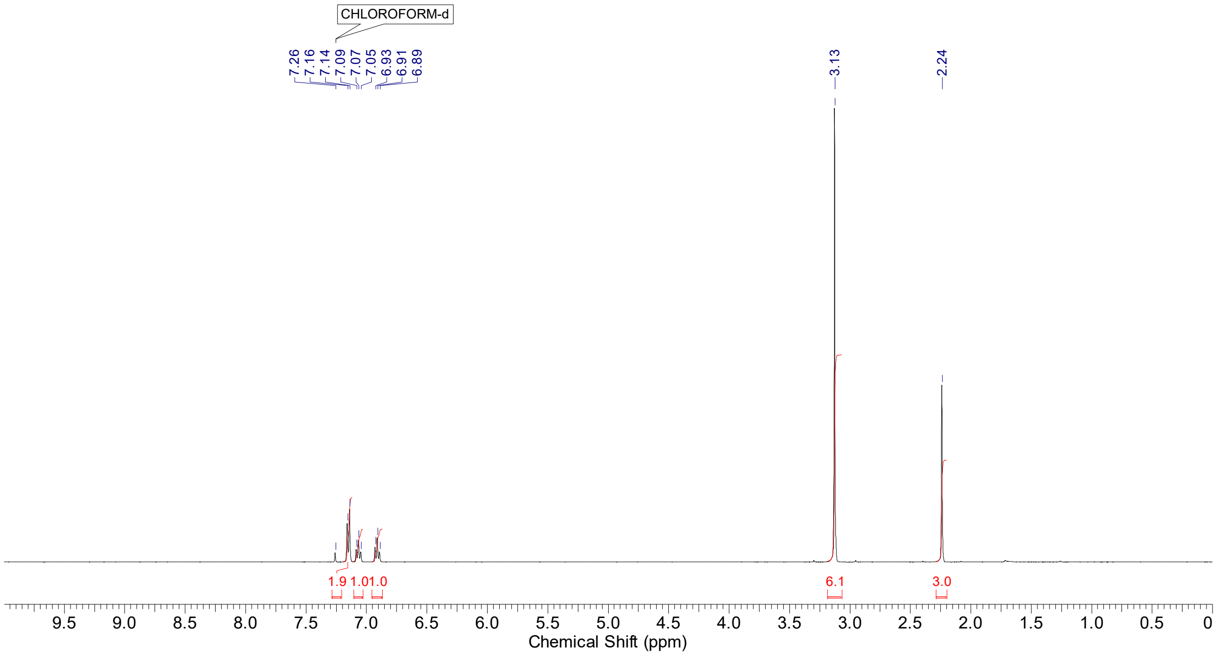

^{13}C NMR (101 MHz, $CDCl_3$)

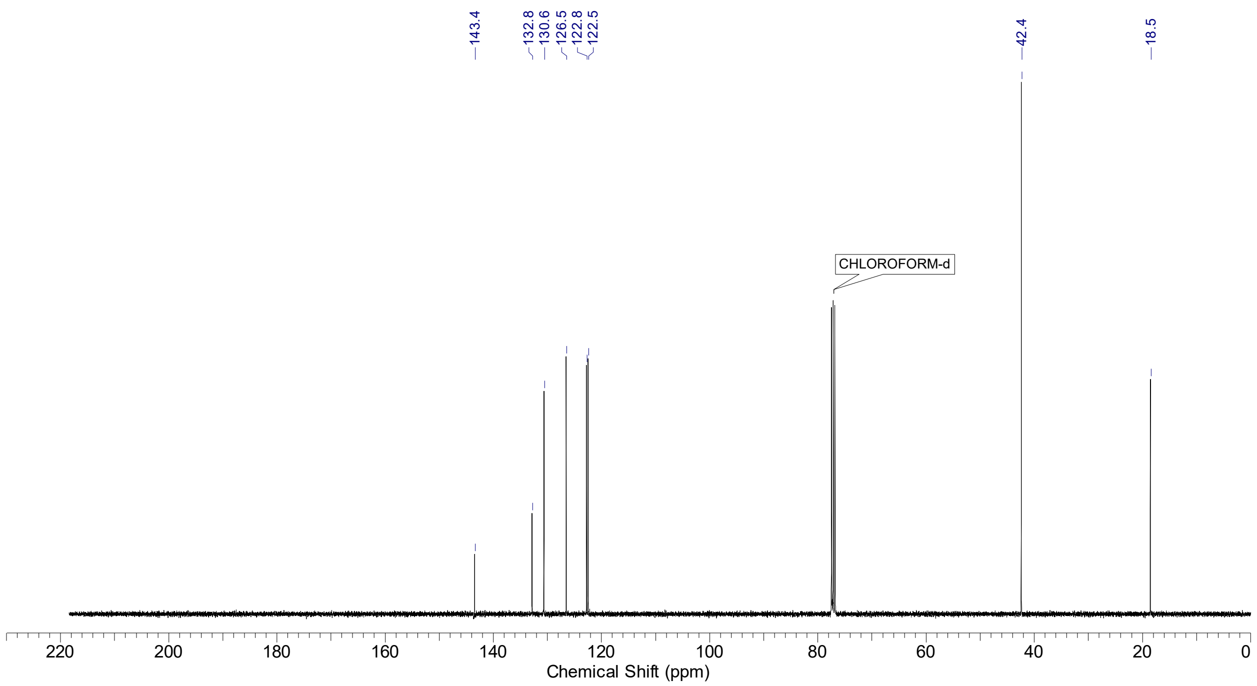

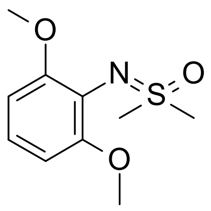

(2,6-dimethoxyphenyl)imino-dimethyl-oxo-λ⁶-sulfane **(3sa)**

^{1}H NMR (400 MHz, $CDCl_3$)

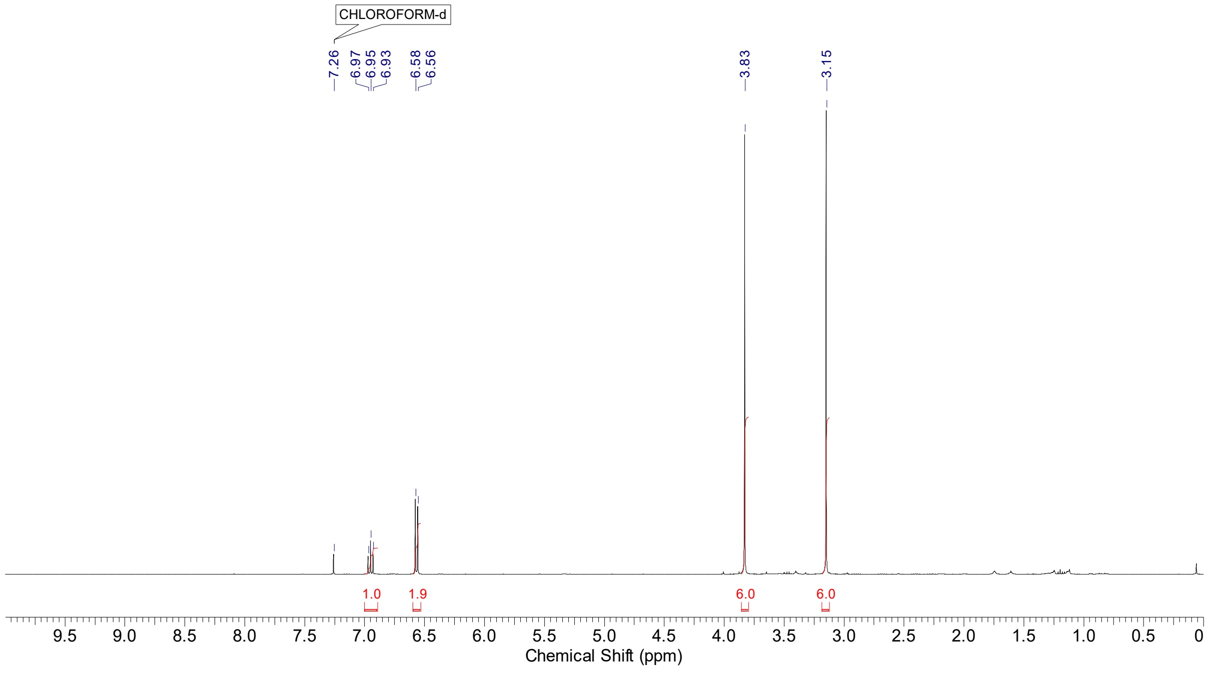

^{13}C NMR (101 MHz, $CDCl_3$) (*ethyl acetate impurity)

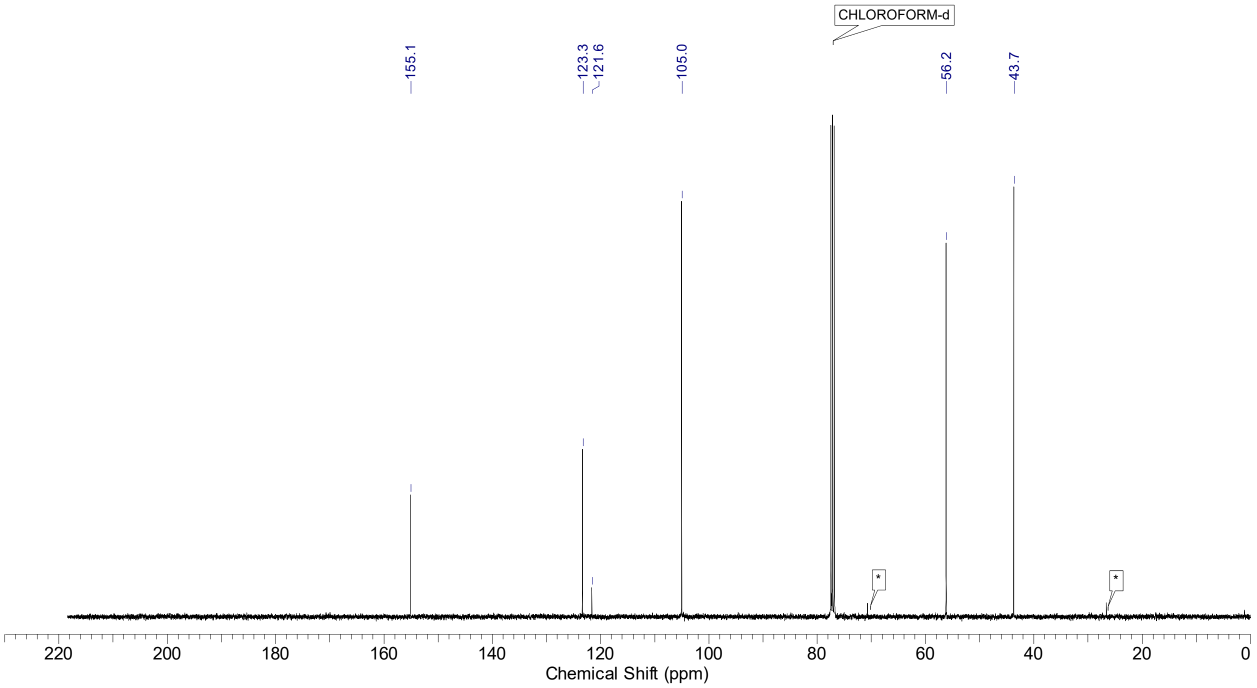

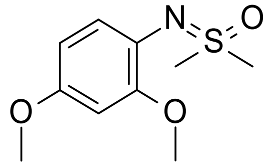

(2,4-dimethoxyphenyl)imino-dimethyl-oxo-λ⁶-sulfane **(3ta)**

^{1}H NMR (400 MHz, $CDCl_3$)

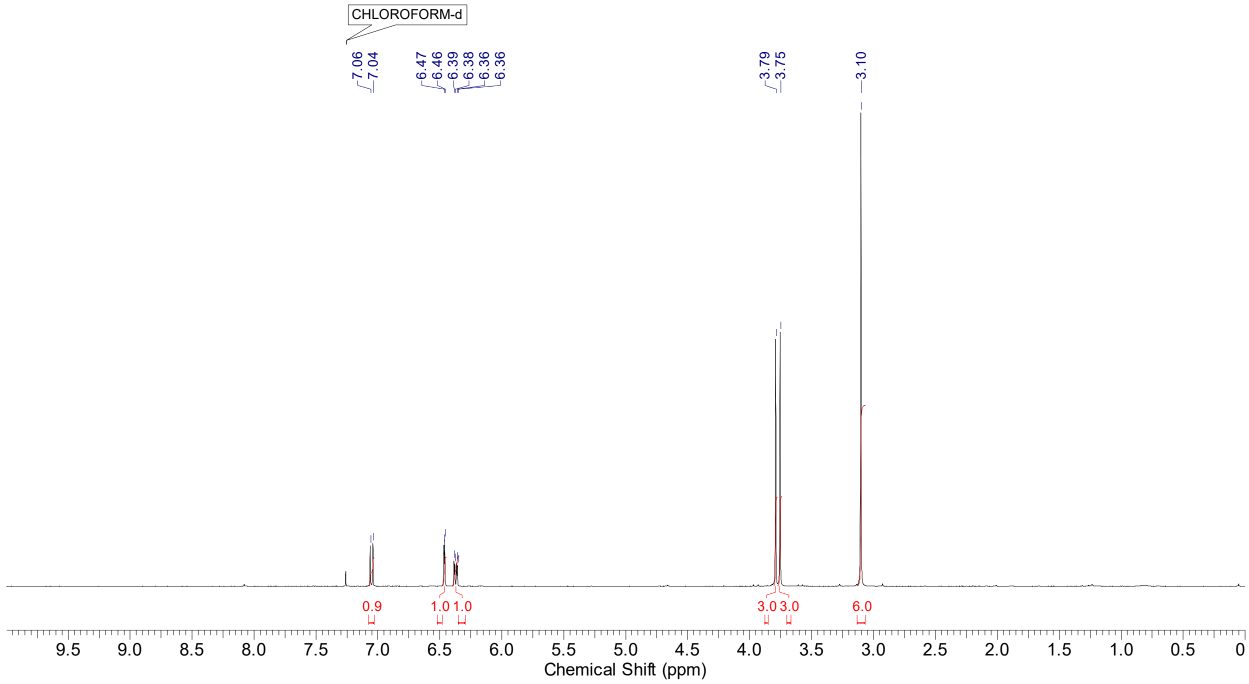

^{13}C NMR (101 MHz, $CDCl_3$)

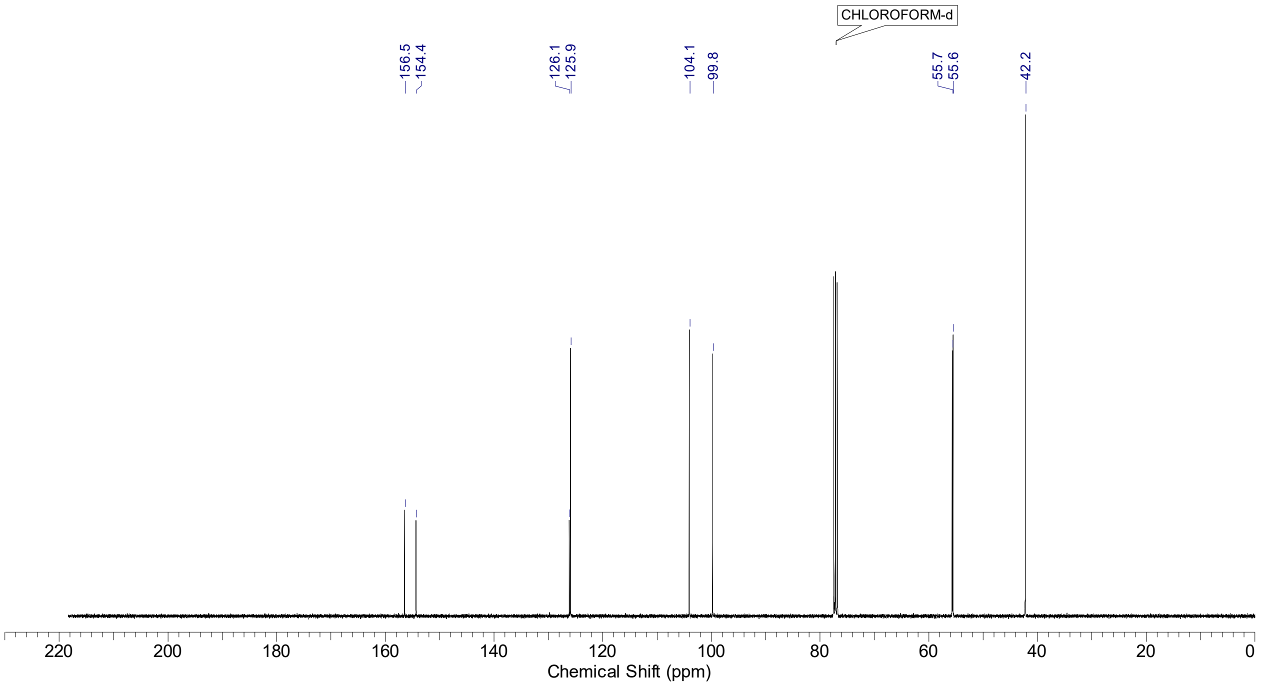

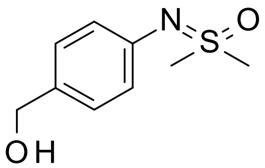

[4-[[dimethyl(oxo)-λ⁶-sulfanylidene]amino]phenyl] methanol **(3ua)**

^{1}H NMR (400 MHz, $CDCl_3$)

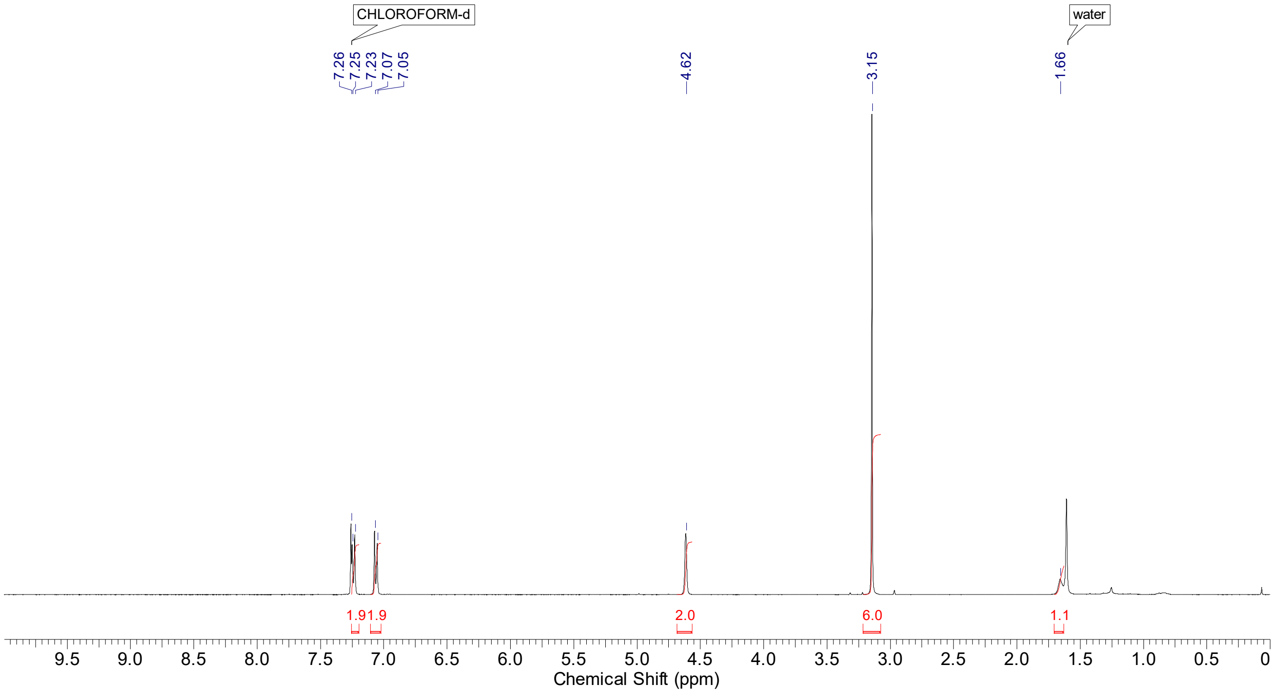

^{13}C NMR (101 MHz, $CDCl_3$)

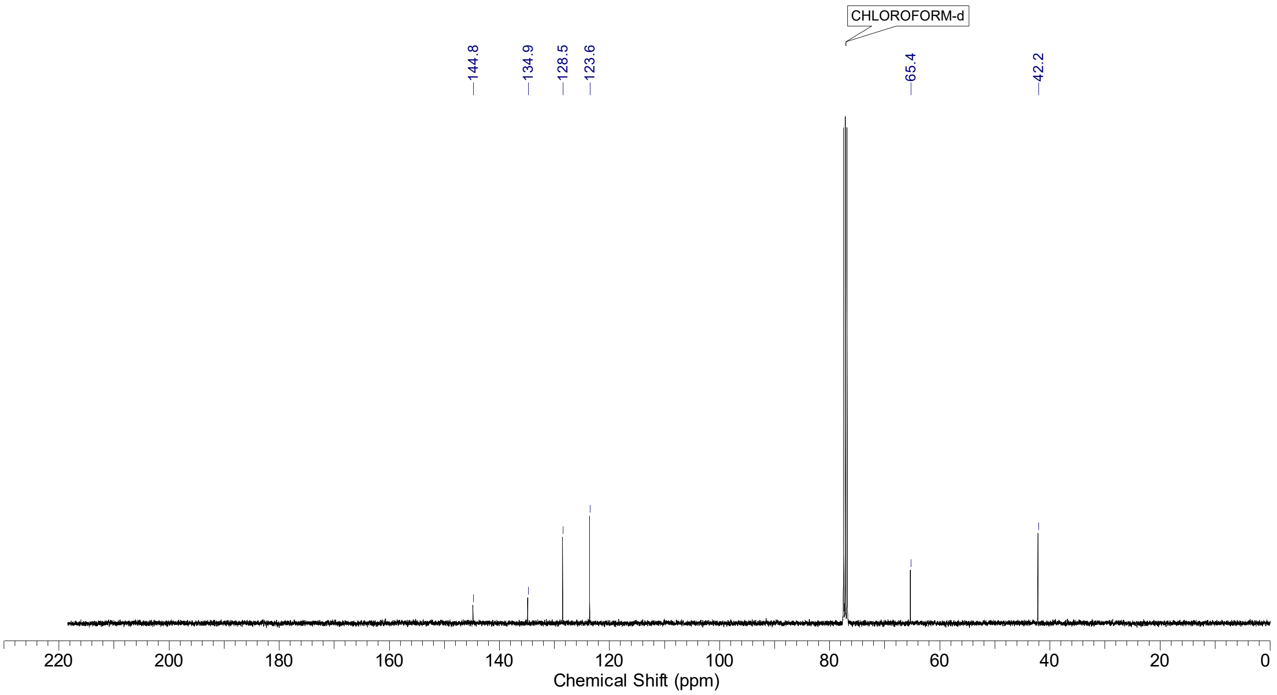

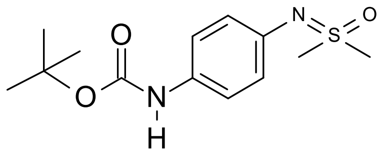

tert-butyl N-[4-[[dimethyl(oxo)-λ⁶-sulfanylidene]amino]phenyl]carbamate **(3va)**

^{1}H NMR (300 MHz, $CDCl_3$)

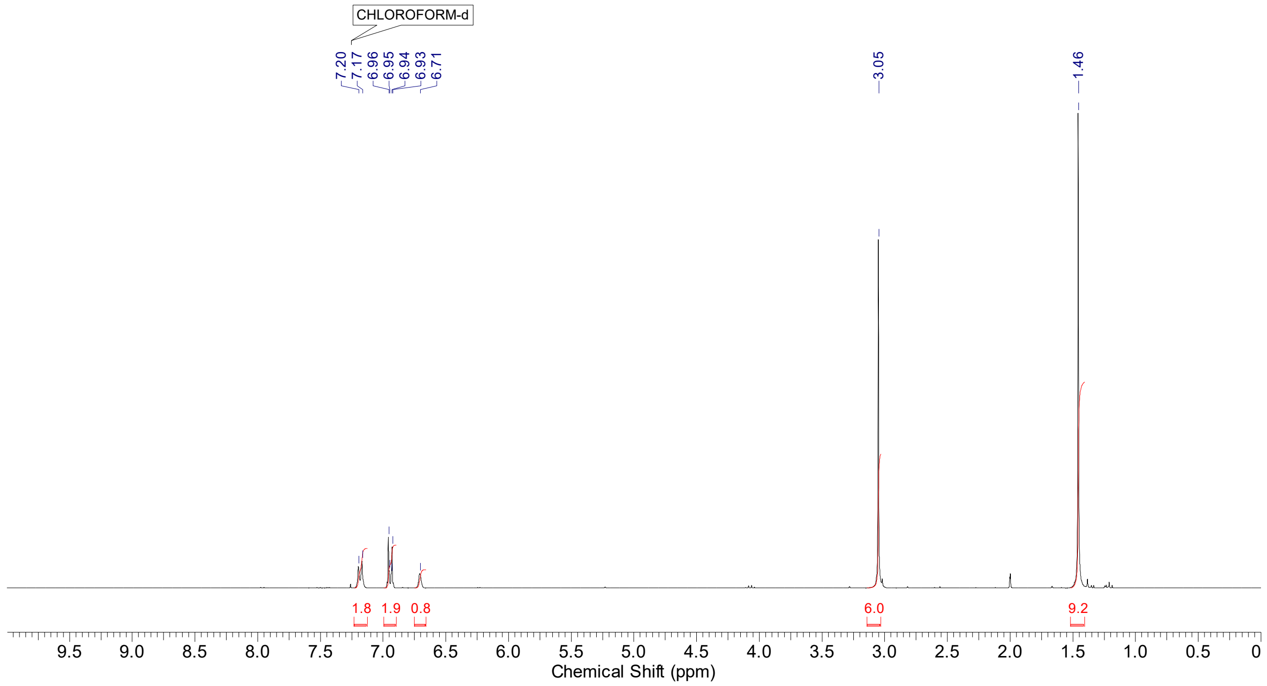

^{13}C NMR (75 MHz, $CDCl_3$)

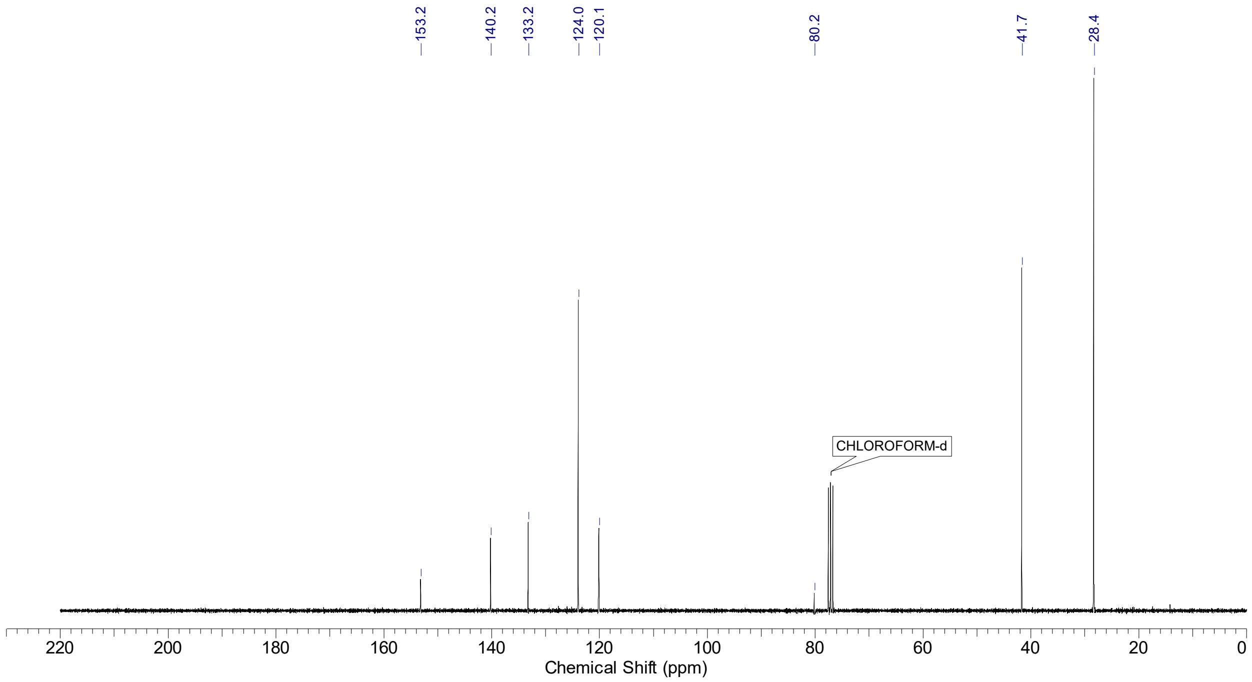

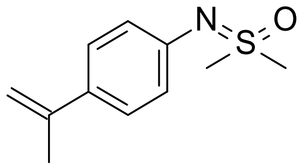

(4-isopropenylphenyl)imino-dimethyl-oxo-λ^6-sulfane **(3wa)**

^{1}H NMR (300 MHz, $CDCl_3$)

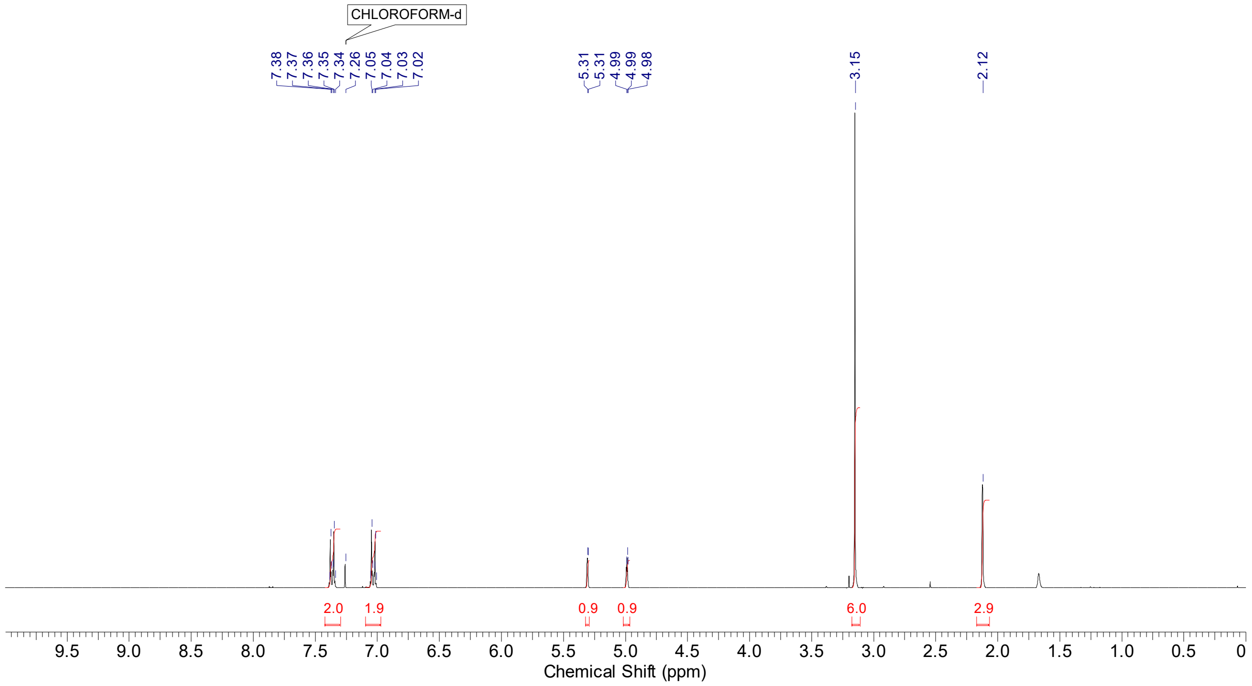

^{13}C NMR (75 MHz, $CDCl_3$)

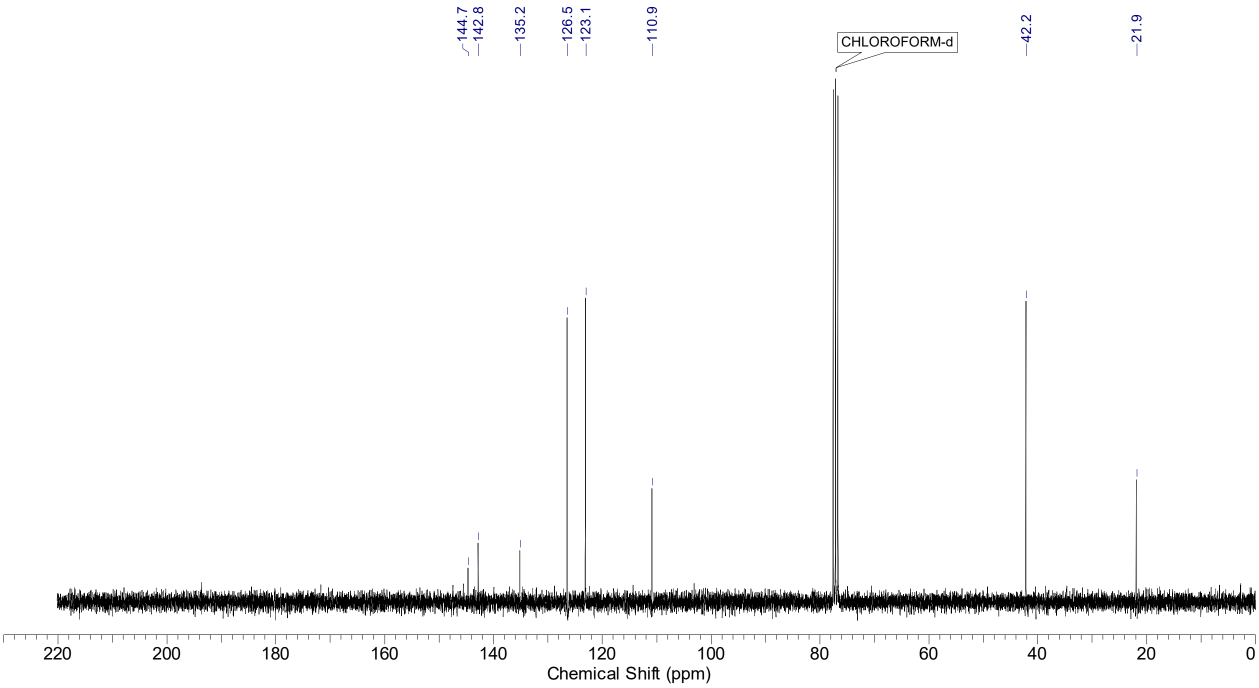

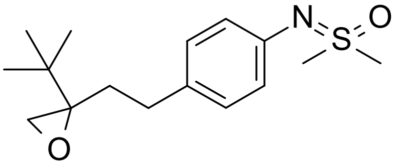

[4-[2-(2-tert-butyloxiran-2-yl)ethyl]phenyl]imino-dimethyl-oxo-λ⁶-sulfane **(3xa)**

^{1}H NMR (300 MHz, $CDCl_3$)

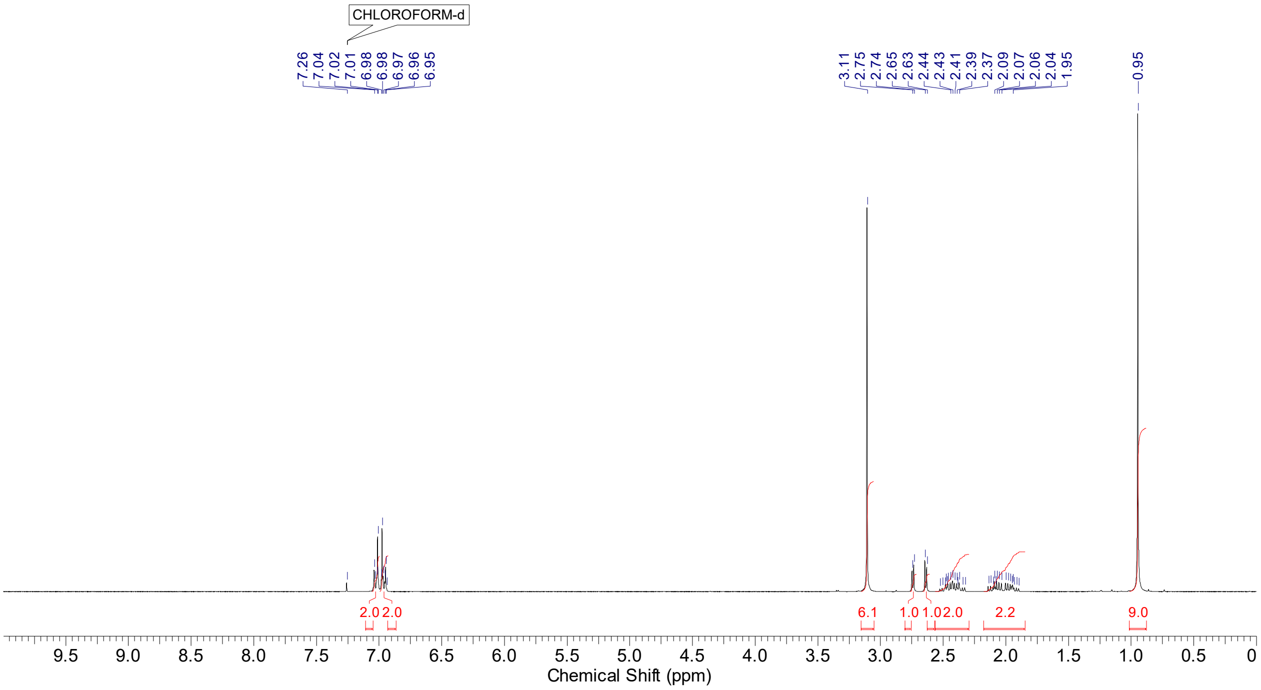

^{13}C NMR (75 MHz, $CDCl_3$)

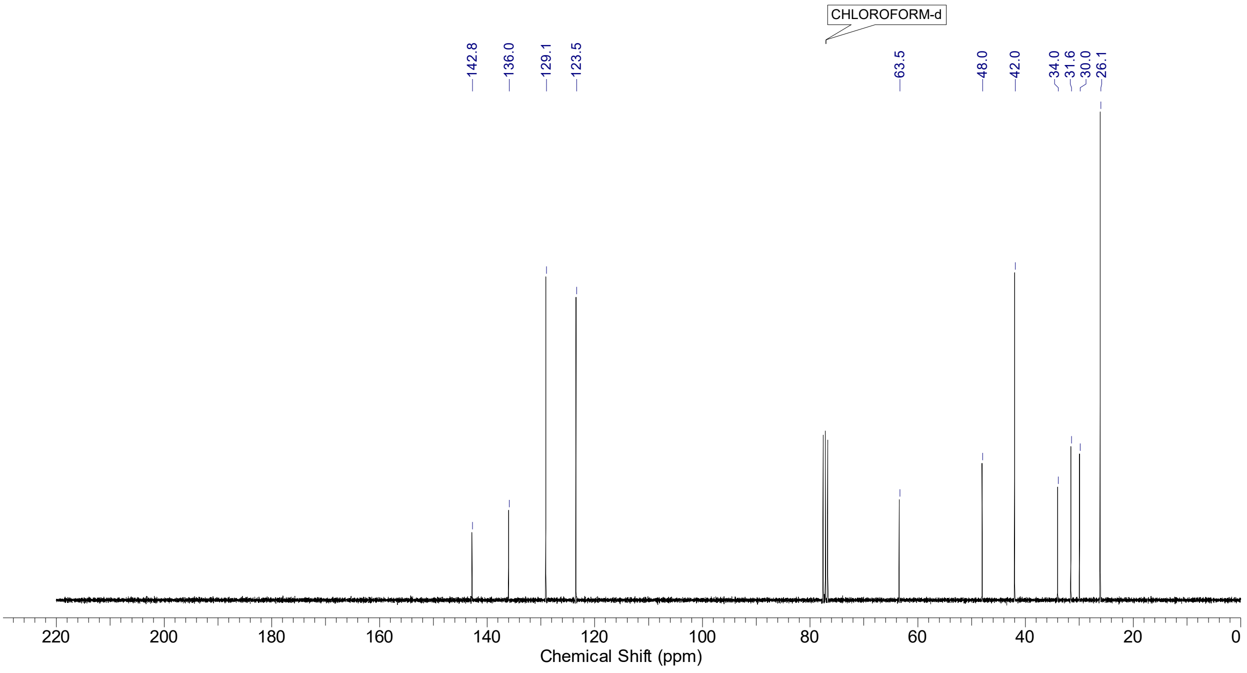

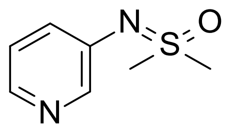

dimethyl-oxo-(3-pyridylimino)-λ⁶-sulfane [CAS: 1621962-58-0] **(3ya)**

^{1}H NMR (300 MHz, $CDCl_3$)

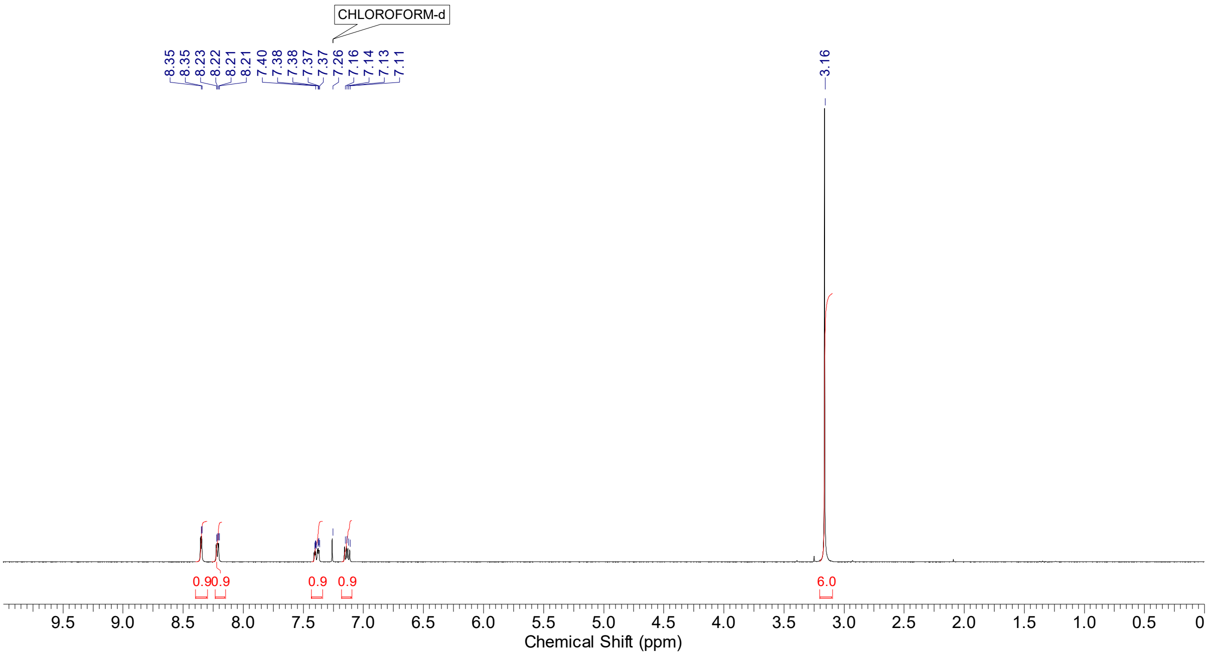

^{13}C NMR (75 MHz, $CDCl_3$)

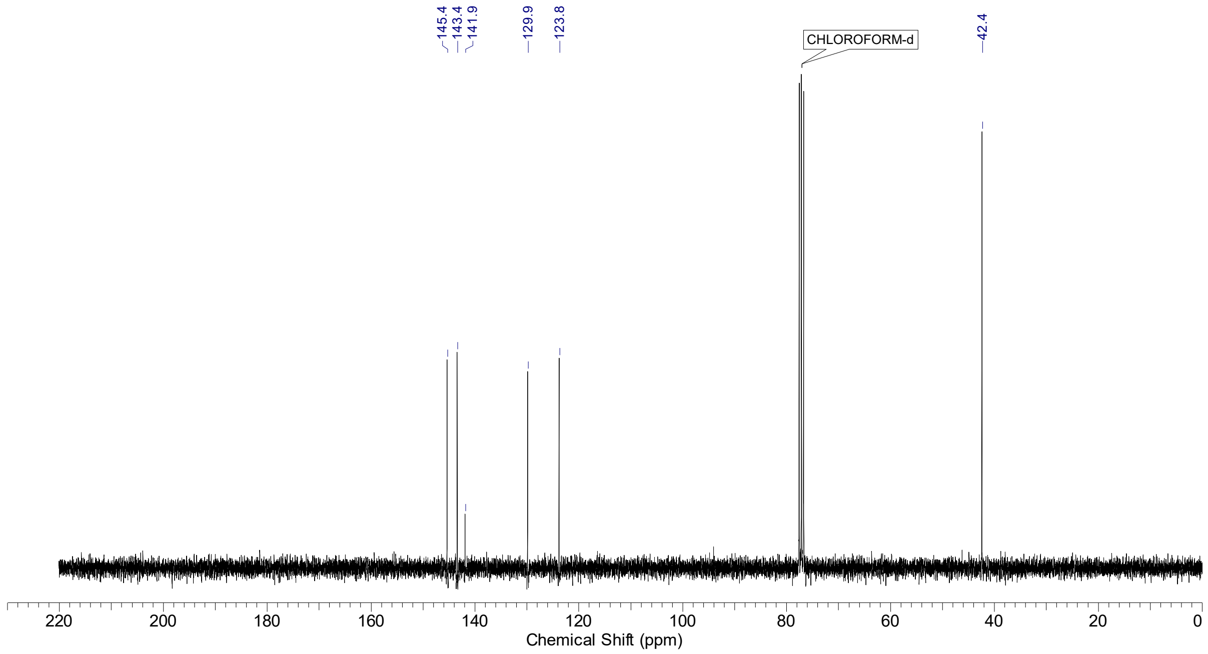

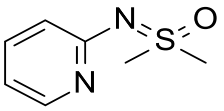

dimethyl-oxo-(2-pyridylimino)-λ⁶-sulfane [CAS: 2059938-84-8] **(3za)**

^{1}H NMR (300 MHz, $CDCl_3$)

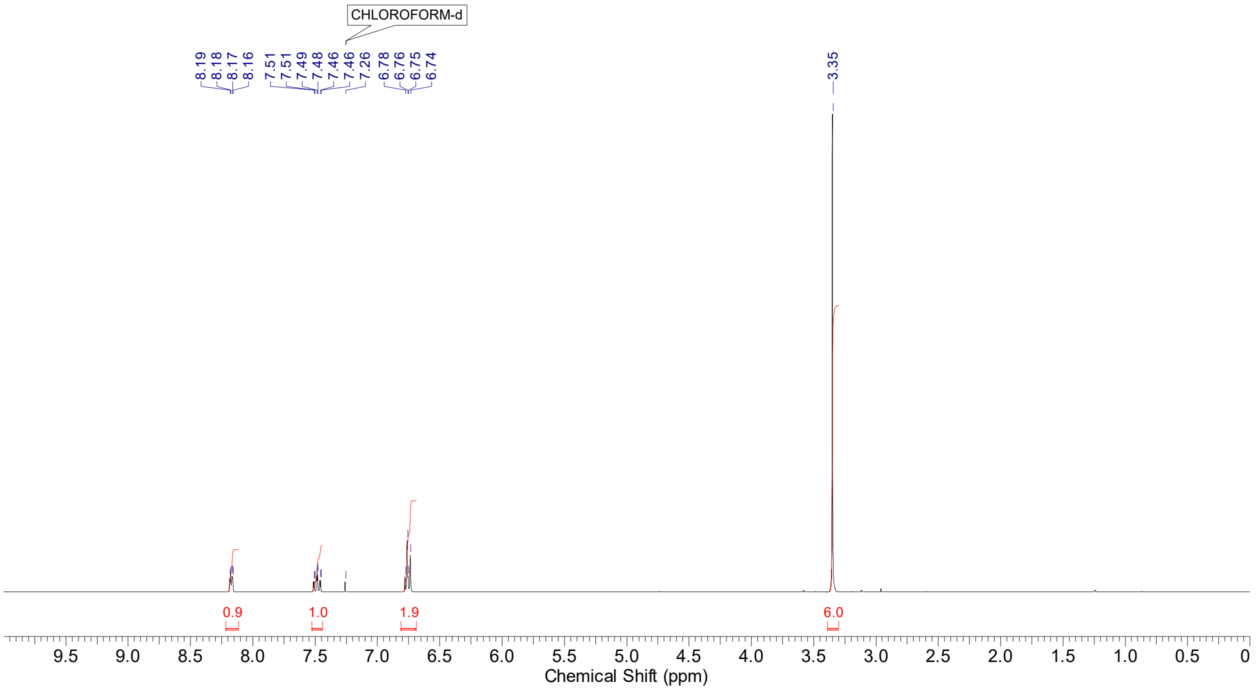

^{13}C NMR (75 MHz, $CDCl_3$)

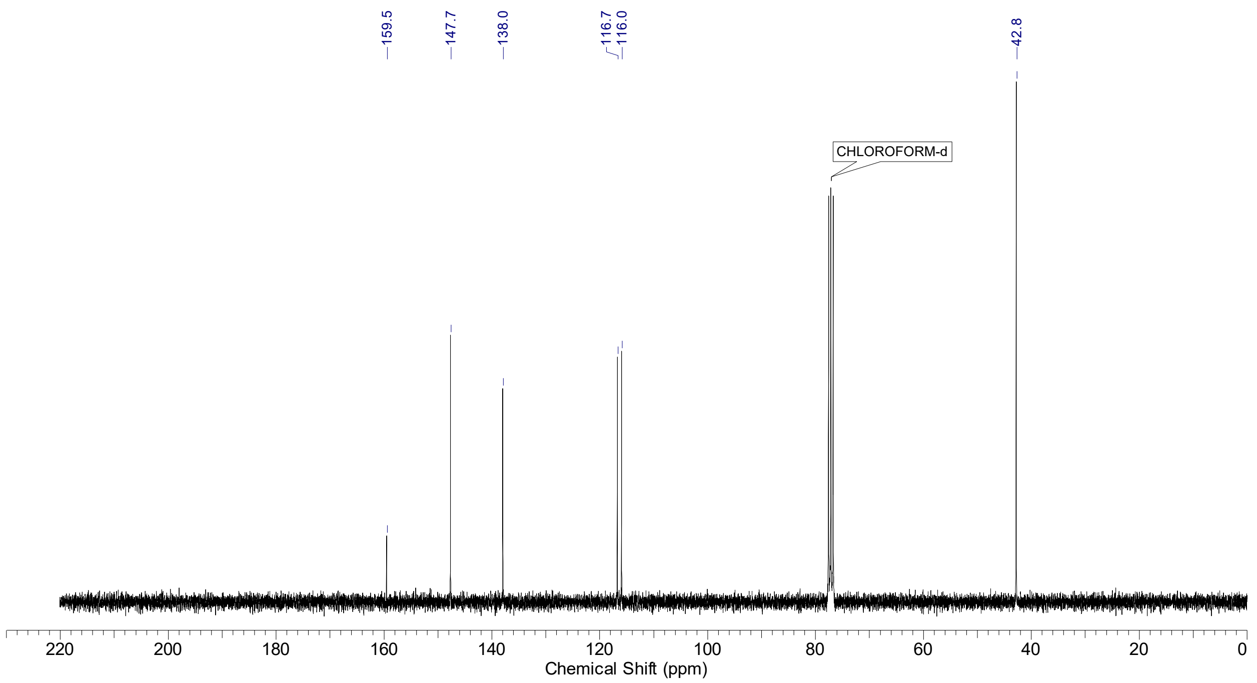

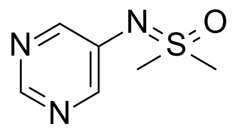

dimethyl-oxo-pyrimidin-5-ylimino-λ⁶-sulfane [CAS: 2377300-50-8] **(4)**

^{1}H NMR (300 MHz, $CDCl_3$)

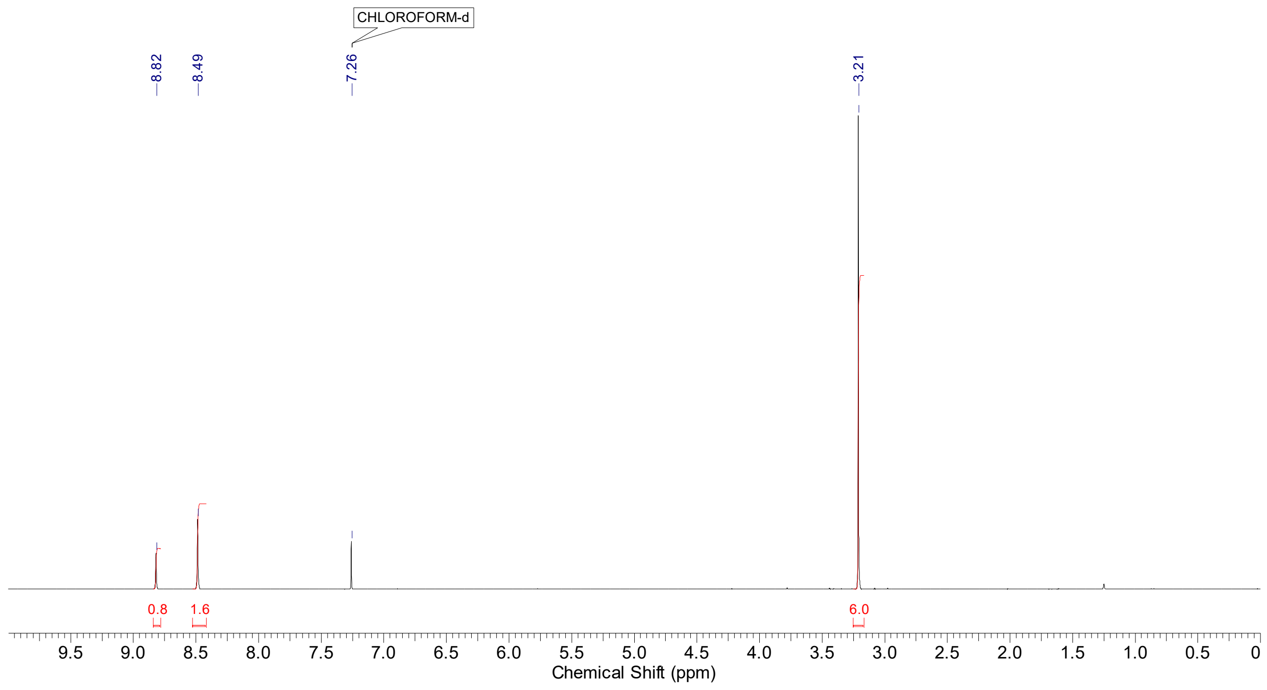

^{13}C NMR (75 MHz, $CDCl_3$)

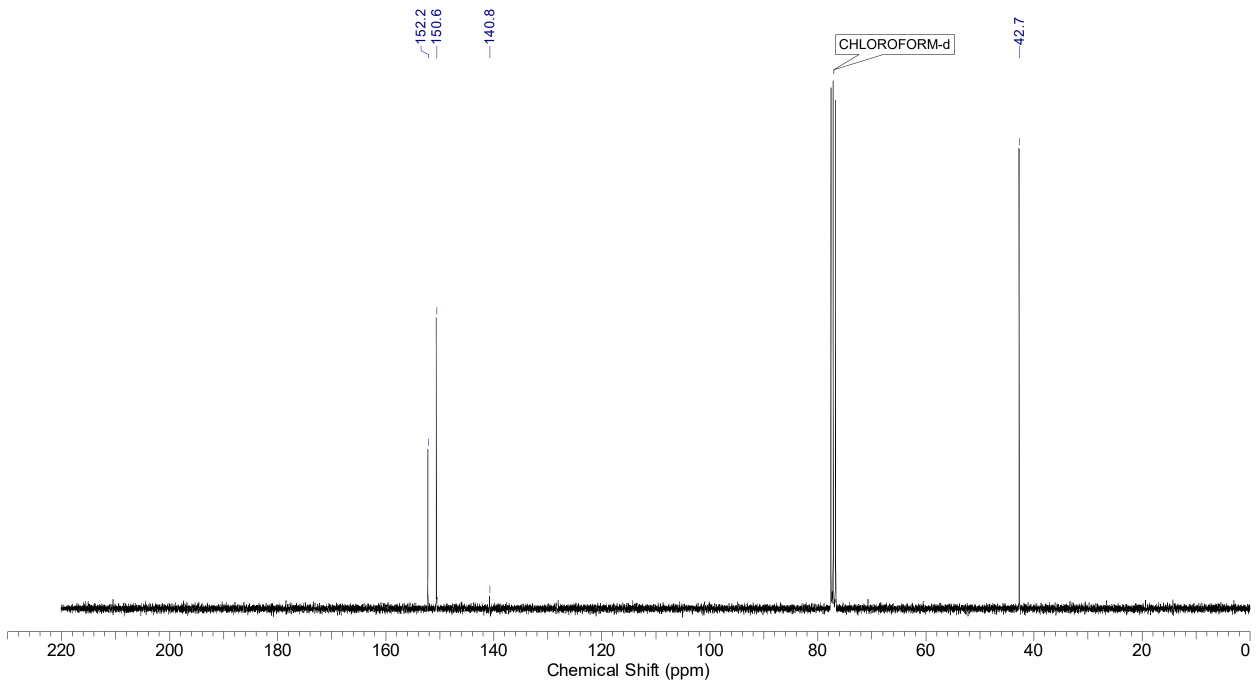

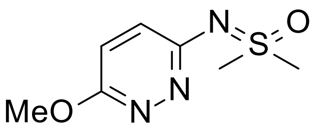

(6-methoxypyridazin-3-yl)imino-dimethyl-oxo-λ⁶-sulfane **(5)**

^{1}H NMR (300 MHz, $CDCl_3$)

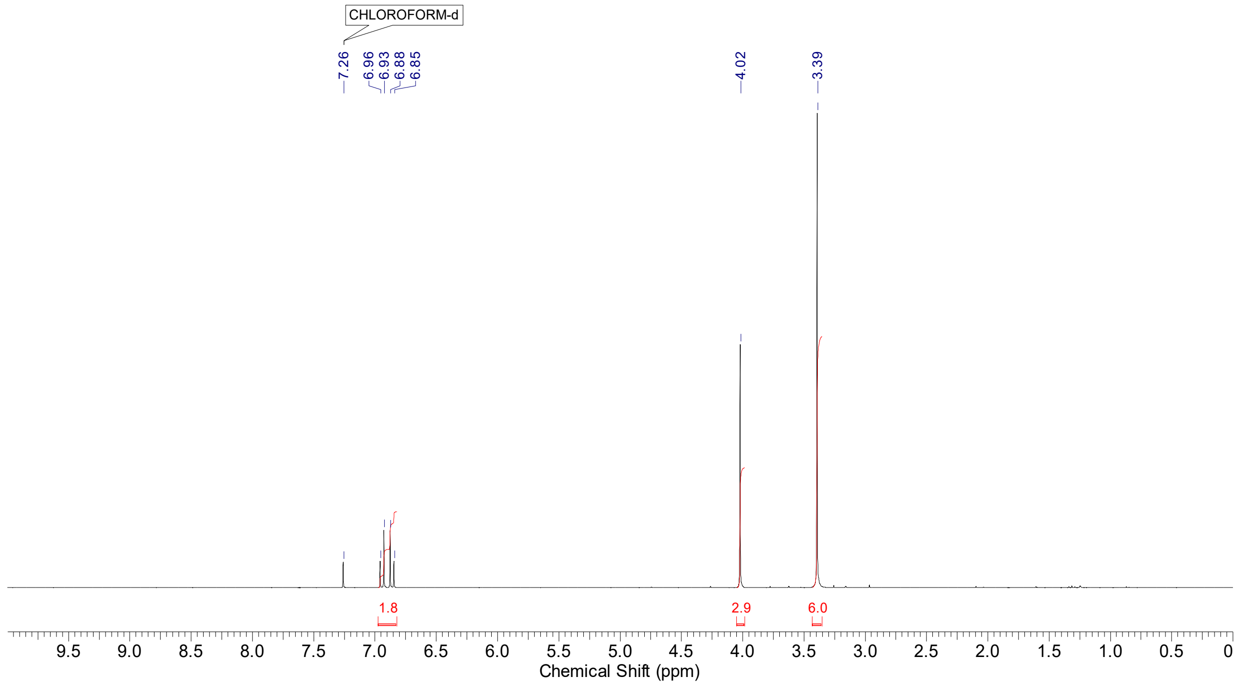

^{13}C NMR (75 MHz, $CDCl_3$)

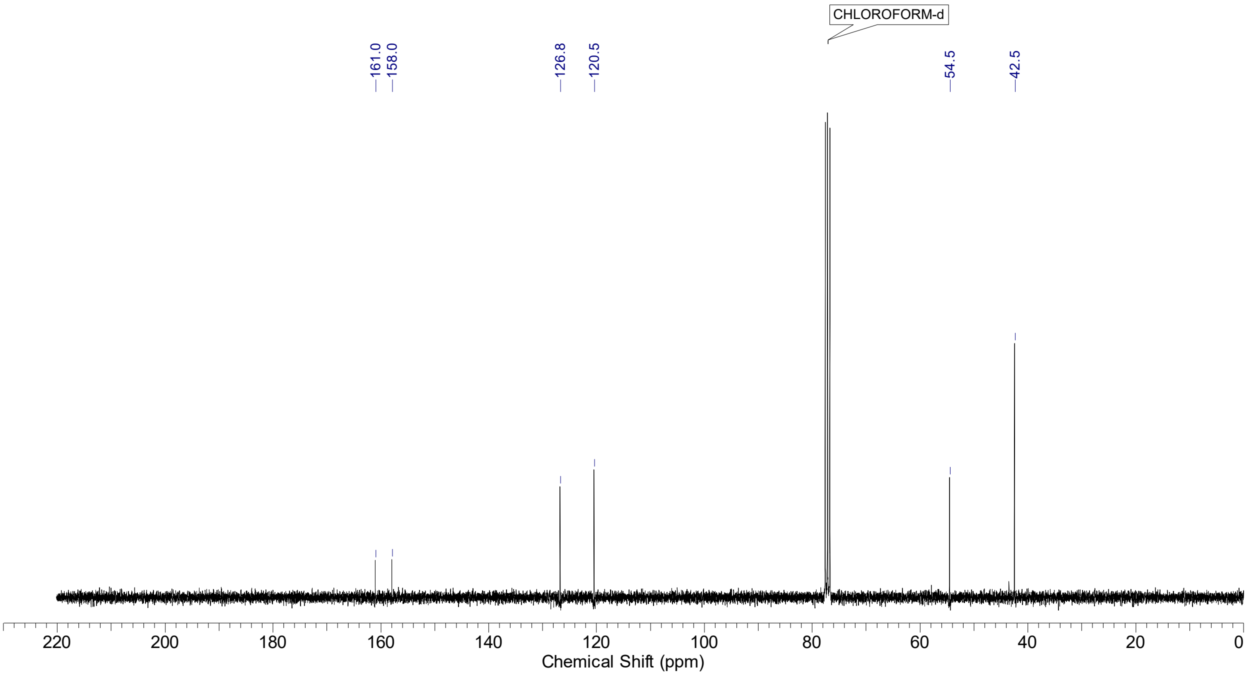

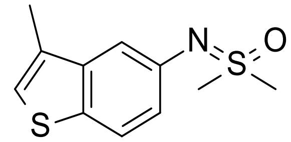

dimethyl-(3-methylbenzothiophen-5-yl)imino-oxo-λ⁶-sulfane **(6)**

^{1}H NMR (400 MHz, $CDCl_3$)

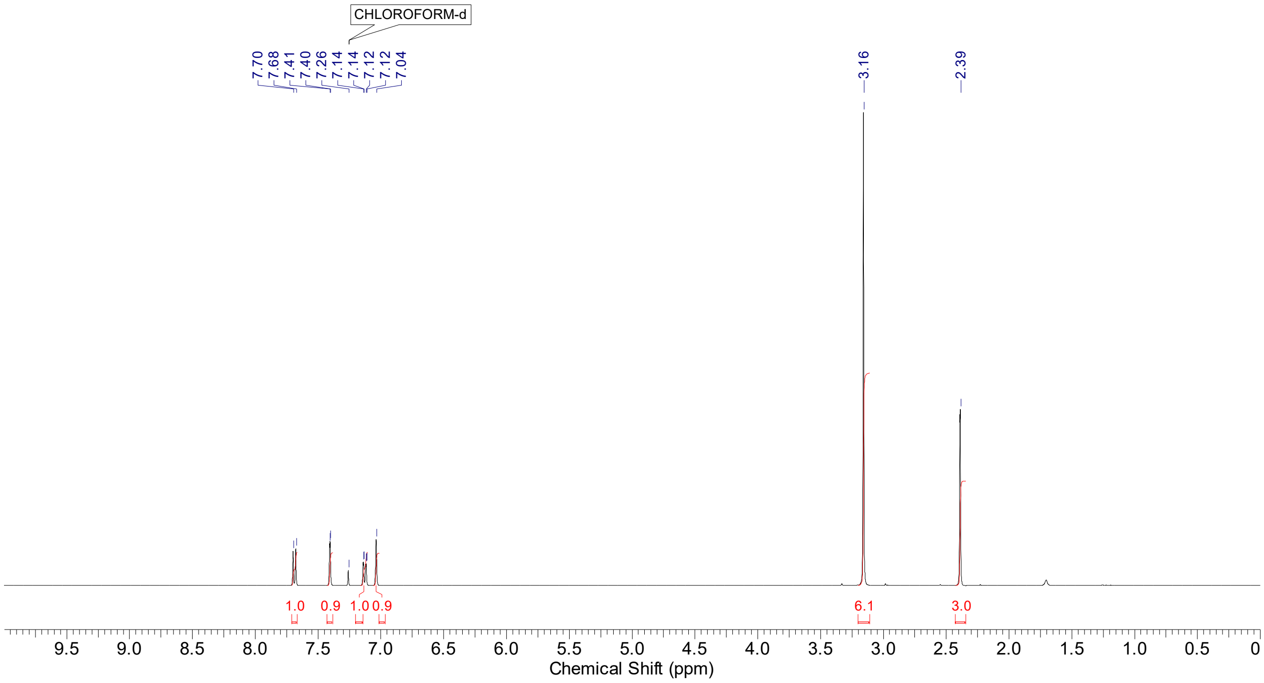

^{13}C NMR (101 MHz, $CDCl_3$)

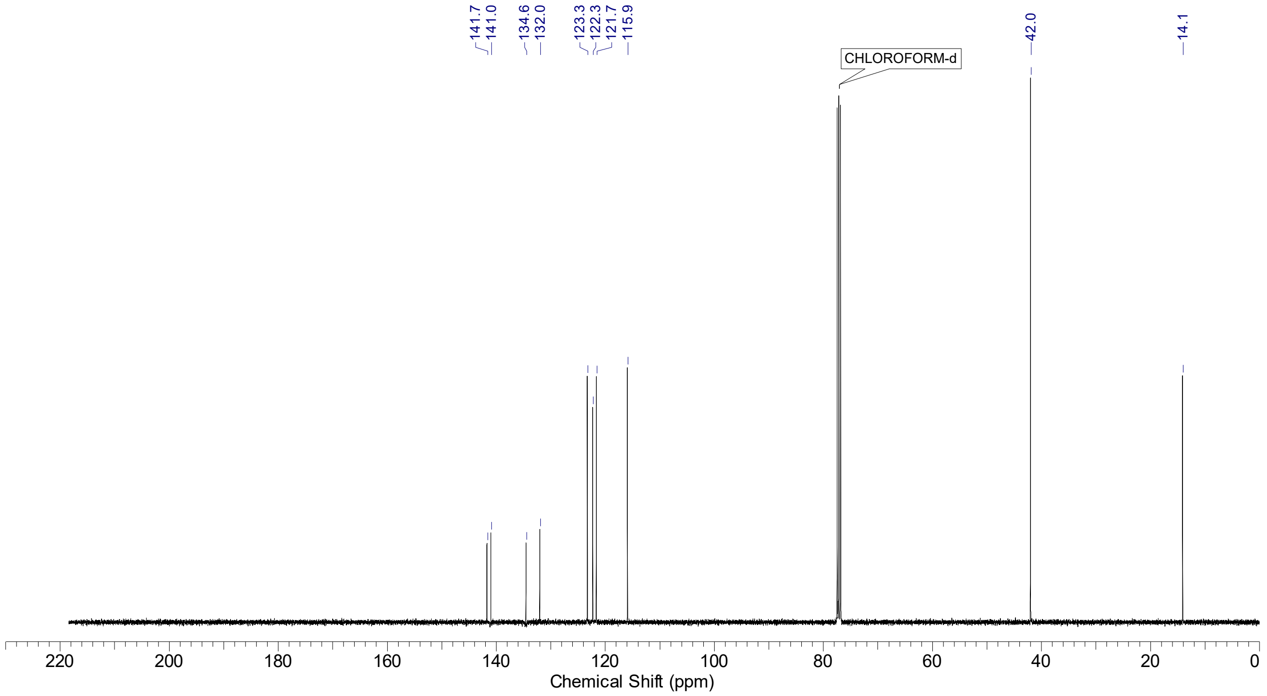

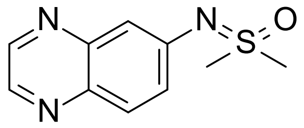

dimethyl-oxo-quinoxalin-6-ylimino-λ⁶-sulfane [CAS: 3067858-92-5] **(7)**

^{1}H NMR (400 MHz, $CDCl_3$)

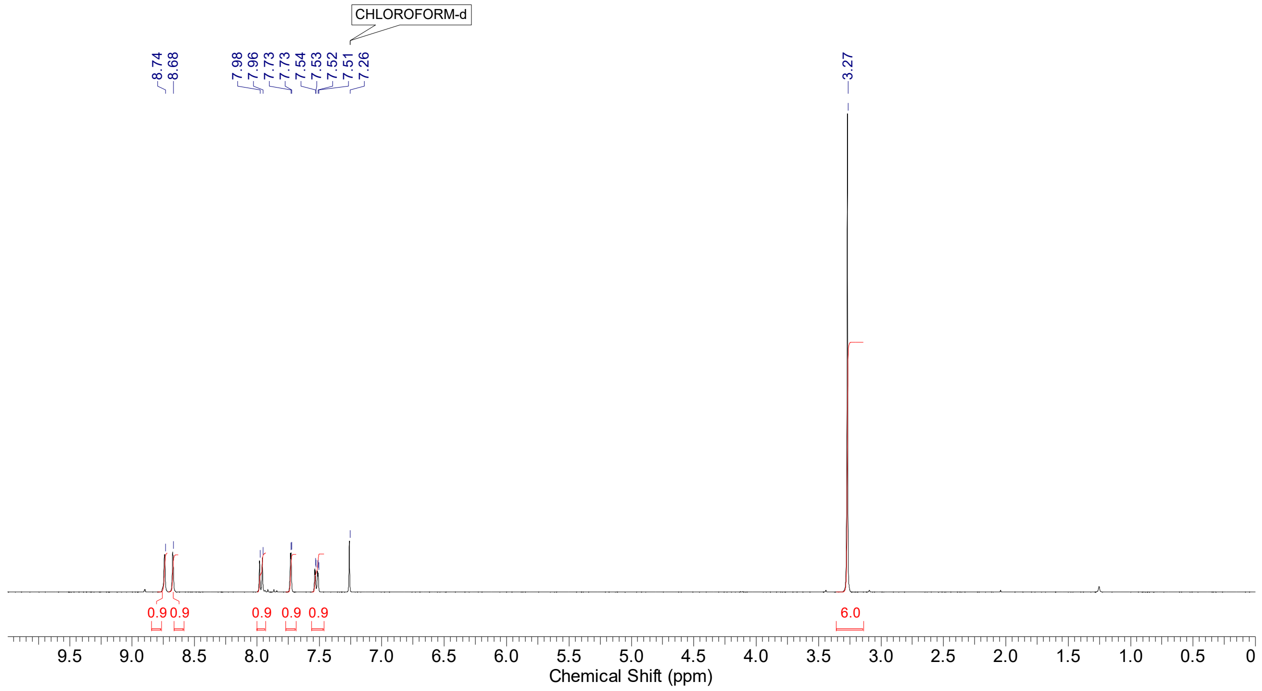

^{13}C NMR (101 MHz, $CDCl_3$)

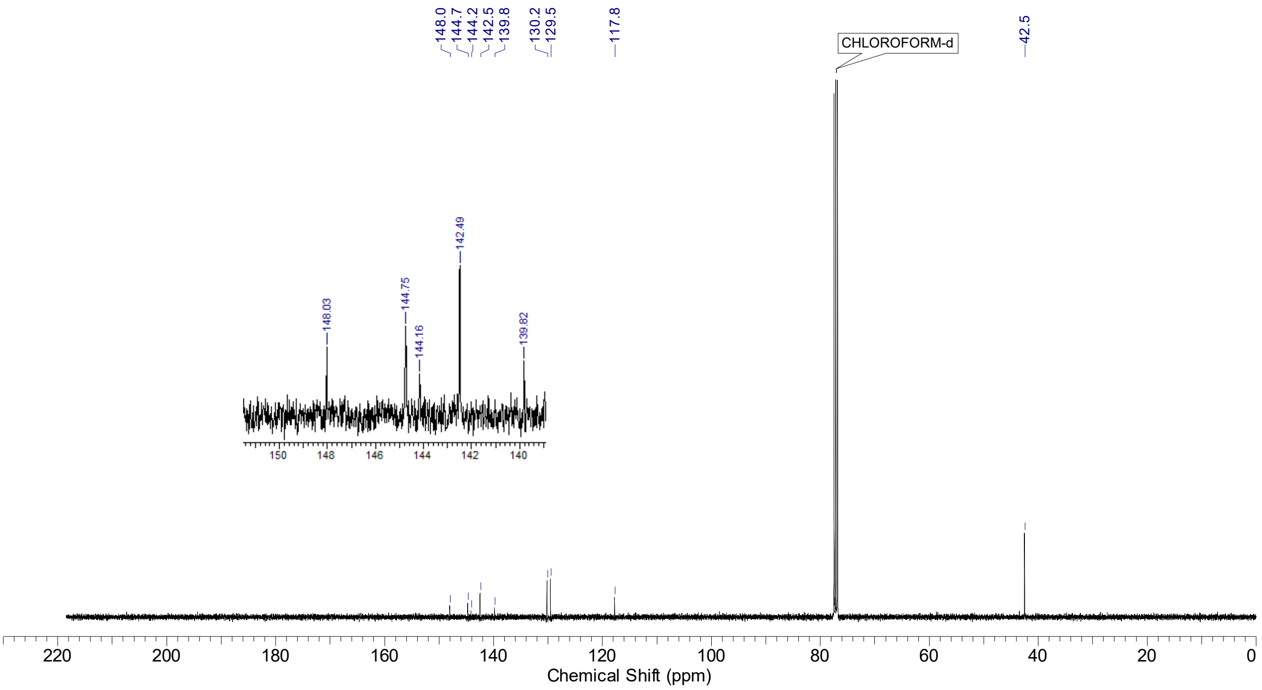

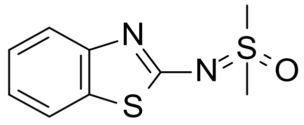

1,3-benzothiazol-2-ylimino-dimethyl-oxo-λ⁶-sulfane [CAS: 3067858-93-6] **(8)**

^{1}H NMR (400 MHz, $CDCl_3$)

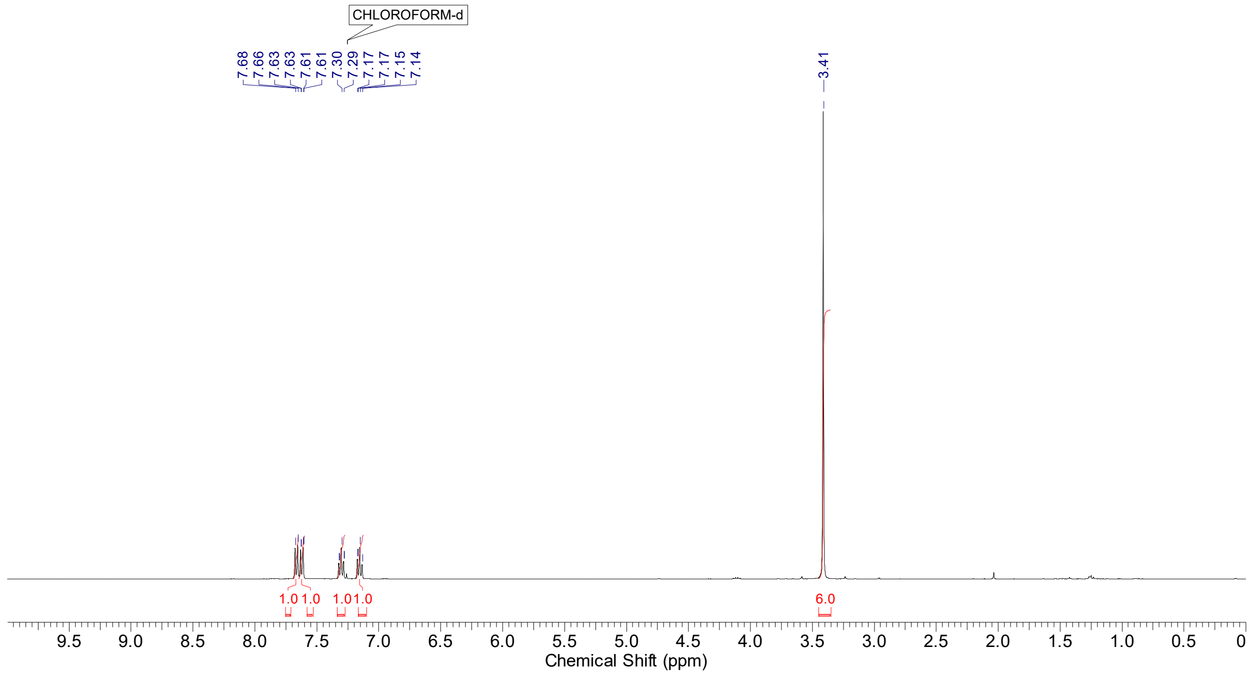

^{13}C NMR (101 MHz, $CDCl_3$)

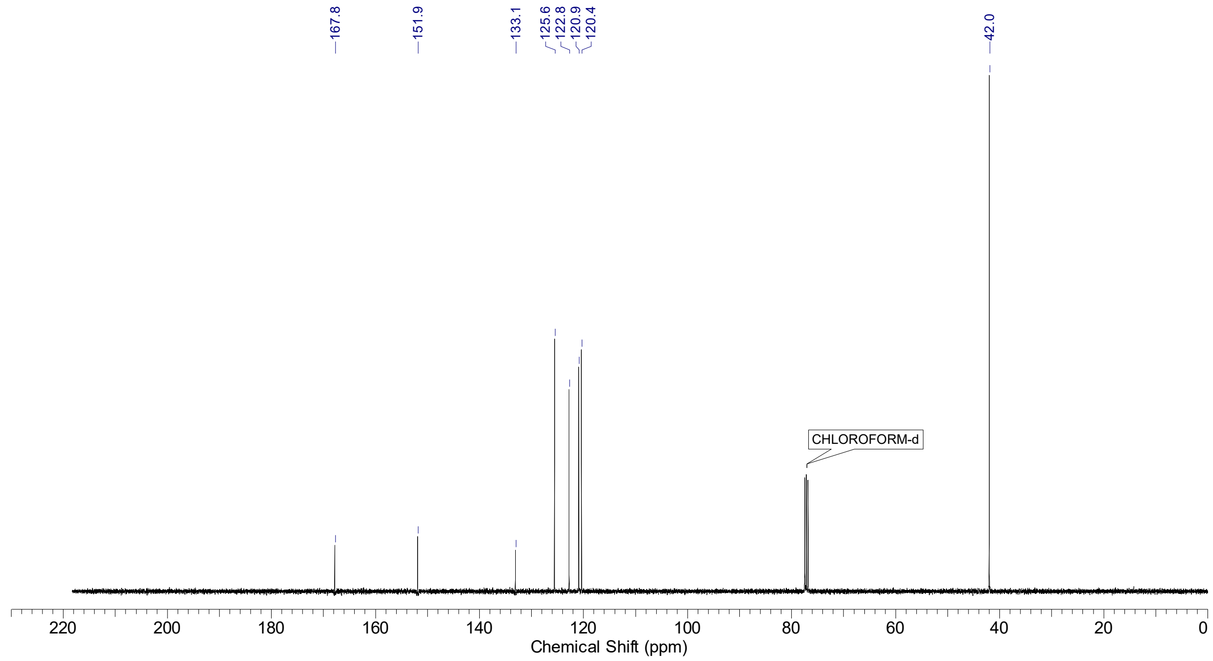

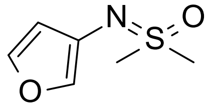

3-furylimino-dimethyl-oxo-λ⁶-sulfane **(9)**

^{1}H NMR (300 MHz, $CDCl_3$)

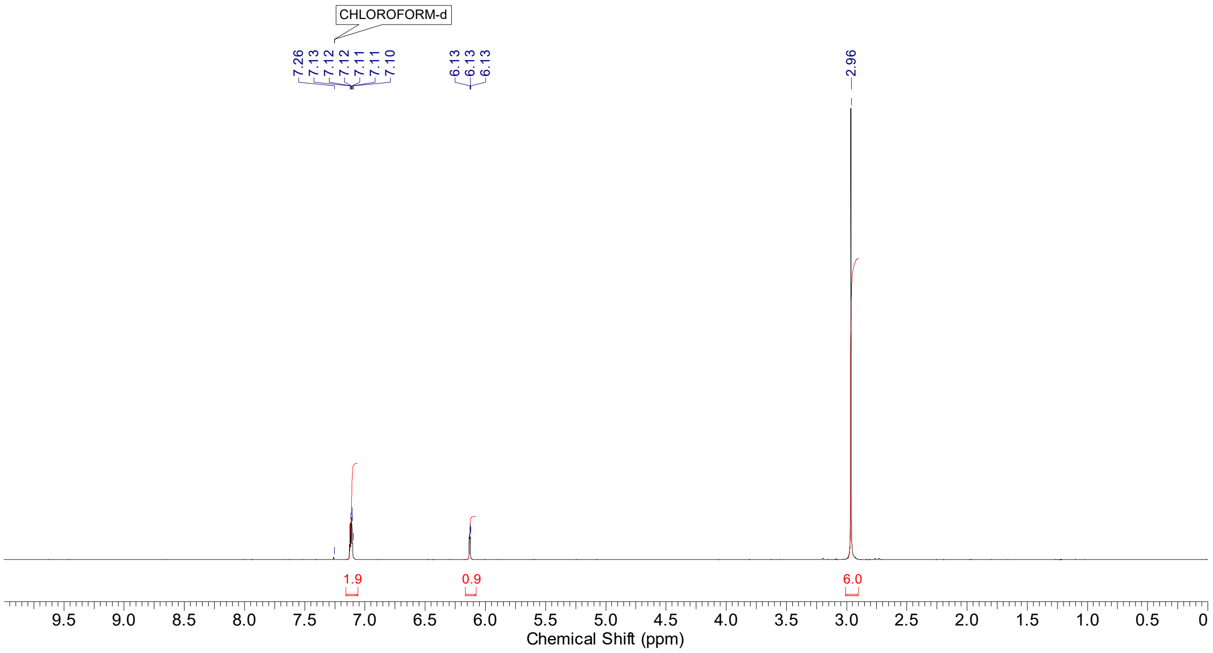

^{13}C NMR (75 MHz, $CDCl_3$)

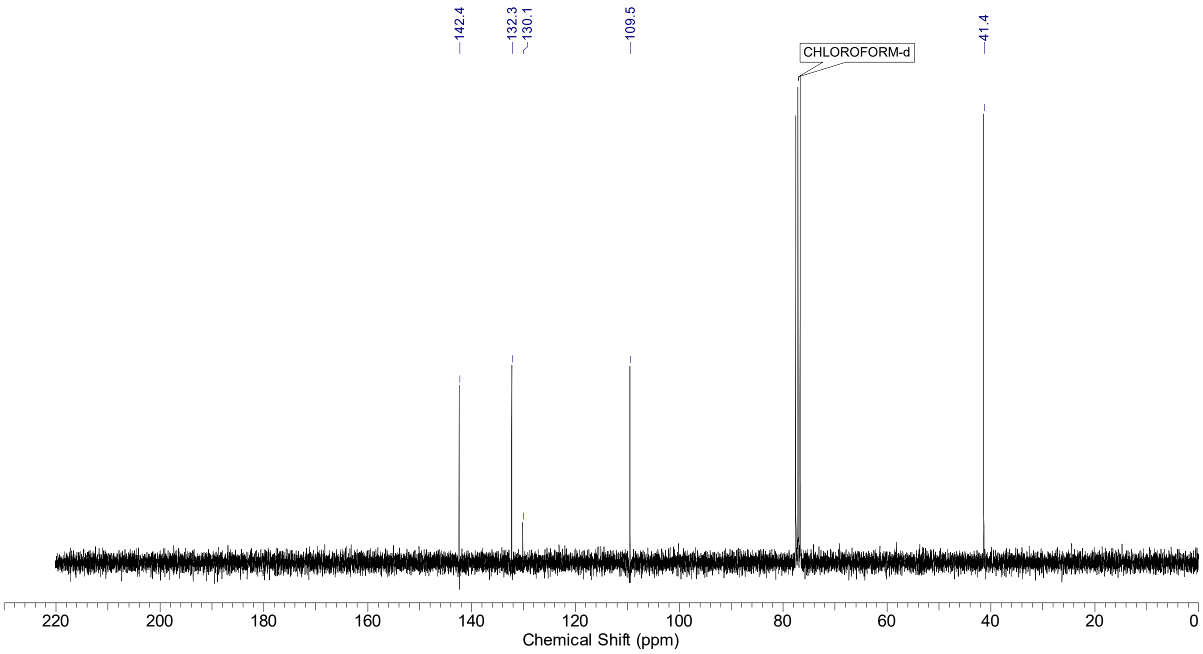

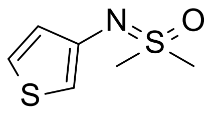

dimethyl-oxo-(3-thienylimino)-λ⁶-sulfane [CAS: 2377300-49-5] **(10)**

^{1}H NMR (300 MHz, $CDCl_3$)

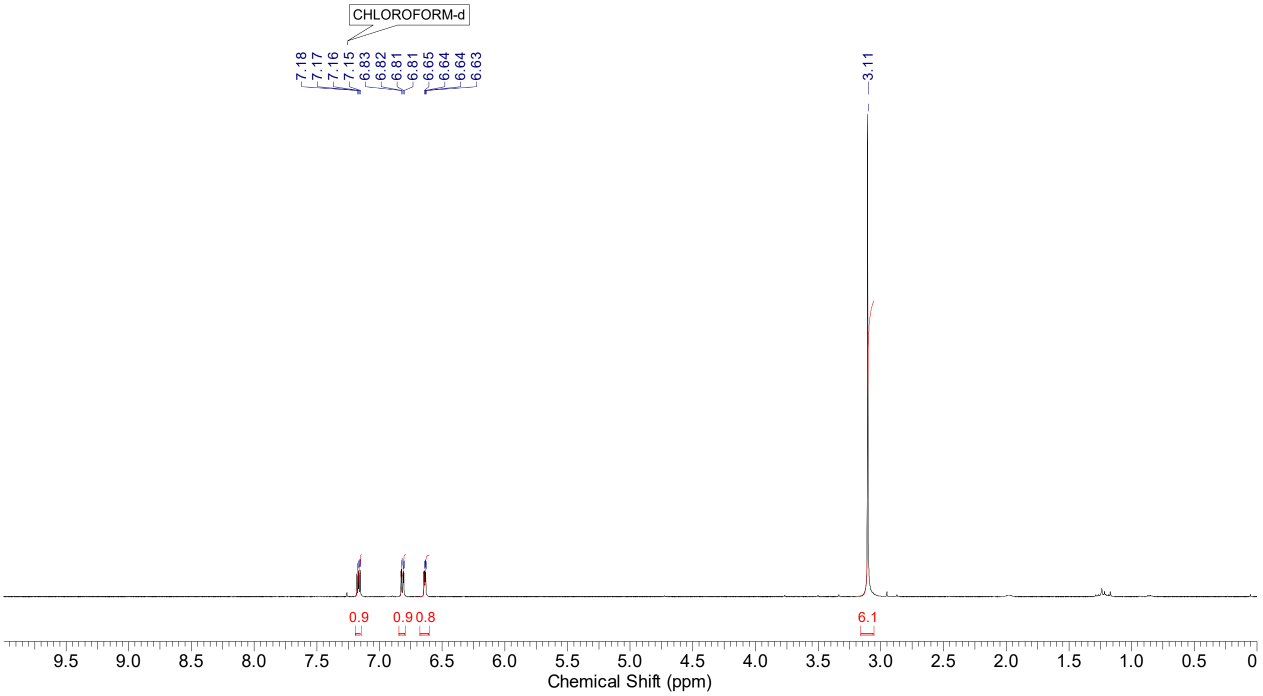

^{13}C NMR (75 MHz, $CDCl_3$)

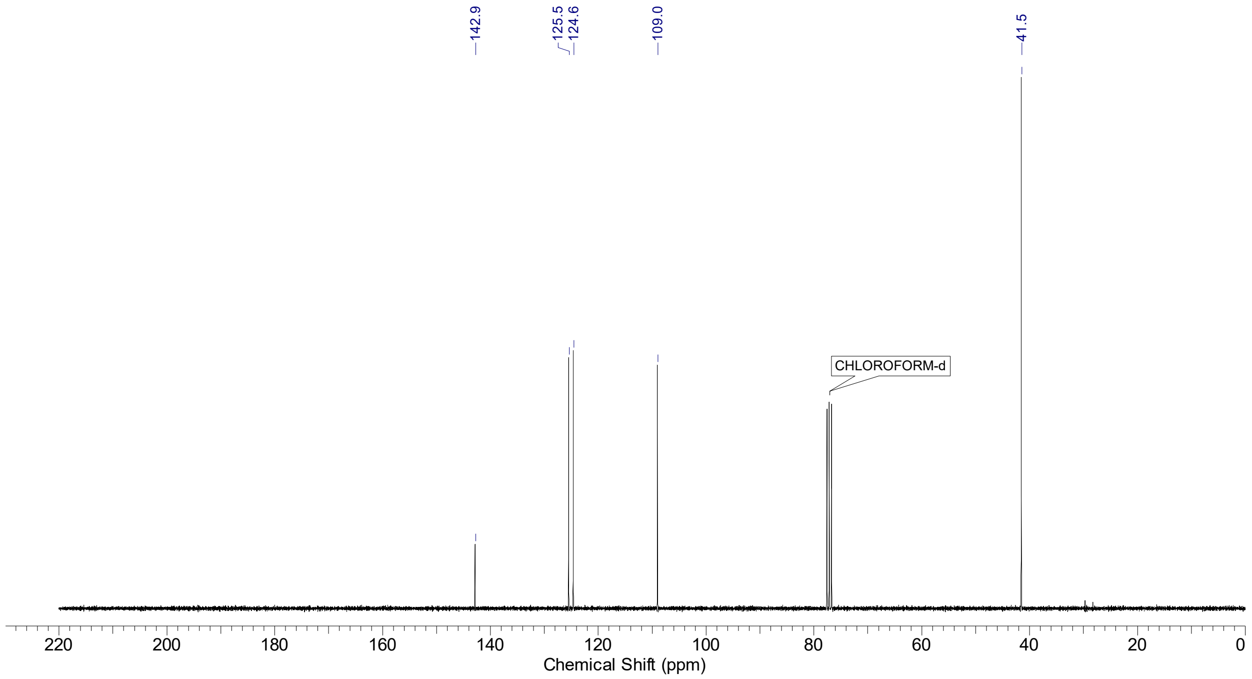

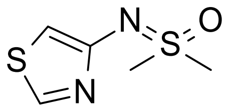

dimethyl-oxo-thiazol-4-ylimino-λ⁶-sulfane **(11)**

^{1}H NMR (300 MHz, $CDCl_3$)

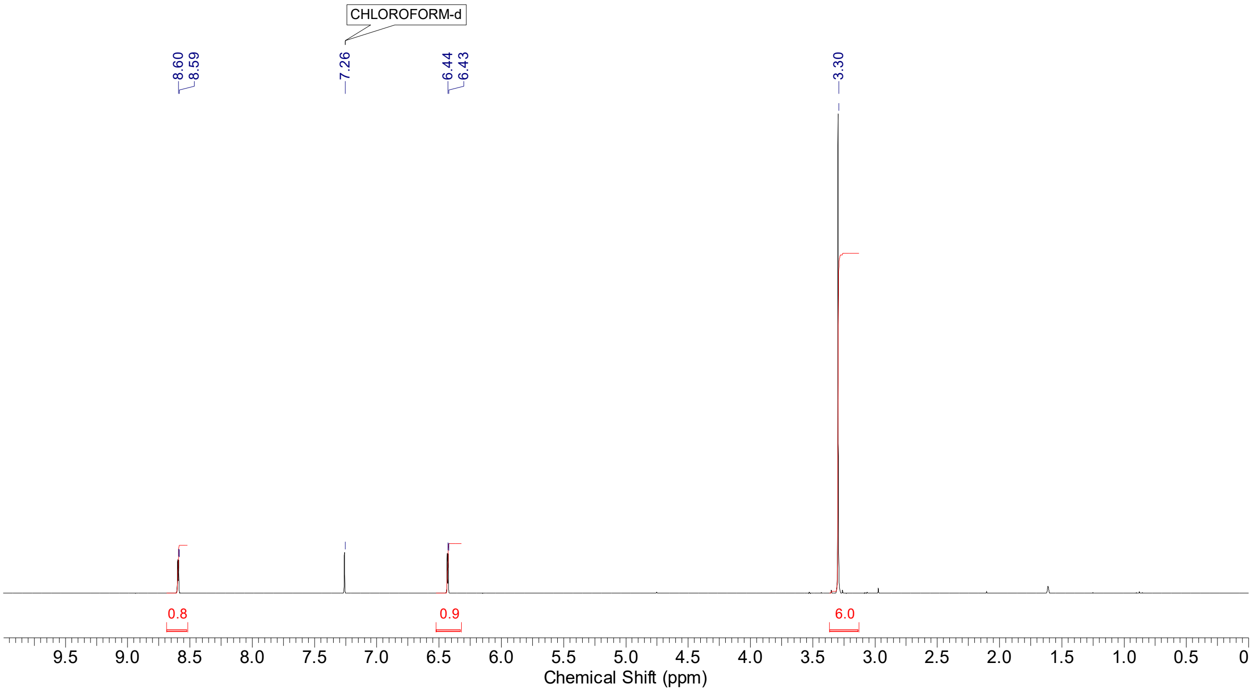

^{13}C NMR (75 MHz, $CDCl_3$)

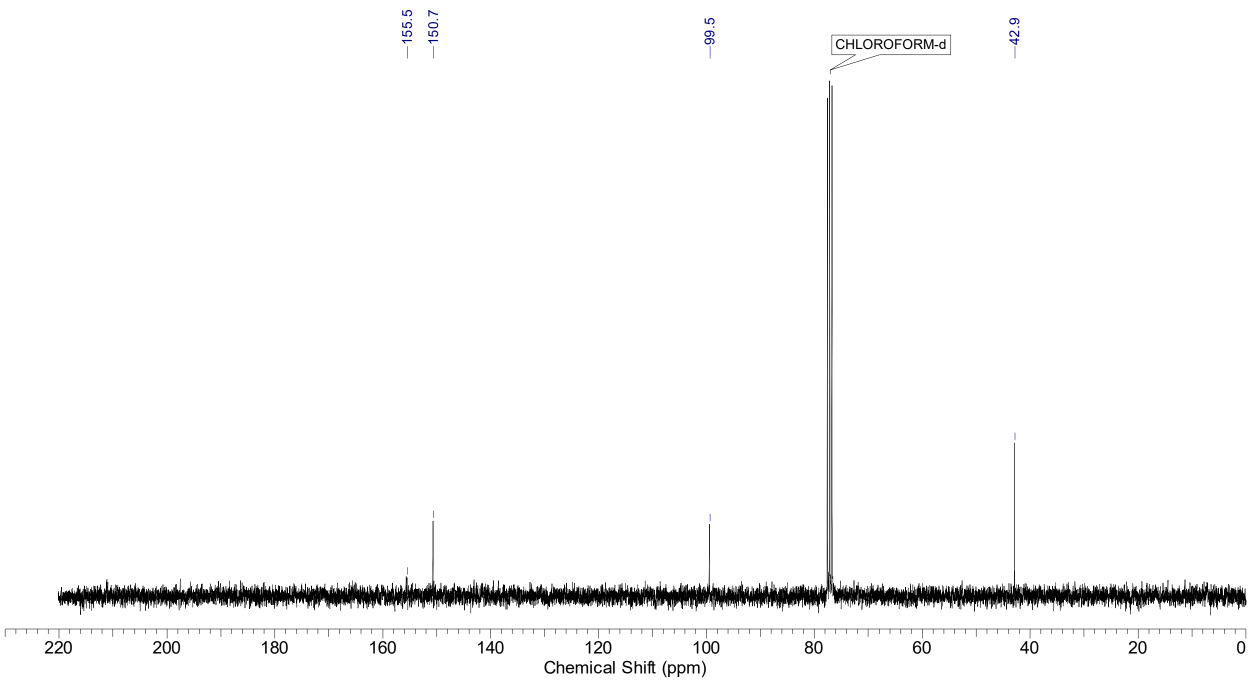

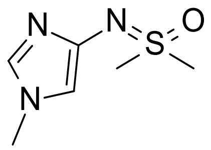

dimethyl-(1-methylimidazol-4-yl)imino-oxo-λ⁶-sulfane **(12)**

^{1}H NMR (300 MHz, $CDCl_3$)

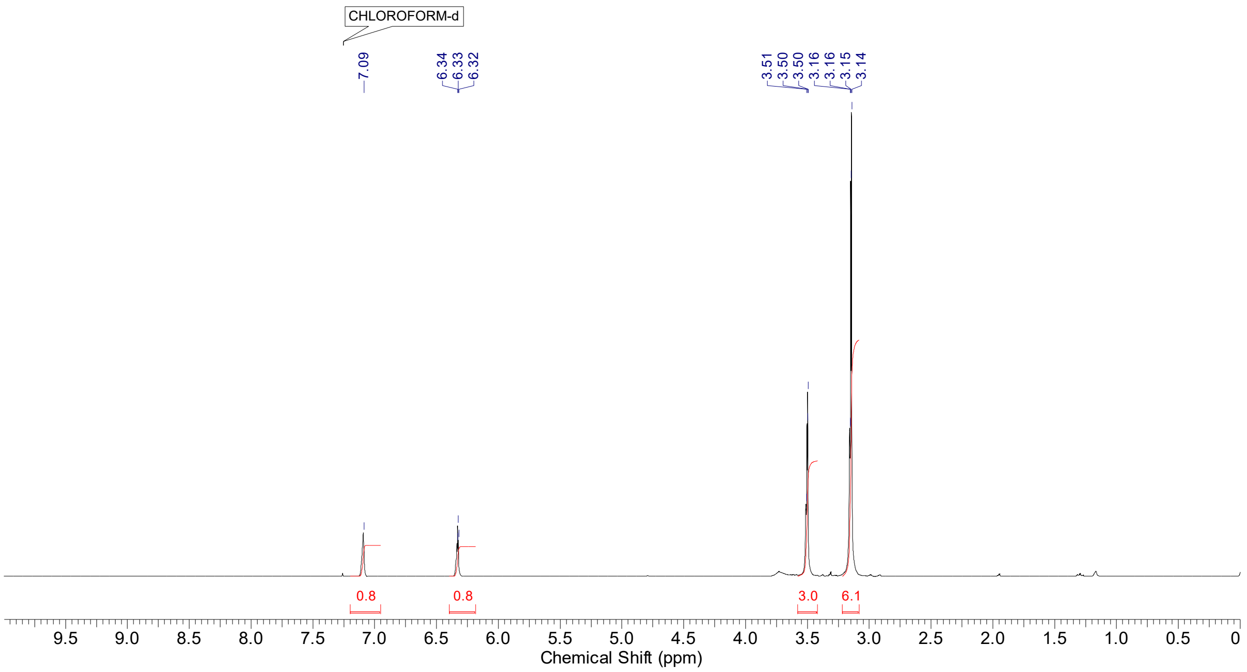

^{13}C NMR (75 MHz, $CDCl_3$)

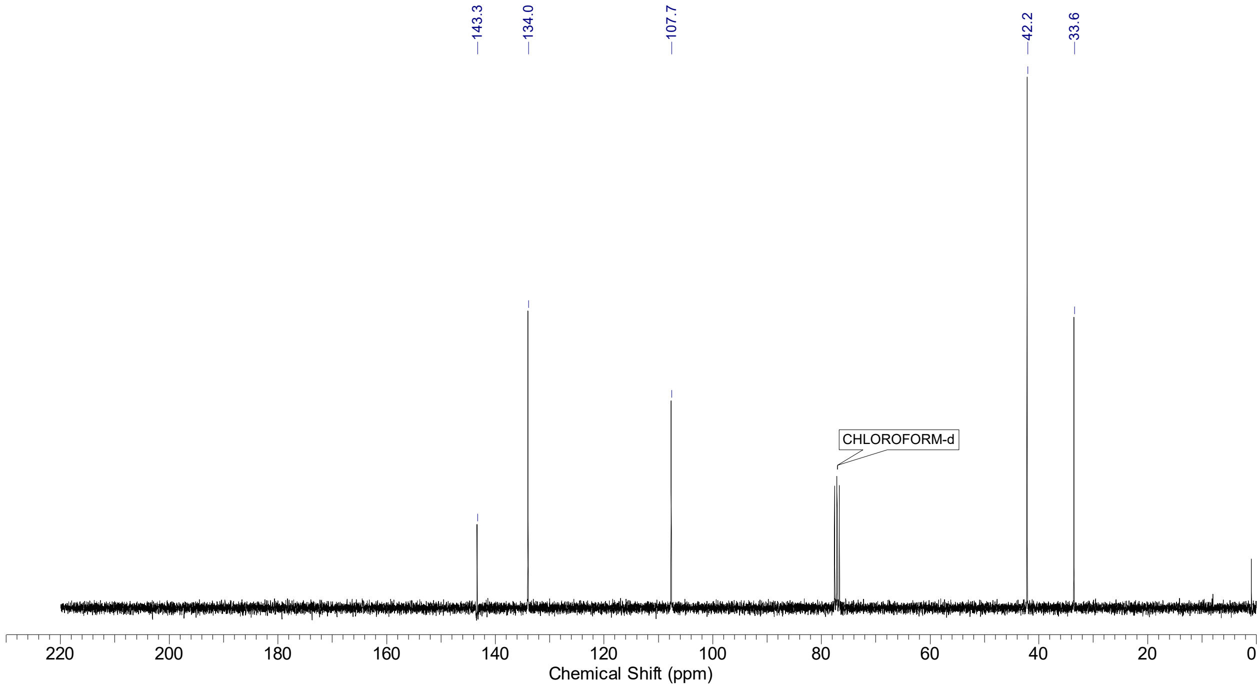

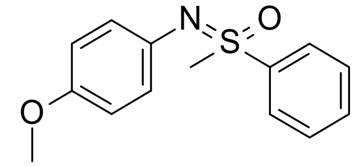

(4-methoxyphenyl)imino-methyl-oxo-phenyl-λ⁶-sulfane [CAS: 83706-37-0] **(3ab)**

^{1}H NMR (400 MHz, $CDCl_3$)

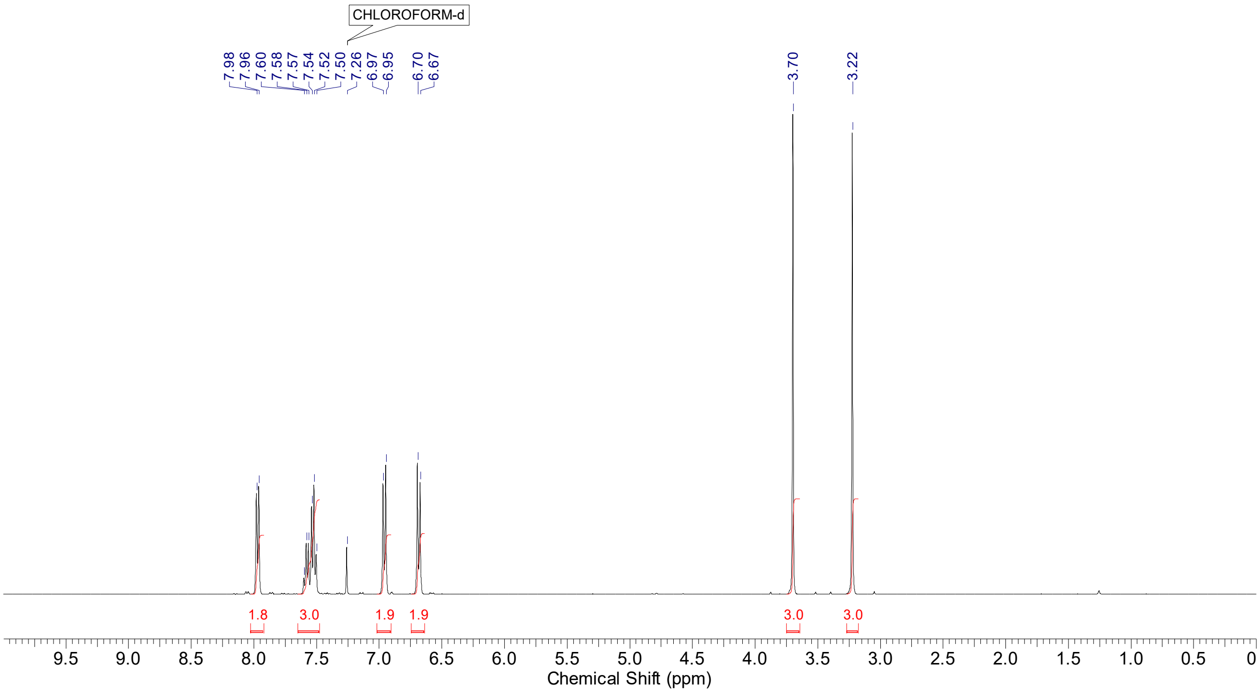

^{13}C NMR (101 MHz, $CDCl_3$)

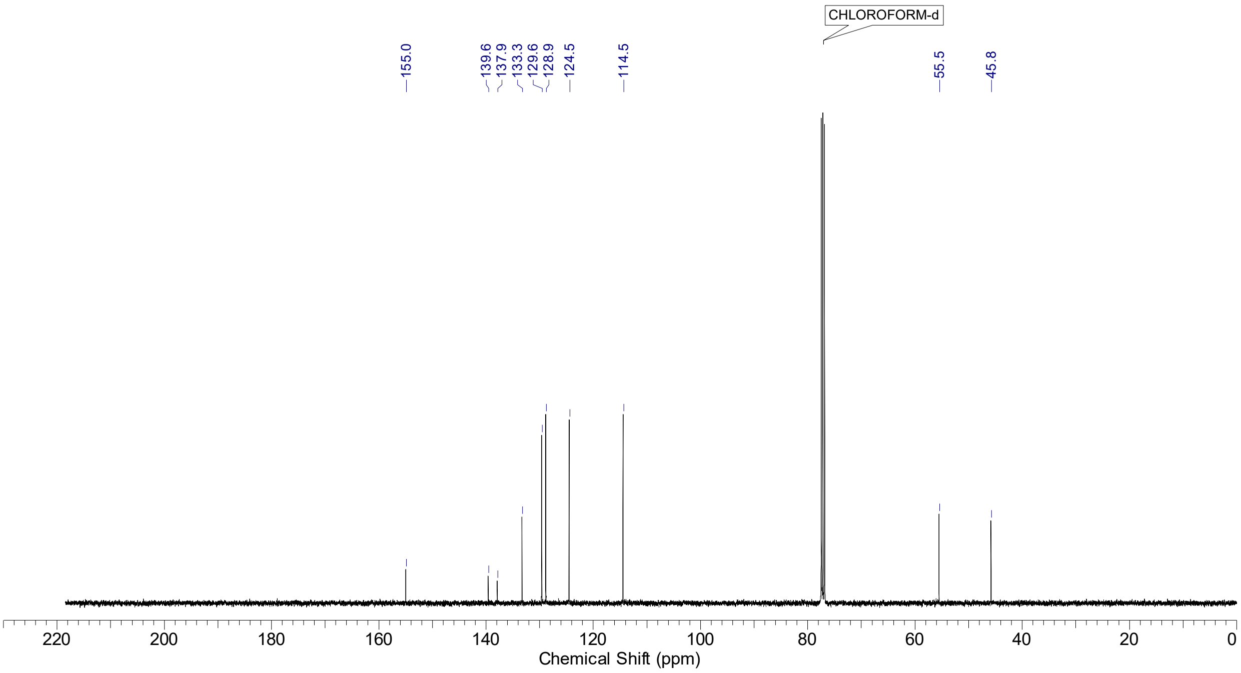

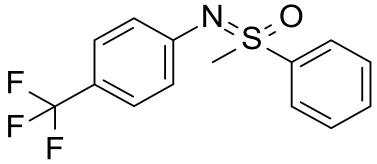

methyl-oxo-phenyl-[4-(trifluoromethyl)phenyl]imino-λ⁶-sulfane [CAS: 257955-73-0] **(3hb)**

^{1}H NMR (400 MHz, $CDCl_3$)

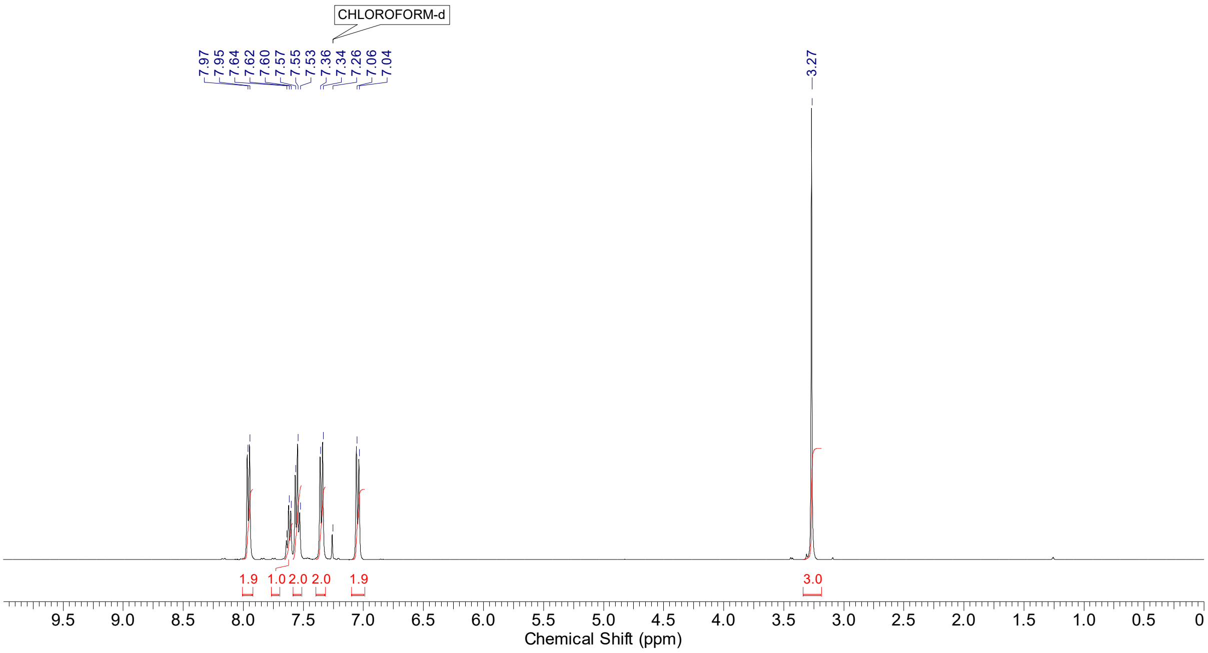

^{13}C NMR (101 MHz, $CDCl_3$)

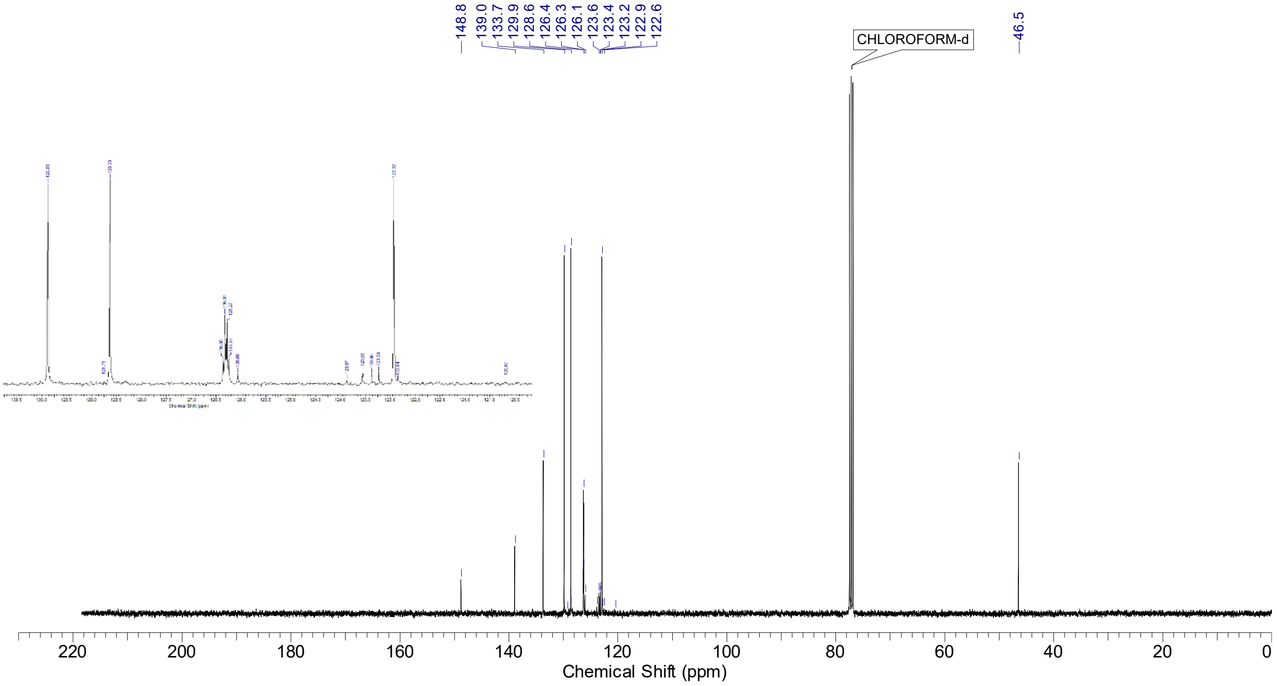

^{19}F NMR (76 MHz, $CDCl_3$, $C_6H_4F_2$)

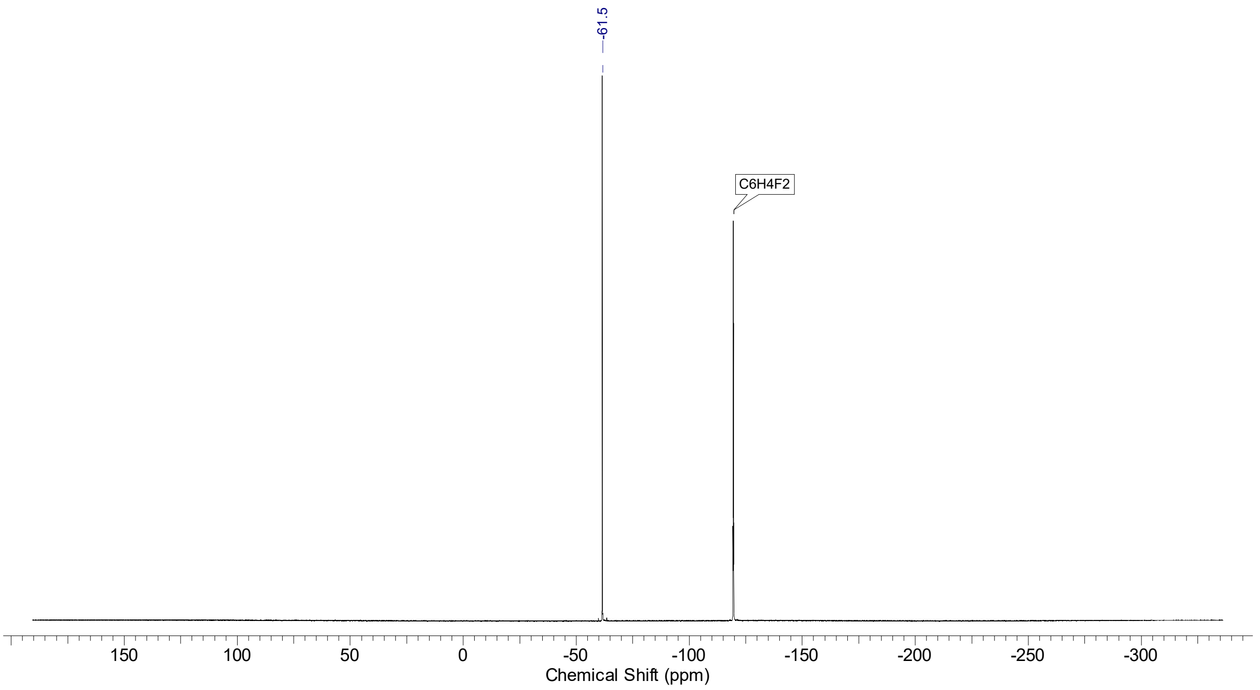

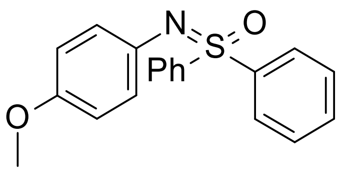

(4-methoxyphenyl)imino-oxo-diphenyl-λ^6-sulfane [CAS: 1374007-24-5] **(3ac)**

^1H NMR (300 MHz, $CDCl_3$)

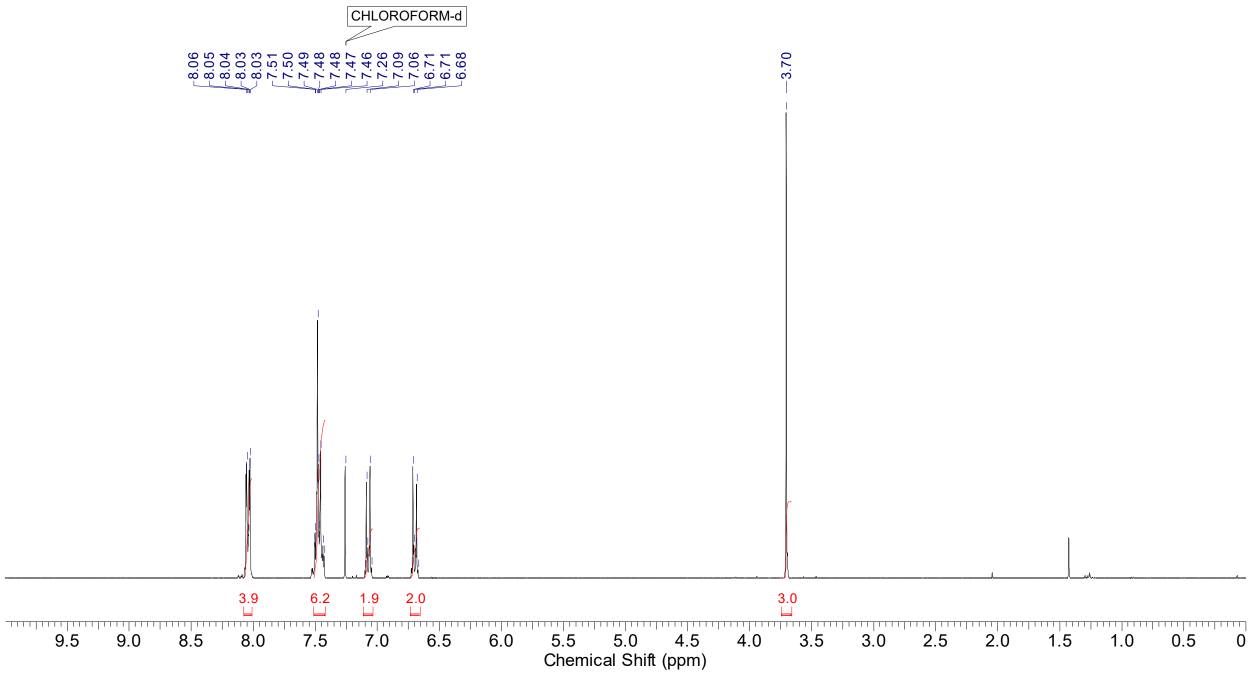

^{13}C NMR (75 MHz, $CDCl_3$)

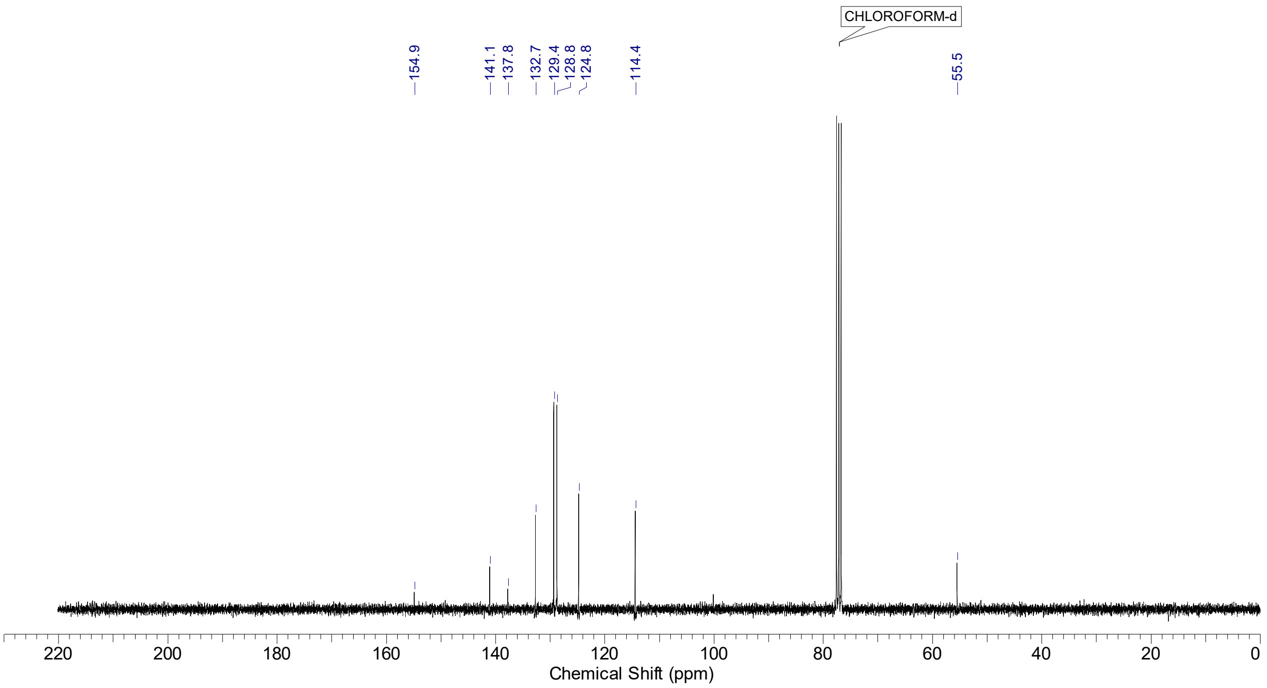

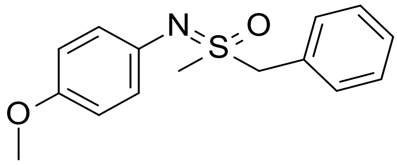

benzyl-(4-methoxyphenyl)imino-methyl-oxo-λ⁶-sulfane **(3ad)**

^{1}H NMR (300 MHz, $CDCl_3$)

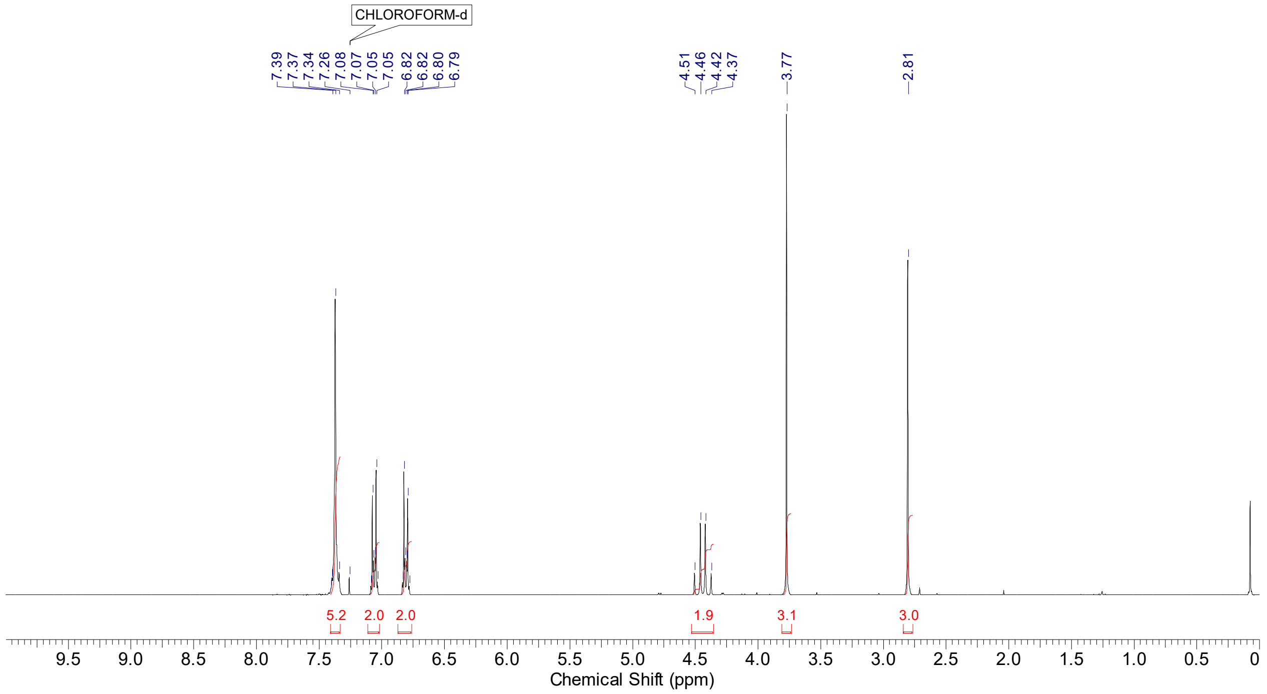

^{13}C NMR (75 MHz, $CDCl_3$)

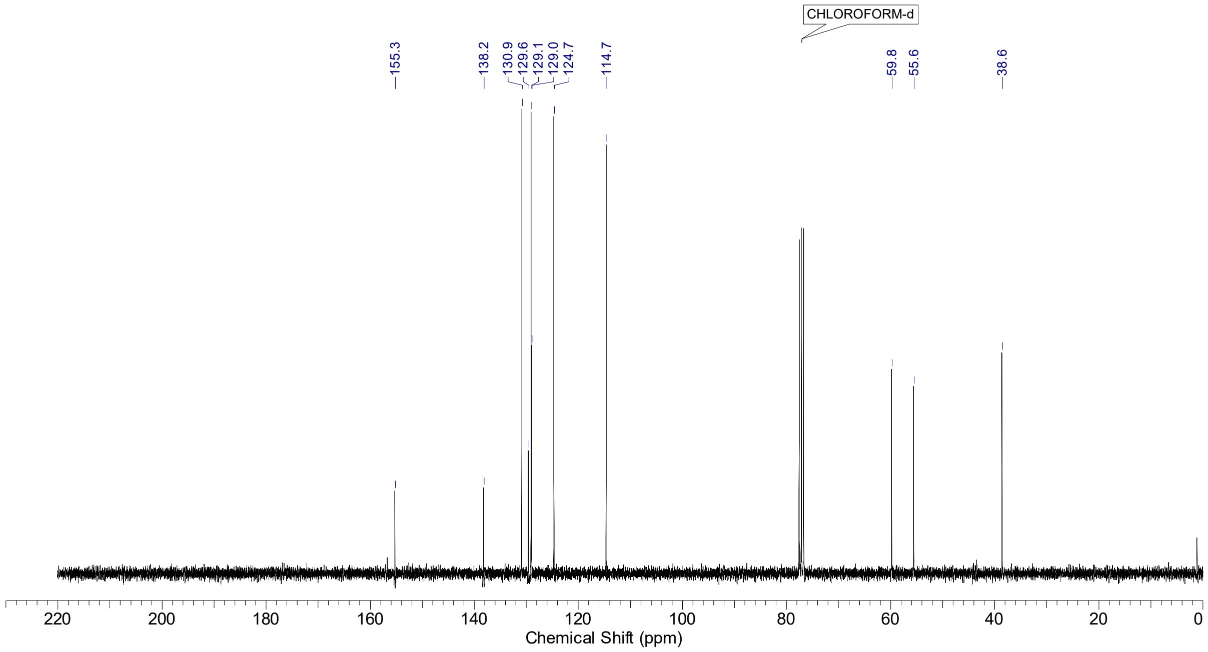

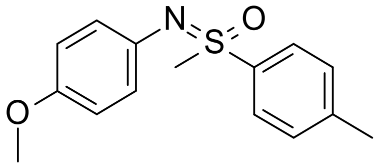

(4-methoxyphenyl)imino-methyl-oxo-(p-tolyl)-λ⁶-sulfane [CAS: 2811710-96-8] **(3ae)**

^{1}H NMR (300 MHz, $CDCl_3$)

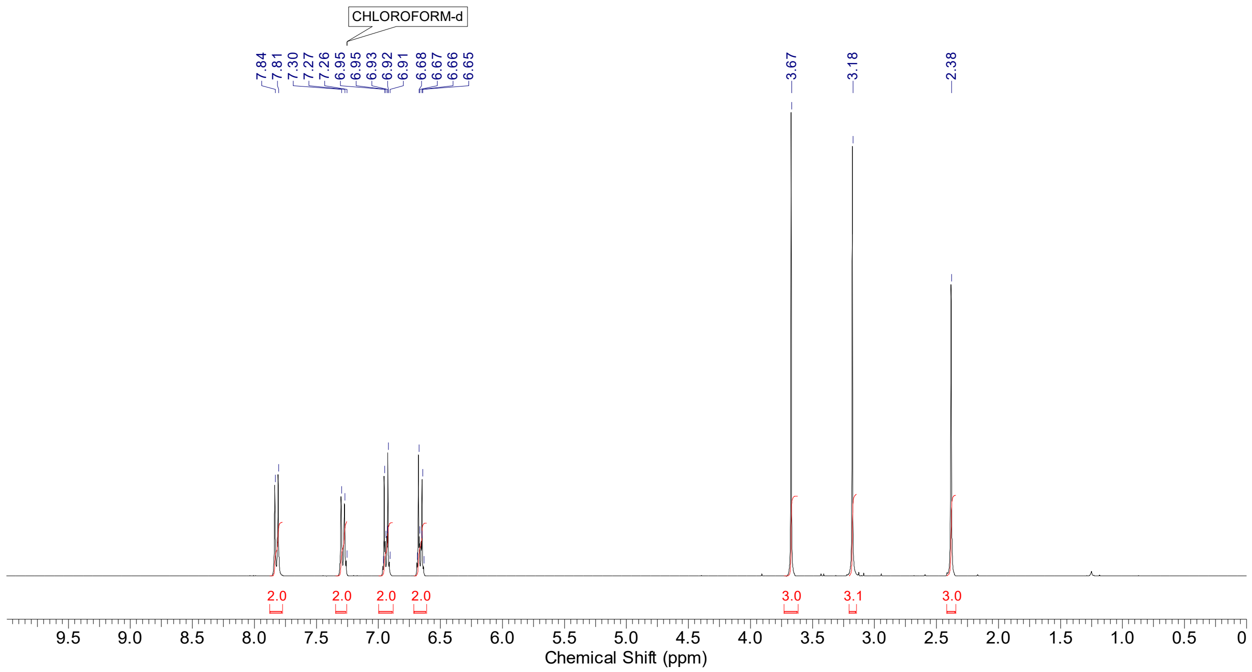

^{13}C NMR (75 MHz, $CDCl_3$)

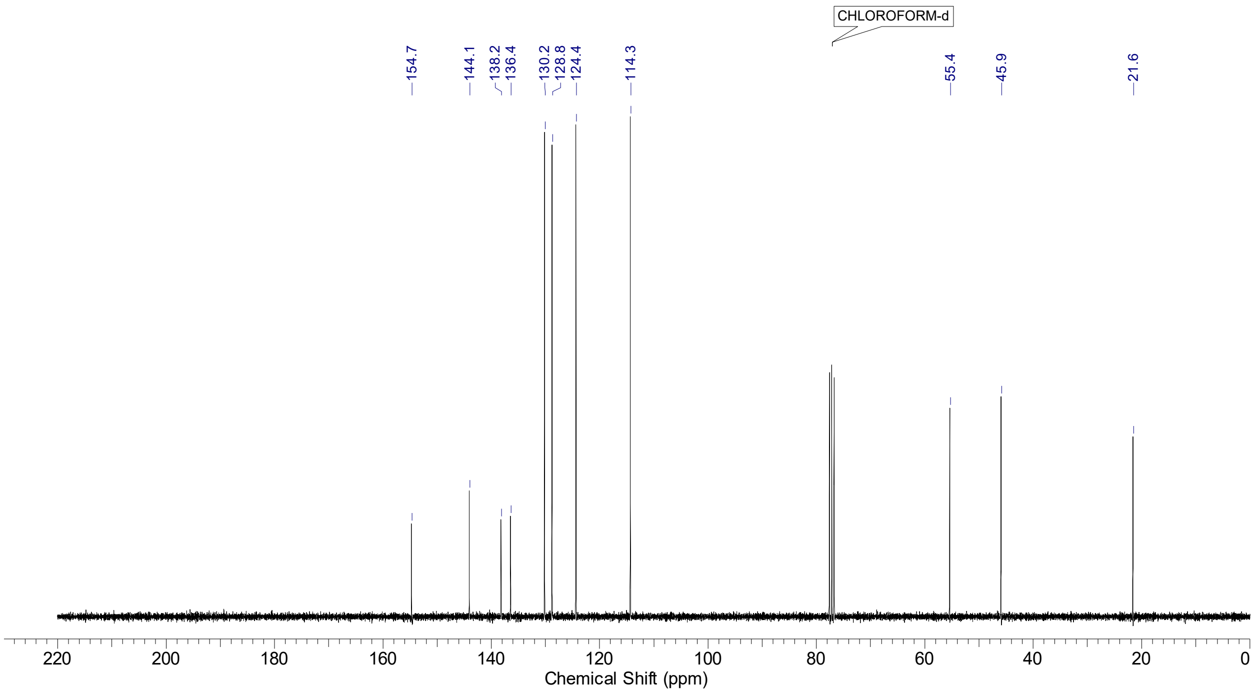

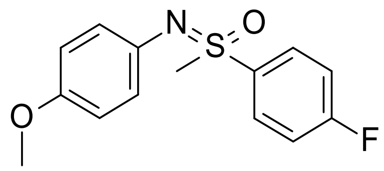

(4-fluorophenyl)-(4-methoxyphenyl)imino-methyl-oxo-λ⁶-sulfane **(3af)**

^{1}H NMR (300 MHz, $CDCl_3$)

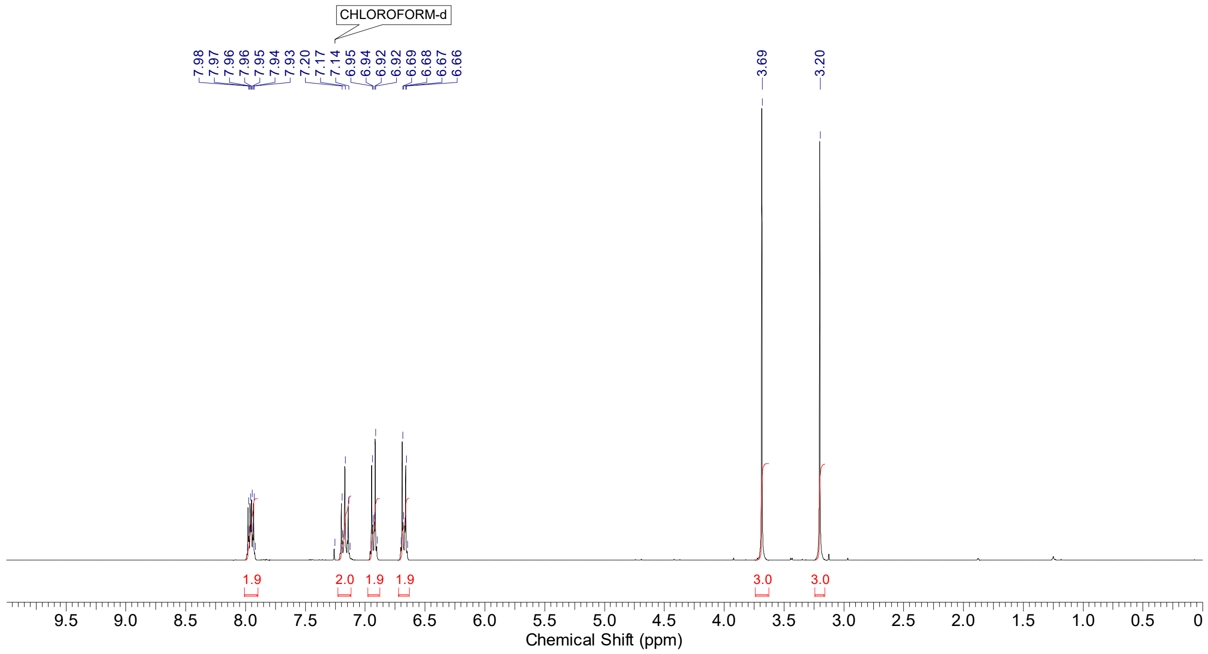

^{13}C NMR (75 MHz, $CDCl_3$)

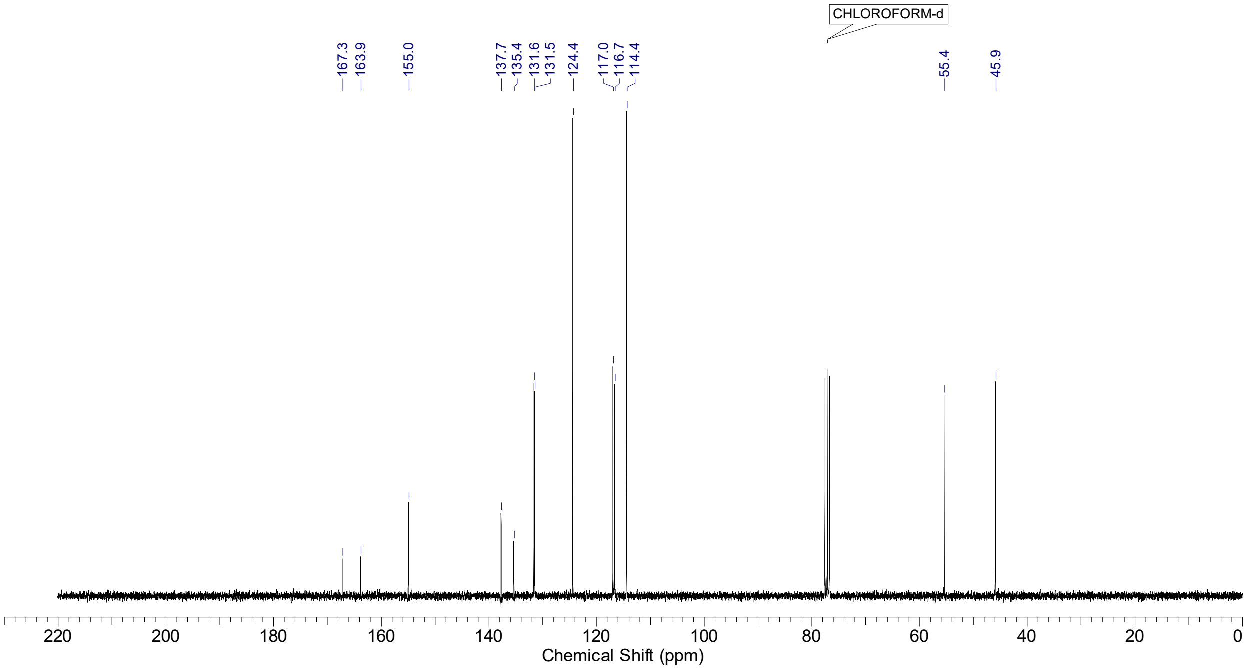

^{19}F NMR (76 MHz, CDCl3, $C_6H_4F_2$)

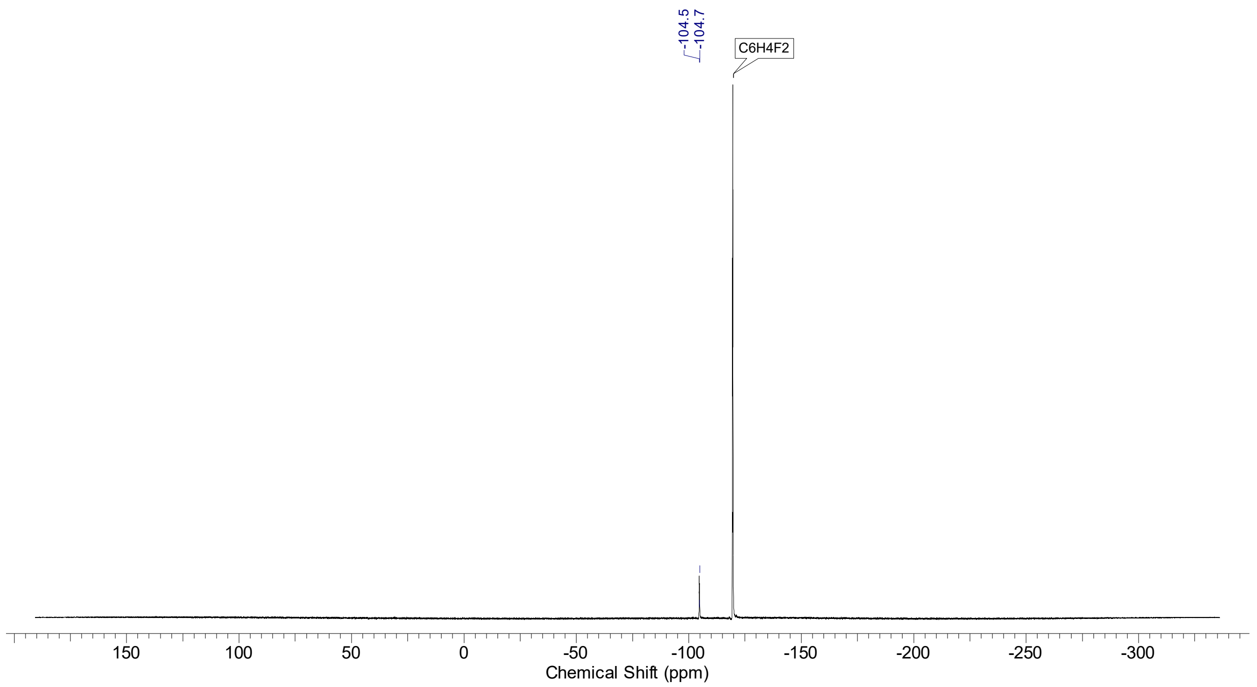

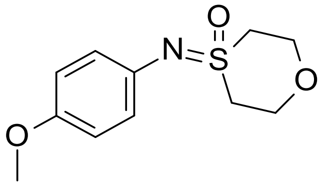

4-(4-methoxyphenyl)imino-1,4-oxathiane 4-oxide **(3ag)**

^{1}H NMR (300 MHz, $CDCl_3$)

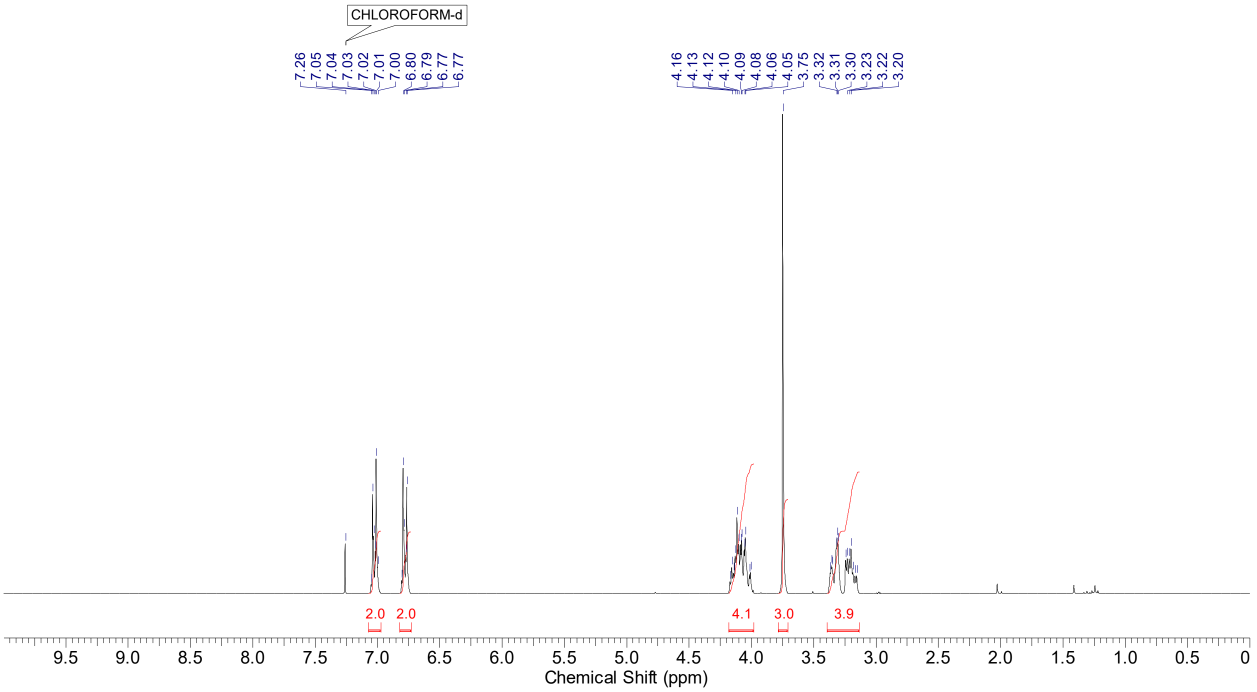

^{13}C NMR (75 MHz, $CDCl_3$)

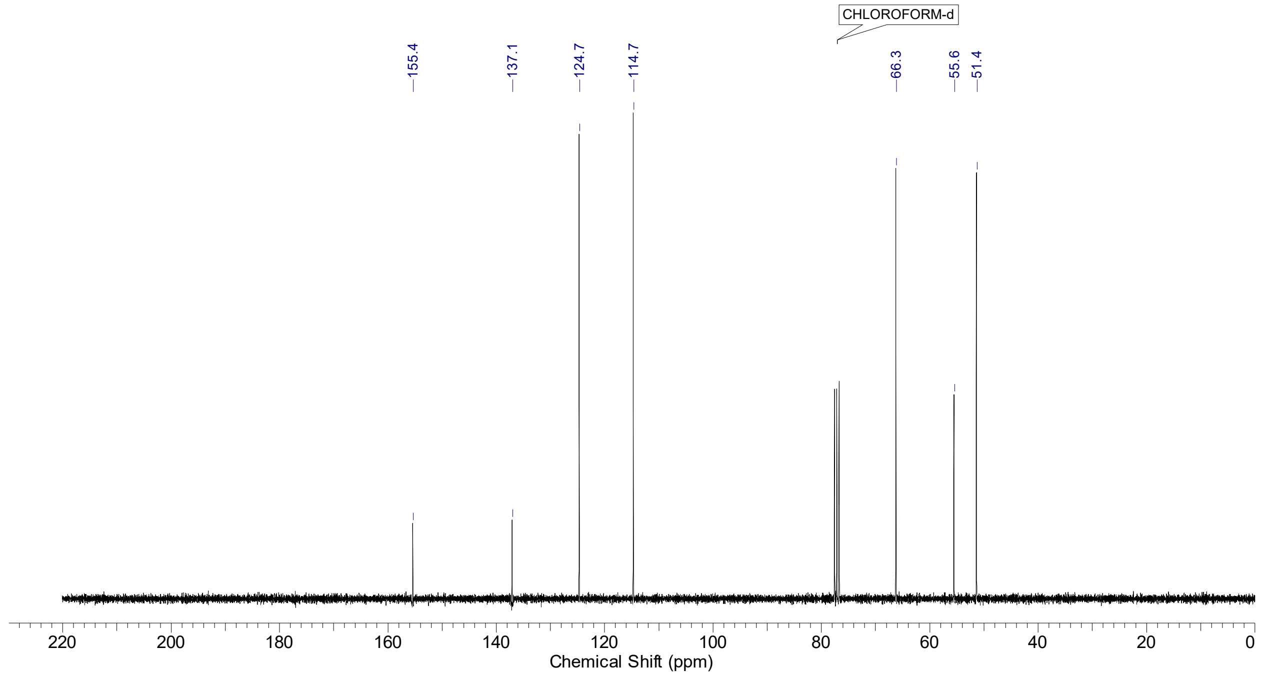

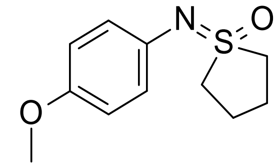

1-(4-methoxyphenyl)iminothiolane 1-oxide **(3ah)**

^{1}H NMR (300 MHz, $CDCl_3$)

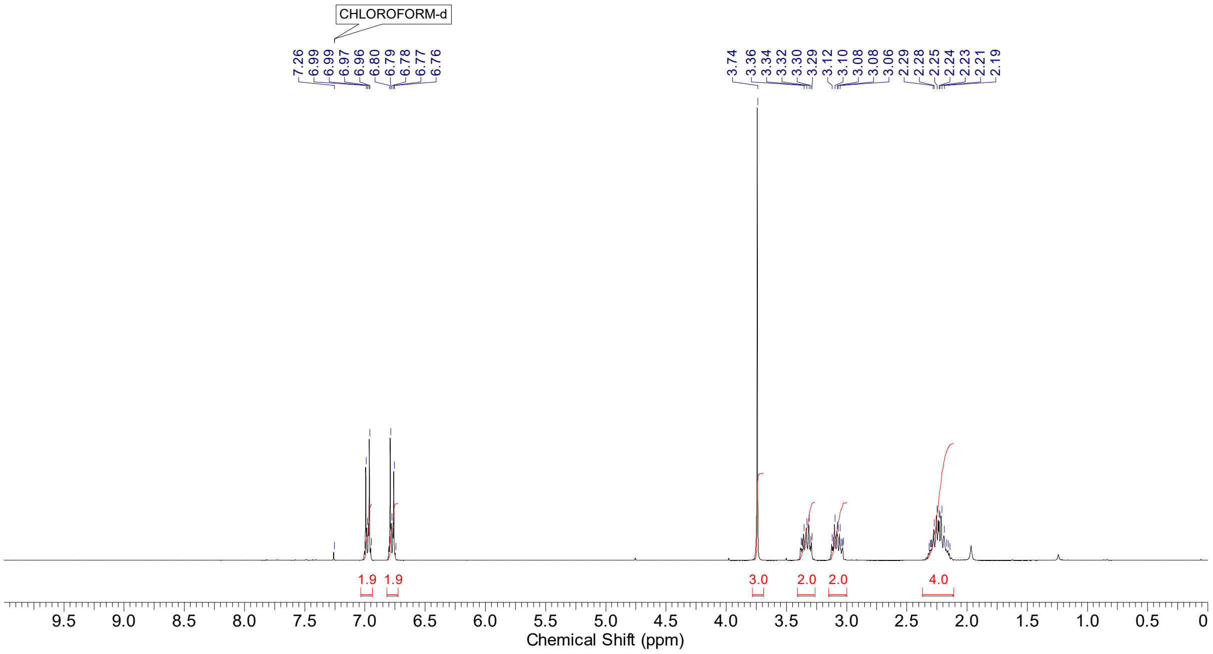

^{13}C NMR (75 MHz, $CDCl_3$)

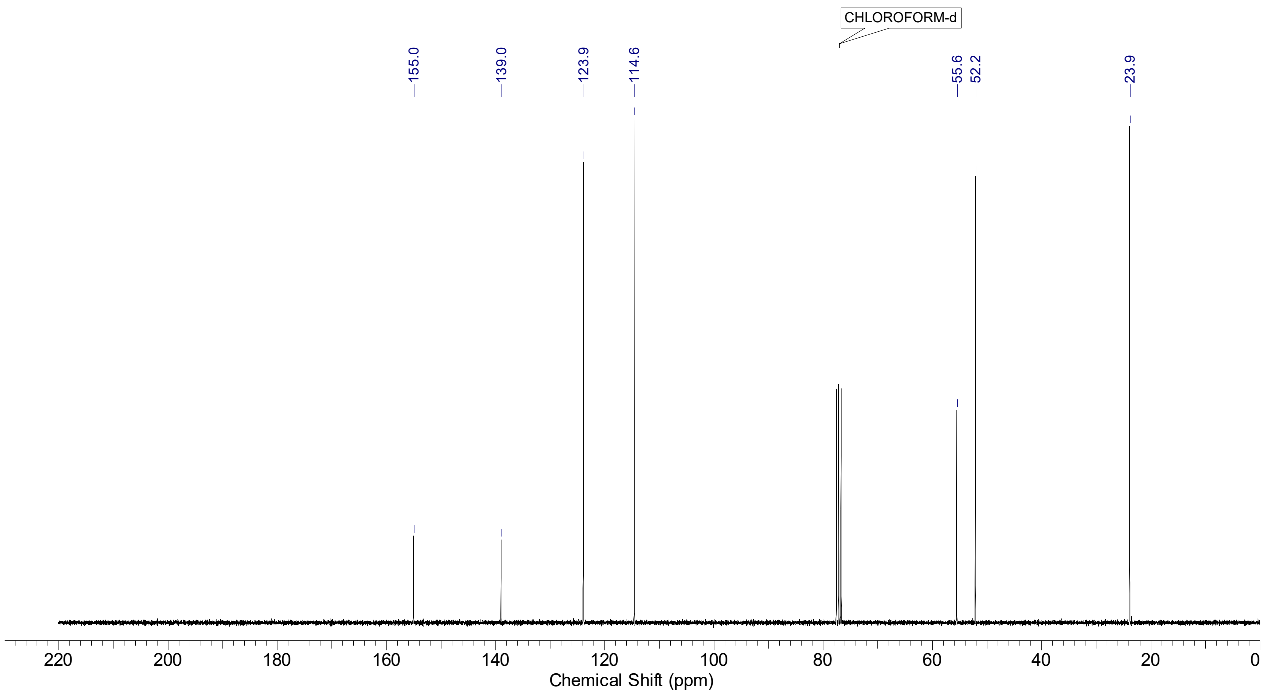

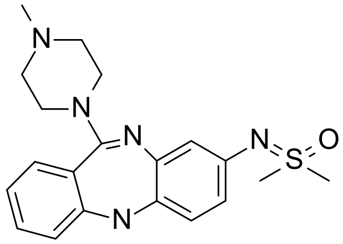

dimethyl-[[6-(4-methylpiperazin-1-yl)-11H-benzo[b][1,4]benzodiazepin-3-yl]imino]-oxo-λ⁶-sulfane **(13)**

^{1}H NMR (400 MHz, $CDCl_3$) (*ethyl acetate impurity)

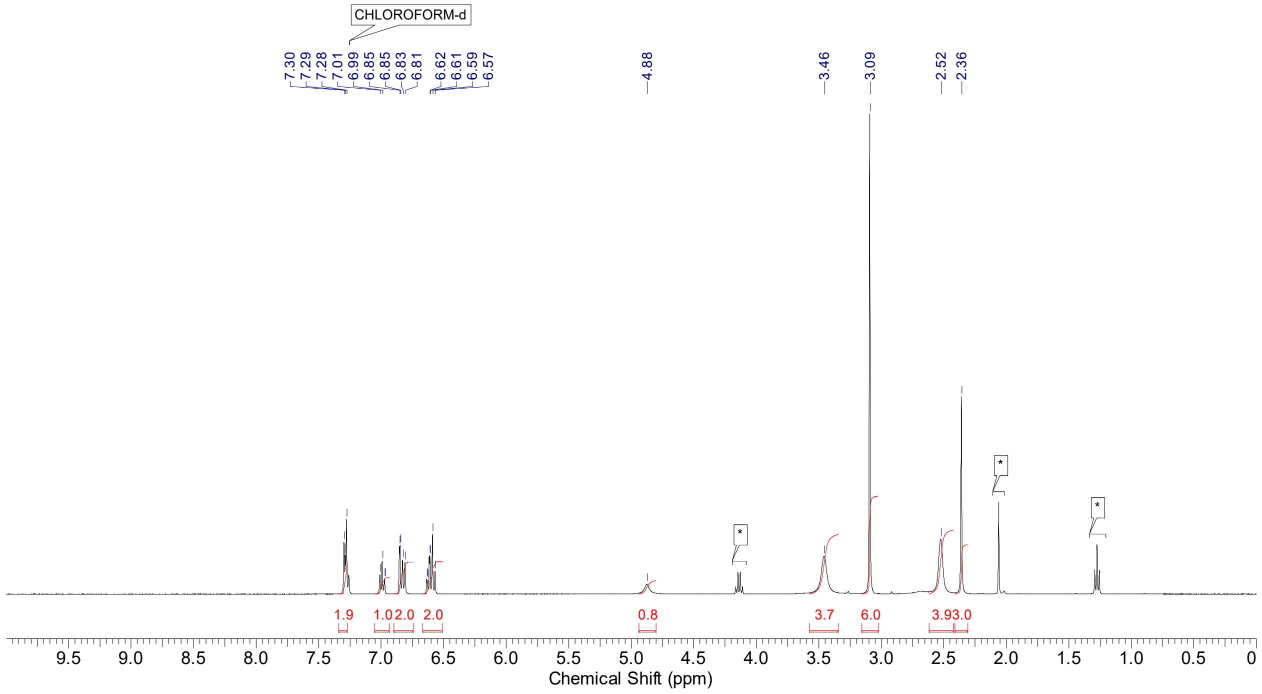

^{13}C NMR (101 MHz, $CDCl_3$) (*ethyl acetate impurity)

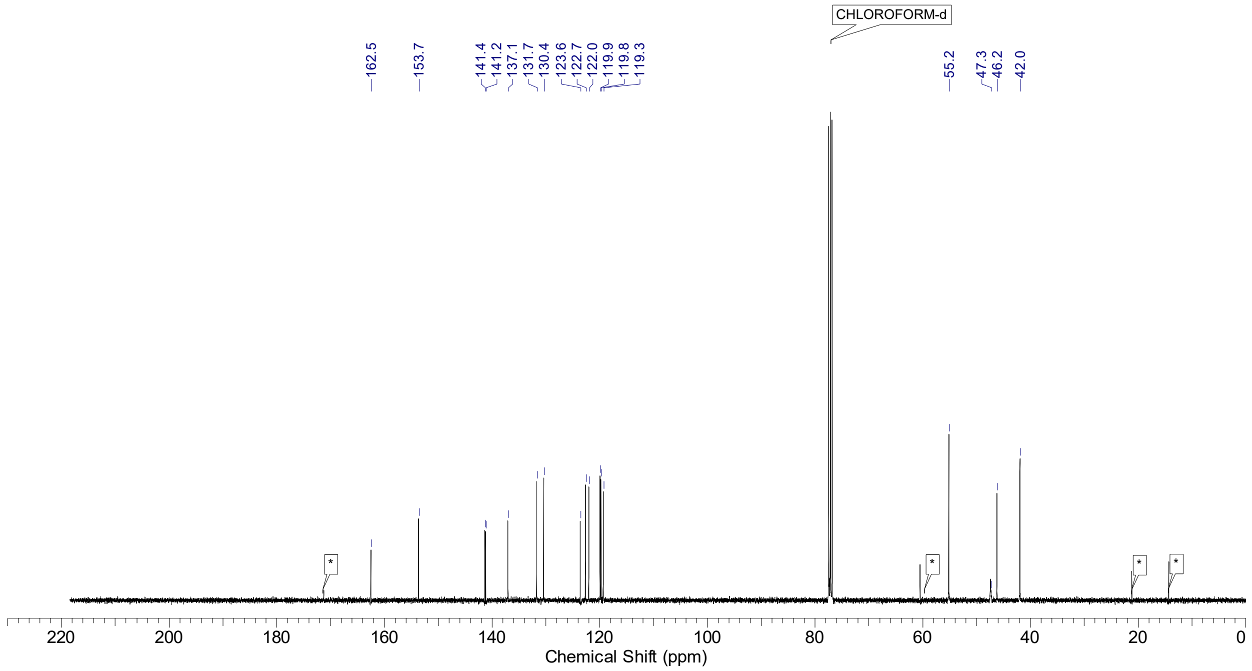

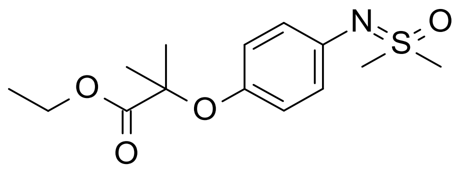

ethyl 2-[4-[[dimethyl(oxo)-λ⁶-sulfanylidene]amino]phenoxy]-2-methyl-propanoate **(14)**

^{1}H NMR (300 MHz, $CDCl_3$)

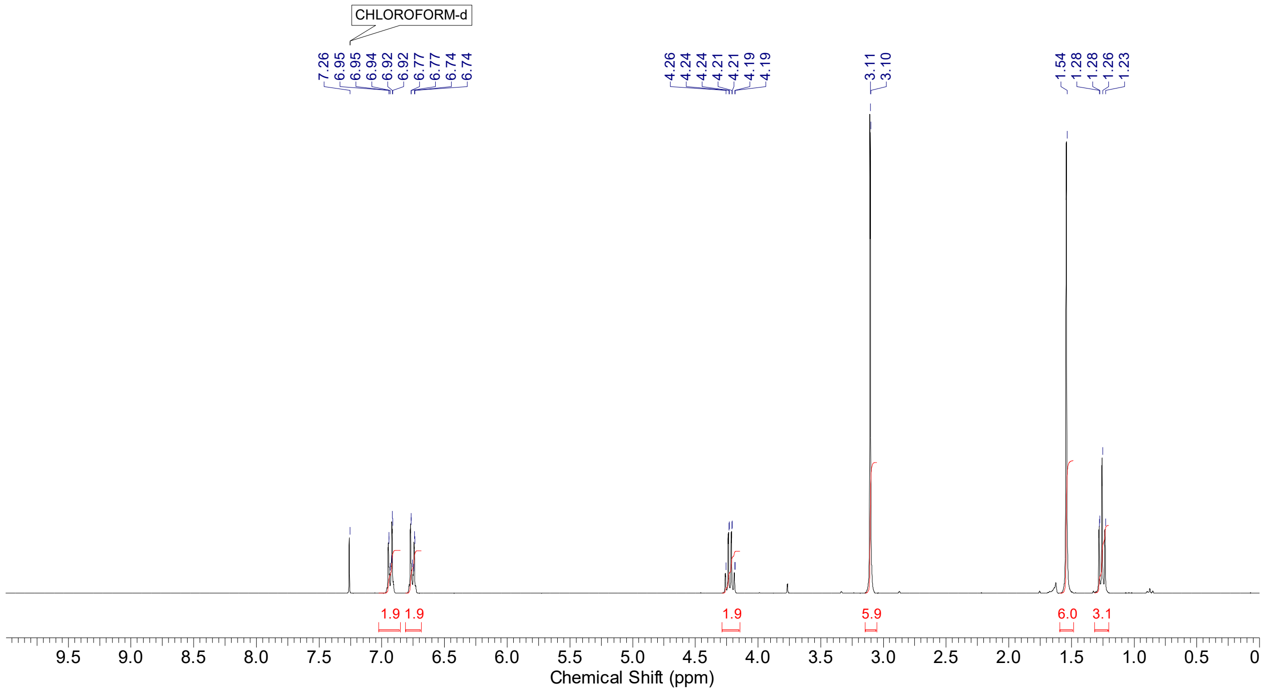

^{13}C NMR (75 MHz, $CDCl_3$)

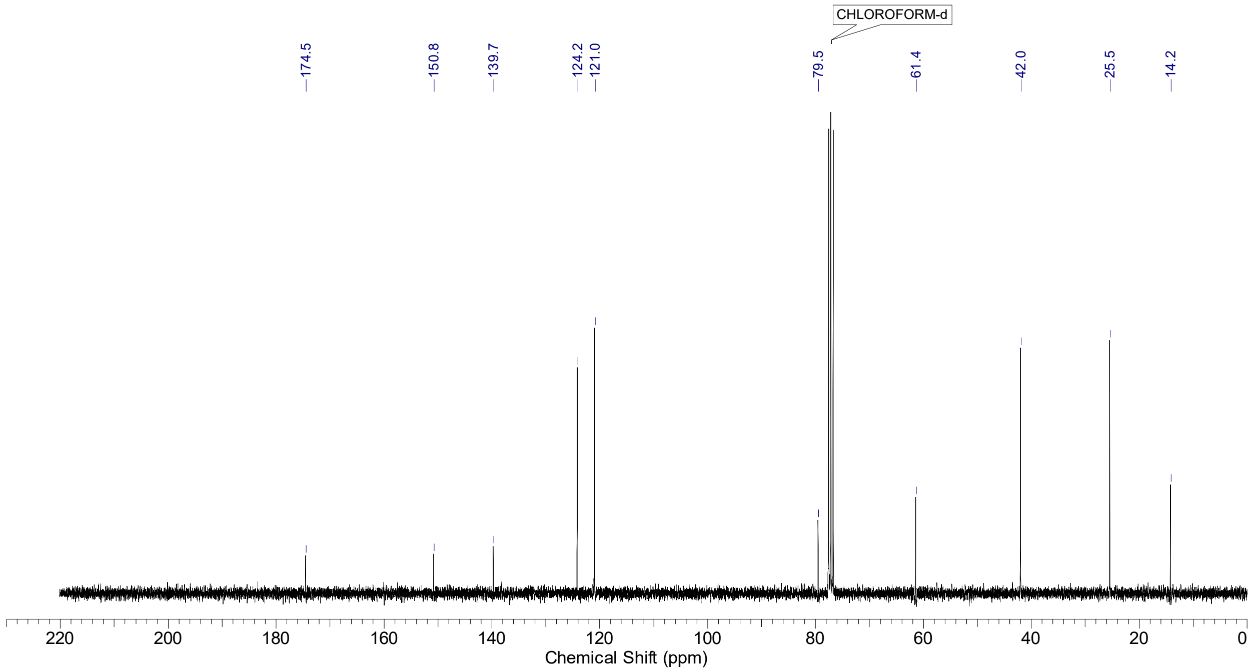

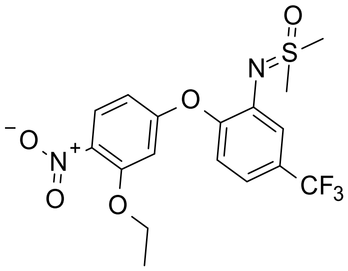

[2-(3-ethoxy-4-nitro-phenoxy)-5-(trifluoromethyl)phenyl]imino-dimethyl-oxo-λ⁶-sulfane **(15)**

^{1}H NMR (400 MHz, $CDCl_3$)

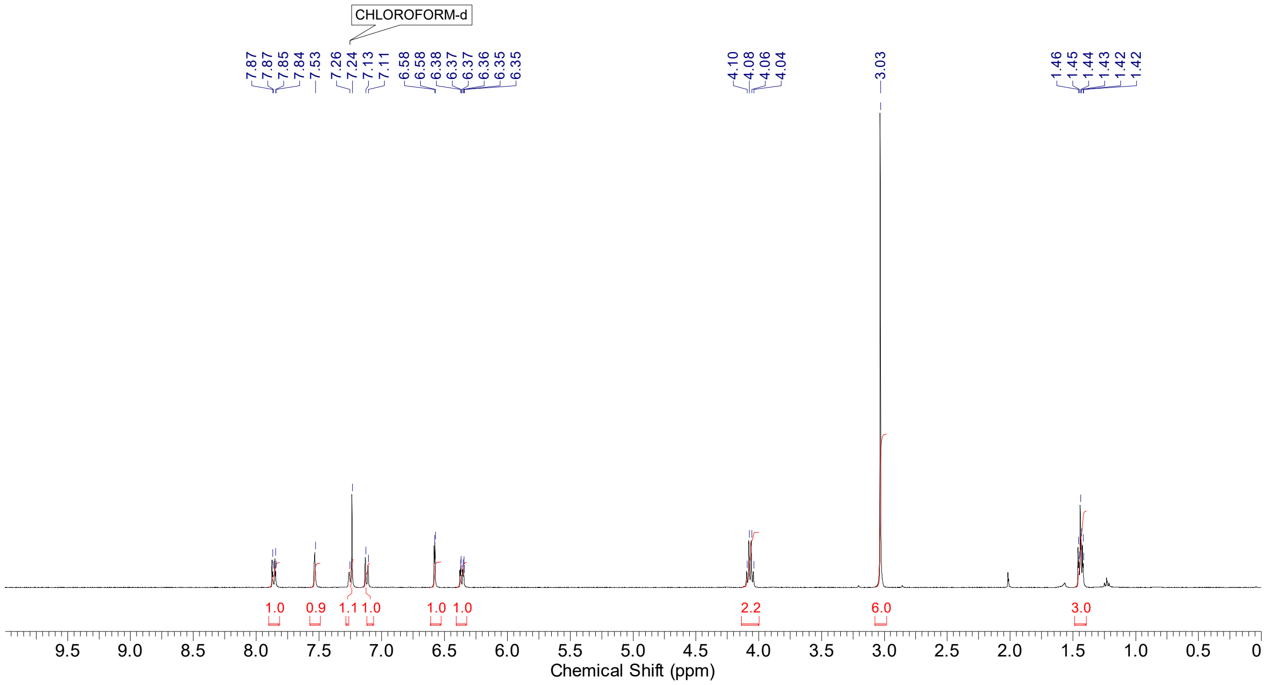

^{13}C NMR (101 MHz, $CDCl_3$)

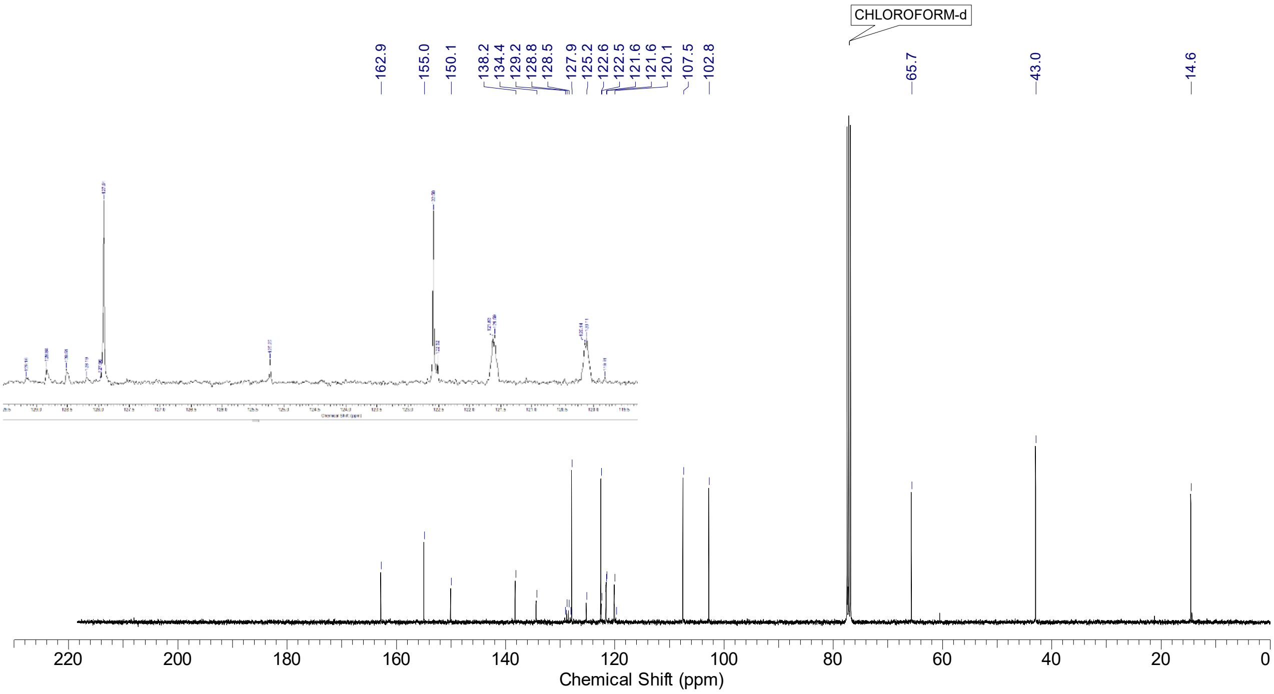

^{19}F NMR (76 MHz, CDCl3, $C_6H_4F_2$)

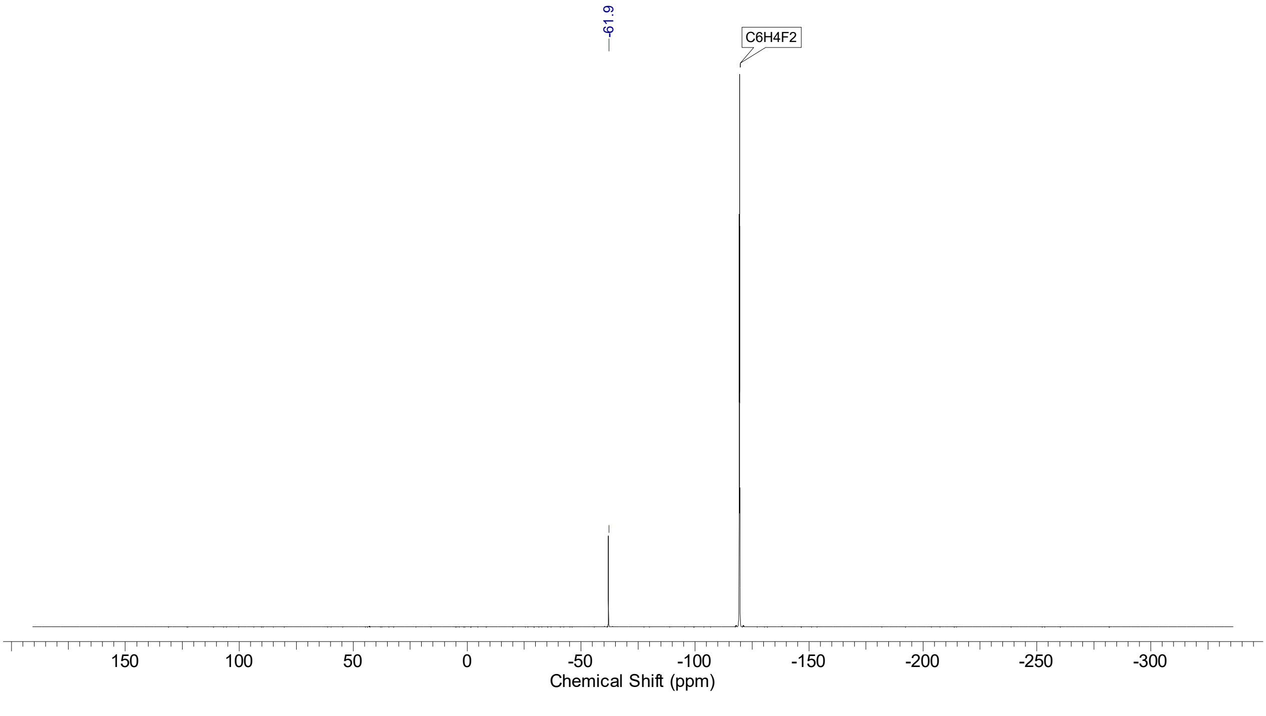

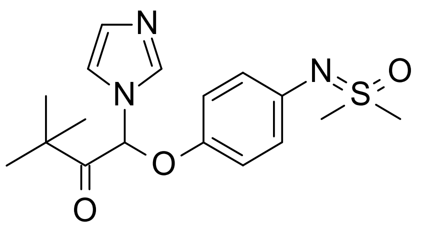

1-[4-[[dimethyl(oxo)-λ^6-sulfanylidene]amino]phenoxy]-1-imidazol-1-yl-3,3-dimethyl-butan-2-one **(16)**

^{1}H NMR (300 MHz, $CDCl_3$)

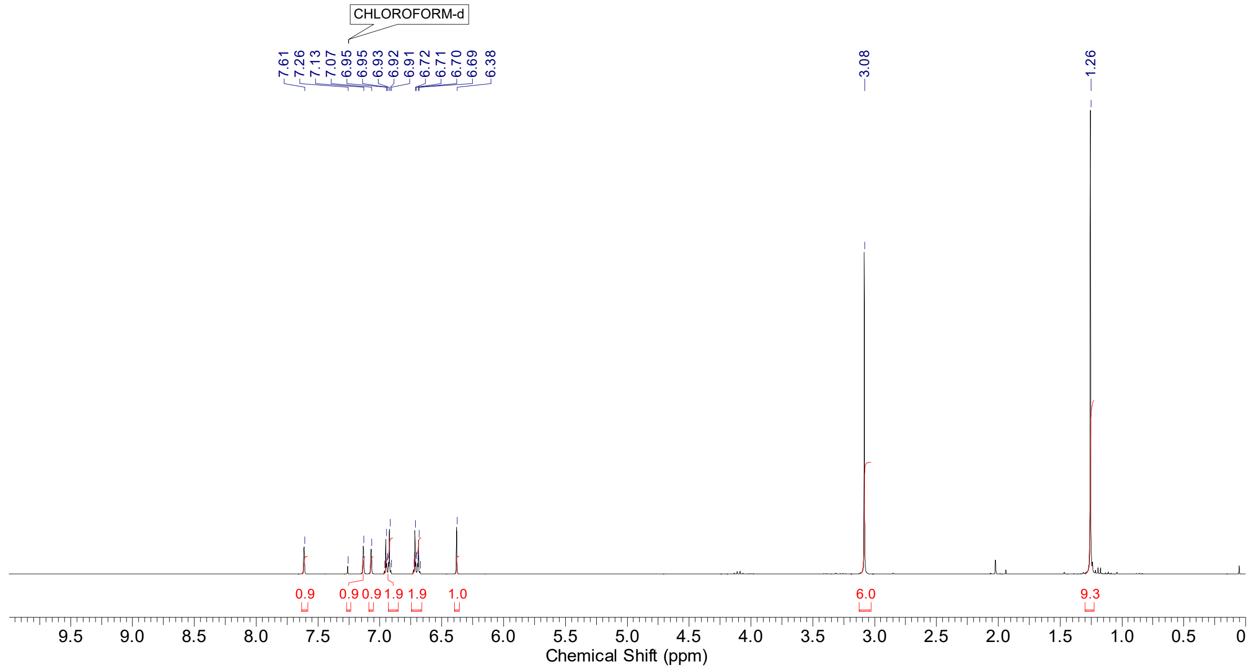

^{13}C NMR (75 MHz, $CDCl_3$)

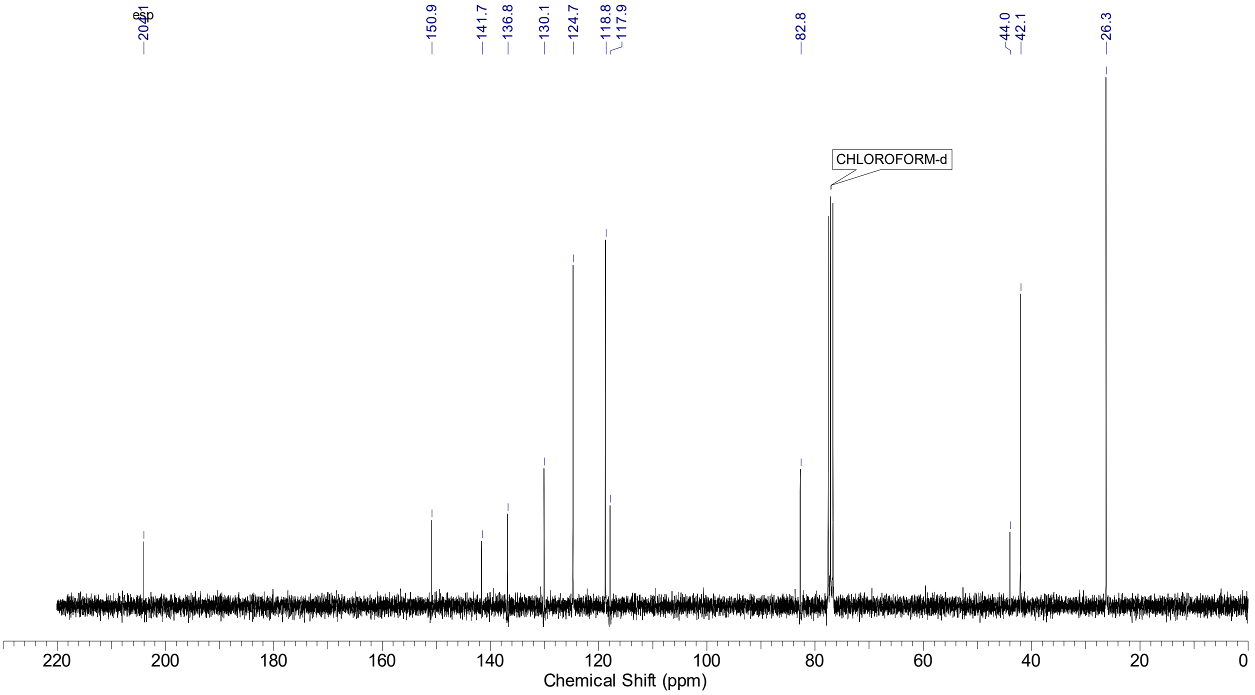

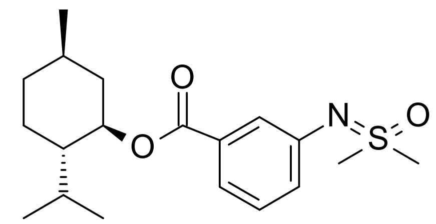

[(1R,2S,5R)-2-isopropyl-5-methyl-cyclohexyl]3-[[dimethyl(oxo)-λ^6sulfanylidene]amino] benzoate **(17)**

^{1}H NMR (300 MHz, $CDCl_3$)

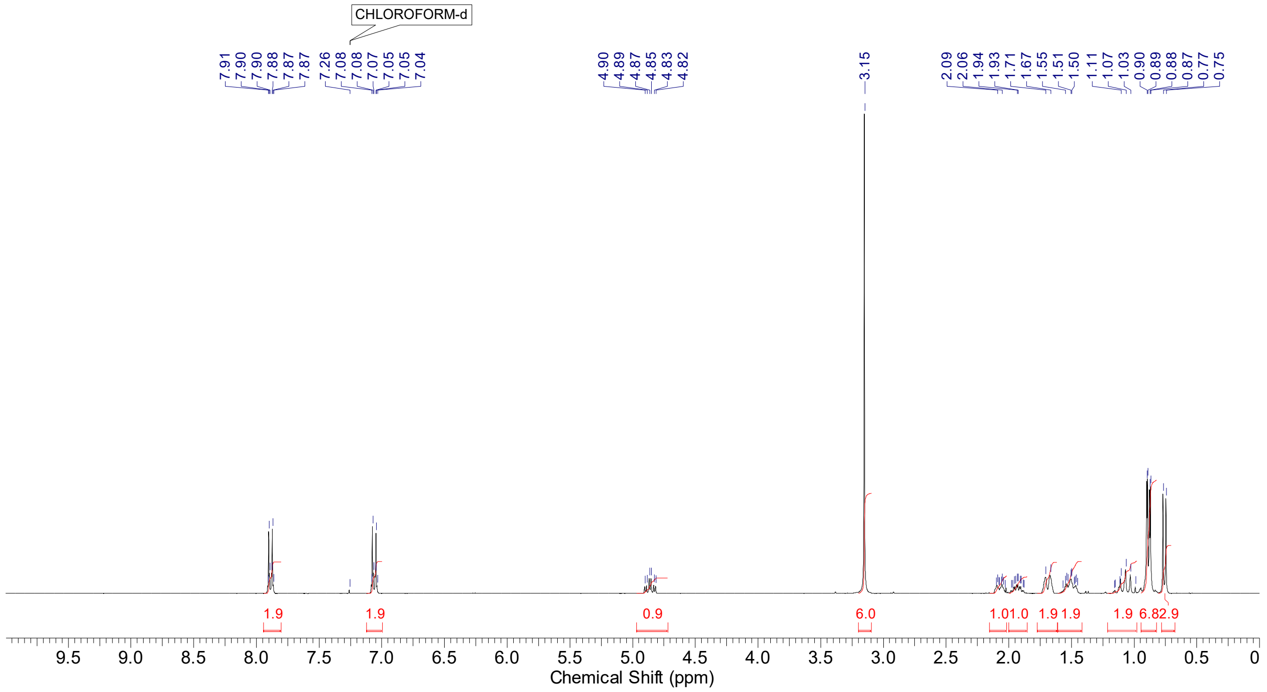

^{13}C NMR (75 MHz, $CDCl_3$)

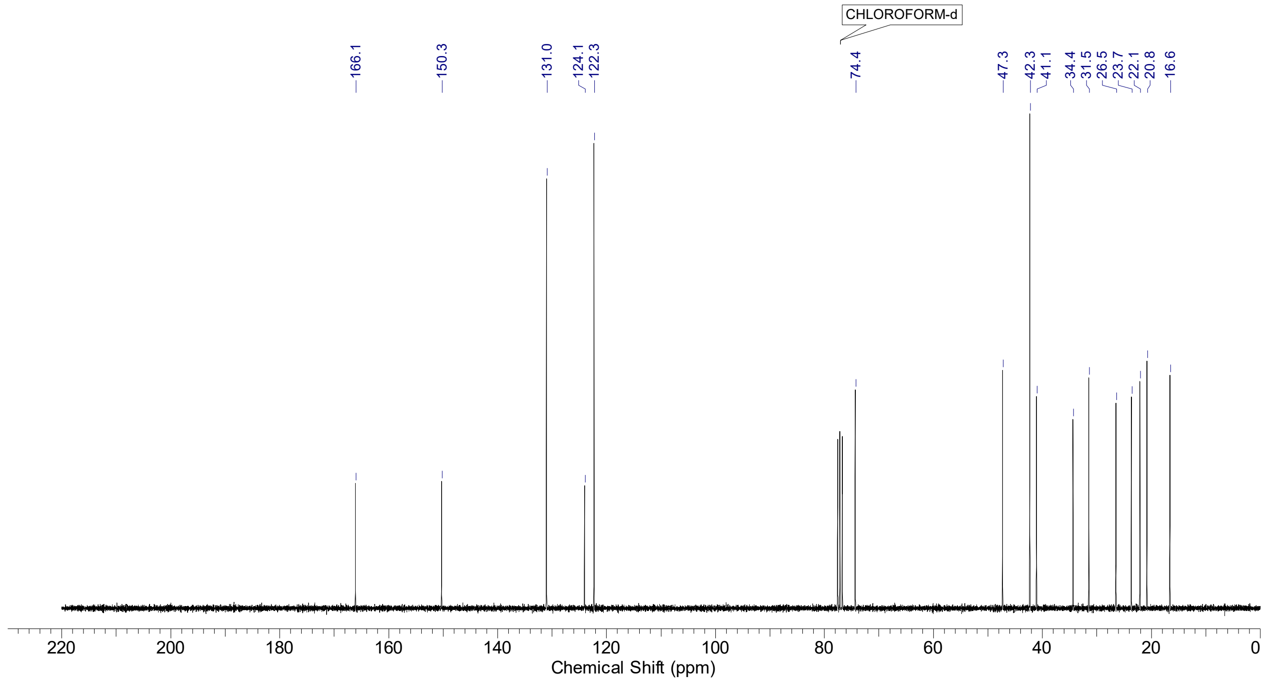

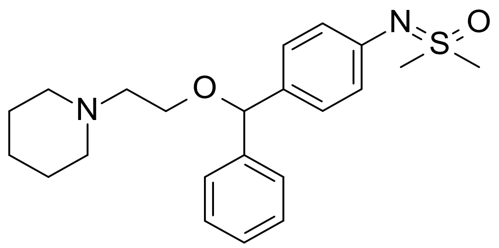

dimethyl-oxo-[4-[phenyl-[2-(1-piperidyl)ethoxy]methyl]phenyl]imino-λ⁶-sulfane **(18)**

^{1}H NMR (300 MHz, $CDCl_3$)

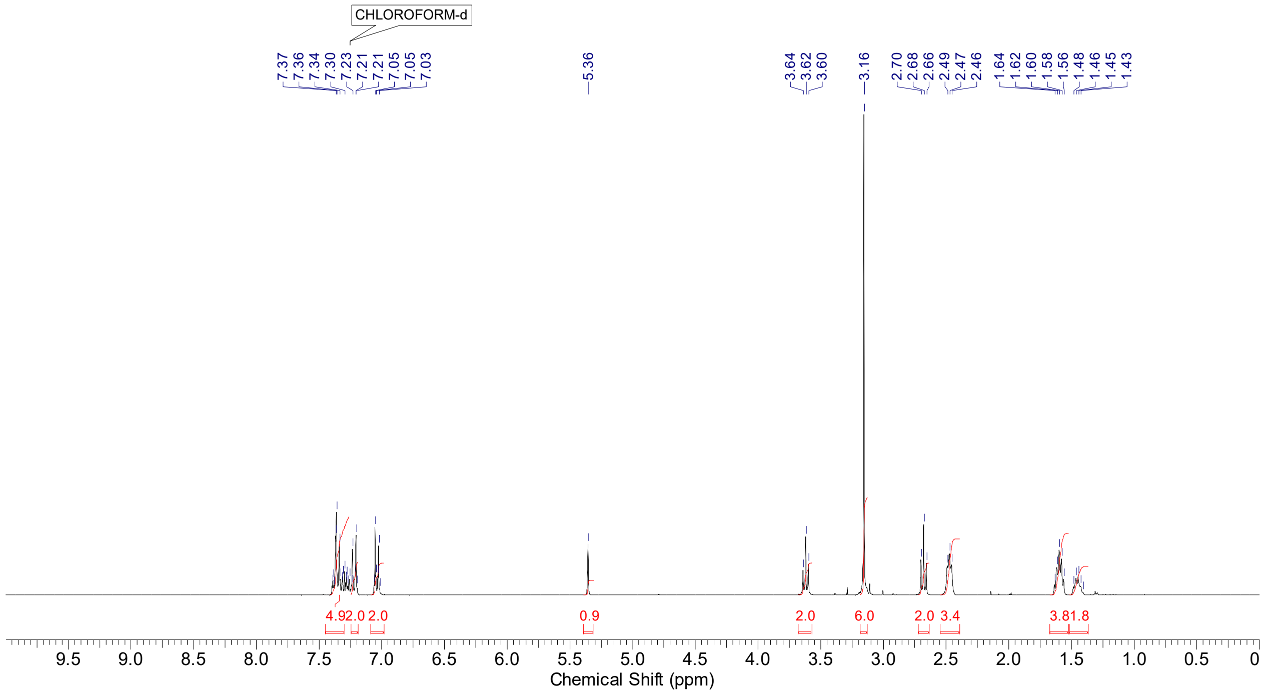

^{13}C NMR (75 MHz, $CDCl_3$)

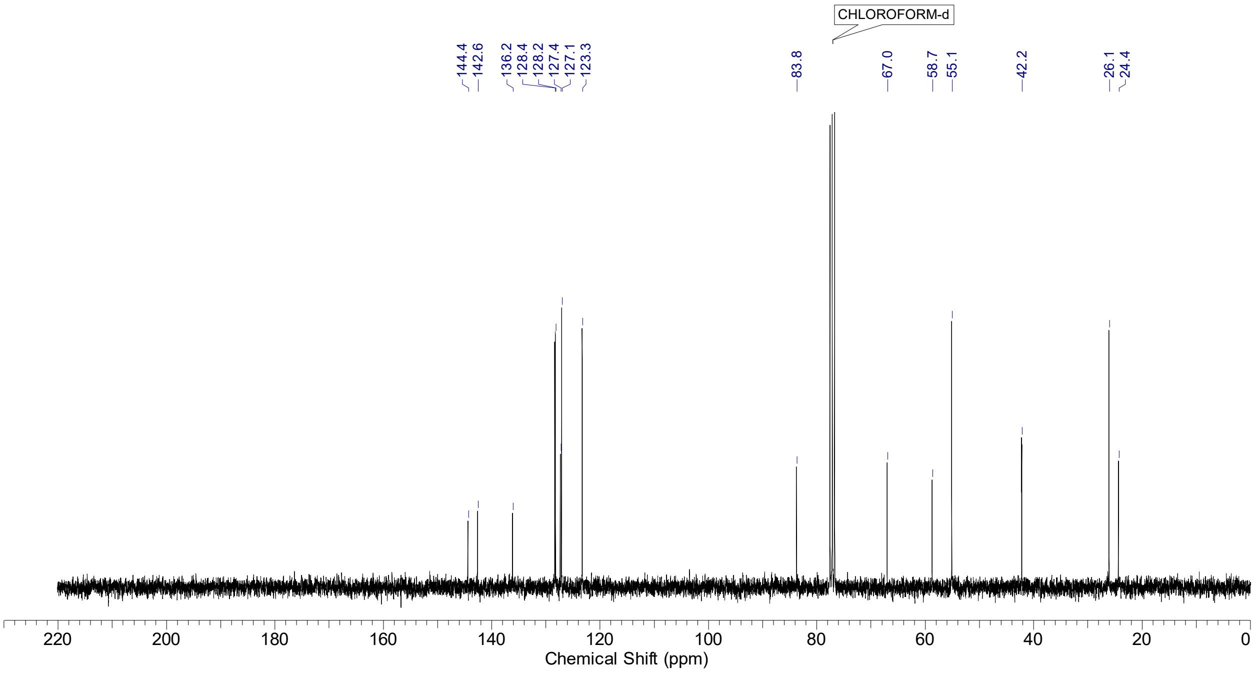

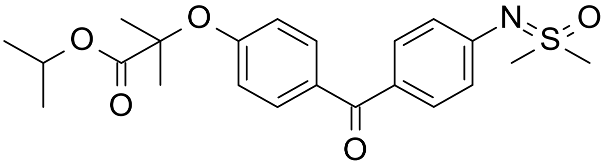

Isopropyl2-[4-[4-[[dimethyl(oxo)-λ⁶-sulfanylidene]amino]benzoyl]phenoxy]-2-methyl-propanoate **(19)**

^{1}H NMR (300 MHz, $CDCl_3$) (*ethyl acetate impurity)

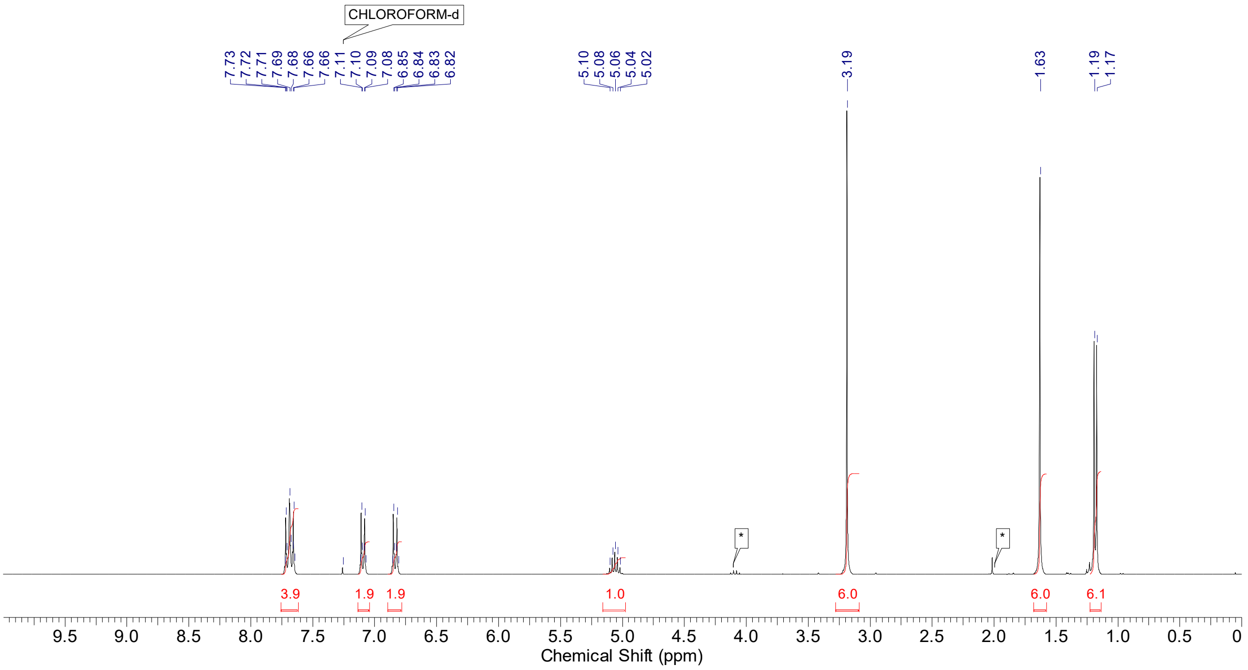

^{13}C NMR (75 MHz, $CDCl_3$)

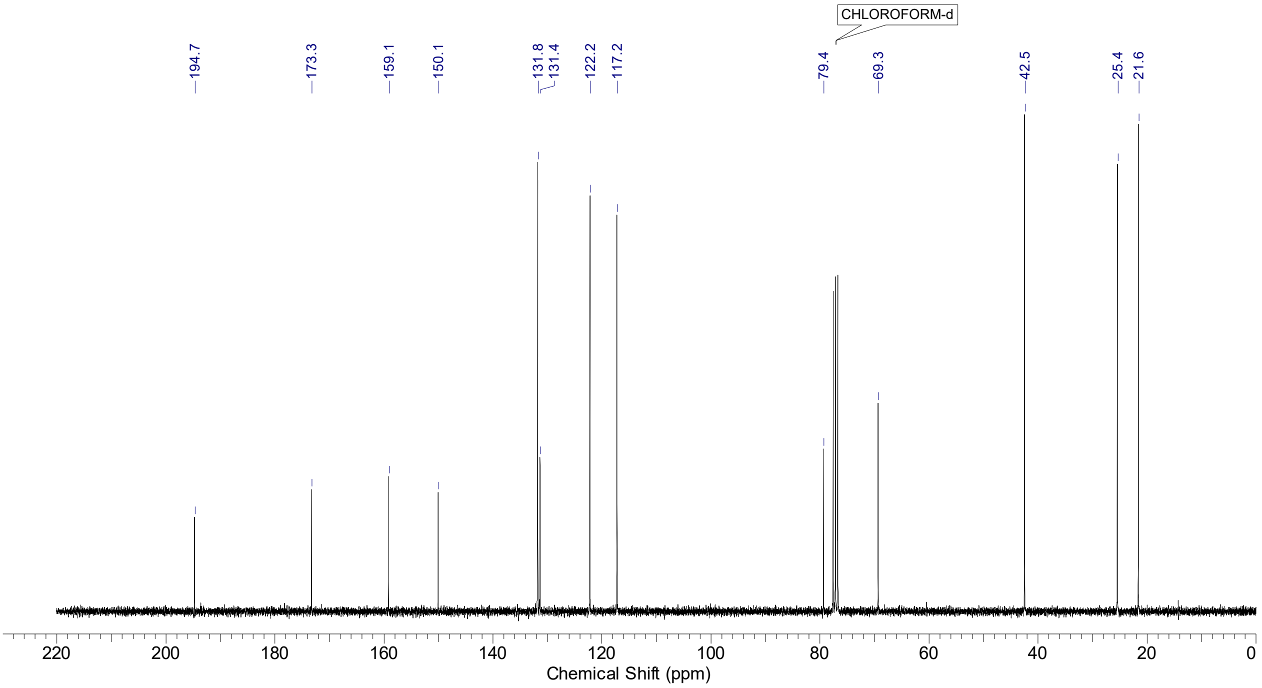

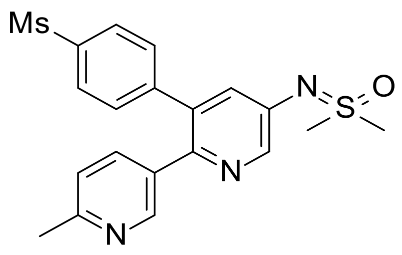

dimethyl-[[6-(6-methyl-3-pyridyl)-5-(4-methylsulfonylphenyl)-3-pyridyl]imino]-oxo-λ⁶-sulfane **(20)**

^{1}H NMR (300 MHz, $CDCl_3$)

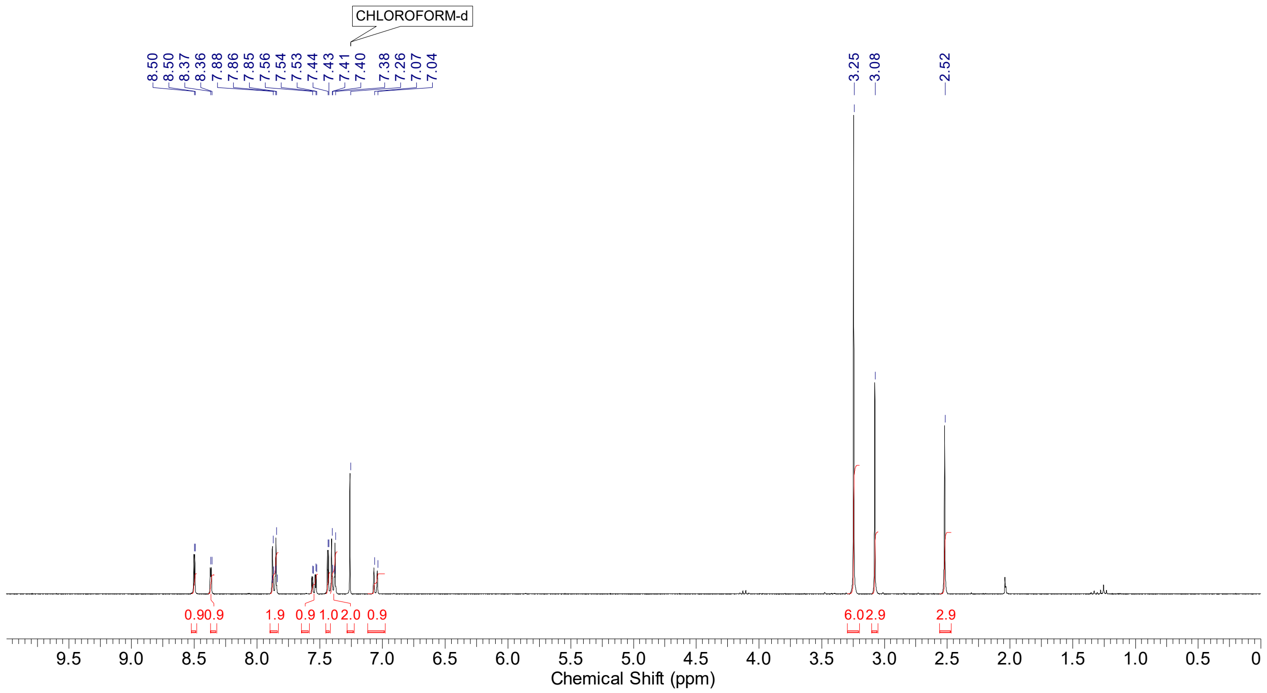

^{13}C NMR (75 MHz, $CDCl_3$)

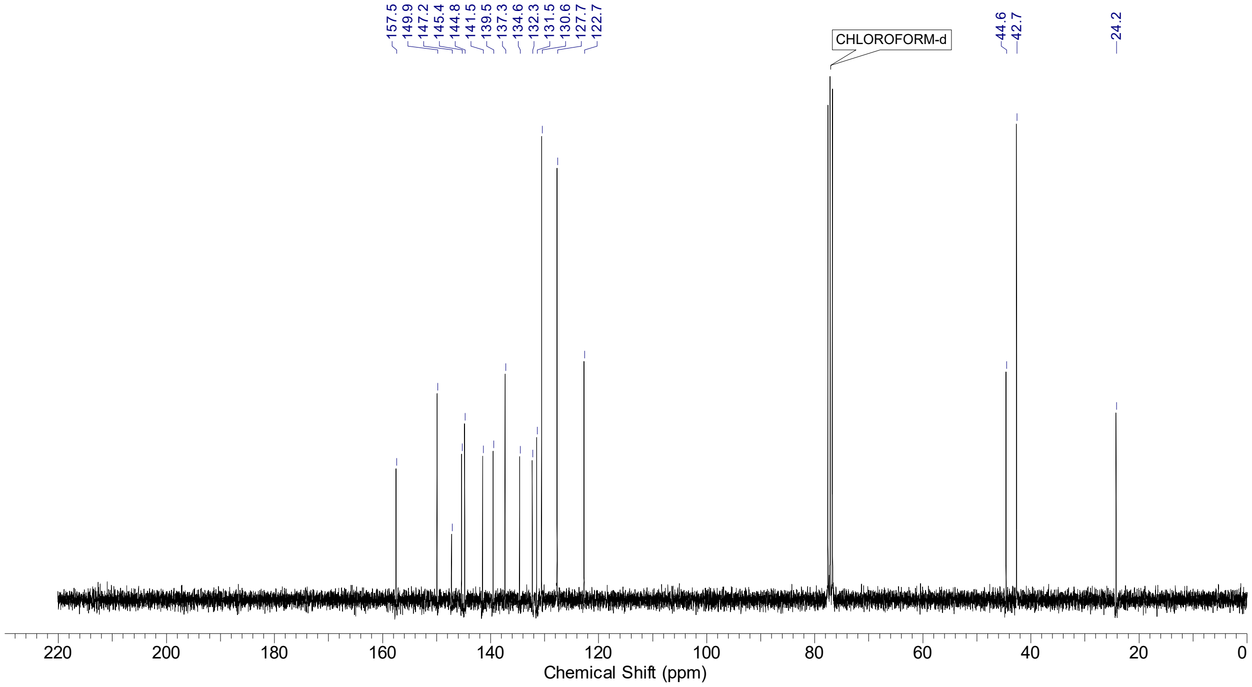

8. References:

[1] S. A. Fisher, C. M. Simon, P. L. Fox, M. J. Cotnam, P. L. DeRoy, M. Stradiotto, "Thermal Nickel-Catalyzed N-Arylation of NH-Sulfoximines with (Hetero)aryl Chlorides Enabled by PhPAd-DalPhos Ligation" *Org. Lett.* **2024**, *26*, 1326–1331.

[2] S. Hande, A. Mfuh, S. Throner, Y. Wu, Q. Ye, X. Zheng, "Photoredox mediated CN cross coupling of sulfoximines with aryl iodides" *Tetrahedron Letters* **2019**, *60*, 151100.

[3] J. Düker, M. Philipp, T. Lentner, J. A. Cadge, J. E. A. Lavarda, R. M. Gschwind, M. S. Sigman, I. Ghosh, B. König, "Cross-Coupling Reactions with Nickel, Visible Light, and tert-Butylamine as a Bifunctional Additive" *ACS Catal.* **2025**, *15*, 817–827.

[4] B. Vaddula, J. Leazer, R. S. Varma, "Copper-Catalyzed Ultrasound-Expedited N-Arylation of Sulfoximines using Diaryliodonium Salts" *Advanced Synthesis & Catalysis* **2012**, *354*, 986–990.

[5] M. Song, L. Zhang, D. Wei, Y. He, J. Jia, H. Li, B. Yuan, "Ultrafast N-arylation of sulfoximines enabled by micellar catalysis in water" *Green Chem.* **2022**, *24*, 6119–6124.

[6] C. Bolm, J. P. Hildebrand, "Palladium-Catalyzed N-Arylation of Sulfoximines with Aryl Bromides and Aryl Iodides" *J. Org. Chem.* **2000**, *65*, 169–175.

[7] J. Brandt, H.-J. Gais, "An efficient resolution of (±)-*S*-methyl-*S*-phenylsulfoximine with (+)-10-camphorsulfonic acid by the method of half-quantities" *Tetrahedron: Asymmetry* **1997**, *8*, 909–912.

9. Author Contributions:

The concept of the Pd-catalyzed N-Arylation of Sulfoximines was developed by S.M. Reactions were carried out by S.M., M.P., K.S. including isolation and characterization of products. S.M. performed mechanistic investigations. N.V.T. performed X-ray crystallography of the Pd complexes. S.M and L.J.G. took care of the project administration. L.J.G. supervised the project. S.M. and L.J.G. wrote the manuscript draft, which was revised by all authors.
